# Taking electrodecarboxylative etherification beyond Hofer–Moest using a radical C–O coupling strategy

**DOI:** 10.1038/s41467-020-18275-1

**Published:** 2020-09-02

**Authors:** Ángel Manu Martínez, Davit Hayrapetyan, Tim van Lingen, Marco Dyga, Lukas J. Gooßen

**Affiliations:** Fakultät für Chemie und Biochemie, Ruhr-Universität Bochum, Universitätsstraße 150, 44801 Bochum, Germany

**Keywords:** Chemistry, Electrochemistry, Organic chemistry

## Abstract

Established electrodecarboxylative etherification protocols are based on Hofer-Moest-type reaction pathways. An oxidative decarboxylation gives rise to radicals, which are further oxidised to carbocations. This is possible only for benzylic or otherwise stabilised substrates. Here, we report the electrodecarboxylative radical-radical coupling of lithium alkylcarboxylates with 1-hydroxybenzotriazole at platinum electrodes in methanol/pyridine to afford alkyl benzotriazole ethers. The substrate scope of this electrochemical radical coupling extends to primary and secondary alkylcarboxylates. The benzotriazole products easily undergo reductive cleavage to the alcohols. They can also serve as synthetic hubs to access a wide variety of functional groups. This reaction prototype demonstrates that electrodecarboxylative C–O bond formation can be taken beyond the intrinsic substrate limitations of Hofer-Moest mechanisms.

## Abstract (web summary)

Since its discovery, the Hofer-Moest mechanism strongly limits the scope of electrochemical decarboxylative etherification. Here, the authors report the electrochemical conversion of carboxylic acids into alkyl benzotriazole ethers expanding the scope to primary and secondary acids beyond the boundaries of Hofer-Moest-type reactions.

## Introduction

Oxygen-containing substituents are abundant in natural products^1^, pharmaceuticals^2^ and functional materials^3^, and efficient synthetic entries are highly sought-after. Decarboxylative C–O bond formation is a particularly attractive synthetic strategy since carboxylic acids are widely available in great structural diversity^4^. Thermal and photochemical strategies for decarboxylative C–O bond formation, including metal-mediated oxidative decarboxylations using Mn^III^^5,6^, In^III^^7^, Tl^III^^8^, Pb^IV^^9,10^ or Ce^IV^^11,12^, as well as precious metal-catalysed procedures such as Ru^13,14^, Ir^15,16^, Ag^17,18^ or Au^19^, are relatively well developed. Photochemical reaction variants proceed under mild conditions but require expensive photocatalysts or pre-functionalised substrates (i.e. redox-active esters)^20–24^.

**Fig. 1:**
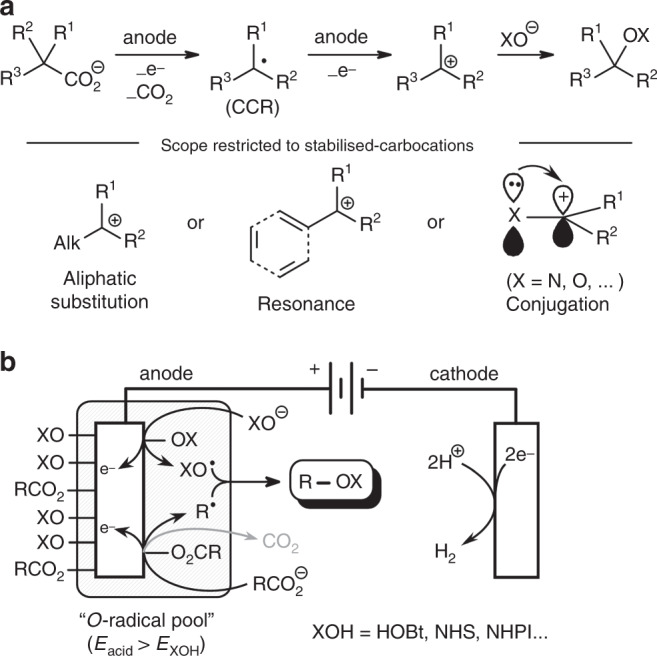
Strategies for electrochemical decarboxylative C–O bond formation. **a** Hofer–Moest electrodecarboxylative C–O bond formation. **b** Electrodecarboxylative radical C–O coupling.

Electrosynthetic concepts have a long history^25^, with contributions, e.g. by Shono^26^, Lund^27^, Torii^28^, Osa^29^ or Grimshaw^30^, but only recently shifted back into the focus of method development^31–35^. In the context of C–O bond formation, electrodecarboxylative strategies may open up synthetic opportunities with a potentially lower environmental footprint^36^. The use of carboxylic acids in electrochemical transformations was pioneered by Faraday and Kolbe^37,38^. Anodic oxidation of carboxylic acids induces their decarboxylation with the formation of carbon-centred radicals, which swiftly undergo homocoupling with formation of alkanes^39–41^. A transfer of this sustainable concept to C–heteroatom bond formation was first described by Hofer and Moest who showed that for some carboxylates, the electrogenerated radicals are oxidised further to carbocations which can be trapped by *O*-nucleophiles (originally H_2_O and MeOH, Fig. 1a)^42,43^.

However, a century after their discovery, many challenges are still associated with Hofer–Moest reactions, some of which appear to be a direct consequence of the underlying reaction mechanism: the alkyl radicals generated at the anode are prone to Kolbe dimerisation and various other side reactions^44–46^. Further anodic oxidation of these intermediates results in highly reactive carbocations, which are prone to rearrangement, elimination or coupling with any nucleophile in their proximity^47,48^. High selectivity can only be achieved if the radical and the positive charge are stabilised by electron-donating substituents^49,50^, resonance^51,52^ or conjugation with α-*O*^53,54^, α-*N*^55,56^ or α-*S* substituents^57^. Still, the *O*-nucleophile is often added in large excess. Synthetic methods for electrodecarboxylative C–O bond formation are therefore limited to tertiary, benzylic/allylic and α-*O*/*N* substituted carboxylates, but simple alkylcarboxylates remain challenging substrates. Acetate has been converted to methanol, albeit in poor yields^58^. However, it has also been used as an inert electrolyte^59^ or oxygen nucleophile in electrodecarboxylative C–O bond formation (Fig. 1a, X = CH_3_C(O))^60,61^. In the leading reference work by Hammerich and Speiser^25^, the electrodecarboxylative synthesis of nitrate esters by Fichter is presented as a viable C–O bond formation strategy for non-stabilised carboxylates^43^. However, this method gives reasonable yields only in the conversion of succinate to ethylene glycol dinitrate (58%)^62^. For simple alkylcarboxylates, the yields were not reported or drop to below 5%^63^.

Baran et al. have recently disclosed a thoroughly optimised Hofer–Moest reaction variant^64^. Their elaborate protocol with stoichiometric silver salts as sacrificial oxidant has enabled the coupling of numerous alcohols in stoichiometric amounts. However, it stays within the intrinsic boundaries of the Hofer–Moest mechanism in that it extends only to tertiary, benzylic/allylic and α-O/N substituted carboxylates (Fig. 1a), but not to primary or secondary carboxylic acids.

A conceptually different approach is clearly required to extend electrochemical decarboxylative C–O bond formation to the full range of carboxylic acids (Fig. 1b). Our mechanistic blueprint involves the following steps: (1) Both the carboxylic acid and the oxygen nucleophile (X–OH) are deprotonated under the reaction conditions and attracted to the anode. (2) The oxygen nucleophiles are oxidised preferentially, yielding XO^•^ radicals. (3) At high current densities, some carbon radicals will also form via Kolbe decarboxylation, surrounded by the XO^•^ species. (4) C–O bond formation would then proceed via the combination of an alkyl and a XO^•^ radical. This radical coupling strategy promises to lift inherent substrate limitations regarding the carboxylate, since no stabilisation of carbocationic intermediates would be required. However, it calls for a particular oxygen reagent that would have to fulfil several prerequisites. (1) It needs to be sufficiently acidic to allow smooth deprotonation. (2) Its oxidation potential must be lower than that of carboxylates. (3) The XO^•^ radicals need to be long-lived and stable towards dimerisation. (4) Their reactivity must be sufficiently high towards alkyl radicals to intercept them before Kolbe-type recombination occurs. (5) Ideally, the reaction would lead to ether products of synthetic value and/or synthetic hubs for further derivatisation. Herein, we present the coupling of carboxylic acids with the oxygen source 1-hydroxybenzotriazole as a prototypical electrodecarboxylative C–O bond-forming reaction that implements the envisioned radical pathway. Its scope extents to non-activated primary and secondary carboxylic acids, and thus goes well beyond intrinsic limitations of Hofer–Moest-type reactions.

## Results

### Reaction optimisation

The unsolved challenges of electrodecarboxylative C–O bond formation were explored using phenylpropionic acid (**1a**, Supplementary Table 1). The distant phenyl group of **1a** is useful for product analysis and isolation, but does not significantly stabilise intermediates, e.g via an anchimeric effect. This was confirmed by comparative reactions with 3-cyclohexanepropionic acid (**1j**, 31 vs 37% yield under optimised conditions, respectively).

Attempted Hofer–Moest etherifications gave styrene and 1,4-diphenylbutane as the major products. Even when employing the oxygen coupling partner as the solvent, the selectivity for C–O bond formation did not exceed 10% (MeOH or H_2_O, NaOH, 500 mA, 30 min). We next probed Fichter’s conditions, in which potassium nitrate serves as the oxygen source^61^, but did not detect any nitrate ester product. These experiments confirmed that aliphatic carboxylates like **1a** are well outside the scope of known electrochemical C–O bond-forming strategies.

**Fig. 2:**
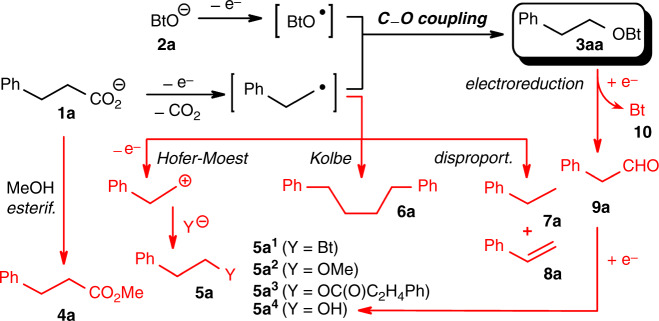
Products derived from the electrolysis of 1a and 2a in MeOH. Red is used for undesired side-products obtained during the electrolysis.

We next tested numerous potential sources of oxygen radicals in stoichiometric quantities, including pyridine *N*-oxide, TEMPO, *N*-hydroxyphthalimide and di-*tert*-butyl peroxide. None of these gave more than trace quantities of the C–O coupling product (Supplementary Table 1). To our delight, the desired reactivity was finally observed for 1-hydroxybenzotriazole (HOBt, **2a**), a reagent commonly used in peptide chemistry. However, besides the targeted benzotriazole ether **3aa**, several other products were formed. These arise, e.g. from esterification (**4a**), decarboxylative couplings with solvents, substrates, byproducts or added bases (**5a**^**i**^), Kolbe dimerisation (**6a**), disproportionation of radical intermediates (**7a, 8a**) or electroreduction of **3aa** to aldehyde **9a** and benzotriazole (Bt, **10**, Fig. 2 and Supplementary Table 1).

For a systematic optimisation of all reaction parameters, 3,3,3-trifluoropropionic acid (**1b**) was chosen as the test substrate to enable rapid analysis of the complex product mixtures by ^19^F NMR. **1b** is a particularly challenging substrate since its anodic decarboxylation yields primary radicals (or cations) destabilised by an electron-withdrawing CF_3_ group. Under classical Hofer–Moest conditions with hydroxide bases and methanol as nucleophile, the Kolbe dimer **6b** was the major product (42%), and products from C–O bond formation (**5b**^2^ and **5b**^4^) were formed in low yields (Table 1, entry 1). With water as the nucleophile, the yield of C–O bond formation was even lower (entry 2).

**Table 1:** Optimisation of the electrodecarboxylative C–O coupling^a^.


Entry	2a	Base (eq.)	Solvent (ratio)	3ba	5b^2^	5b^4^	6b	9b
1	–	NaOH (1.0)	MeOH	<1	10	<1	42	<1
2^b^	–	”	H_2_O	<1	<1	6	<1	11
3	1.0	LiOH (1.8)	MeOH	17	5	13	3	2
4^b^	”	”	MeCN, DMF, DCM or H_2_O	<1	<1	<1	<1	<1
5	”	”	MeOH/Py (1:1)	25	<1	10	7	5
6	”	”	MeOH/Py (4:1)	36	<1	12	<1	6
7	”	Bu_4_NOH (1.8)	”	18	<1	17	<1	11
8	”	KOH (1.8)	”	13	<1	8	<1	27
9	”	LiOAc (1.8)	”	18	3	3	3	4
10	”	Li_2_CO_3_ (1.8)	”	54	<1	8	<1	9
11	”	Li_2_CO_3_ (0.8)	”	47	<1	7	<1	8
12	”	Li_2_CO_3_ (3.0)	”	48	<1	2	<1	4
13^c^	”	Li_2_CO_3_ (1.8)	”	43	<1	8	<1	8
14^d^	”	”	”	<1	<1	<1	<1	<1
15^e^	”	”	”	40	<1	6	11	6
16	1.5	”	”	**68**	<1	7	<1	9
17	5.0	”	”	23	<1	3	<1	2
18^f^	1.5	”	”	50	<1	5	<1	10
19^g^	”	”	”	59	<1	9	<1	8
20^h^	”	”	”	66	<1	6	<1	9
21^i^	”	”	”	57	<1	8	<1	5
22^j^	”	”	”	<1	<1	**70**	<1	12

Bold font indicates optimal conditions.

^a^Reaction conditions: 1.0 mol of TFPA **1b**, HOBt·H_2_O **2a**, base, Pt(+)/Pt(–) (2.0 cm^2^), undivided cell, 500 mA for 30 min (9.33 F mol^–1^), 12 ml solvent, r.t., 30 min. Yields determined by ^19^F NMR using (trifluoromethoxy) benzene as the internal standard.

^b^>70% of **1b**.

^c^Carbon anode.

^d^Ag anode.

^e^Ni anode.

^f^300 mA.

^g^700 mA.

^h^60 min.

^i^0 °C.

^j^In situ hydrogenolysis over Pd/C.

Fichter-type nitration did not give any alcohol derivatives (Supplementary methods). In the presence of HOBt·H_2_O, encouraging quantities of **3ba** were formed along with small quantities of Hofer–Moest adducts (**5b**^2^ and **5b**^4^), **6b** and trifluoroacetaldehyde **9b** (entry 3). Its formation is in line with reports on electroreductive N–O cleavage reactions yielding aldehydes^65^. Methanol was found to be the ideal solvent for the pursued C–O coupling, while solvents such as MeCN, DMF, DCM or H_2_O were ineffective (entry 4 and Supplementary Table 2). In combination with methanol, a pyridine co-solvent markedly improved the selectivity for **3ba** (entries 5–6). This may be rationalised by its coordination to the anode, separating the radicals.

The choice and amount of base are critical to ensure deprotonation of both HOBt **2a** (p*K*_a_ = 4.6)^66^ and **1b** (p*K*_a_ = 3.0)^67^. Lithium bases gave the best results, in particular a 1.8-fold excess of Li_2_CO_3_ (entries 6–11). Pt was optimal as the anode material (entries 12–14). A slight excess of HOBt was beneficial, whereas a larger excess of HOBt decreased the reaction rate and led to an insoluble coating of the anode as well as to benzotriazole formation (entries 15–16). A reaction time of 30 min at room temperature and a constant current of 500 mA using Pt electrodes (2.0 × 1.0 cm) were found to be optimal (entries 17–20). Structural variations of HOBt had little effect on the reaction outcome (Supplementary Table 3).

The benzotriazole ester can be cleaved in situ by hydrogenating the reaction mixture over Pd/C, furnishing alcohol **5b**^4^ in 70% yield along with minor quantities of **9b** (entry 22).

### Structural scope

The examples in Table 2 demonstrate that the electrodecarboxylative radical C–O coupling is broadly applicable. Many primary and secondary carboxylates, most of which are well outside of the scope of Hofer–Moest protocols, were successfully converted to the corresponding HOBt ethers. Slight adjustments of the conditions (LiOH vs Li_2_CO_3_ as the base, different stoichiometry) were sometimes necessary to obtain optimal yields (Supplementary Table 4). Common functionalities such as halides, olefins, amides, esters, ketones, alcohols, or ethers were tolerated, and even complex structures gave reasonable yields. The reaction was easily scaled up both in batch and flow-conditions with no loss of yield (Supplementary methods). As for many other electrochemical reactions, some yields are only moderate at this prototype stage of development, but the inexpensive starting materials and straightforward protocol make up for this. Both the carboxylic acid and HOBt are usually fully converted, and the products are obtained along with only traces of easy-to-separate byproducts. The yields can be improved by specifically adapting the conditions for a given substrate, e.g. for **3b’a**, NaOH is more effective than LiOH. Starting from readily available difluoroacetic acid, the reaction gives convenient and sustainable access to Ngai difluoromethoxylating reagents (**3f′b**–**3f′d**)^68^, which effectively promote the direct C–H difluoromethoxylation of (hetero)arenes. Tertiary carboxylic acids or those with a strong stabilising effect for the corresponding CCR (e.g. α-amino acids) are generally not compatible.

**Table 2:**
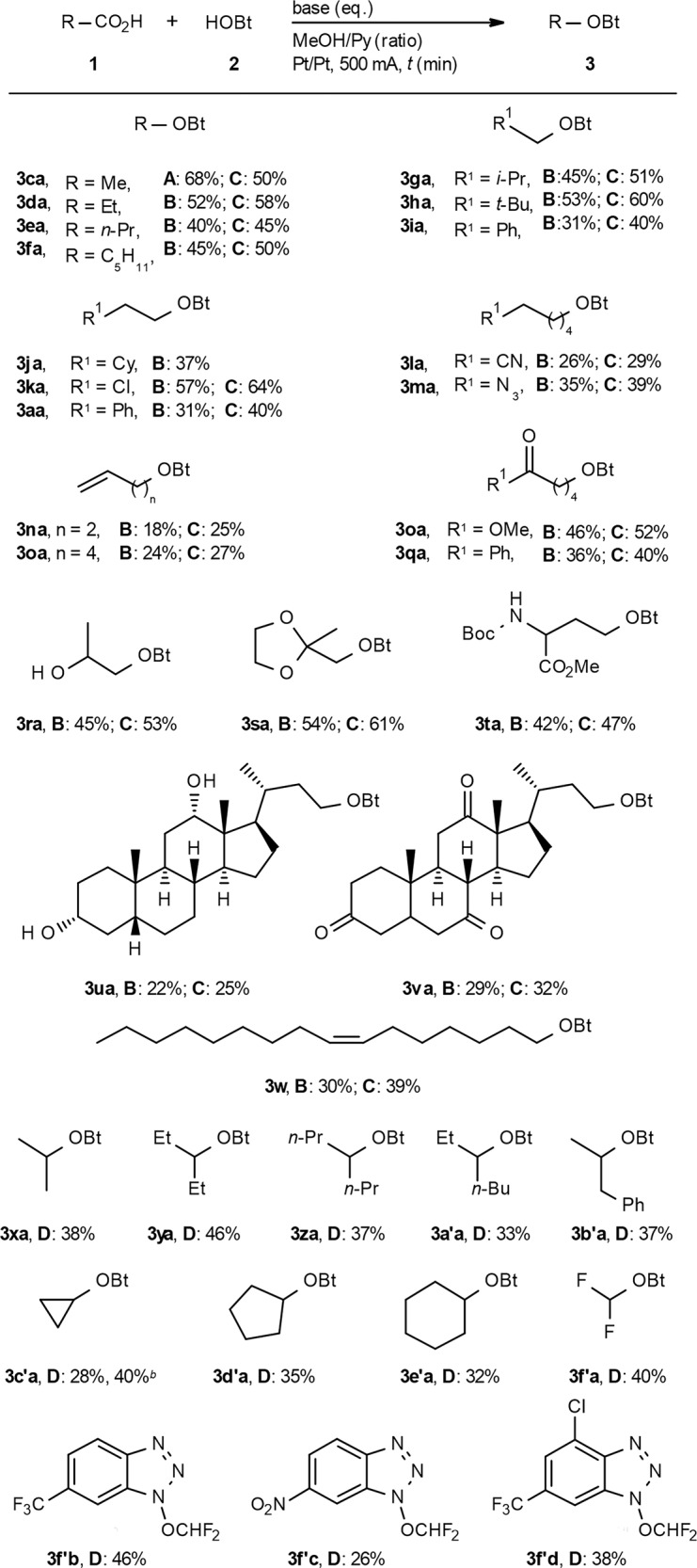
Scope of the electrodecarboxylative radical C–O coupling^a^.

^a^Conditions: Pt/Pt (2 cm^2^), undivided cell, 500 mA for 30 min (9.33 F mol^–1^), 12 ml of MeOH/Py (4:1), r.t. Method A: **1** (1.0 mmol), **2** (1.5 eq.), Li_2_CO_3_ (1.8 eq.); Method B: **1** (0.5 mmol), **2** (1.0 eq.), LiOH (1.8 eq.), 500 mA for 15 min (4.66 F mol^–1^); Method C: **1** (0.5 mmol), **2** (2.5 eq.), LiOH (1.8 eq.); Method D: **2** (0.5 mmol), **1** (2.5 eq.), LiOH (2.5 eq.), MeOH/Py (2:1).

^b^NaOH (5.0 eq.). Isolated yields based on the stoichiometry-limiting substrate.

### Derivatisation

**Fig. 3:**
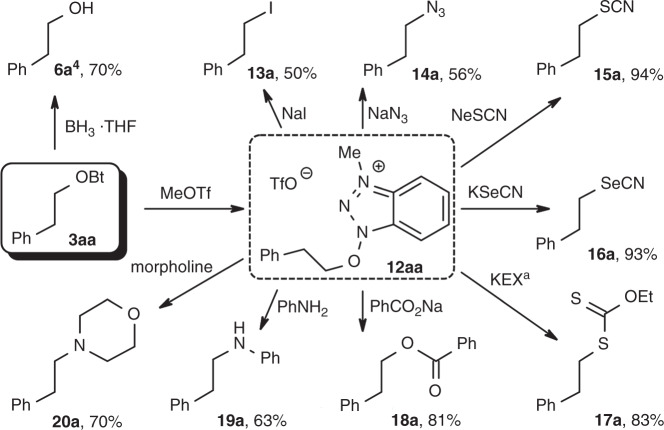
Activation and derivatisation of 3aa. ^a^KEX = potassium ethyl xanthate.

As demonstrated for the hydrocinnamate derivative **3aa**, the benzotriazole moiety can be removed by reductive N–O cleavage to give the corresponding alcohol. By quaternisation with methyl triflate, the BtO-moiety can be turned into a leaving group for nucleophilic substitutions, giving access to esters, ethers, azides, thiocyanates and xanthates (Fig. 3).

### Mechanistic studies

To shed some light on the mechanism of the electrochemical bond-forming step, a series of control experiments were performed (Supplementary methods). Addition of TEMPO slowed down the electrolysis of **1a** considerably, and besides **3aa**, the TEMPO-coupled product **3a-OTEMP** was detected (Supplementary methods).

**Fig. 4:**
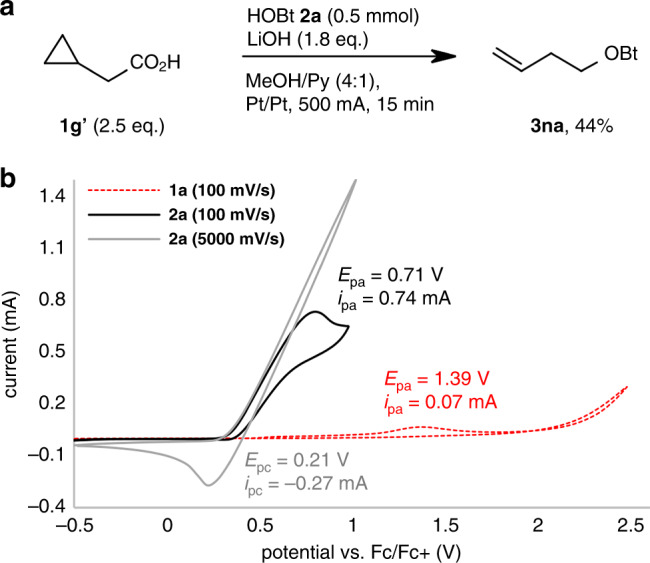
Mechanistic insights. **a** Radical clock experiment. **b** Cyclic voltammetry of hydrocinnamic acid (**1a**) and HOBt (**2a**). Voltammograms recorded in 100 mM solutions of analyte with (*n*-Bu)_4_NPF_6_ or (*n*-Bu)_4_NBF_4_ (100 mM) in MeOH/pyridine (4:1). Starting potential: −0.5 V; Pt disk (2 mm diameter, working electrode); Pt wire (counter electrode); Ag/Ag^+^ redox couple (reference electrode). Potential scale calibrated against external ferrocene/ferrocenium^75^. Red dotted line: hydrocinnamic acid **1a** (100 mV s^−1^); black line: HOBt **2a** (100 mV s^−1^); grey line: HOBt **2a** (5000 mV s^−1^).

Electrolysis of cyclopropylacetic acid **1g′** led to the formation of the homoallyl ether **3na** via ring opening (Fig. 4a). These findings both support a pathway via carbon-centred radical intermediates. Oxidation to carbocations is unlikely since this would inevitably have led to larger quantities of methyl ether products in MeOH as the solvent.

Cyclic voltammetry confirmed that the oxidation of HOBt takes place already at 0.71 V, whereas that of hydrocinnamic acid **1a** requires 1.39 V (vs Fc/Fc^+^ with Ag/Ag^+^ as reference electrode, Fig. 4b). Electrochemically generated acyloxy radicals undergo swift decarboxylation, rendering this oxidation step irreversible^69^.

The voltammogram of HOBt features a small reduction peak at high scan rates (5000 instead of 100 mV/s), which indicates a certain persistence of the BtO^•^ radical^70,71^. This radical has been characterised by UV/vis and EPR spectroscopy, and a half-life of 110 s in MeCN has been reported^72,73^. It is known to decay into a non-reducible compound, presumably benzotriazole^69,74^. Although BtO^•^-decomposition is strongly accelerated by the presence of H-donors^69^, its lifetime is still in the range of hundreds of milliseconds in a mixture of MeOH/pyridine (Supplementary methods). In addition, when using excess HOBt, benzotriazole becomes the main product, and benzotriazole-derived products coat the anode. These data seem to point to the intermediacy of surface-bound BtO^•^ radicals.

## Discussion

In conclusion, this work provides the proof of concept for an electrodecarboxylative radical combination approach to C–O bond formation. The prototype protocol allows the electrochemical conversion of carboxylic acids into alkyl benzotriazole ethers, which were shown to act as versatile synthons or useful fluoroalkoxylation reagents. The reaction scope extents to non-activated primary and secondary carboxylic acids, many of which are well outside the intrinsic boundaries of Hofer–Moest-type reactions. It paves the way for the development of high-yielding and generally applicable electrodecarboxylative C–O or C–N bond formations based on new generations of efficient heteroatom radical sources.

## Methods

### General procedure for the electrodecarboxylative C–O cross-coupling

A 20 ml vessel equipped with two Pt electrodes (2.0 × 1.0 cm) was charged with a solution of the corresponding acid (**1**), 1-hydroxybenzotriazole (**2**) and the base in 12 ml of a mixture of MeOH/Py. The reaction mixture was electrolysed at a current of 500 mA for 15–30 min at room temperature. Then, the volatiles were removed in vacuo and the residue purified by chromatography (*n*-hexane/EtOAc, 4:1), yielding the corresponding C–O coupling product **3**. Note: due to its explosive nature, HOBt was used exclusively in the form of its stable hydrate.

### Synthesis of 1-(2,2,2-trifluoroethoxy)-1*H*-benzo[*d*][1,2,3]triazole (3ba), exemplifies use of Method A

Compound **3ba** was prepared following the general procedure using a solution of 3,3,3-trifluoropropionic acid (**1b**, 91 µl, 1.0 mmol), 1-hydroxybenzotriazole monohydrate (**2a**, 230 mg, 1.50 mmol) and Li_2_CO_3_ (134 mg, 1.80 mmol) in 12 ml of a 4:1-mixture of MeOH/Py. A current of 500 mA was applied for 30 min (9.33 F mol^–1^). **3ba** was isolated as a colourless solid (135 mg, 62%, m.p. 59–61 °C).

### Synthesis of 1-(2-chloroethoxy)-1*H*-benzo[*d*][1,2,3]triazole (3ka), exemplifies use of Method B

Compound **3ka** was prepared following the general procedure using a solution of 3-chloropropionic acid (**1k**, 55 mg, 0.50 mmol), 1-hydroxybenzotriazole monohydrate (**2a**, 78 mg, 0.50 mmol) and LiOH (22 mg, 0.90 mmol) in 12 ml of a 4:1-mixture of MeOH/Py. A current of 500 mA was applied for 15 min at room temperature (9.33 F mol^–1^). **3ka** was isolated as an orange oil (56 mg, 57%).

### Synthesis of 1-ethoxy-1*H*-benzo[*d*][1,2,3]triazole (3da), exemplifies use of Method C

Compound **3da** was prepared following the general procedure using a solution of propionic acid (**1d** 94 μl, 1.3 mmol), 1-hydroxybenzotriazole monohydrate (**2a**, 78 mg, 0.50 mmol) and LiOH (22 mg, 0.90 mmol) in 12 ml of a 4:1-mixture of MeOH/Py. A current of 500 mA was applied for 15 min at room temperature. **3da** was isolated as a brown oil (47 mg, 58%).

### Synthesis of 1-(pentan-3-yloxy)-1*H*-benzo[*d*][1,2,3]triazole (3ya), exemplifies use of Method D

Compound **3ya** was prepared using a solution of 2-ethylbutyric acid (**1y**, 158 μl, 1.25 mmol), 1-hydroxybenzotriazole monohydrate (**2a**, 78 mg, 0.50 mmol) and LiOH (31 mg, 1.3 mmol) in a 2:1-mixture of MeOH/Py. A current of 500 mA was applied for 15 min at room temperature. **3ya** was isolated as an orange oil (47 mg, 46%).

### Procedure for the in situ hydrogenation of 3,3,3-trifluoropropionic acid (1b)

A 20 ml vessel equipped with two Pt electrodes (2.0 × 1.0 cm) was charged with a solution of 3,3,3-trifluoropropionic acid (**1b**, 91 µl, 1.0 mmol), 1-hydroxybenzotriazole monohydrate (**2a**, 230 mg, 1.50 mmol) and Li_2_CO_3_ (134 mg, 1.80 mmol) in a 4:1-mixture of MeOH/Py (12 ml). The reaction mixture was electrolysed at a current of 500 mA for 30 min at room temperature. The crude reaction mixture was diluted with MeOH to 20 ml, to give a 0.034 M solution of **3ba** assuming a conversion similar to that of previous experiments (i.e. 68%). An aliquot of 4.0 ml (0.14 mmol of **3ba**) was introduced into a 10 ml vial together with palladium on carbon (10% Pd) (7.0 mg, 5 mol%). The vial was placed in an autoclave, the mixture was pressurised with hydrogen (30 bar), and stirred at room temperature for 12 h. The crude mixture was analysed by ^19^F NMR using trifluoromethoxybenzene (20 µl, 0.15 mmol, 1.1 eq.) as internal standard, showing the formation of **5b**^4^ and **11b** in 70% and 12% yield, respectively.

## Supplementary information

Supplementary Information

## Data Availability

The authors declare that the data supporting the findings of this study are available within the paper and its Supplementary information files.
