# Supplementary material for: Taking electrodecarboxylative etherification beyond Hofer–Moest using a radical C–O coupling strategy
Source: Nat Commun. 2020 Sep 2;11:4407. doi: 10.1038/s41467-020-18275-1 (PMC7468261; doi:10.1038/s41467-020-18275-1)
Supplement: Supplementary file 1 — Supplementary Information [file 41467_2020_18275_MOESM1_ESM.pdf]

Supporting Information

Taking Electrodecarboxylative Etherification beyond Hofer-Moest using a Radical C–O Coupling Strategy

Ángel Manu Martínez[1], Davit Hayrapetyan[1], Tim van Lingen[1], Marco Dyga[1] and Lukas J. Gooßen[1]*

[1]*Fakultät für Chemie und Biochemie, Ruhr-Universität Bochum*
Universitätsstraße. 150, 44801 Bochum (Germany)

Index

Supplementary Note 1 1
Supplementary Tables 2
Evaluation of *O*-sources under Kolbe conditions 2
Evaluation of reported C–O coupling methods for conflicting substrates 2
Hofer-Moest conditions 2
Fichter conditions[2–12] 3
Optimization studies 6
Standard conditions A 6
Evaluation of the benzotriazole moiety 7
Standard conditions B and C 7
Standard conditions D 8
Unsucessful substrates 8
Supplementary Methods 8
Typical procedure for the synthesis of HOBt-derivatives 8
Synthesis of 4-((*tert*-butoxycarbonyl)amino)-5-methoxy-5-oxopentanoic acid (**3t**) 9
General procedure for electrochemical decarboxylative C(sp^3)–O cross-coupling 10
Primary carboxylic acids 10
Secondary carboxylic acids 17
Synthesis of difluoromethoxylating agents 20
In situ hydrogenation of TPFA (**1b**) 21
Samarium-mediated hydroxylation of **3aa** 22
Scale-up 22
Batch Conditions 22
Continuous Flow Conditions 23
General procedure for the activation of HOBt-derivatives 23
Derivatization of **12aa** *via* nucleophilic substitution 24
Iodination 24
Azidation 24
Thiocyanation 24
Selenocyanation 25
Xanthate esterification 25
Esterification 25
Amination 26
Mechanistic studies 26
Radical scavenger experiment 26
Radical clock experiment 27
Cyclic voltammetric studies 27
NMR Spectra 34
Supplementary References 88

Supplementary Note 1

General remarks: Solvents were purchased (puriss p.A.) from commercial suppliers and purified by standard procedures before use.[1] Commercial substrates were purchased from *Merck®* (*Sigma-Aldrich®*), *Acros®*, *Alfa Aesar®* and *Fluorochem®*, and were used without further purification.

All reactions were monitored by gas chromatography (GC) using *n*-undecane as an internal standard. GC-analyzes were carried out on an *Agilent® HP-5 capillary column* (phenylmethyl siloxane, 30 m × 320 × 0.25, 100/2.3-30-300/3), using the following conditions: Carrier gas: N_2; Initial Temperature: 60 °C; Final Temperature: 300 °C; Rate: 30 $°C \cdot min^{-1}$; Hold Time: 8 min; Column Flow: 1.0 mL min^{-1}; Detector: FID, Temperature FID: 330 °C.

Nuclear magnetic resonance (^{1}H, ^{13}C and ^{19}F NMR) spectra were recorded on *Bruker®* 400, 300 and 250 MHz spectrometers at ambient temperature using chloroform-d, dimethylsulfoxide-d_6 and methanol-d_4 as solvents, with proton, carbon and fluorine resonances at 400/300/250, 101/75/63 and 377/235 MHz, respectively. ^{19}F NMR experiments were measured on a Bruker DPX-250 (fluorine resonance at 235 MHz), and a Spinsolve® spectrometer by Magritek® (fluorine resonance at 41 MHz), using hexafluorobenzene and trifluoromethoxybenzene as standards. All NMR-data are reported in parts per million (ppm) relative to the solvent signal.

Column chromatography was performed on a *Teledyne ISCO CombiFlash® Companion®* or a *Reveleris® X2* (*BÜCHI®*) *Flash Chromatography-System* using *Reveleris®* packed columns (12 g).

Mass spectral data were acquired on a GC-MS *Agilent® 5977B MSD*™. High-resolution mass spectrometry (HRMS) analyzes were obtained on a *Waters® GCT Premier CAB163*™ with a TOF mass analyzer or on a *Thermo Fischer Scientific® LTQ Orbitrap XL*™ with positive ion mode.

Melting points were measured on a *Mettler Toledo® One Click™-MP70*.

Infrared experiments were carried out on a *Bruker® Vertex 70 Spectrometer*™ with *Universal ATR sampling Accessory*.

Electrolytic reactions were performed in oven-dried 20.0 mL glass vessels (Supplementary Figure 1) equipped with a Teflon™-coated stirring bar, under air at room temperature (Supplementary Figure 2). The vessels were sealed using a screw cap carrying two platinum 10.0 mm × 20.0 mm sheet electrodes (Supplementary Figure 3) and placed in an aluminium block as a heat sink. A constant current was supplied by a *Rohde&Schwarz® Programmable 4-Channel High-Performance Power Supply HMP4040*™.

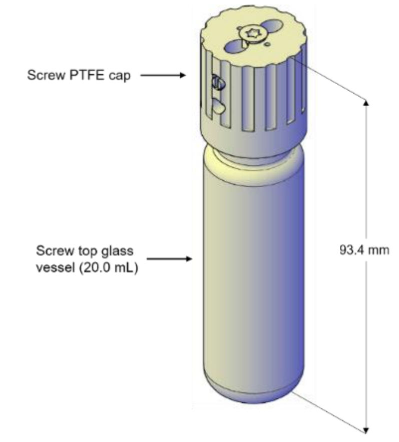

Supplementary Figure 1. Sketch view of the electrolytic cell.

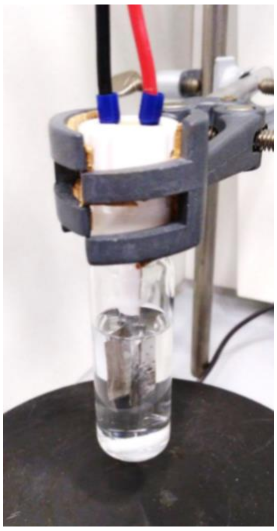

Supplementary Figure 2. Electrolytic cell.

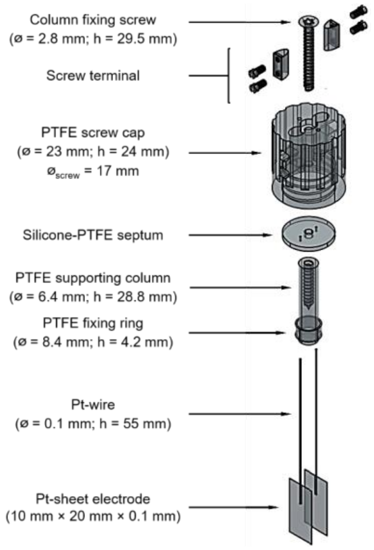

Supplementary Figure 3. Detailed view of the cap.

Supplementary Tables

Evaluation of *O*-sources under Kolbe conditions

Supplementary Table 1. Evaluation of O-sources under Kolbe conditions.[a]

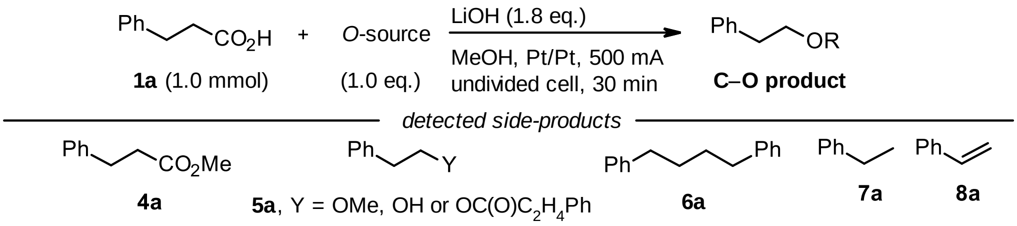

#	*O*-source	C–O product	4a (%)	5a (%)	6a (%)	7a (%)	8a (%)
**1**	none	–	2	10	12	8	18
**2**	H_2O	–	–	7	–	2	3
**3**	$K_2S_2O_8$	–	–	6	1	–	–
**4**	TEMPO[b]	–	–	11	2	–	–
**5**	$HOBt\cdot H_2O$[c]	**6**	2	12	1	2	2
**6**	NHPI[d]	–	3	–	–	–	–
**7**	NHS[e]	–	3	–	–	–	–
**8**	Py-*N*-O[f]	–	3	18	4	4	2
**9**	*t*-BuOOH	–	–	11	2	2	2
**10**	CHP[g]	–	–	3	1	1	1
**11**	DCP[h]	–	–	10	3	3	2
**12**	DTBP[i]	–	–	10	3	3	1
**13**	H_2O_2	–	3	18	4	4	2
**14**	TBPB[j]	–	34	10	2	3	1

[a] *Reaction conditions*: **1a** (76 mg, 0.50 mmol), *O*-source (1.0 eq.), LiOH (1.8 eq.), MeOH (12 mL), 250 mA, 30 min, r.t.; Yields were determined by GC-analysis using *n*-undecane as internal standard. [b] (2,2,6,6-Tetramethylpiperidin-1-yl)oxyl. [c] Hydroxybenzotriazole. [d] *N*-Hydroxyphthalimide. [e] *N*-Hydroxysuccinimide. [f] Pyridine-*N*-oxide. [g] Cumene hydroperoxide. [h] Dicumyl peroxide. [i] Di-*tert*-butyl peroxide. [j] *tert*-Butyl peroxybenzoate

Evaluation of reported C–O coupling methods for conflicting substrates

Hofer-Moest conditions

❖ **Hydrocinnamic acid (1a)**

In methanol: A 20 mL vessel equipped with two Pt-electrodes (2.0 × 1.0 cm), was charged with a solution of hydrocinnamic acid **1a** (152 mg, 1.00 mmol) and NaOH (72 mg, 1.8 mmol, 1.8 eq.) in MeOH (12 mL). The reaction mixture was electrolyzed at a current of 500 mA for 30 min at room temperature. GC and GC-MS analysis of the resulting crude showed that styrene and 1,4-diphenylbutane (Kolbe dimer) were the major products, together with small amounts of non-selective derivatives of Hofer-Moest.

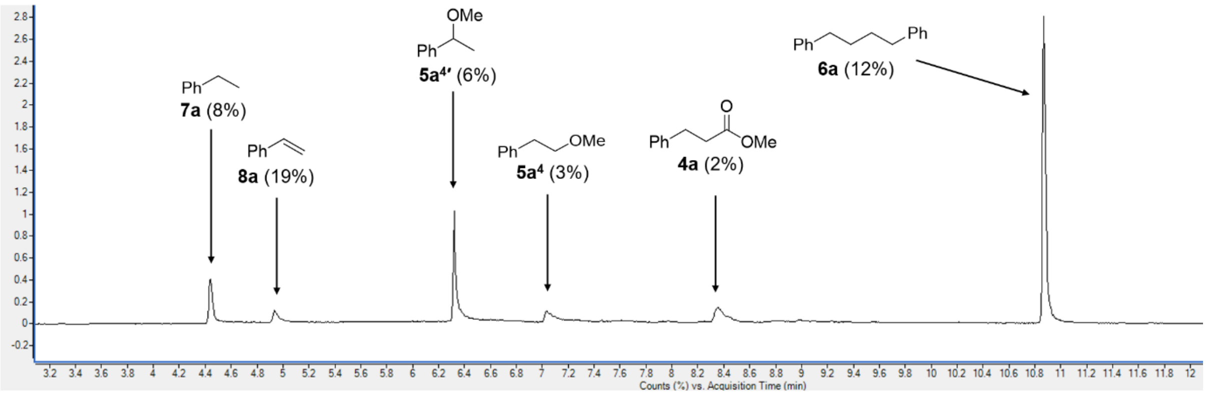

Supplementary Figure 4. GC-MS Spectrum of the Hofer-Moest reaction of **1a** (in MeOH).

In water: A 20 mL vessel equipped with two Pt-electrodes (2.0 × 1.0 cm), was charged with a solution of hydrocinnamic acid **1a** (152 mg, 1.00 mmol) and NaOH (72 mg, 1.8 mmol, 1.8 eq.) in H_2O (12 mL). The reaction mixture was electrolyzed at a current of 500 mA for 30 min at room temperature. ^{19}F NMR-analysis of the resulting crude showed traces of the expected trifluoroethanol (<2%), along with a mixture of products derived from elimination processes such as ethylbenzene (**7a**) and/or styrene (**8a**).

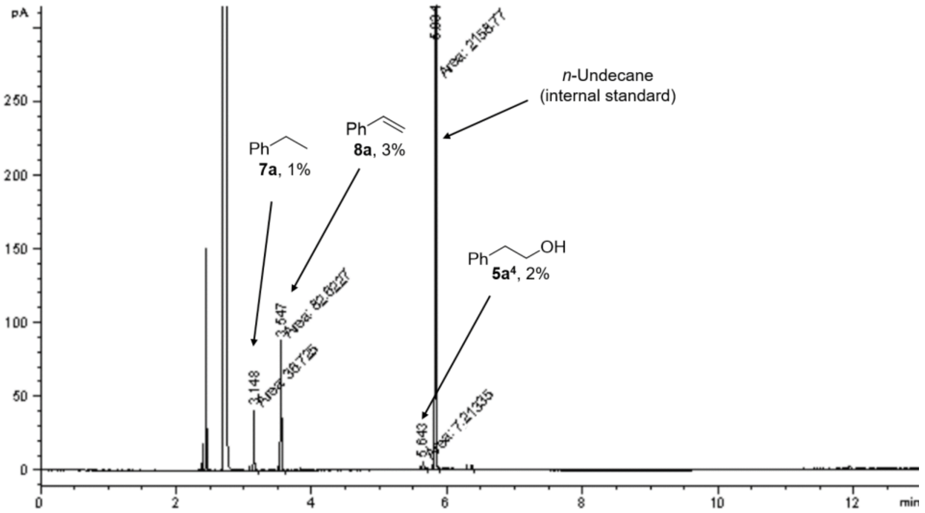

Supplementary Figure 5. GC Spectrum of the Hofer-Moest reaction of **1a** (in H_2O).

❖ **Trifluoroporoionic acid (1b)**

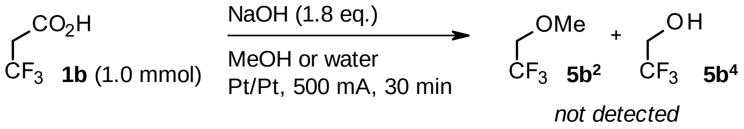

Supplementary Figure 6. Hofer-Moest reactions of **1b** in both MeOH and H_2O.

In methanol: A 20 mL vessel equipped with two Pt-electrodes (2.0 × 1.0 cm), was charged with a solution of TFPA **1b** (91 µL, 1.0 mmol) and NaOH (72 mg, 1.8 mmol, 1.8 eq.) in MeOH (12 mL). The reaction mixture was electrolyzed at a current of 500 mA for 30 min at room temperature. ^{19}F NMR-analysis of the resulting crude showed that 1,1,1,4,4,4-hexafluorobutane (Kolbe dimer) was the major product (42%) along with 10% of methyl ether **5b**[2] and traces of 2,2,2-trifluoroethanol **5b**[4].

In water: A 20 mL vessel equipped with two Pt-electrodes (2.0 × 1.0 cm), was charged with a solution of TFPA **1b** (91 µL, 1.0 mmol) and NaOH (72 mg, 1.8 mmol, 1.8 eq.) in H_2O (12 mL). The reaction mixture was electrolyzed at a current of 500 mA for 30 min at room temperature. ^{19}F NMR-analysis of the resulting crude showed no trace of the expected trifluoroethanol, but a mixture of products derived from elimination or rearrangement processes.

Fichter conditions[2–12]

❖ **Hydrocinnamic acid (1a)**

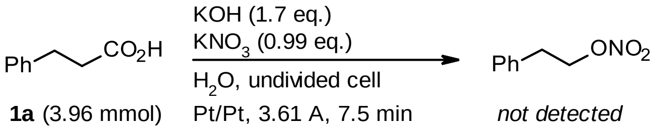

Supplementary Figure 7. Reaction of **1a** under Fichter's conditions.

Following a procedure described by Fichter *et al.*,[2] a 20.0 mL vessel equipped with two Pt-electrodes (2.0 × 1.0 cm), was charged with a solution of hydrocinnamic acid **1a** (601 mg, 3.96 mmol), KOH

(447 mg, 6.88 mmol, 1.71 eq.) and KNO_3 (403 mg, 3.94 mmol, 0.996 eq.) in H_2O (12 mL). A total charge of 27 C was passed through the cell using a current density of 900 mA/cm^2 (3.6 A) for 7.5 min at room temperature. The crude was extracted with Et_2O (2 × 5.0 mL) and *n*-pentane (2 × 5.0 mL), the organic phases were collected, dried over Mg_2SO_4 and analysed by GC and GC-MS. A mixture of Kolbe and Hofer-Moest products was produced, with no trace of the corresponding nitrate ester.

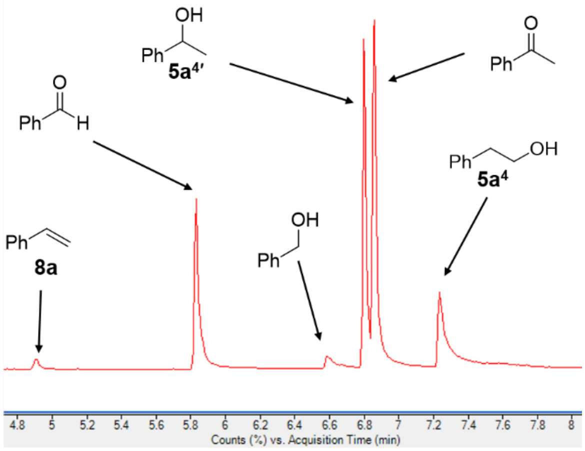

Supplementary Figure 8. GC-MS Spectrum of the electrolysis of **1a** under Fichter's conditions.

❖ Trifluoropropionic acid (1b)

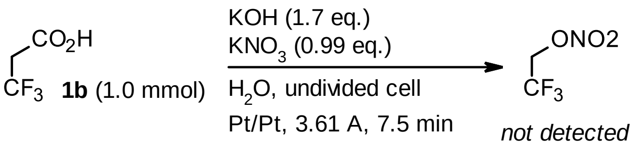

Supplementary Figure 9. Reaction of **1b** under Fichter's conditions.

Following a procedure described by Fichter *et al.*,[2] a 20 mL vessel equipped with two Pt-electrodes (2.0 × 1.0 cm), was charged with a solution of TFPA **1b** (360 µL, 3.96 mmol), KOH (447 mg, 6.88 mmol, 1.71 eq.) and KNO_3 (403 mg, 3.94 mmol, 0.996 eq.) in H_2O (12 mL). A total charge of 27 C was passed through the cell using a current density of 900 mA/cm^2 (3.6 A) for 7.5 min at room temperature. The crude was extracted with Et_2O (2 × 5.0 mL) and *n*-pentane (2 × 5.0 mL), the organic phases were collected, dried over Mg_2SO_4 and analysed by GC and GC-MS. ^{19}F NMR-analysis of the resulting crude showed no trace of the corresponding nitrate ester but a mixture of by-products that were presumably generated by elimination, over-oxidation, or rearrangement processes.

❖ Hexanoic acid (1f)

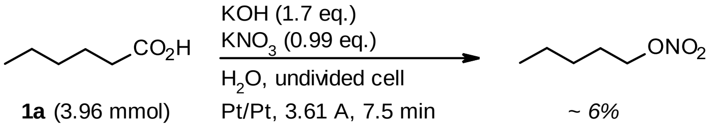

Supplementary Figure 10. Reaction of **1f** under Fichter's conditions.

Following a procedure described by Fichter *et al.*,[2] a 20.0 mL vessel equipped with two Pt-electrodes (2.0 × 1.0 cm), was charged with a solution of hexanoic acid **1f** (501 mg, 3.96 mmol), KOH (447 mg, 6.88 mmol, 1.71 eq.) and KNO_3 (403 mg, 3.94 mmol, 0.996 eq.) in H_2O (12 mL). A total charge of 27 C was passed through the cell using a current density of 900 mA/cm^2 (3.6 A) for 7.5 min at room temperature. The crude was extracted with Et_2O (2 × 5.0 mL) and *n*-pentane (2 × 5.0 mL), the organic phases were collected, dried over Mg_2SO_4 and analysed by GC and GC-MS. As documented by Fichter

et al.,[8] although small amounts of pentyl nitrate were detected (~ 6%), the main products were those corresponding to Kolbe electrolysis in an aqueous medium.

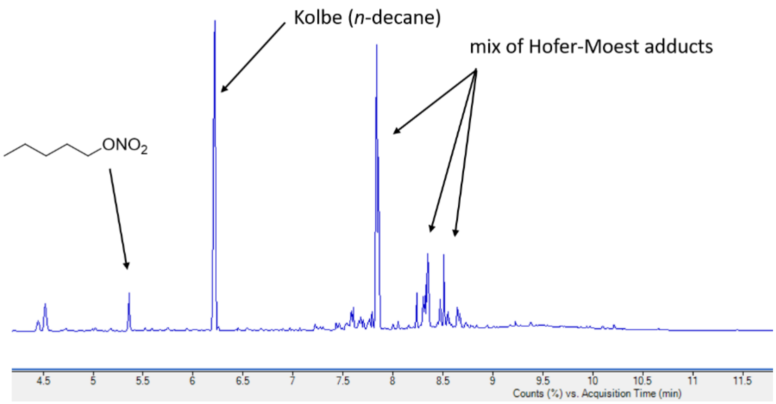

Supplementary Figure 11. GC-MS Spectrum of the electrolysis of **1f** under Fichter's conditions.

Optimization studies

Standard conditions A

Supplementary Table 2. Optimisation of electrodecarboxylative C–O coupling with TPFA **1b**.[a]

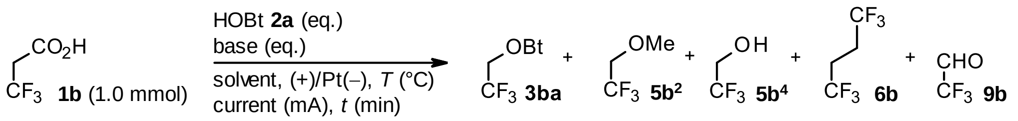

#	2a (eq.)	base (eq.)	solvent (ratio)	A(+)	mA	*t* (min)	*T* (°C)	Yield (%)				
								**3ba**	**5b^2**	**5b^4**	**6b**	**9b**
**1**	–	NaOH (1.0)	MeOH	Pt	500	30	rt	<1	10	<1	42	<1
**2**[b]	–	NaOH (1.0)	H_2O	Pt	500	30	rt	<1	<1	8	<1	11
**3**	–	LiOH (1.8)	MeOH	Pt	500	30	rt	<1	8	<1	44	<1
**4**	1.0	–	MeOH	Pt	500	30	rt	<1	<1	<1	<1	<1
**5**	1.0	LiOH (1.8)	MeOH	–	–	30	70	<1	<1	<1	<1	<1
**6**	1.0	LiOH (1.8)	MeOH	Pt	500	30	rt	17	<1	18	3	2
**7**[b]	1.0	LiOH (1.8)	MeCN	Pt	500	30	rt	<1	<1	<1	<1	<1
**8**[b]	1.0	LiOH (1.8)	DMF	Pt	500	30	rt	<1	<1	<1	<1	<1
**9**[b]	1.0	LiOH (1.8)	DCM	Pt	500	30	rt	<1	<1	<1	<1	<1
**10**[b]	1.0	LiOH (1.8)	H_2O	Pt	500	30	rt	<1	<1	<1	<1	<1
**11**[b]	1.0	LiOH (1.8)	TFE	Pt	500	30	rt	<1	<1	<1	<1	<1
**12**[b]	1.0	LiOH (1.8)	DMSO	Pt	500	30	rt	<1	<1	<1	<1	<1
**13**[b]	1.0	LiOH (1.8)	toluene	Pt	500	30	rt	<1	<1	<1	<1	<1
**14**[b]	1.0	LiOH (1.8)	*i*-PrOH	Pt	500	30	rt	<1	<1	<1	<1	<1
**15**	1.0	LiOH (1.8)	MeOH/Py (1:1)	Pt	500	30	rt	25	<1	20	7	5
**16**	1.0	LiOH (1.8)	MeOH/Py (4:1)	Pt	500	30	rt	36	<1	12	<1	6
**17**	1.0	Bu_4NOH (1.8)	MeOH/Py (4:1)	Pt	500	30	rt	18	<1	17	<1	11
**18**	1.0	KOH (1.8)	MeOH/Py (4:1)	Pt	500	30	rt	13	<1	8	<1	27
**19**	1.0	LiOAc (1.8)	MeOH/Py (4:1)	Pt	500	30	rt	18	3	3	3	4
**20**	1.0	Li_2CO_3 (1.8)	MeOH/Py (4:1)	Pt	500	30	rt	54	<1	8	<1	9
**21**	1.0	LiTFA (1.8)	MeOH/Py (4:1)	Pt	500	30	rt	13	<1	5	4	5
**22**	1.0	$LiClO_4$ (1.8)	MeOH/Py (4:1)	Pt	500	30	rt	<1	<1	4	<1	2
**23**	1.0	$LiBF_4$ (1.8)	MeOH/Py (4:1)	Pt	500	30	rt	2	<1	3	2	2
**24**	1.0	LiO(*t*-Bu) (1.8)	MeOH/Py (4:1)	Pt	500	30	rt	12	<1	2	3	2
**25**	1.0	NaOH (1.8)	MeOH/Py (4:1)	Pt	500	30	rt	5	<1	6	<1	11
**26**	1.0	Li_2CO_3 (0.8)	MeOH/Py (4:1)	Pt	500	30	rt	47	<1	7	<1	8
**27**	1.0	Li_2CO_3 (3.0)	MeOH/Py (4:1)	Pt	500	30	rt	48	<1	2	<1	4
**28**	1.0	Li_2CO_3 (1.8)	MeOH/Py (4:1)	C	500	30	rt	43	<1	8	<1	8
**29**	1.0	Li_2CO_3 (1.8)	MeOH/Py (4:1)	Ag	500	30	rt	<1	<1	<1	<1	<1
**30**	1.0	Li_2CO_3 (1.8)	MeOH/Py (4:1)	Ni	500	30	rt	40	<1	6	11	6
**31**[c]	1.5	Li_2CO_3 (1.8)	MeOH/Py (4:1)	Pt	500	30	rt	**68**	<1	7	<1	9
**32**	5.0	Li_2CO_3 (1.8)	MeOH/Py (4:1)	Pt	500	30	rt	23	<1	3	<1	2
**33**	1.5	Li_2CO_3 (1.8)	MeOH/Py (4:1)	Pt	500	30	rt	50	<1	5	<1	10
**34**	1.5	Li_2CO_3 (1.8)	MeOH/Py (4:1)	Pt	500	30	rt	59	<1	9	<1	8
**35**	1.5	Li_2CO_3 (1.8)	MeOH/Py (4:1)	Pt	500	30	rt	66	<1	6	<1	9
**36**	1.5	Li_2CO_3 (1.8)	MeOH/Py (4:1)	Pt	500	30	rt	57	<1	8	<1	5
**37**	1.5	Li_2CO_3 (1.8)	MeOH/2-Cl-5-CF_3-Py (4:1)	Pt	500	30	rt	34	<1	4	<1	4
**38**	1.5	Li_2CO_3 (1.8)	MeOH/3-Ac-Py (4:1)	Pt	500	30	rt	55	<1	3	<1	2
**39**	1.5	Li_2CO_3 (1.8)	MeOH/3-CO_2Me-Py (4:1)	Pt	500	30	rt	52	<1	3	<1	<1
**40**	1.5	Li_2CO_3 (1.8)	MeOH/4-CO_2Me-Py (4:1)	Pt	500	30	rt	65	<1	3	2	2
**41**	1.5	Li_2CO_3 (1.8)	MeOH/4-Ac-Py (4:1)	Pt	500	30	rt	38	<1	2	0	3
**42**	1.5	Li_2CO_3 (1.8)	MeOH/4-Me-Py (4:1)	Pt	500	30	rt	61	<1	3	0	4
**43**	1.5	Li_2CO_3 (1.8)	MeOH/4-OMe-Py (4:1)	Pt	500	30	rt	44	<1	3	0	3
**44**	1.5	Li_2CO_3 (1.8)	MeOH/DMAP (4:1)	Pt	500	30	rt	<1	<1	<1	<1	<1
**45**	1.5	Li_2CO_3 (1.8)	MeOH/isoquinoline (4:1)	Pt	500	30	rt	34	<1	<1	2	<1
**46**	1.5	Li_2CO_3 (1.8)	MeOH/Py (4:1)	Pt	500	30	0	57	<1	6	<1	11
**47**[d]	1.5	Li_2CO_3 (1.8)	MeOH/Py (4:1)	Pt	500	15	rt	<1	<1	**70**	<1	12

[a] Reaction conditions: **1b** (1.0 mmol), **2a**, base, anode(+)/cathode(–) (2.0 cm^2), undivided cell, current (mA), solvent (12 mL), *T* (°C), *t* (min). Yields determined by ^{19}F NMR using (trifluoromethoxy)benzene as the internal standard. [b] >70% of **1b**.
[c] Reaction conditions “A”. [d] Pd/C-hydrogenolysis *in situ*

Evaluation of the benzotriazole moiety

Supplementary Table 3. Evaluation of substituted hydroxybenzotriazoles

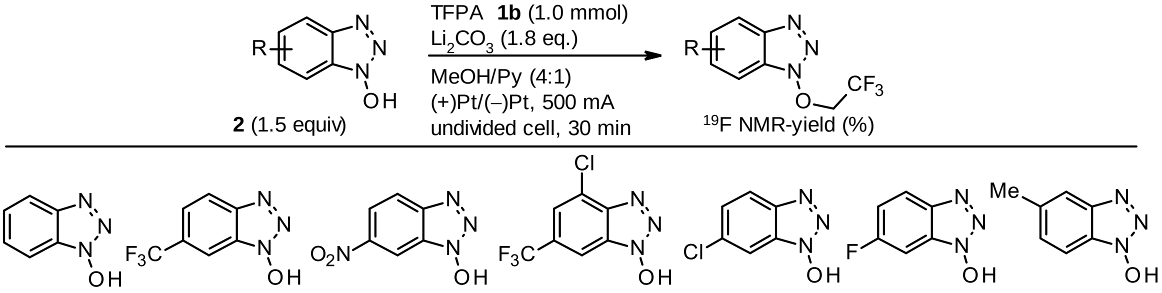

2a, 68% **2b**, 32% **2c**, 28% **2d**, 40% **2e**, 37% **2f**, 44% **2g**, 52%

Standard conditions B and C

Supplementary Table 4. Optimization of the electrodecarboxylative C–O coupling with hydrocinnamic acid **1a**.[a]

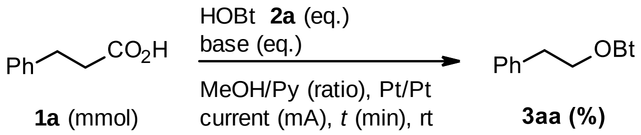

Entry	1a (mmol)	2a (eq.)	base	(eq.)	solvent (ratio)	mA	*t* (min)	3aa (%)
**1**	1.0	1.5	Li_2CO_3	1.8	MeOH/Py (4:1)	500	30	–
**2**	0.5	1.5	LiOH	1.8	MeOH/Py (4:1)	500	30	14
**3**	0.5	1.0	LiOH	1.8	MeOH/Py (4:1)	500	30	26
**4**[b]	0.5	1.0	LiOH	1.8	MeOH/Py (4:1)	500	15	**31**
**5**	0.5	1.0	LiOH	1.8	MeOH/Py (1:1)	500	15	19
**6**	0.5	1.0	LiOH	1.8	MeOH/Py (9:1)	500	15	22
**7**	0.5	1.0	LiOH	1.8	MeOH/Py (4:1)	200	15	22
**8**	0.5	1.0	LiOH	1.8	MeOH/Py (4:1)	700	15	24
**9**	0.5	1.0	LiOH	2.5	MeOH/Py (4:1)	500	15	25
**10**[c]	2.5	1.0	LiOH	1.8	MeOH/Py (4:1)	500	15	**40**

[a] *Reaction conditions*: **1a** (mmol), **2a** (eq.), base (eq.), MeOH/Py (ratio, 12 mL), current (mA), *t* (min), r.t.; Yields were determined by GC-analysis using *n*-undecane as internal standard. [b] Reaction conditions "B". [c] Reaction conditions "C".

Secondary products detected in the Kolbe electrolysis of **1a** and **2a**:

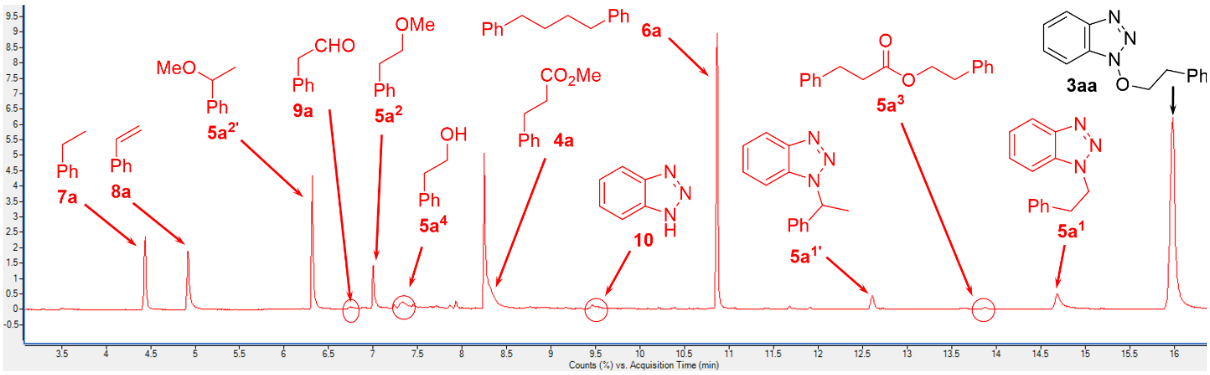

Supplementary Figure 12. GC-MS Spectrum of the standard Kolbe electrolysis of **1a** in the presence of **2a**.

Standard conditions D

Supplementary Table 5. Optimization of the electrodecarboxylative C–O coupling with difluoroacetic acid **1f′**.[a]

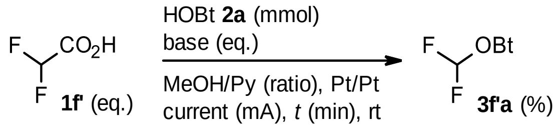

Entry	1f′ (eq.)	LiOH (eq.)	Solvent (ratio)	mA	*t* (min)	3f′a (%)
**1**	1.0	1.8	MeOH	500	15	12
**2**	1.0	2.5	MeOH	500	15	18
**3**	2.5	2.5	MeOH	500	15	23
**4**	2.5	2.5	MeOH	300	25	17
**5**	2.5	2.5	MeOH	700	11	20
**6**	2.5	2.5	MeOH	500	10	26
**7**	2.5	2.5	MeOH	500	25	34
**8**	2.5	2.5	MeOH/Py (1:1)	500	15	29
**9**[b]	2.5	2.5	MeOH/Py (2:1)	500	15	**39**
**10**	2.5	2.5	MeOH/Py (4:1)	500	15	34

[a]*Reaction conditions*: **2a** (0.50 mmol), **1f′** (eq.), base (eq.), MeOH/Py (ratio, 12 mL), current (mA), *t* (min), r.t.; Yields were determined by GC-analysis using *n*-undecane as internal standard. [b] Reaction conditions "D".

Unsucessful substrates

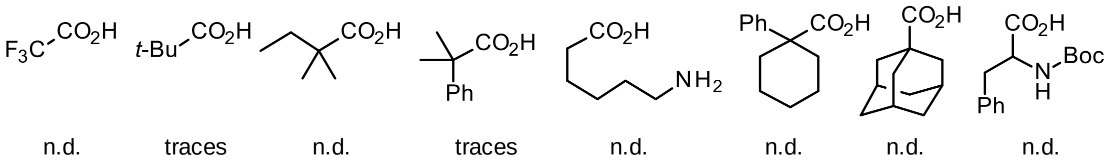

Supplementary Figure 13. Unsuccessful substrates in radical electrodecarboxylative etherification.

Supplementary Methods

Typical procedure for the synthesis of HOBt-derivatives

Synthesis of 6-(Trifluoromethyl)-1*H*-benzo[*d*][1,2,3]triazol-1-ol (2b) [CAS: 26198-21-0]

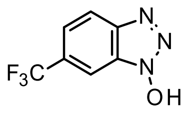

A mixture of the 4-chloro-3-nitrobenzotrifluoride (1.2 mL, 8.0 mmol) and hydrazine hydrate (7.4 mL, 4.0 eq.) was heated at reflux in absolute EtOH (10 mL) for 36 h. After removal of the solvent under reduced pressure, the residue was dissolved in a 10% aqueous solution of Na_2CO_3 and extracted with Et_2O to remove any starting material. Then, HCl (12 M) was added dropwise to precipitate the product, which was filtered, washed with water (2 × 5.0 mL) and dried *in vacuo* yielding **2b** as a pale yellow solid (1.41 g, 87%, mp 147 – 148 °C). **^{1}H NMR (400 MHz, DMSO-d$_6$)** δ = 14.08 (bs, 1H), 8.23 (d, *J* = 8.8 Hz, 1H), 8.18 (s, 1H), 7.69 (dd, *J* = 8.8, 1.2 Hz, 1H) ppm. **^{13}C NMR (101 MHz, DMSO-d$_6$)** δ = 144.0, 127.7 (q, *J* = 32.1 Hz), 127.1, 124.0 (q, *J* = 272.6 Hz), 121.0, 120.7 (q, *J* = 2.9 Hz), 108.4 (q, *J* = 4.6 Hz) ppm. **HRMS (ESI)** *m/z* calcd. for $C_7H_4F_3N_3O$ $[M+H]^+$ 204.0379, found 204.0378. The analytical data (NMR, HRMS analysis) matched those reported in the literature.[13]

6-Nitro-1*H*-benzo[*d*][1,2,3]triazol-1-ol (2c) [CAS: 26185-63-7]

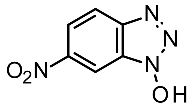

Compound **2c** was prepared following the general procedure from 2-chloro-1,5-dinitrobenzene (1.62 g, 8.00 mmol) yielding **2c** as a pale yellow solid (1.41 g, 52%, mp 190 – 192 °C). **^{1}H NMR (300 MHz, MeOD-d$_4$)** δ = 8.56 (dd, *J* = 2.1, 0.7 Hz, 1H), 8.18 (dd, *J* = 9.2, 2.1 Hz, 1H), 8.01 (dd, *J* = 9.2, 0.7 Hz, 1H) ppm. **^{13}C NMR (75 MHz, MeOD-d$_4$)** δ = 148.1, 145.9, 128.7, 121.0, 120.8, 108.4, 49.0 ppm. **HRMS (ESI)** *m/z* calcd. for $C_6H_4N_4O_3$ $[M+H]^+$ 181.0356, found 181.0357. The analytical data (NMR, HRMS analysis) matched those reported in the literature.[14]

4-Chloro-6-(trifluoromethyl)-1*H*-benzo[*d*][1,2,3]triazol-1-ol (2d) [CAS: 2287307-42-8]

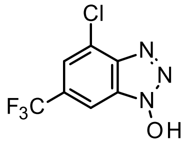

Compound **2d** was prepared following the general procedure from 1,2-dichloro-3-nitro-5-(trifluoromethyl)benzene (2.08 g, 8.00 mmol) yielding **2d** as a colorless solid (1.09 g, 57%, mp 143 °C with decomposition). **^{1}H NMR (300 MHz, MeOD-d$_4$)** δ = 7.77 (d, *J* = 1.2 Hz, 1H), 7.43 (d, *J* = 1.1 Hz, 1H) ppm. **^{13}C NMR (75 MHz, MeOD-d$_4$)** δ = 142.6, 129.6, 129.2 (q, *J* = 33.3 Hz), 126.3, 124.8 (q, *J* = 271.9 Hz), 121.3 (q, *J* = 3.1 Hz), 109.1 (q, *J* = 4.8 Hz) ppm. **^{19}F NMR (235 MHz, MeOD-d$_4$)** δ = −61.17 ppm. **HRMS (ESI)** *m/z* calcd. for $C_7H_4ClF_3N_3O$ $[M+H]^+$ 237.9990, found 237.9993. The analytical data (NMR, HRMS analysis) matched those reported in the literature.

Synthesis of 4-((*tert*-butoxycarbonyl)amino)-5-methoxy-5-oxopentanoic acid (3t)

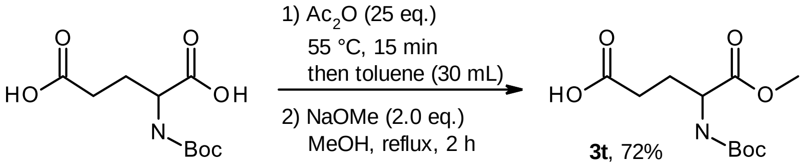

Supplementary Figure 14. Synthesis of **3t** from *N*-Boc glutamic acid.

An oven-dried 50.0 mL vessel was charged with commercially available *N*-Boc glutamic acid (1.20 g, 5.00 mmol) and Ac_2O (5.0 mL, 10 eq.). The resulting mixture was stirred at 55 °C for 15 min. Then, toluene (30.0 mL) was added, and the solution was concentrated to dryness. Traces of AcOH were removed by drying *in vacuo* over KOH. Then, the residue was dissolved in MeOH (15 mL), following by the addition of NaOMe (413 mg, 2.00 eq.). The mixture was stirred at reflux for 2 h. After dilution with water, the solution was acidified with HCl (12 M) to pH = 1, and extracted with EtOAc (2 × 5.0 mL). The combined organic layers were dried over $MgSO_4$ and concentrated by evaporation to give **3t** as a colorless oil (939 mg, 72%). The analytical data (^{1}H NMR and ^{13}C NMR) matched those reported in the literature.[15–17]

General procedure for electrochemical decarboxylative C(sp^3)–O cross-coupling

Primary carboxylic acids

Synthesis of 1-(2,2,2-trifluoroethoxy)-1*H*-benzo[*d*][1,2,3]triazole (3ba) [CAS: 1855674-60-0]

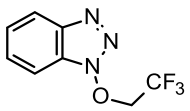

General procedure (*Conditions A*): A 20 mL vessel equipped with two Pt-electrodes (2.0 × 1.0 cm), was charged with a solution of TFPA **1b** (91 µL, 1.0 mmol), 1-hydroxybenzotriazole monohydrate **2a** (230 mg, 1.50 mmol, 1.50 eq.) and Li_2CO_3 (133 mg, 1.80 mmol, 1.80 eq.) in a 4:1-mixture of MeOH/Py (12 mL). The reaction mixture was electrolyzed at a current of 500 mA for 30 min (cumulated charge: 9.33 F·mol^{-1}) at room temperature. The volatiles were removed *in vacuo* and the residue purified by chromatography (*n*-hexane/EtOAc, 4:1) yielding **3ba** as a colorless solid (135 mg, 62%, m.p. 59 – 61 °C). **^{1}H NMR (300 MHz, CDCl$_3$)** δ = 8.03 (d, *J* = 8.4 Hz, 1H), 7.65 – 7.51 (m, 2H), 7.47 – 7.38 (m, 1H), 4.97 (q, *J* = 8.0 Hz, 2H) ppm. **^{13}C NMR (101 MHz, CDCl$_3$)** δ = 143.4, 128.9, 127.4, 125.2, 122.3 (q, *J* = 279.2 Hz), 120.4, 108.7, 75.0 (q, *J* = 35.7 Hz) ppm. **IR (ATR)** $\tilde{v}$ = 2972, 1271, 1166, 1087, 1019, 962, 742 cm^{-1}. **MS (EI)** *m/z* (%) = 217.3 (4) [M]$^+$, 206.9 (4), 189.1 (69), 106.1 (26), 90.1 (100). **HRMS (ESI)** *m/z* calcd. for $C_8H_6F_3N_3O$ [M+H]$^+$ 218.0536, found 218.0535.

Note: due to its explosive nature, HOBt was used exclusively in the form of its stable hydrate.

1-Phenethoxy-1*H*-benzo[*d*][1,2,3]triazole (3aa) [CAS: 1637647-71-2]

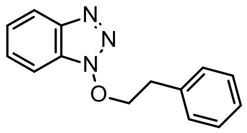

Compound **3aa** was prepared following the general procedure (*conditions B*) from hydrocinnamic acid **1a** (77 mg, 0.50 mmol) yielding **3aa** as an orange oil (37 mg, 31%); *Conditions C*: **1a** (188 mg, 1.25 mmol) to give **3aa** (48 mg, 40%). **^{1}H NMR (300 MHz, CDCl$_3$)** δ = 7.97 (d, *J* = 8.3 Hz, 1H), 7.41 (ddd, *J* = 8.1, 6.9, 1.1 Hz, 1H), 7.37 – 7.23 (m, 7H), 4.73 (t, *J* = 6.9 Hz, 2H), 3.16 (t, *J* = 6.9 Hz, 2H) ppm. **^{13}C NMR (63 MHz, CDCl$_3$)** δ = 143.4, 136.6, 129.0, 128.7, 127.9, 127.3, 127.0, 124.6, 120.2, 108.7, 81.0, 34.6 ppm. **HRMS (ESI)** *m/z* calcd. for $C_{14}H_{13}N_3O$ [M+H]$^+$ 240.1131, found 240.1133. The analytical data (NMR, HRMS analysis) matched those reported in the literature.[18]

1-Methoxy-1*H*-benzo[*d*][1,2,3]triazole (3ca) [CAS: 22713-34-4]

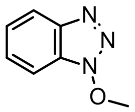

Compound **3ca** was prepared following the general procedure (*conditions A*) from acetic acid **1c** (57 µL, 1.0 mmol) yielding **3ca** as a yellow oil (101 mg, 68%); *Conditions C*: **1a** (71 µL, 1.3 mmol) to give **3aa** (75 mg, 50%). **^{1}H NMR (300 MHz, CDCl$_3$)** δ = 8.01 (d, *J* = 8.4 Hz, 1H), 7.62 – 7.48 (m, 2H), 7.42 – 7.35 (m, 1H), 4.37 (s, 3H) ppm. **^{13}C NMR (75 MHz, CDCl$_3$)** δ = 143.7, 128.1, 126.8, 124.7, 120.4, 108.6, 67.7 ppm. The analytical data (^{1}H NMR and ^{13}C NMR) matched those reported in the literature.[18]

Synthesis of 1-ethoxy-1*H*-benzo[*d*][1,2,3]triazole (3da) [CAS: 57223-16-2]

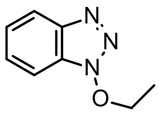

Compound **3da** was prepared following the general procedure (*conditions B*) from propionic acid **1d** (38 μL, 0.50 mmol) yielding **3da** as a brown oil (42 mg, 52%); *Conditions C*: **1d** (94 μL, 1.3 mmol) to give **3da** (47 mg, 58%). **^{1}H NMR (250 MHz, $CDCl_3$)** δ = 8.0 (dd, *J* = 8.4, 0.8 Hz, 1H), 7.60 – 7.46 (m, 2H), 7.42 – 7.32 (m, 1H), 4.62 (q, *J* = 7.1 Hz, 2H), 1.48 (t, *J* = 7.1 Hz, 3H) ppm. **^{13}C NMR (63 MHz, $CDCl_3$)** δ = 143.6, 128.0, 127.7, 124.6, 120.3, 108.8, 76.7, 13.8 ppm. **HRMS (ESI)** *m/z* calcd. for $C_8H_9N_3O$ $[M+H]^+$ 164.0818, found 164.0819. The analytical data (NMR, HRMS analysis) matched those reported in the literature.[18]

1-Propoxy-1*H*-benzo[*d*][1,2,3]triazole (3ea) [CAS: 60454-98-0]

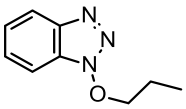

Compound **3ea** was prepared following the general procedure (*conditions B*) from butyric acid **1e** (46 μL, 0.50 mmol) yielding **3ea** as a brown oil (46 mg, 52%); *Conditions C*: **1e** (116 μL, 1.25 mmol) to give **3ea** (51 mg, 58%). **^{1}H NMR (300 MHz, $CDCl_3$)** δ = 7.99 (d, *J* = 8.4 Hz, 1H), 7.60 – 7.44 (m, 2H), 7.36 (ddd, *J* = 8.1, 6.8, 1.2 Hz, 1H), 4.49 (t, *J* = 6.6 Hz, 2H), 1.96 – 1.78 (m, 2H), 1.11 (t, *J* = 7.4 Hz, 3H) ppm. **^{13}C NMR (75 MHz, $CDCl_3$)** δ = 143.5, 127.9, 127.5, 124.6, 120.3, 108.7, 82.4, 21.5, 10.2 ppm. The analytical data (NMR, HRMS analysis) matched those reported in the literature.[19]

1-(Pentyloxy)-1*H*-benzo[*d*][1,2,3]triazole (3fa) [CAS: 60455-00-7]

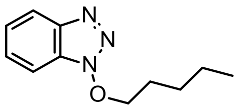

Compound **3fa**was prepared following the general procedure (*conditions B*) from hexanoic acid **1f** (63 μL, 0.50 mmol) yielding **3fa** as a brown oil (41 mg, 40%). *Conditions C*: acid **1f** (156 μL, 1.25 mmol) to give **3fa** (46 mg, 45%); **^{1}H NMR (300 MHz, $CDCl_3$)** δ = 7.99 (d, *J* = 8.4 Hz, 1H), 7.58 – 7.46 (m, 2H), 7.36 (ddd, *J* = 8.1, 6.7, 1.2 Hz, 1H), 4.53 (t, *J* = 6.7 Hz, 2H), 1.85 (dq, *J* = 13.5, 6.8 Hz, 2H), 1.60 – 1.31 (m, 4H), 0.93 (t, *J* = 7.2 Hz, 3H) ppm. **^{13}C NMR (75 MHz, $CDCl_3$)** δ = 143.5, 127.9, 127.5, 124.6, 120.3, 108.7, 81.1, 27.8, 27.8, 22.4, 14.0 ppm. The analytical data (NMR, HRMS analysis) matched those reported in the literature.[19]

1-Isobutoxy-1*H*-benzo[*d*][1,2,3]triazole (3ga) [CAS: 1882571-07-4]

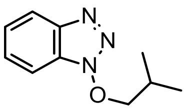

Compound **3ga** was prepared following the general procedure (*conditions B*) from isovaleric acid **1g** (55 μL, 0.50 mmol) yielding **3ga** as an orange oil (43 mg, 45%); *Conditions C*: **1g** (137 μL, 1.25 mmol) to give **3ga** (49 mg, 51%). **^{1}H NMR (300 MHz, $CDCl_3$)** δ = 8.0 (d, *J* = 8.4 Hz, 1H), 7.60 – 7.46 (m, 2H), 7.41 – 7.33 (m, 1H), 4.31 (d, *J* = 6.7 Hz, 2H), 2.29 – 2.11 (m, 1H), 1.13 (d, *J* = 6.7 Hz, 3H) ppm. **^{13}C NMR (75 MHz, $CDCl_3$)** δ = 143.6, 127.9, 127.3, 124.6, 120.3, 108.7, 87.0, 30.9, 27.5, 19.0 ppm. **IR (ATR)** $\tilde{\nu}$ = 2964, 2876, 1470, 1446, 1384, 1367, 1264, 1240, 1089, 976, 782, 767, 742 cm^{-1}. **MS (EI)**

m/z (%) = 191.1 (20) $[M]^+$, 163.1 (62), 107.1 (50), 91.1 (47), 79.1 (51), 77.1 (99), 57.1 (100). **HRMS (ESI)** *m/z* calcd. for $C_{10}H_{13}N_3O$ $[M+H]^+$ 192.1131, found 192.1132.

1-(Neopentyloxy)-1*H*-benzo[*d*][1,2,3]triazole (3ha) [CAS: 2160874-31-5]

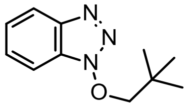

Compound **3ha** was prepared following the general procedure (*conditions B*) from *tert*-butylacetic acid **1h** (64 µL, 0.50 mmol) yielding **3ha** as an orange oil (54 mg, 53%); *Conditions C*: **1h** (160 µL, 1.25 mmol) to give **3ha** (62 mg, 60%). **^{1}H NMR (300 MHz, $CDCl_3$)** δ = 7.98 (d, *J* = 8.4 Hz, 1H), 7.56 – 7.45 (m, 2H), 7.39 – 7.32 (m, 1H), 4.19 (s, 2H), 1.16 (s, 9H) ppm. **^{13}C NMR (75 MHz, $CDCl_3$)** δ = 143.6, 127.9, 127.1, 124.6, 120.3, 108.6, 90.4, 32.1, 26.9, 26.5 ppm. **IR (ATR)** $\tilde{\nu}$ = 2961, 2875, 1476, 1446, 1388, 1367, 1264, 1240, 1090, 963, 742 cm^{-1}. **MS (EI)** *m/z* (%) = 205.1 (17) $[M]^+$, 77.1 (45), 107.1 (73), 91.1 (23), 77.1 (59), 71.1 (100). **HRMS (ESI)** *m/z* calcd. for $C_{11}H_{15}N_3O$ $[M+H]^+$ 206.1288, found 206.1288.

1-(Benzyloxy)-1*H*-benzo[*d*][1,2,3]triazole (3ia) [CAS: 68930-15-4]

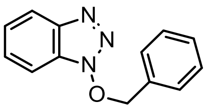

Compound **3ia** was prepared following the general procedure (*conditions B*) from phenylacetic acid **1i** (62 µL, 0.50 mmol) yielding **3ia** as an orange oil (35 mg, 31%); *Conditions C*: **1i** (156 µL, 1.25 mmol) to give **3ia** (45 mg, 40%). **^{1}H NMR (300 MHz, $CDCl_3$)** δ = 7.99 – 7.95 (m, 1H), 7.41 – 7.27 (m, 7H), 7.24 – 7.19 (m, 1H), 5.54 (s, 2H) ppm. **^{13}C NMR (75 MHz, $CDCl_3$)** δ = 143.3, 133.2, 130.0, 129.8, 128.9, 128.0, 127.8, 124.5, 120.0, 108.8, 82.7 ppm. The analytical data (NMR, HRMS analysis) matched those reported in the literature.[18]

1-(2-Cyclohexylethoxy)-1*H*-benzo[*d*][1,2,3]triazole (3ja)

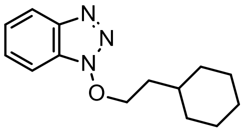

Compound **3ja** was prepared following the general procedure (*conditions B*) from 3-cyclohexanepropionic acid **1j** (86 µL, 0.50 mmol) yielding **3ja** as a dark orange oil (45 mg, 37%). **^{1}H NMR (300 MHz, $CDCl_3$)** δ = 8.01 (d, *J* = 8.4 Hz, 1H), 7.59 – 7.45 (m, 2H), 7.42 – 7.33 (m, 1H), 4.59 (t, *J* = 6.8 Hz, 2H), 1.84 – 1.50 (m, 8H), 1.36 – 1.12 (m, 3H), 1.06 – 0.90 (m, 2H) ppm. **^{13}C NMR (75 MHz, $CDCl_3$)** δ = 143.6, 128.0, 127.5, 124.6, 120.3, 108.7, 79.4, 35.4, 34.4, 33.3, 26.4, 26.2 ppm. **IR (ATR)** $\tilde{\nu}$ = 2921, 2850, 1264, 1239, 969, 953, 741 cm^{-1}. **MS (EI)** *m/z* (%) = 245.2 (6) $[M]^+$, 109.1 (90), 93.1 (36), 91. 1 (51), 81.1 (42), 69.1 (100), 67.1 (93), 55.1 (100). **HRMS (ESI)** *m/z* calcd. for $C_{14}H_{19}N_3O_2$ $[M+H]^+$ 246.1601, found 246.1600.

1-(2-Chloroethoxy)-1*H*-benzo[*d*][1,2,3]triazole (3ka) [CAS: 213249-23-1]

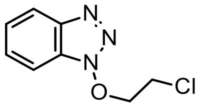

Compound **3ka** was prepared following the general procedure (*conditions B*) from 3-chloropropionic acid **1k** (54 mg, 0.50 mmol) yielding **3ka** as an orange oil (56 mg, 57%); *Conditions C*: **1k** (136 mg,

1.25 mmol) to give **3ka** (63 mg, 64%). **^{1}H NMR (300 MHz, CDCl$_3$)** δ = 7.98 (dt, *J* = 8.4, 0.9 Hz, 1H), 7.66 (dt, *J* = 8.3, 0.9 Hz, 1H), 7.54 – 7.47 (m, 1H), 7.41 – 7.33 (m, 1H), 4.81 – 4.76 (m, 2H), 3.86 – 3.79 (m, 2H) ppm. **^{13}C NMR (75 MHz, CDCl$_3$)** δ = 143.4, 128.4, 127.5, 124.9, 120.2, 108.8, 79.6, 40.2 ppm. **IR (ATR)** $\tilde{\nu}$ = 3068, 2961, 1445, 1430, 1362, 1265, 1239, 1090, 992, 949, 781, 767, 742, 671 cm^{-1}. **MS (EI)** *m/z* (%) = 197.1 (12) [M]$^+$, 169.1 (76), 106.1 (45), 91.1 (47), 90.1 (100). **HRMS (ESI)** *m/z* calcd. for $C_8H_8ClN_3O$ [M+H]$^+$ 198.0429, found 198.0429.

6-((1*H*-Benzo[*d*][1,2,3]triazol-1-yl)oxy)hexanenitrile (3la)

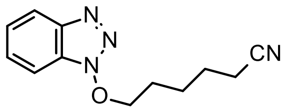

Compound **3la** was prepared following the general procedure (*conditions B*) from 6-cyanohexanoic acid **1l** (71 mg, 0.50 mmol) yielding **3la** as a yellow oil (30 mg, 26%); *Conditions C*: **1l** (176 mg, 1.25 mmol) to give **3la** (46 mg, 40%). **^{1}H NMR (300 MHz, CDCl$_3$)** δ = 8.01 (dt, *J* = 8.4, 0.9 Hz, 1H), 7.59 – 7.48 (m, 2H), 7.42 – 7.35 (m, 1H), 4.56 (t, *J* = 6.3 Hz, 2H), 2.47 – 2.35 (m, 2H), 1.97 – 1.84 (m, 2H), 1.95 – 1.87 (m, 2H), 1.82 – 1.71 (m, 4H) ppm. **^{13}C NMR (75 MHz, CDCl$_3$)** δ = 143.5, 128.2, 127.3, 124.8, 120.3, 119.5, 108.6, 80.2, 27.4, 25.1, 25.0, 17.2 ppm. **IR (ATR)** $\tilde{\nu}$ = 2948, 2870, 2245, 1730, 1446, 1365, 1264, 1240, 1091, 747 cm^{-1}. **HRMS (ESI)** *m/z* calcd. for $C_{12}H_{14}N_4O$ [M+H]$^+$ 231.1240, found 231.1240.

1-((5-Azidopentyl)oxy)-1*H*-benzo[*d*][1,2,3]triazole (3ma)

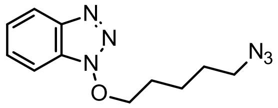

Compound **3ma** was prepared following the general procedure (*conditions B*) from 6-azidohexanoic acid **1m** (79 mg, 0.50 mmol) yielding **3ma** as a yellow oil (43 mg, 35%); *Conditions C*: **1m** (196 mg, 1.25 mmol) to give **3ma** (48 mg, 39%). **^{1}H NMR (300 MHz, CDCl$_3$)** δ = 8.01 (dt, *J* = 8.4, 0.9 Hz, 1H), 7.59 – 7.48 (m, 2H), 7.42 – 7.34 (m, 1H), 4.55 (t, *J* = 6.4 Hz, 2H), 3.38 – 3.29 (m, 2H), 1.95 – 1.83 (m, 2H), 1.73 – 1.64 (m, 4H) ppm. **^{13}C NMR (75 MHz, CDCl$_3$)** δ = 143.6, 128.1, 127.4, 124.7, 120.4, 108.6, 80.6, 51.2, 28.6, 27.7, 23.1 ppm. **IR (ATR)** $\tilde{\nu}$ = 2943, 2868, 2093, 1446, 1359, 1263, 1240, 1089, 743 cm^{-1}. **MS (EI)** *m/z* (%) = 246.2 (4) [M]$^+$, 132.1 (45), 131.1 (59), 119. 1 (100). **HRMS (ESI)** *m/z* calcd. for $C_{11}H_{14}N_6O$ [M+H]$^+$ 247.1302, found 247.1299.

1-(But-3-en-1-yloxy)-1*H*-benzo[*d*][1,2,3]triazole (3na)

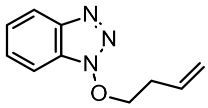

Compound **3na** was prepared following the general procedure (*conditions B*) from 4-pentenoic acid **1n** (51 µL, 0.50 mmol) yielding **3na** as an orange oil (17 mg, 18%); *Conditions C*: **1n** (128 µL, 1.25 mmol) to give **3na** (24 mg, 25%). **^{1}H NMR (300 MHz, CDCl$_3$)** δ = 8.0 (d, *J* = 8.4 Hz, 1H), 7.59 – 7.46 (m, 2H), 7.40 – 7.34 (m, 1H), 5.90 (ddt, *J* = 17.0, 10.2, 6.6 Hz, 1H), 5.27 – 5.13 (m, 2H), 4.60 (t, *J* = 6.7 Hz, 2H), 2.60 (qt, *J* = 6.7, 1.4 Hz, 2H) ppm. **^{13}C NMR (75 MHz, CDCl$_3$)** δ = 143.5, 132.7, 128.0, 127.5, 124.7, 120.3, 118.4, 108.8, 79.7, 32.4 ppm. **HRMS (ESI)** *m/z* calcd. for $C_{10}H_{11}N_3O$ [M+H]$^+$ 190.0375, found 190.0376.

1-(Hex-5-en-1-yloxy)-1*H*-benzo[*d*][1,2,3]triazole (3oa)

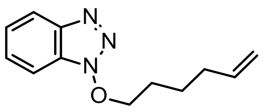

Compound **3oa** was prepared following the general procedure (*conditions B*) from 6-heptenoic acid **1p** (68 µL, 0.50 mmol) yielding **3oa** as an orange oil (26 mg, 24%); *Conditions C*: **1p** (169 µL, 1.25 mmol) to give **3oa** (29 mg, 27%). **^{1}H NMR (300 MHz, $CDCl_3$)** δ = 8.01 (d, *J* = 8.4 Hz, 1H), 7.60 – 7.47 (m, 2H), 7.43 – 7.34 (m, 1H), 5.82 (ddt, *J* = 16.9, 10.2, 6.7 Hz, 1H), 5.09 – 4.94 (m, 2H), 4.56 (t, *J* = 6.6 Hz, 2H), 2.16 (dd, *J* = 14.2, 7.2 Hz, 2H), 1.88 (dq, *J* = 8.4, 6.9 Hz, 2H), 1.73 – 1.61 (m, 2H) ppm. **^{13}C NMR (75 MHz, $CDCl_3$)** δ = 143.6, 138.0, 128.0, 127.5, 124.7, 120.4, 115.3, 108.7, 80.9, 33.3, 27.6, 25.0 ppm. **IR (ATR)** $\tilde{\nu}$ = 2938, 1738, 1641, 1445, 1363, 1204, 1240, 1090, 912, 766, 745 cm^{-1}. **MS (EI)** *m/z* (%) = 217.2 (15) $[M]^+$, 134.1 (58), 130.1 (94), 118.1 (39), 117.1 (33), 91.1 (51), 77.1 (37), 55.1 (100). **HRMS (ESI)** *m/z* calcd. for $C_{12}H_{15}N_3O$ $[M+H]^+$ 218.1288, found 218.1288.

1-Propoxy-1*H*-benzo[*d*][1,2,3]triazole (3pa)

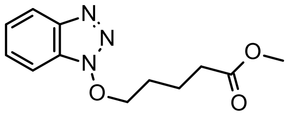

Compound **3pa** was prepared following the general procedure (*conditions B*) from mono-methyl adipate **1p** (74 µL, 0.50 mmol) yielding **3pa** as a pale yellow solid (57 mg, 46%, mp 49 – 50 °C); *Conditions C*: **1p** (185 µL, 1.25 mmol) to give **3pa** (65 mg, 52%). **^{1}H NMR (300 MHz, $CDCl_3$)** δ = 8.0 (dt, *J* = 8.4, 0.9 Hz, 1H), 7.59 – 7.46 (m, 2H), 7.41 – 7.33 (m, 1H), 4.59 – 4.50 (m, 2H), 3.67 (s, 3H), 2.47 – 2.38 (m, 2H), 1.95 – 1.87 (m, 4H) ppm. **^{13}C NMR (101 MHz, $CDCl_3$)** δ = 173.5, 143.5, 128.0, 127.3, 124.6, 120.3, 108.6, 80.3, 51.6, 33.4, 27.5, 21.2 ppm. **IR (ATR)** $\tilde{\nu}$ = 2952,1734, 1437, 1365, 1264, 1240, 1198, 1170, 1090, 745 cm^{-1}. **MS (EI)** *m/z* (%) = 249.1 (44) $[M]^+$, 160.1 (51), 115.1 (61), 91. 1 (47), 83. 1 (37), 73.1 (72), 59.1 (55), 55.1 (100). **HRMS (ESI)** *m/z* calcd. for $C_{12}H_{15}N_3O_3$ $[M+H]^+$ 250.1186, found 250.1191.

5-((1*H*-Benzo[*d*][1,2,3]triazol-1-yl)oxy)-1-phenylpentan-1-one (3qa)

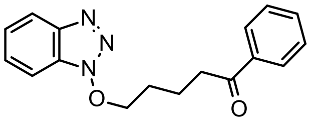

Compound **3qa** was prepared following the general procedure (*conditions B*) from 5-benzoylpentanoic acid **1q** (103 mg, 0.50 mmol) yielding **3qa** as a yellow solid (53 mg, 36%, mp 95 – 96 °C); *Conditions C*: **1q** (258 mg, 1.25 mmol) to give **3qa** (59 mg, 40%). **^{1}H NMR (300 MHz, $CDCl_3$)** δ = 8.03 – 7.92 (m, 3H), 7.60 – 7.33 (m, 6H), 4.59 (t, *J* = 6.2 Hz, 2H), 3.09 (t, *J* = 6.8 Hz, 2H), 2.09 – 1.90 (m, 4H) ppm. **^{13}C NMR (75 MHz, $CDCl_3$)** δ = 199.5, 143.5, 136.8, 133.2, 128.7, 128.6, 128.0, 127.4, 124.6, 120.2, 108.7, 80.7, 37.8, 27.7, 20.3 ppm. **IR (ATR)** $\tilde{\nu}$ = 3063, 2950, 1683, 1597, 1580, 1448, 1363, 1263, 1239, 1205, 1090, 973, 744, 691 cm^{-1}. **HRMS (ESI)** *m/z* calcd. for $C_{17}H_{17}N_3O_2$ $[M+H]^+$ 296.1394, found 296.1391.

1-((1*H*-Benzo[*d*][1,2,3]triazol-1-yl)oxy)propan-2-ol (3ra) [CAS: 1866463-44-6]

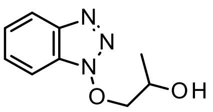

Compound **3ra** was prepared following the general procedure (*conditions B*) from 3-hydroxybutyric acid **1r** (46 µL, 0.50 mmol) yielding **3ra** as an orange oil (43 mg, 45%); *Conditions C*: **1r** (116 µL, 1.25 mmol) to give **3ra** (51 mg, 53%). **^{1}H NMR (300 MHz, $CDCl_3$)** δ = 8.0 (dt, *J* = 8.4, 0.8 Hz, 1H), 7.63 (dt, *J* = 8.3,

0.9 Hz, 1H), 7.54 – 7.48 (m, 1H), 7.43 – 7.34 (m, 1H), 4.50 (dd, *J* = 10.0, 2.5 Hz, 1H), 4.39 – 4.24 (m, 2H), 3.03 (bs, 1H), 1.29 (d, *J* = 6.3 Hz, 3H) ppm. **^{13}C NMR (75 MHz, $CDCl_3$)** δ = 143.5, 128.3, 127.3, 124.9, 120.3, 108.8, 85.4, 65.0, 18.7 ppm. **IR (ATR)** $\tilde{\nu}$ = 3367, 2976, 2937, 1446, 1374, 1265, 1240, 1155, 1093, 980, 783, 767, 742 cm^{-1}. **MS (EI)** *m/z* (%) = 1943.0 (3) [M]$^+$, 165.1 (11), 119. 1 (100). **HRMS (ESI)** *m/z* calcd. for $C_9H_{11}N_3O_2$ [M+H]$^+$ 194.0924, found 194.0924.

1-((2-Methyl-1,3-dioxolan-2-yl)methoxy)-1*H*-benzo[*d*][1,2,3]triazole (3sa)

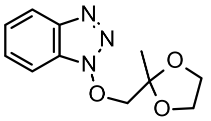

Compound **3sa** was prepared following the general procedure (*conditions B*) from 2-methyldioxolan-2-ylacetic acid **1s** (73 mg, 0.50 mmol) yielding **3sa** as a yellow oil (64 mg, 54%); *Conditions C*: **1s** (183 mg, 1.25 mmol) to give **3sa** (75 mg, 64%). **^{1}H NMR (300 MHz, $CDCl_3$)** δ = 7.99 (dt, *J* = 8.4, 0.9 Hz, 1H), 7.63 (dt, *J* = 8.3, 1.0 Hz, 1H), 7.54 – 7.46 (m, 1H), 7.41 – 7.33 (m, 1H), 4.46 (s, 2H), 4.04 – 3.99 (m, 4H), 1.58 (s, 3H) ppm. **^{13}C NMR (75 MHz, $CDCl_3$)** δ = 143.5, 128.0, 127.2, 124.7, 120.3, 109.0, 106.8, 82.1, 65.4, 22.2 ppm. **IR (ATR)** $\tilde{\nu}$ = 2988, 2891, 1446, 1379, 1263, 1226, 1090, 1049, 982, 853, 744 cm^{-1}. **MS (EI)** *m/z* (%) = 235.1 (5) [M]$^+$, 107. 1 (27), 101.1 (13), 91.1 (11), 87.1 (100). **HRMS (ESI)** *m/z* calcd. for $C_{11}H_{13}N_3O_3$ [M+H]$^+$ 236.1030, found 236.1031.

Methyl 4-((1*H*-benzo[*d*][1,2,3]triazol-1-yl)oxy)-2-((*tert*-butoxycarbonyl)amino)butanoate (3ta)

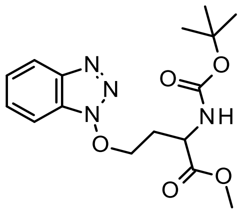

Compound **3ta** was prepared following the general procedure (*conditions B*) from the pre-synthesised salt dicyclohexylammonium 4-((*tert*-butoxycarbonyl)amino)-5-methoxy-5-oxopentanoate **1t** (131 mg, 0.500 mmol) yielding **3ta** as an orange oil (74 mg, 42%); *Conditions C*: **1t** (328mg, 1.25 mmol) to give **3ta** (82 mg, 47%). **^{1}H NMR (300 MHz, $CDCl_3$)** δ = 7.99 (d, *J* = 8.4 Hz, 1H), 7.68 (d, *J* = 8.3 Hz, 1H), 7.54 – 7.46 (m, 1H), 7.42 – 7.34 (m, 1H), 5.40 – 5.30 (m, 1H), 4.64 (t, *J* = 6.0 Hz, 1H), 3.77 (s, 3H), 2.58 – 2.17 (m, 2H), 1.44 (s, 9H) ppm. **^{13}C NMR (75 MHz, $CDCl_3$)** δ = 172.5, 155.5, 143.5, 128.2, 127.3, 124.8, 120.3, 109.0, 76.9, 52.8, 50.8, 31.3, 28.4 ppm. **IR (ATR)** $\tilde{\nu}$ = 3347, 2977, 1742,1707, 1514, 1446, 1366, 1240, 1158, 745 cm^{-1}. **HRMS (ESI)** *m/z* calcd. for $C_{16}H_{22}N_4O_5$ [M+H]$^+$ 351.1663, found 351.1666.

(3*R*,5*R*,8*R*,9*S*,10*S*,12*S*,13*R*,14*S*,17*R*)-17-((*R*)-4-((1*H*-Benzo[*d*][1,2,3]triazol-1-yl)oxy)butan-2-yl)-10,13-dimethylhexadecahydro-1*H*-cyclopenta[*a*]phenanthrene-3,12-diol (3ua)

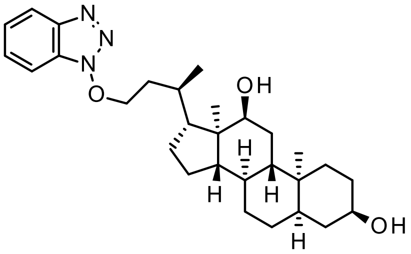

Compound **3ua** was prepared following the general procedure (*conditions B*) from deoxycholic acid **1u** (196 mg, 0.500 mmol) yielding **3ua** as a yellow oil (53 mg, 22%); *Conditions C*: **1u** (490 mg, 1.25 mmol) to give **3ua** (60 mg, 25%). **^{1}H NMR (300 MHz, $CDCl_3$)** δ = 8.01 (d, *J* = 8.4 Hz, 1H), 7.60 – 7.47 (m, 2H), 7.42 – 7.35 (m, 1H), 4.64 – 4.55 (m, 2H), 4.0 (t, *J* = 2.8 Hz, 1H), 3.67 – 3.54 (m, 1H), 2.14 – 2.0 (m,

1H), 1.87 – 1.21 (m, 25H), 1.09 (d, *J* = 6.2 Hz, 3H), 0.90 (s, 3H), 0.71 (s, 3H) ppm. **^{13}C NMR (75 MHz, $CDCl_3$)** δ = 143.6, 128.0, 127.4, 124.7, 120.3, 108.8, 79.4, 73.2, 71.8, 48.3, 47.6, 46.7, 42.1, 36.5, 36.1, 35.3, 34.2, 34.0, 33.7, 32.9, 30.5, 28.9, 27.7, 27.2, 26.2, 23.7, 23.2, 17.9, 12.8 ppm. **IR (ATR)** $\tilde{\nu}$ = 3379 (bs), 2930, 2862, 1736, 1446, 1369, 1264, 1240, 1090, 1042, 944, 740 cm^{-1}. **HRMS (ESI)** *m/z* calcd. for $C_{29}H_{43}N_3O_3$ $[M+H]^+$ 482.3377, found 482.3373.

(8*R*,9*S*,10*S*,13*R*,14*S*,17*R*)-17-((*R*)-4-((1*H*-Benzo[*d*][1,2,3]triazol-1-yl)oxy)butan-2-yl)-10,13-dimethyldecahydro-1*H*-cyclopenta[*a*]phenanthrene-3,7,12(2*H*,4*H*,8*H*)-trione (3va)

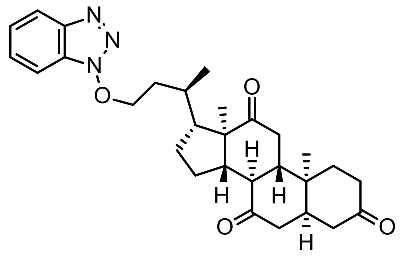

Compound **3va** was prepared following the general procedure (*conditions B*) from dehydrocholic acid **1v** (201 mg, 0.500 mmol) yielding **3va** as a colorless solid (71 mg, 29%, mp 215 – 216 °C); *Conditions C*: **1v** (503 mg, 1.25 mmol) to give **3va** (79 mg, 32%). **^{1}H NMR (300 MHz, $CDCl_3$)** δ = 8.01 (d, *J* = 8.4 Hz, 1H), 7.61 – 7.48 (m, 2H), 7.41 – 7.35 (m, 1H), 4.63 – 4.56 (m, 2H), 2.96 – 2.81 (m, 4H), 2.38 – 2.22 (m, 6H), 2.21 – 1.99 (m, 8H), 1.96 – 1.81 (m, 2H), 1.69 – 1.60 (m, 3H), 1.40 (s, 3H), 1.11 (s, 3H), 0.97 (d, *J* = 6.2 Hz, 3H) ppm. **^{13}C NMR (75 MHz, $CDCl_3$)** δ = 212.0, 209.1, 208.7, 143.6, 128.0, 127.4, 124.7, 120.4, 108.7, 79.2, 57.0, 51.9, 49.0, 46.9, 46.0, 45.6, 45.0, 42.9, 38.7, 36.6, 36.1, 35.3, 33.7, 33.1, 27.8, 25.2, 22.0, 19.1, 11.9 ppm. **IR (ATR)** $\tilde{\nu}$ = 2962, 1707, 1433, 1384, 1266, 1091, 747 cm^{-1}. **HRMS (ESI)** *m/z* calcd. for $C_{29}H_{37}N_3O_4$ $[M+H]^+$ 492.2857, found 492.2859.

(*E*)-1-(Hexadec-7-en-1-yloxy)-1*H*-benzo[*d*][1,2,3]triazole (3wa)

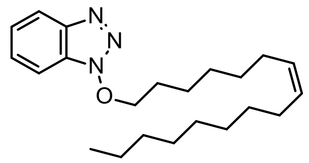

Compound **3wa** was prepared following the general procedure (*conditions B*) from oleic acid **1w** (159 µL, 0.500 mmol) yielding **3wa** as a yellow oil (70 mg, 39%); *Conditions C*: **1w** (397 µL, 1.25 mmol) to give **3wa** (70 mg, 39%). **^{1}H NMR (300 MHz, $CDCl_3$)** δ = 8.0 (dt, *J* = 8.4, 0.9 Hz, 1H), 7.58 – 7.46 (m, 2H), 7.41 – 7.33 (m, 1H), 5.41 – 5.27 (m, 2H), 4.53 (t, *J* = 6.7 Hz, 2H), 2.09 – 1.95 (m, 4H), 1.91 – 1.79 (m, 2H), 1.60 – 1.48 (m, 2H), 1.39 – 1.22 (m, 16H), 0.86 (t, *J* = 6.7 Hz, 3H) ppm. **^{13}C NMR (101 MHz, $CDCl_3$)** δ = 143.6, 130.2, 129.7, 127.9, 127.5, 124.6, 120.3, 108.7, 81.0, 31.9, 29.8, 29.7, 29.6, 29.4, 29.1, 29.1, 28.1, 27.3, 27.2, 25.6, 22.7, 14.1 ppm. **IR (ATR)** $\tilde{\nu}$ = 2923, 2853, 1464, 1446, 1364, 1264, 1240, 1087, 741 cm^{-1}. **HRMS (ESI)** *m/z* calcd. for $C_{22}H_{35}N_3O$ $[M+H]^+$ 358.2853, found 358.2847.

Secondary carboxylic acids

Synthesis of 1-Isopropoxy-1*H*-benzo[*d*][1,2,3]triazole (3xa) [CAS: 57223-17-3]

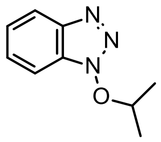

Compound **3xa** was prepared following the general procedure (*conditions D*) from isobutyric acid **1x** (116 µL, 1.25 mmol) yielding **3xa** as an orange oil (34 mg, 38%). **^{1}H NMR (250 MHz, $CDCl_3$)** δ = 8.04 (d, *J* = 8.4 Hz, 1H), 7.64 – 7.47 (m, 2H), 7.44 – 7.33 (m, 1H), 4.97 (hept, *J* = 6.2 Hz, 1H), 1.47 (d, *J* = 6.2 Hz, 6H) ppm. **^{13}C NMR (63 MHz, $CDCl_3$)** δ = 143.5, 128.6, 128.0, 124.5, 120.3, 109.0, 84.0, 21.0 ppm. **IR (ATR)** $\tilde{\nu}$ = 2983, 2936, 1146, 1378, 1265, 1241, 1086, 900, 744 cm^{-1}. **MS (EI)** *m/z* (%) = 177.1 (13) [M]$^+$, 107.1 (91), 91.1 (33), 79.1 (22), 77.1 (66), 57.2 (100). **HRMS (ESI)** *m/z* calcd. for $C_{11}H_{15}N_3O$ [M+H]$^+$ 178.0975, found 178.0976.

1-(Pentan-3-yloxy)-1*H*-benzo[*d*][1,2,3]triazole (3ya)

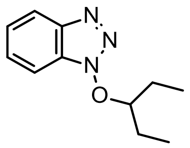

Compound **3ya** was prepared following the general procedure (*conditions D*) from 2-ethylbutyric acid **1y** (158 µL, 1.25 mmol) yielding **3ya** as an orange oil (47 mg, 46%). **^{1}H NMR (300 MHz, $CDCl_3$)** δ = 7.99 (dt, *J* = 8.4, 0.9 Hz, 1H), 7.58 – 7.44 (m, 2H), 7.39 – 7.32 (m, 1H), 4.51 (p, *J* = 5.9 Hz, 1H), 1.88 – 1.68 (m, 4H), 1.08 (t, *J* = 7.4 Hz, 6H) ppm. **^{13}C NMR (75 MHz, $CDCl_3$)** δ = 143.5, 128.4, 127.8, 124.5, 120.3, 109.0, 93.7, 24.7, 9.3 ppm. **IR (ATR)** $\tilde{\nu}$ = 2970, 2938, 2881, 1461, 1445, 1264, 1240, 1085, 920, 892, 767, 740 cm^{-1}. **HRMS (ESI)** *m/z* calcd. for $C_{11}H_{15}N_3O$ [M+H]$^+$ 206.1288, found 206.1288.

1-(Heptan-4-yloxy)-1*H*-benzo[*d*][1,2,3]triazole (3za)

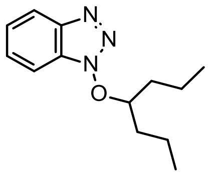

Compound **3za** was prepared following the general procedure (*conditions D*) from valproic acid **1z** (196 µL, 1.25 mmol)) yielding **3za** as a yellow oil (43 mg, 37%). **^{1}H NMR (300 MHz, $CDCl_3$)** δ = 8.0 (dt, *J* = 8.4, 0.8 Hz, 1H), 7.57 – 7.45 (m, 2H), 7.40 – 7.33 (m, 1H), 4.63 (p, *J* = 5.9 Hz, 1H), 1.79 – 1.64 (m, 4H), 1.64 – 1.47 (m, 4H), 0.95 (t, *J* = 7.3 Hz, 6H) ppm. **^{13}C NMR (75 MHz, $CDCl_3$)** δ = 143.5, 128.5, 127.9, 124.5, 120.3, 108.9, 91.3, 34.6, 18.4, 14.1 ppm. **IR (ATR)** $\tilde{\nu}$ = 2960, 2935, 2874, 1465, 1445, 1265, 1240, 1087, 925, 742 cm^{-1}. **MS (EI)** *m/z* (%) = 233.2 (4) [M]$^+$, 107.1 (92), 91.1 (32), 79.1 (21), 77.1 (66), 57.2 (100). **HRMS (ESI)** *m/z* calcd. for $C_{13}H_{19}N_3O$ [M+H]$^+$ 234.1601, found 234.1603.

1-(Hexan-2-yloxy)-1*H*-benzo[*d*][1,2,3]triazole (3a′a)

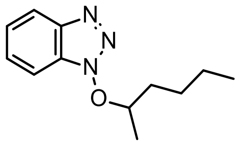

Compound **3a′a** was prepared following the general procedure (*conditions D*) from 2-methylhexanoic acid **1a′** (177 µL, 1.25 mmol) yielding **3a′a** as a yellow oil (36 mg, 33%). **^{1}H NMR (300 MHz, $CDCl_3$)** δ = 8.0 (dt, *J* = 8.4, 0.9 Hz, 1H), 7.57 – 7.45 (m, 2H), 7.40 – 7.33 (m, 1H), 4.75 (h, *J* = 6.2 Hz, 1H), 1.95 – 1.81 (m, 1H), 1.77 – 1.63 (m, 1H), 1.59 – 1.46 (m, 2H), 1.46 – 1.37 (m, 2H), 1.36 (d, *J* = 6.2 Hz, 3H), 0.93 (t, *J* = 7.2 Hz, 3H) ppm. **^{13}C NMR (75 MHz, $CDCl_3$)** δ = 143.5, 128.5, 127.9, 124.5, 120.3, 109.0, 87.9, 34.6, 27.5, 22.7, 19.1, 14.0 ppm. **IR (ATR)** $\tilde{\nu}$ = 2957, 2934, 2872, 1715, 1446, 1380, 1265, 1240, 1158, 1088, 780 743 cm^{-1}. **MS (EI)** *m/z* (%) = 219.2 (18) [M]$^+$, 109.1 (35), 107.1 (100). **HRMS (ESI)** *m/z* calcd. for $C_{12}H_{17}N_3O$ [M+H]$^+$ 220.1444, found 220.1445.

1-((1-Phenylpropan-2-yl)oxy)-1*H*-benzo[*d*][1,2,3]triazole (3b′a)

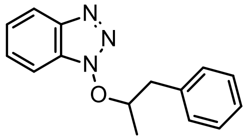

Compound **3b′a** was prepared following the general procedure (*conditions D*) from α-methylhydrocinnamic acid **1b′** (205 mg, 1.25 mmol) yielding **3b′a** as a yellow oil (47 mg, 37%). **^{1}H NMR (300 MHz, $CDCl_3$)** δ = 7.98 (dt, *J* = 8.1, 1.0 Hz, 1H), 7.43 – 7.27 (m, 7H), 7.14 – 7.10 (m, 1H), 4.95 – 4.82 (m, 1H), 3.22 (dd, *J* = 14.0, 6.9 Hz, 1H), 3.02 (dd, *J* = 14.0, 6.3 Hz, 1H), 1.43 (d, *J* = 6.2 Hz, 3H) ppm. **^{13}C NMR (75 MHz, $CDCl_3$)** δ = 143.4, 136.9, 129.6, 128.7, 128.2, 127.9, 127.0, 124.5, 120.2, 108.9, 88.4, 41.5, 19.0 ppm. **IR (ATR)** $\tilde{\nu}$ = 3029, 2980, 2932, 1496, 1446, 1381, 1265, 1240, 1089, 743, 700 cm^{-1}. **MS (EI)** *m/z* (%) = 253.1 (2) [M]$^+$, 180.1 (14), 119.1 (14), 92.1 (12), 91.1 (100). **HRMS (ESI)** *m/z* calcd. for $C_{15}H_{15}N_3O$ [M+H]$^+$ 254.1288, found 254.1286.

1-Cyclopropoxy-1*H*-benzo[*d*][1,2,3]triazole (3c′a) [CAS: 1864424-46-3]

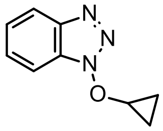

Compound **3c′a** was prepared following the general procedure (*conditions D*) from cyclopropanecarboxylic acid **1c′** (100 µL, 1.25 mmol) yielding **3c′a** as an orange oil (25 mg, 28%). Noteworthy, the yield could be improved to 40% by using NaOH (102 mg, 2.5 mmol) as the base. **^{1}H NMR (300 MHz, $CDCl_3$)** δ = 8.01 (dt, *J* = 8.4, 0.9 Hz, 1H), 7.59 – 7.47 (m, 2H), 7.41 – 7.34 (m, 1H), 4.65 (tt, *J* = 6.1, 2.6 Hz, 1H), 1.30 – 1.22 (m, 2H), 0.82 – 0.74 (m, 2H) ppm. **^{13}C NMR (75 MHz, $CDCl_3$)** δ = 143.5, 128.1, 127.5, 124.6, 120.3, 108.7, 63.4, 6.7 ppm. **IR (ATR)** $\tilde{\nu}$ = 3018, 1445, 1340, 1265, 1240, 1148, 1089, 1021, 817, 782, 767, 741 cm^{-1}. **MS (EI)** *m/z* (%) = 175.1 (19) [M]$^+$, 147. 1 (80), 119.1 (40), 106.1 (44), 91.1 (100). **HRMS (ESI)** *m/z* calcd. for $C_9H_9N_3O$ [M+H]$^+$ 176.0818, found 176.0818.

1-(Cyclopentyloxy)-1*H*-benzo[*d*][1,2,3]triazole (3d′a) [CAS: 60455-02-9]

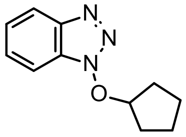

Compound **3d′a** was prepared following the general procedure (*conditions D*) from cyclopentanecarboxylic acid **1d′** (136 μL, 1.25 mmol) yielding **3d′a** as an orange oil (36 mg, 35%). **^{1}H NMR (300 MHz, CDCl$_3$)** δ = 8.0 (dt, *J* = 8.4, 0.9 Hz, 1H), 7.58 – 7.45 (m, 2H), 7.41 – 7.32 (m, 1H), 5.30 – 5.23 (m, 1H), 2.12 – 1.90 (m, 4H), 1.88 – 1.67 (m, 4H) ppm. **^{13}C NMR (75 MHz, CDCl$_3$)** δ = 143.5, 128.3, 127.9, 124.5, 120.3, 108.8, 93.6, 31.5, 23.6 ppm. **HRMS (ESI)** *m/z* calcd. for $C_{11}H_{13}N_3O$ $[M+H]^+$ 204.1131, found 204.1132. The analytical data (NMR, HRMS analysis) matched those reported in the literature.[19]

1-(Cyclohexyloxy)-1*H*-benzo[*d*][1,2,3]triazole (3e′a) [CAS: 1876594-76-1]

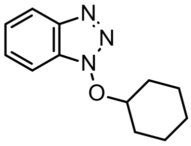

Compound **3e′a** was prepared following the general procedure (*conditions D*) from cyclohexanecarboxylic acid **1e′** (156 μL, 1.25 mmol) yielding **3e′a** as an orange oil (35 mg, 32%). **^{1}H NMR (300 MHz, CDCl$_3$)** δ = 8.0 (dt, *J* = 8.4, 0.9 Hz, 1H), 7.58 – 7.45 (m, 2H), 7.40 – 7.33 (m, 1H), 4.62 (tt, *J* = 9.5, 3.9 Hz, 1H), 2.07 – 1.95 (m, 2H), 1.93 – 1.80 (m, 2H), 1.77 – 1.52 (m, 3H), 1.41 – 1.24 (m, 3H) ppm. **^{13}C NMR (75 MHz, CDCl$_3$)** δ = 143.5, 128.6, 127.9, 124.5, 120.2, 109.1, 88.9, 30.9, 25.2, 23.7 ppm. **IR (ATR)** $\tilde{\nu}$ = 2936, 2859, 1446, 1362, 1239, 1088, 1007, 932, 912, 899, 781, 767, 742 cm^{-1}. **MS (EI)** *m/z* (%) = 217.2 (10) $[M]^+$, 107.1 (79), 91.1 (28), 83.1 (43), 81.1 (42), 79.1 (27), 77.1 (65), 55.1 (100). **HRMS (ESI)** *m/z* calcd. for $C_{12}H_{15}N_3O$ $[M+H]^+$ 218.1288, found 218.1287.

1-(Difluoromethoxy)-1*H*-benzo[*d*][1,2,3]triazole (3f′a) [CAS: 1861169-68-7]

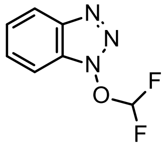

Compound **3f′a** was prepared following the general procedure (*conditions D*) from difluoroacetic acid **1f′** (79 μL, 1.3 mmol) yielding **3f′a** as a yellow oil (37 mg, 40%). **^{1}H NMR (300 MHz, CDCl$_3$)** δ = 8.06 (d, *J* = 8.4 Hz, 1H), 7.58 (s, 2H), 7.45 (d, *J* = 21.7 Hz, 1H), 6.92 (t, *J* = 68.4 Hz, 1H) ppm. **^{13}C NMR (75 MHz, CDCl$_3$)** δ = 143.3, 129.4, 129.1, 125.4, 120.6, 117.5 (t, *J* = 276.3 Hz), 108.9 cm^{-1}. **^{19}F NMR (235 MHz, CDCl$_3$)** δ = −88.72 ppm. **HRMS (ESI)** *m/z* calcd. for $C_7H_6F_2N_3O$ $[M+H]^+$ 186.0473, found 186.0472.

Synthesis of difluoromethoxylating agents

Synthesis of 1-(difluoromethoxy)-6-(trifluoromethyl)-1*H*-benzo[*d*][1,2,3]triazole (3f′b)

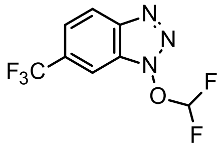

Compound **3f′b** was prepared following the general procedure (*conditions D*) from 6-(trifluoromethyl)-1*H*-benzo[*d*][1,2,3]triazol-1-ol **2b** (102 mg, 0.50 mmol) and difluoroacetic acid **1f′** (79 µL, 1.3 mmol, 2.5 eq.) yielding **3f′b** as a colorless oil (58mg, 46%). **^{1}H NMR (250 MHz, $CDCl_3$)** δ = 8.57 (s, 1H), 8.35 (dd, *J* = 9.1, 2.0 Hz, 1H), 8.24 (d, *J* = 9.1 Hz, 1H), 7.00 (t, *J* = 67.4 Hz, 1H) ppm. **HRMS (ESI)** *m/z* calcd. for $C_8H_5F_5N_3O$ $[M+H]^+$ 254.0347, found 254.0347.

1-(Difluoromethoxy)-6-nitro-1*H*-benzo[*d*][1,2,3]triazole (3f′c)

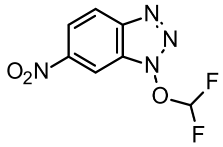

Compound **3f′c** was prepared following the general procedure (*conditions D*) from 6-nitro-1*H*-benzo[*d*][1,2,3]triazol-1-ol **2c** (90 mg, 0.50 mmol) and difluoroacetic acid **1f′** (79 µL, 1.3 mmol, 2.5 eq.) yielding **3f′c** as a colorless solid (30 mg, 26%, mp 71 – 73 °C). **^{1}H NMR (250 MHz, $CDCl_3$)** δ = 8.57 (s, 1H), 8.35 (d, *J* = 9.1 Hz, 1H), 8.24 (d, *J* = 9.1 Hz, 1H), 7.01 (t, *J* = 67.4 Hz, 1H) ppm. **^{13}C NMR (63 MHz, $CDCl_3$)** δ = 148.2, 145.0, 128.6, 121.7, 120.3, 117.0 (t, *J* = 278.7 Hz), 106.2 ppm. **^{19}F NMR (235 MHz, $CDCl_3$)** δ = −87.40 (d, *J* = 67.5 Hz) ppm. **HRMS (ESI)** *m/z* calcd. for $C_7H_5F_2N_4O_3$ $[M+H]^+$ 231.0324, found 231.0325.

4-Chloro-1-(difluoromethoxy)-6-(trifluoromethyl)-1*H*-benzo[*d*][1,2,3]triazole (3f′d)

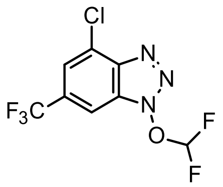

Compound **3f′d** was prepared following the general procedure (*conditions D*) from 4-chloro-6-(trifluoromethyl)-1*H*-benzo[*d*][1,2,3]triazol-1-ol **2d** (119 mg, 0.500 mmol) and difluoroacetic acid **1f′** (79 µL, 1.3 mmol, 2.5 eq.) yielding **3f′d** as colorless solid (55 mg, 38% mp 73 – 74 °C). **^{1}H NMR (250 MHz, $CDCl_3$)** δ = 7.85 (s, 1H), 7.70 (s, 1H), 7.06 (dd, *J* = 101.2, 33.8 Hz, 1H) ppm. **^{13}C NMR (63 MHz, $CDCl_3$)** δ = 142.0, 132.5 (q, *J* = 33.9 Hz), 130.0 (s), 127.9 (q, *J* = 9.0 Hz), 122.9 (q, *J* = 273.5 Hz), 122.2 (q, *J* = 3.2 Hz), 117.3 (t, *J* = 278.7 Hz), 106.0 (q, *J* = 4.5 Hz) ppm. **^{19}F NMR (235 MHz, $CDCl_3$)** δ = −61.71, −86.97 ppm. **HRMS (ESI)** *m/z* calcd. for $C_8H_4ClF_5N_3O$ $[M+H]^+$ 287.9958, found 287.9959.

***In situ* hydrogenation of TPFA (1b)**

Synthesis of 2,2,2-trifluoroethanol (5b[4]) [CAS: 75-89-8]

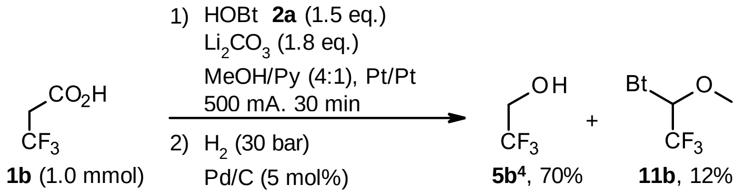

Supplementary Figure 15. *In situ* hydrogenation of **1b**.

A 20 mL vessel equipped with two Pt-electrodes (2.0 × 1.0 cm), was charged with a solution of TFPA **1b** (91 µL, 1.0 mmol), 1-hydroxybenzotriazole monohydrate **2a** (230 mg, 1.50 mmol, 1.50 eq.) and Li_2CO_3 (134 mg, 1.80 mmol, 1.80 eq.) in a 4:1-mixture of MeOH/Py (12 mL). The reaction mixture was electrolyzed at a current of 500 mA for 30 min at room temperature. Assuming a maximum conversion (*i.e.* 68%), the crude was diluted with MeOH until 20 mL to give a 0.034 M solution of **3ba**. An aliquot of 4.0 mL (0.136 mmol of **3ba**) was introduced into a 10 mL vial together with palladium on carbon (10% Pd) (7 mg, 5 mol%). The vial was placed in an autoclave, the mixture was pressurized with hydrogen (30 bar), and stirred at room temperature for 12 h. Analysis by ^{19}F NMR, using trifluoromethoxybenzene (20 µL, 0.15 mmol, 1.1 eq.) as internal standard (Supplementary Figure 16), showing the formation of both **5b[4]** and **11b** in 70% and 12% yield, respectively.

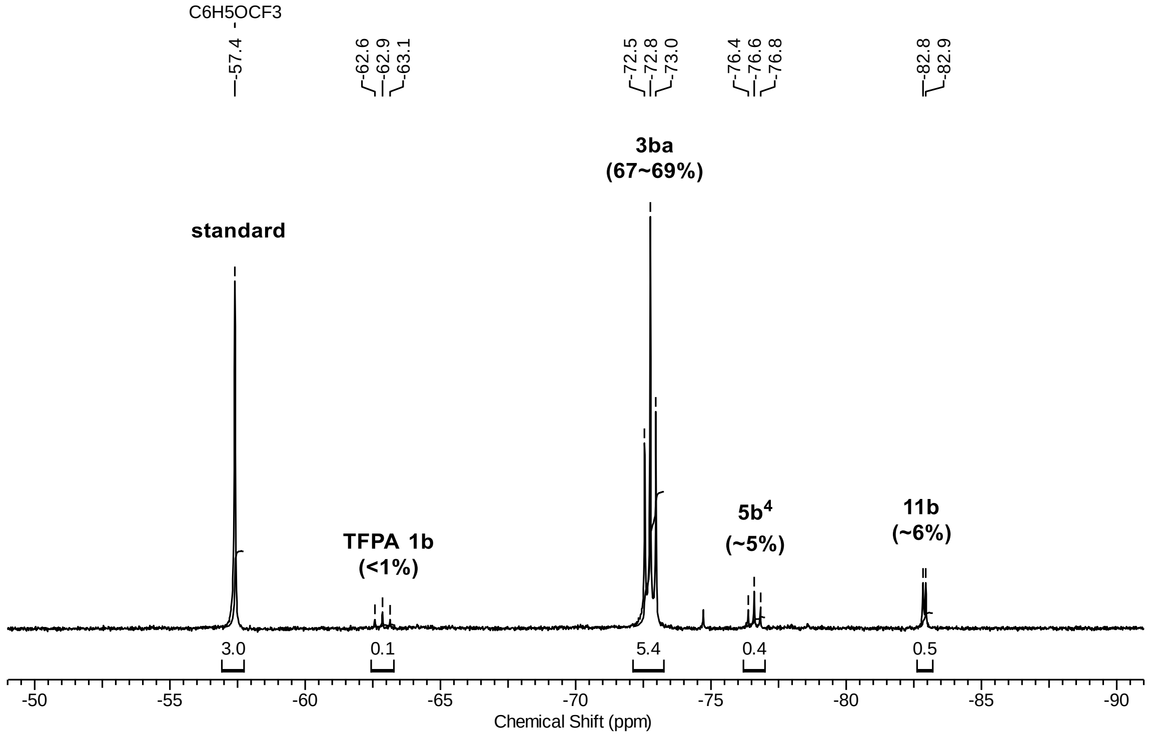

Supplementary Figure 16. Representative ^{19}F NMR-spectrum of electrolysis crude of TPFA (**1b**).

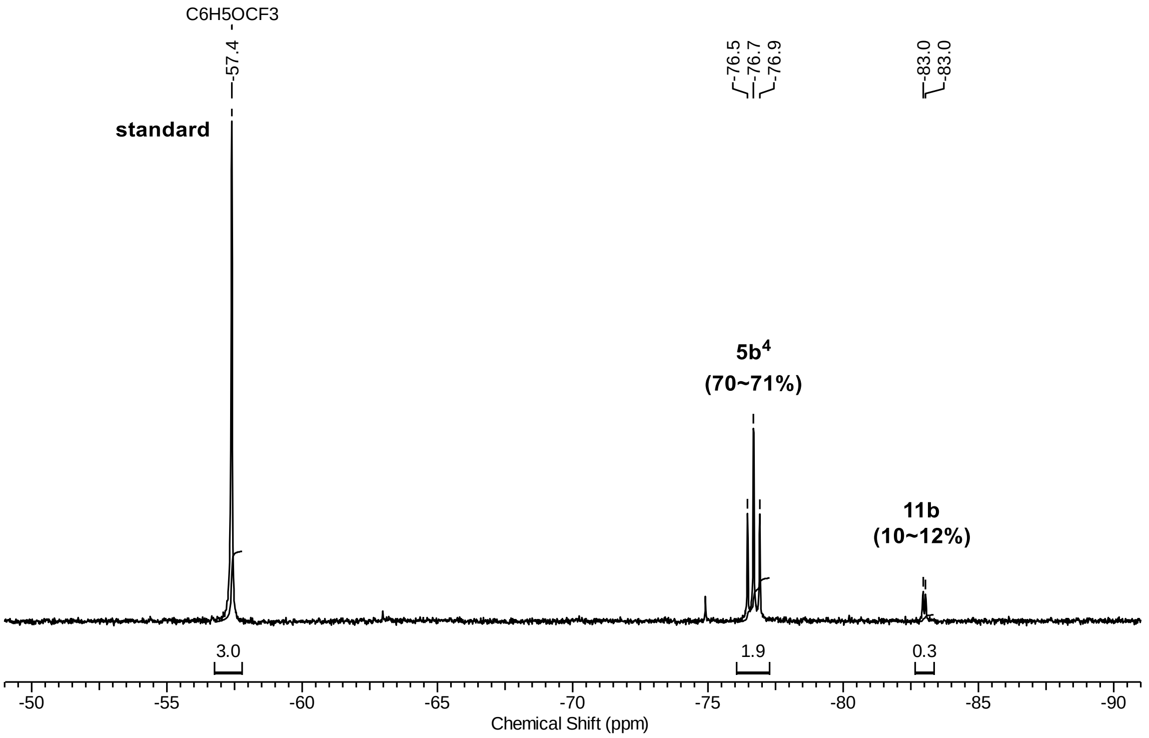

Supplementary Figure 17. ^{19}F NMR spectrum of one-pot electrolysis/hydrogenation crude of TPFA (**1b**).

Samarium-mediated hydroxylation of 3aa

Synthesis of 2-phenylethanol (5a[4]) [CAS: 60-12-8]

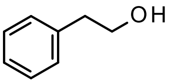

An oven dried 20.0 mL vial was charged with BH_3 in THF (1 M, 1.25 mmol), and additional THF ("freshly dried", 3.0 mL). The content were cooled to 0-5 °C, followed by addition of 1-phenethoxy-1*H*-benzo[*d*][1,2,3]triazole **3aa** (0.50 mmol) *via* syringe within 15 min. The resulting mixture was stirred at room temperature for 12 h, then decomposed with 2 M HCl to give a homogeneous solution. Solvents were removed *in vacuo* and a mixture 1:1 (Et_2O:H_2O) was added. Then, HCl (2 M) was added until acidic pH, and the organic layer was isolated, dried over $MgSO_4$ and dried *in vacuo* give **5a[4]** as a colorless oil with an intense rose-like odor (30 mg, 49%) **^{1}H NMR (300 MHz, $CDCl_3$)** δ = 7.42 – 7.31 (m, 2H), 7.31 – 7.23 (m, 3H), 3.89 (t, *J* = 6.6 Hz, 2H), 2.91 (t, *J* = 6.6 Hz, 2H), 1.66 (s, 1H) ppm. **^{13}C NMR (63 MHz, $CDCl_3$)** δ = 138.6, 129.1, 128.6, 126.5, 63.7, 39.3 ppm. The analytical data (^{1}H NMR and ^{13}C NMR) matched those reported in the literature.[20]

Scale-up

Batch Conditions

According to general procedure (*conditions A*), a 100 mL vessel equipped with two Pt-electrodes (2.0 × 1.0 cm), was charged with a solution of TFPA **1b** (0.45 mL, 5.0 mmol), 1-hydroxybenzotriazole monohydrate **2a** (1.2 mg, 7.5 mmol, 1.5 eq.) and Li_2CO_3 (672 mg, 9.00 mmol, 1.80 eq in a 4:1-mixture of MeOH/Py (60 mL). The reaction mixture was electrolyzed at a current of 500 mA for 2.5 h at room temperature. ^{19}F NMR-analysis of the resulting crude, using trifluoromethoxybenzene (50 µL, 1.5 mmol, 0.075 eq.) as internal standard, showed the formation of the desired product **3ba** in 68% yield (65% isolated).

Continuous Flow Conditions

A 100 mL vessel was charged with a solution of TFPA **1b** (0.45 mL, 5.0 mmol), 1-hydroxybenzotriazole monohydrate **2a** (1.2 mg, 7.5 mmol, 1.5 eq.) and Li_2CO_3 (672 mg, 9.00 mmol, 1.80 eq.) in a 4:1-mixture of MeOH/Py (60 mL). Then solution was injected at a flow rate of 0.4 mL·min^{-1} in a self-designed flow-electrolytic cell (Supplementary Figure 19) consisting on a Pt-anode (1.04 cm^2) and a stainless steel-cathode (1.04 cm^2), separated by 0.5 mm (flow channel volume = 0.52 mL; residence time = 78 s). ^{19}F NMR-analysis of the resulting crude, using trifluoromethoxybenzene (200 µL, 1.52 mmol, 0.300 eq.) as internal standard, showed the formation of the desired product **3ba** in 66% yield (60% isolated).

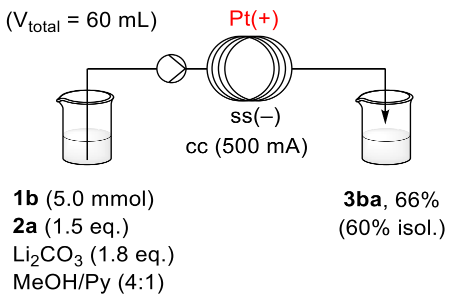

Supplementary Figure 18. Standard reaction (scale-up)

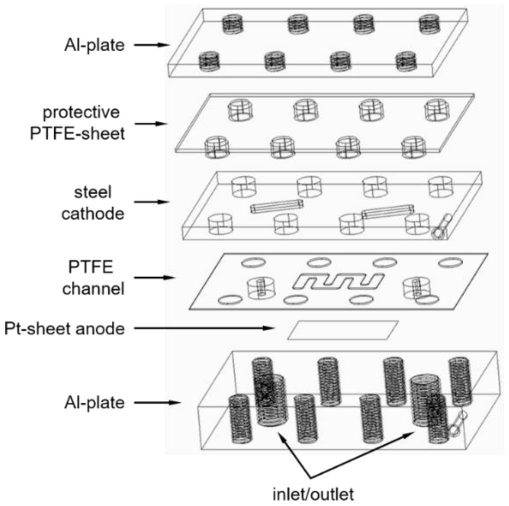

Supplementary Figure 19. Electro-flow reactor

General procedure for the activation of HOBt-derivatives

Synthesis of 3-methyl-1-phenethoxy-1*H*-benzo[*d*][1,2,3]triazol-3-ium trifluoromethane sulfonate (12aa)

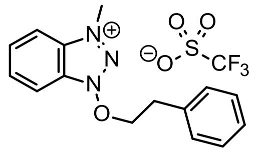

Following a modified procedure described by Woerpel *et al.*,[21] an oven-dried 20 mL vessel was charged with 1-phenethoxy-1*H*-benzo[*d*][1,2,3]triazole **3aa** (1.91 g, 8.00 mmol) and dissolved in DCM (10 mL). Then, to the stirred solution of MeOTf (1.09 mL, 1.20 eq.) was added and the solution was stirred for 2 h. Et_2O was then added dropwise until the solution turned cloudy. More Et_2O (20 mL) was added. The bottom organic layer was washed (2 × 5.0 mL) and concentrated *in vacuo* yielding **12aa** as a colorless oil (2.64 g, 82%). **^{1}H NMR (300 MHz, DMSO-d$_6$)** δ = 8.09 (d, *J* = 8.6 Hz, 1H), 7.87 – 7.74 (m, 2H), 7.47 (d, *J* = 8.5 Hz, 1H), 7.35 – 7.28 (m, 5H), 5.11 (t, *J* = 6.4 Hz, 2H), 4.56 (s, 3H), 3.26 (t, *J* = 6.4 Hz, 2H) ppm. **^{13}C NMR (63 MHz, DMSO-d$_6$)** δ = 136.2, 135.2, 131.8, 131.7, 129.1, 128.9, 128.5, 126.8, 120.67 (d, *J* = 322.3 Hz), 114.3, 111.9, 83.9, 38.1, 33.3 ppm. **^{19}F NMR (235 MHz, DMSO-d$_6$)** δ = −79.36 ppm. **IR (ATR)** $\tilde{\nu}$ = 3070, 1609, 1499, 1456, 1351, 1257, 1223, 1151, 1028, 752, 702, 635, 572, 516 cm^{-1}.

Derivatization of 12aa *via* nucleophilic substitution

Iodination

Synthesis of (2-iodoethyl)benzene (13a) [CAS: 17376-04-4]

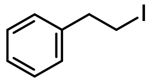

Following a modified procedure from Woerpel *et al.*,[21] an oven-dried 20 mL vessel was charged with 3-methyl-1-phenethoxy-1*H*-benzo[*d*][1,2,3]triazol-3-ium trifluoromethanesulfonate **12aa** (202 mg, 0.500 mmol) and NaI (150 mg, 1.00 mmol, 2.00 eq.). Then, acetone (5.0 mL) was added, and the resulting mixture was stirred at room temperature for 16 h. The mixture was cooled, diluted with water and extracted with DCM. The organic layer was washed with water (2 × 5.0 mL), brine (2 × 5.0 mL), dried over $MgSO_4$, filtered and concentrated *in vacuo*. The resulting residue was purified by flash chromatography yielding **13a** as a colorless oil (57 mg, 50%). **^{1}H NMR (250 MHz, $CDCl_3$)** δ = 7.39 – 7.15 (m, 5H), 3.41 – 3.30 (m, 2H), 3.24 – 3.12 (m, 2H) ppm. **^{13}C NMR (63 MHz, $CDCl_3$)** δ = 140.77, 128.78, 128.46, 127.0, 40.52, 5.65 ppm. The analytical data (^{1}H NMR and ^{13}C NMR) matched those reported in the literature.[22]

Azidation

Synthesis of (2-azidoethyl)benzene (14a) [CAS: 6926-44-9]

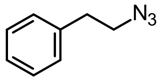

An oven-dried 20 mL vessel was charged with 3-methyl-1-phenethoxy-1*H*-benzo[*d*][1,2,3] triazol-3-ium trifluoromethanesulfonate **12aa** (202 mg, 0.5 mmol), NaN_3 (98 mg, 1.5 mmol, 3.0 eq.) and NaI (374 mg, 2.50 mmol, 5.00 eq.). Then, *N*,*N*-dimethylformamide (5.0 mL) was added *via* syringe, and the resulting mixture was stirred at 80 °C overnight. The mixture was cooled, diluted with water and extracted with DCM. The organic layer was washed with water (2 × 5.0 mL), brine (2 × 5.0 mL), dried over $MgSO_4$, filtered and concentrated *in vacuo*. The resulting residue was purified by flash chromatography yielding **14a** as a yellow oil (41 mg, 56%). **^{1}H NMR (250 MHz, $CDCl_3$)** δ = 7.51 – 7.24 (m, 1H), 3.62 (t, *J* = 7.3 Hz, 1H), 3.02 (t, *J* = 7.3 Hz, 1H) ppm. **^{13}C NMR (63 MHz, $CDCl_3$)** δ = 138.1, 128.8, 128.7, 126.8, 52.5, 35.4 ppm. The analytical data (^{1}H NMR and ^{13}C NMR) matched those reported in the literature.[23]

Thiocyanation

Synthesis of (2-thiocyanatoethyl)benzene (15a) [CAS: 5654-72-8]

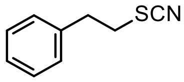

An oven-dried 20 mL vessel was charged with 3-methyl-1-phenethoxy-1*H*-benzo[*d*][1,2,3] triazol-3-ium trifluoromethanesulfonate **12aa** (202 mg, 0.500 mmol) and NaSCN (83 mg, 1.0 mmol, 2.0 eq.). Then, MeCN (5.0 mL) was added *via* syringe, and the resulting mixture was stirred at 80 °C overnight. The mixture was cooled, diluted with water and extracted with DCM. The organic layer was washed with water (2 × 5.0 mL), brine (2 × 5.0 mL), dried over $MgSO_4$, filtered and concentrated *in vacuo*. The resulting residue was purified by flash chromatography yielding **15a** as a yellow oil (76 mg, 94%). **^{1}H NMR (250 MHz, $CDCl_3$)** δ = 7.41 – 7.27 (m, 3H), 7.26 – 7.19 (m, 2H), 3.31 – 2.99 (m, 4H) ppm. **^{13}C NMR (63 MHz, $CDCl_3$)** δ = 137.7, 128.8, 128.7, 127.3, 112.0, 36.0, 35.1 ppm. The analytical data (^{1}H NMR and ^{13}C NMR) matched those reported in the literature.[24]

Selenocyanation

Synthesis of (2-selenocyanatoethyl)benzene (16a) [CAS: 2218515-35-4]

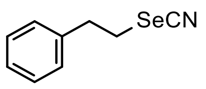

An oven-dried 20 mL vessel was charged with 3-methyl-1-phenethoxy-1*H*-benzo[*d*][1,2,3] triazol-3-ium trifluoromethanesulfonate **12aa** (202 mg, 0.500 mmol) and KSeCN (109 mg, 0.750 mmol, 1.50 eq.). Then, MeCN (5.0 mL) was added *via* syringe, and the resulting mixture was stirred at 80 °C overnight. The mixture was cooled, diluted with water and extracted with DCM. The organic layer was washed with water (2 × 5.0 mL), brine (2 × 5.0 mL), dried over $MgSO_4$, filtered and concentrated *in vacuo*. The resulting residue was purified by flash chromatography yielding **16a** as a dark orange oil (97 mg, 93%). **^{1}H NMR (250 MHz, $CDCl_3$)** δ = 7.49 – 7.28 (m, 5H), 3.43 – 3.25 (m, 4H) ppm. **^{13}C NMR (63 MHz, $CDCl_3$)** δ = 128.9, 128.6, 127.3, 101.4, 36.9, 30.5 ppm. The analytical data (^{1}H NMR and ^{13}C NMR) matched those reported in the literature.[25]

Xanthate esterification

Synthesis of *O*-ethyl *S*-phenethyl carbonodithioate (17a) [CAS: 3278-36-2]

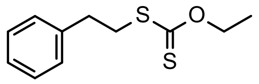

Following a modified procedure from Woerpel *et al.*,[21] an oven-dried 20 mL vessel was charged with 3-methyl-1-phenethoxy-1*H*-benzo[*d*][1,2,3]triazol-3-ium trifluoromethanesulfonate **12aa** (202 mg, 0.500 mmol) and 5.0 mL of toluene. Then, potassium ethyl xanthate (165 mg, 1.00 mmol, 2.00 eq.) was added, and the resulting mixture was stirred at room temperature for 16 h. The mixture was cooled and extracted with DCM. The organic layer was washed with water (2 × 5.0 mL), brine (2 × 5.0 mL), dried over $MgSO_4$, filtered and concentrated *in vacuo*. The resulting residue was purified by flash chromatography yielding **17a** as a pale yellow oil (92 mg, 82%). **^{1}H NMR (300 MHz, $CDCl_3$)** δ = 7.26 – 7.13 (m, 5H), 4.57 (q, *J* = 7.1 Hz, 2H), 3.32 – 3.24 (m, 2H), 2.95 – 2.88 (m, 2H), 1.34 (t, *J* = 7.1 Hz, 3H) ppm. **^{13}C NMR (63 MHz, $CDCl_3$)** δ = 214.7, 139.8, 128.6, 126.6, 69.9, 37.1, 34.9, 13.8 ppm. The analytical data (^{1}H NMR and ^{13}C NMR) matched those reported in the literature.[26]

Esterification

Synthesis of phenethyl benzoate (18a) [CAS: 94-47-3]

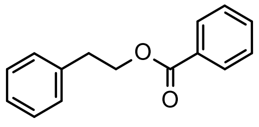

An oven-dried 20 mL vessel was charged with 3-methyl-1-phenethoxy-1*H*-benzo[*d*][1,2,3] triazol-3-ium trifluoromethanesulfonate **12aa** (202 mg, 0.500 mmol) and sodium benzoate (108 mg, 0.750 mmol, 1.50 eq.). Then, MeCN (5.0 mL) was added *via* syringe, and the resulting mixture was stirred at 80 °C overnight. The mixture was cooled, diluted with water and extracted with DCM. The organic layer was washed with water (2 × 5.0 mL), brine (2 × 5.0 mL), dried over $MgSO_4$, filtered and concentrated *in vacuo*. The resulting residue was purified by flash chromatography yielding **18a** as a yellow oil (59 mg, 81%). **^{1}H NMR (250 MHz, $CDCl_3$)** δ = 8.16 – 8.09 (m, 2H), 7.69 – 7.60 (m, 1H), 7.57 – 7.48 (m, 2H), 7.47 – 7.30 (m, 5H), 4.64 (t, *J* = 7.0 Hz, 2H), 3.18 (t, *J* = 7.0 Hz, 2H) ppm. **^{13}C NMR (63 MHz, $CDCl_3$)** δ = 166.6, 138.0, 133.0, 130.4, 129.6, 129.0, 128.6, 128.4, 126.6, 65.5, 35.3 ppm. The analytical data (^{1}H NMR and ^{13}C NMR) matched those reported in the literature.[27]

Amination

Synthesis of *N*-phenethylaniline (19a) [CAS: 1739-00-0]

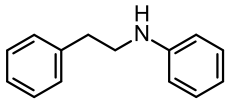

An oven-dried 20 mL vessel was charged with 3-methyl-1-phenethoxy-1*H*-benzo[*d*][1,2,3] triazol-3-ium trifluoromethanesulfonate **12aa** (202 mg, 0.500 mmol). Aniline (205 µL, 2.25 mmol, 4.50 eq.) and MeCN (5.0 mL) were added *via* syringe, and the resulting mixture was stirred at 80 °C overnight. The mixture was cooled, diluted with water and extracted with DCM. The organic layer was washed with water (2 × 5.0 mL), brine (2 × 5.0 mL), dried over $MgSO_4$, filtered and concentrated *in vacuo*. The resulting residue was purified by flash chromatography yielding **19a** as an orange oil (62 mg, 63%). **^{1}H NMR (250 MHz, $CDCl_3$)** δ = 7.48 – 7.26 (m, 7H), 6.83 (t, *J* = 7.3 Hz, 1H), 6.73 (d, *J* = 8.5 Hz, 2H), 3.75 (bs, 1H), 3.52 (t, *J* = 7.0 Hz, 2H), 3.03 (t, *J* = 7.0 Hz, 2H) ppm. **^{13}C NMR (63 MHz, $CDCl_3$)** δ = 148.1, 139.4, 129.4, 128.9, 128.7, 117.5, 113.1, 45.1, 35.6 ppm. The analytical data (^{1}H NMR and ^{13}C NMR) matched those reported in the literature.[28]

Synthesis of 4-phenethylmorpholine (20a) [CAS: 46346-12-7]

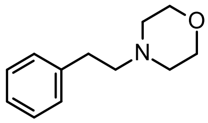

An oven-dried 20 mL vessel was charged with 3-methyl-1-phenethoxy-1*H*-benzo[*d*][1,2,3] triazol-3-ium trifluoromethanesulfonate **12aa** (202 mg, 0.500 mmol). Morpholine (108 µL, 1.25 mmol, 2.50 eq.) and MeCN (5.0 mL) were added *via* syringe, and the resulting mixture was stirred at 80 °C overnight. The mixture was cooled, diluted with water and extracted with DCM. The organic layer was washed with water (2 × 5.0 mL), brine (2 × 5.0 mL), dried over $MgSO_4$, filtered and concentrated *in vacuo*. The resulting residue was purified by flash chromatography yielding **20a** as a pale orange oil (67 mg, 70%). **^{1}H NMR (250 MHz, $CDCl_3$)** δ = 7.32 – 7.13 (m, 5H), 3.76 – 3.68 (m, 4H), 2.84 – 2.74 (m, 2H), 2.62 – 2.54 (m, 2H), 2.53 – 2.46 (m, 4H) ppm. **^{13}C NMR (63 MHz, $CDCl_3$)** δ = 140.2, 128.7, 128.5, 126.1, 67.0, 60.9, 53.8, 33.4 ppm. The analytical data (^{1}H NMR and ^{13}C NMR) matched those reported in the literature.[29]

Mechanistic studies

Radical scavenger experiment

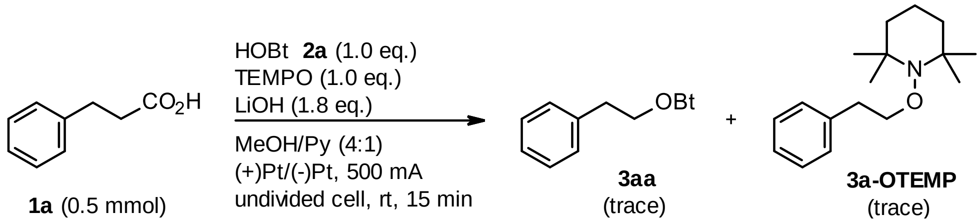

Supplementary Figure 20. Standard electrodecarboxylative etherification of **1a** in the presence of TEMPO.

An oven-dried 20 mL vessel was charged with a solution of hydrocinnamic acid **1a** (71 µL, 0.50 mmol) hydroxybenzotriazole monohydrate **2a** (68 mg, 0.50 mmol, 1.0 eq.), LiOH (22 mg, 0.90 mmol, 1.8 eq.) and TEMPO (80 mg, 0.50 mmol, 1.0 eq.) in a 5:1-mixture of MeOH/Py (12 mL). The reaction vessel was sealed under air with a screw cap provided with two platinum-electrodes (2.0 × 1.0 cm), and a current of 500 mA was applied for 15 min at room temperature. The crude was re-diluted in DCM,

washed with water (2 × 5.0 mL), brine (2 × 5.0 mL), dried over $MgSO_4$, filtered and concentrated *in vacuo*. An aliquot of the resulting mixture was analyzed analyzed by GC-MS (*n*-undecane as the internal standard), evidencing the presence of traces of decarboxylative coupling between **1a** with both the HOBt (**3aa**) and TEMPO (**3a-OTEMP**).

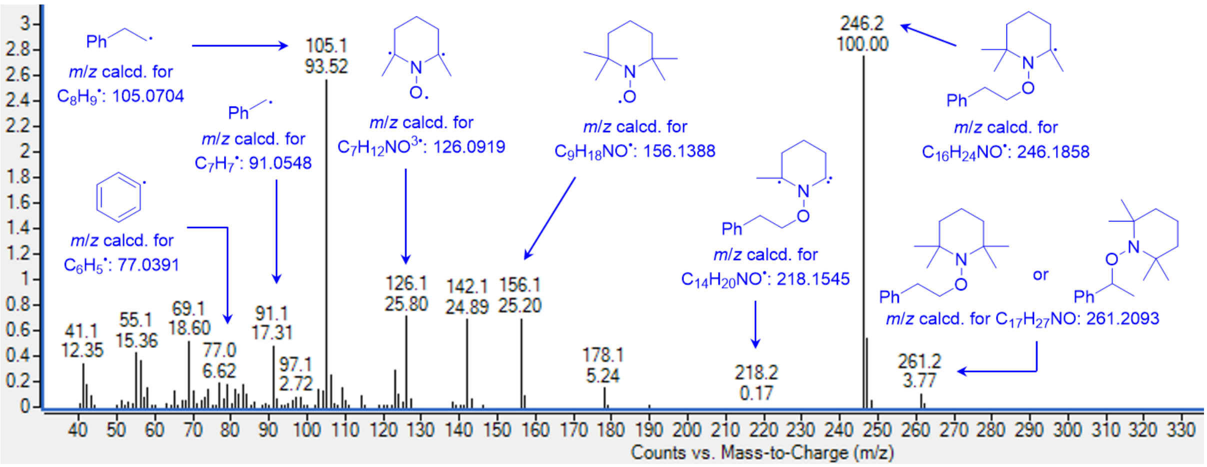

Supplementary Figure 21. EI Mass spectrum of compound **3a-OTEMP**.

Additionally, product **3a-OTEMP** could be isolated as a colorless oil and characterized by flash chromatography (*n*-hexane/EtOAc, 4:1). **^{1}H NMR (250 MHz, $CDCl_3$)** δ = 7.41 – 7.23 (m, 5H), 4.02 (t, *J* = 7.0 Hz, 2H), 2.90 (t, *J* = 7.0 Hz, 2H), 1.70 – 1.29 (m, 6H), 1.14 (s, 12H). **^{13}C NMR (63 MHz, $CDCl_3$)** δ = 139.7, 129.2, 128.2, 126.0, 59.8, 39.7, 35.5, 33.0, 20.2, 17.2 ppm. The analytical data matched those reported in the literature [CAS: 131428-11-0].[30]

Radical clock experiment

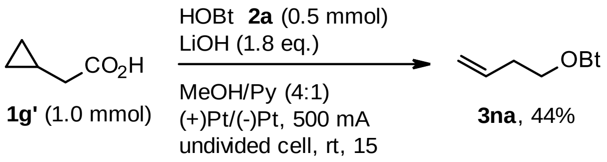

Supplementary Figure 22. Standard electrodecarboxylative etherification of **1g′**.

An oven-dried 20 mL vessel was charged with **2a** (0.50 mmol) and LiOH (2.0 eq.). Then, a mixture 4:1 of MeOH/Py (15 mL) and cyclopropylacetic acid **1g′** (98 µL, 1.3 mmol, 2.5 eq.) were added *via* syringe. The reaction vessel was sealed under air with a screw cap provided with two platinum electrodes (1.0 × 2.0 cm), and a constant current (500 mA) was applied to the stirring vials for 15 min at room temperature. The crude was diluted in EtOAc, washed with a solution of Na_2CO_3 (2 × 5.0 mL), water (2 × 5.0 mL), brine (2 × 5.0 mL), dried over $MgSO_4$, filtered and concentrated *in vacuo*. The resulting residue was purified by flash chromatography yielding 1-(but-3-en-1-yloxy)-1*H*-benzo[*d*][1,2,3]triazole **3na** as the only product. Yellow oil (42 mg, 44%). The analytical data (NMR, HRMS analysis) matched those reported in the literature [CAS: 77204-11-6].[18]

Cyclic voltammetric studies

Cyclovoltammetry measurements were carried out using a Pt-disk working electrode (2 mm diameter, *CH Instruments*) and a Pt-wire counter electrode. A *Haber-Luggin* dual-reference electrode system as described by Speiser *et al*. was used.[31] This system consists of a Ag-wire, which is submerged in a solution of $AgPF_6$ (0.01 M) and Bu_4NPF_6 (0.1 M) in MeCN. The silver wire is connected through a 0.01 µF capacitor to a Pt-wire, whose tip is placed in proximity to the working electrode. The reference solution is connected to the solution of analyte *via* a double junction and a Haber-Luggin capillary. The potential scale was calibrated against external ferrocene (Fc). The samples were measured with

Bu_4NPF_6 (0.1 M) as supporting electrolyte. MeCN was dried by passing through a column of activated alumina using a *MBRAUN Solvent Purification System*. MeOH was dried by stirring with powdered activated molecular sieves (3Å) for 10 days and distilling the resulting mixture. All solvents were degassed by bubbling dry argon into the liquid for at least 15 minutes. The potentiostat was an *EmStat³* from *PalmSens BV*. Unless stated otherwise, a scan rate of 100 mV/s was used, and for each sample, ten potential sweeps between −0.5 and +2.5 V *vs*. Ag/Ag^+ were performed; the first (red trace) and the tenth (blue trace) sweep are displayed. No significant changes are observed over the course of 10 cyclic voltammetry cycles, which demonstrates that the electrode surface is stable at these potentials.

- *Measurements in MeOH:*

If pure MeOH is used as the solvent, strong oxidation of the solvent is observed, which hinders the analysis of the oxidation of the substrates. The oxidation of phenylpropionic acid **1a** is not visible due to overlap with the oxidation of MeOH. On the other hand, HOBt·H_2O **2a** clearly shows an oxidation peak (E_{pa}=1.30 V). Interestingly, a small cathodic peak (E_{pc}=0.12 V) is observed during the return sweep, possibly resulting from reduction of $BtO^•$ radicals.

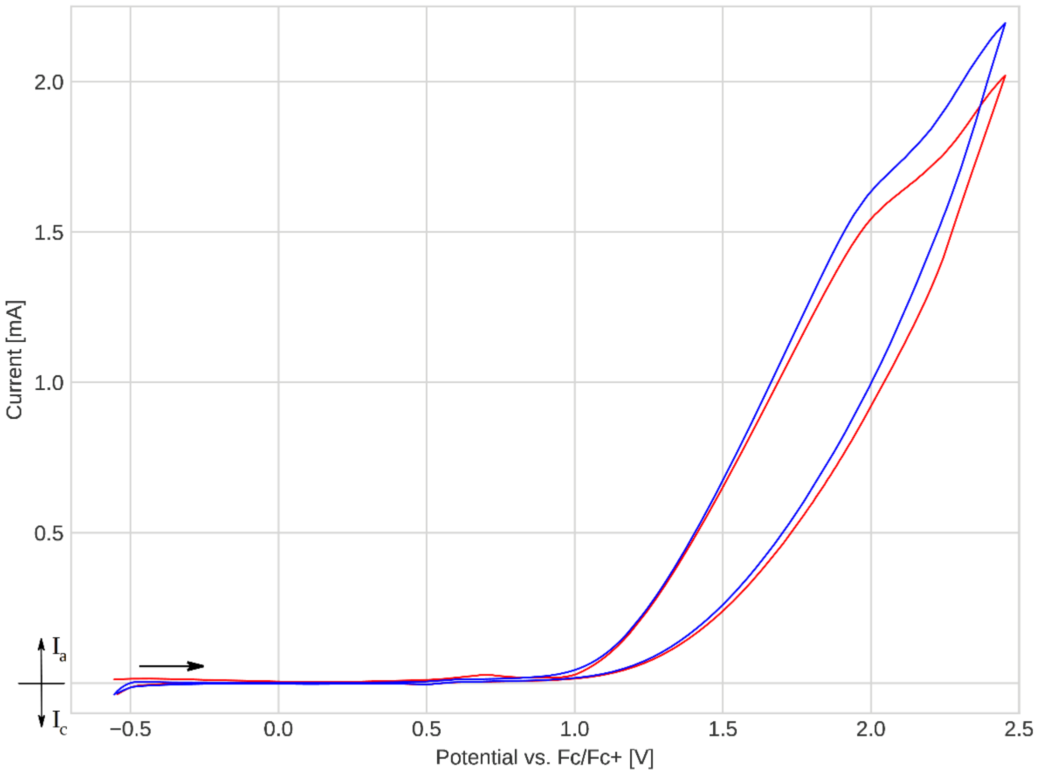

Supplementary Figure 23. Blank (Bu_4NPF_6, MeOH).

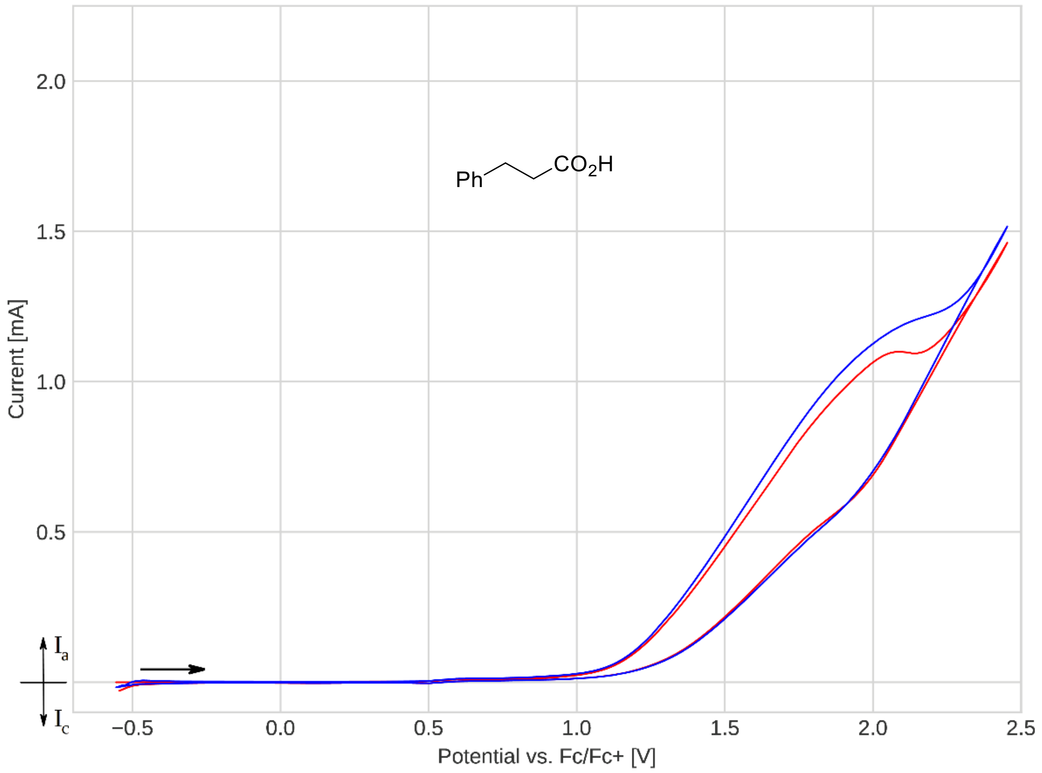

Supplementary Figure 24. Hydrocinnamic acid **1a** (100 mM, MeOH)

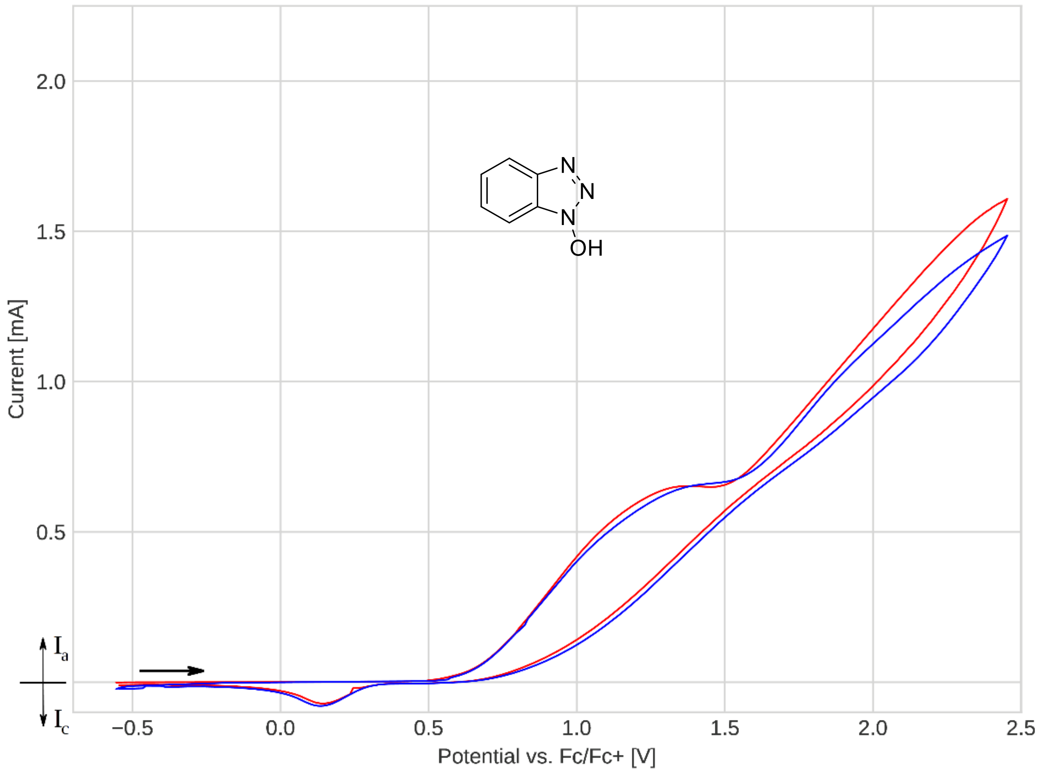

Supplementary Figure 25. HOBt·H_2O **2a** (100 mM, MeOH)

- *Measurements of hydrocinnamic acid **1a** in MeOH/Py (4:1):*

Addition of pyridine has several striking effects on the cyclic voltammograms. In the blank measurement, up to a potential of around 2 V, only relatively small currents are observed compared to pure MeOH. This demonstrates that, while pyridine slowly gets oxidized itself, it suppresses the oxidation of MeOH. The addition of Li_2CO_3 shifts the oxidation peak of the acid to lower potential (from 1.49 to 1.20 V).

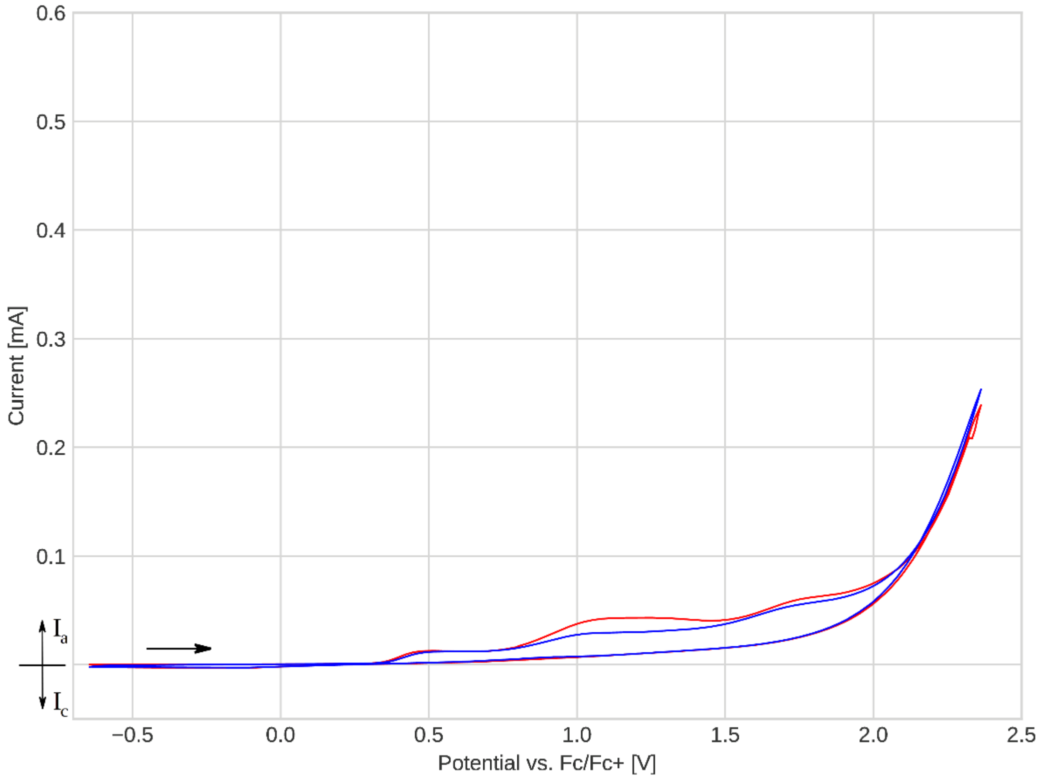

Supplementary Figure 26. Blank (Bu_4NPF_6, MeOH/Pyridine 4:1).

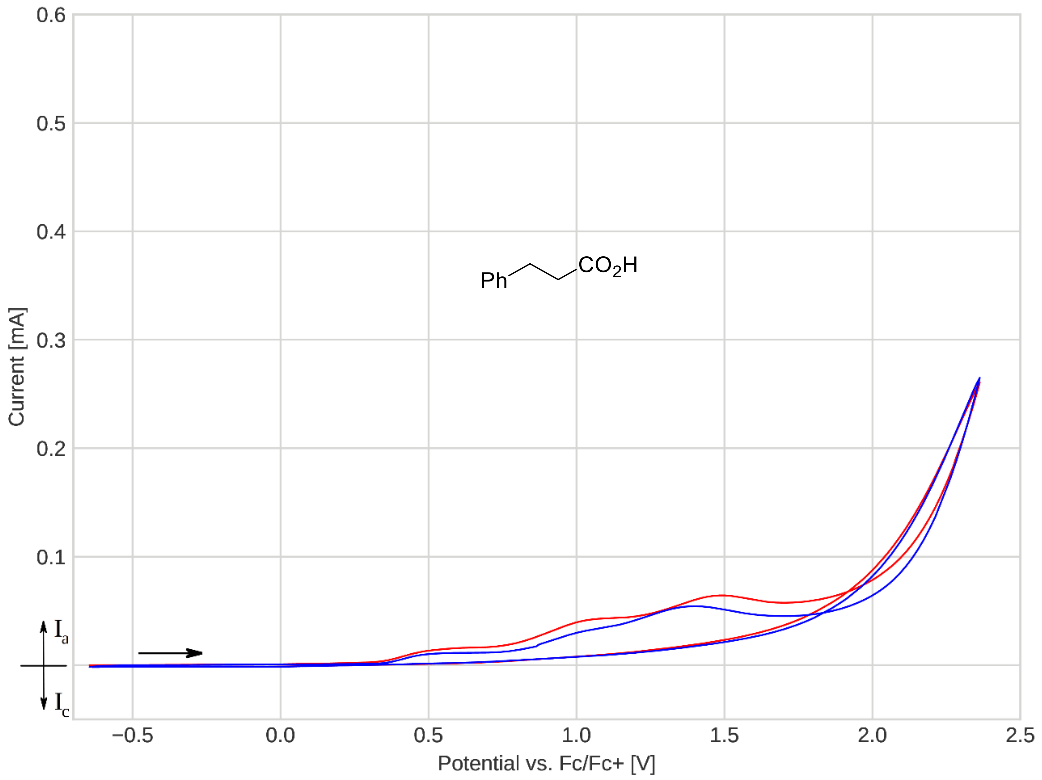

Supplementary Figure 27. Hydrocinnamic acid **1a** (100 mM, MeOH/Pyridine 4:1)

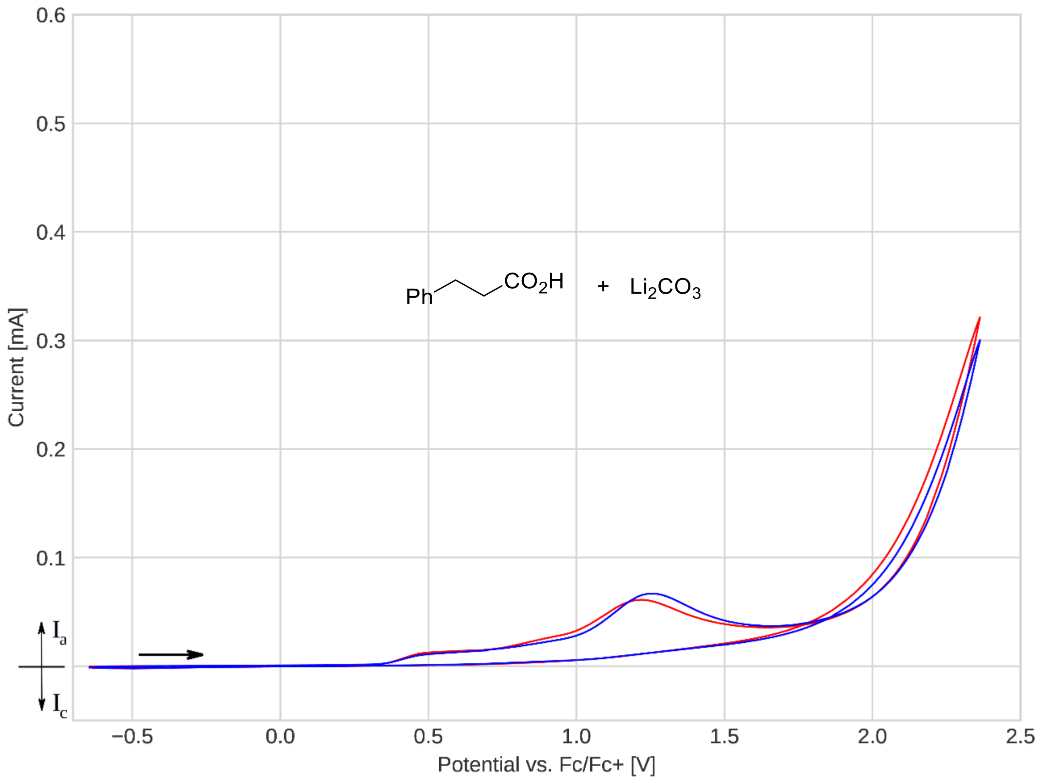

Supplementary Figure 28. 1a (100 mM) + Li_2CO_3 (sat.) (MeOH/Pyridine 4:1)

- *Measurements of HOBt·H_2O **2a** in MeOH/Py (4:1) with varying scan rate:*

These measurements were performed using Bu_4NBF_4 as supporting electrolyte and $AgBF_4$ as reference, instead of their respective PF_6-salts. For the scans with varying scan rate, the potential sweep was performed between –0.5 and +1.0 V vs. Ag/Ag^+, and only the first scans are displayed.

In MeOH/pyridine (4:1), the oxidation potential of HOBt (E_{pa} = 0.71 V) is much lower than in pure MeOH (E_{pa} = 1.30 V). This is likely due to deprotonation of HOBt to BtO^- facilitating anodic oxidation towards the $BtO^•$ radical, which has been previously characterized in literature for H_2O and MeCN solutions.[32–34] At scan rates of 500 mV/s and higher, our measurements show a reduction peak at potentials between 0.1 and 0.22 V, corresponding to the reduction of $BtO^•$ to BtO^-. While Galli *et al.* measured a half-life of 110 seconds in MeCN solution,[34] our measurements in MeOH/pyridine (4:1) indicate a lifetime of $BtO^•$ in the range of hundreds of milliseconds. Addition of Li_2CO_3 results in an increased conductivity, but has no influence on the redox potentials.

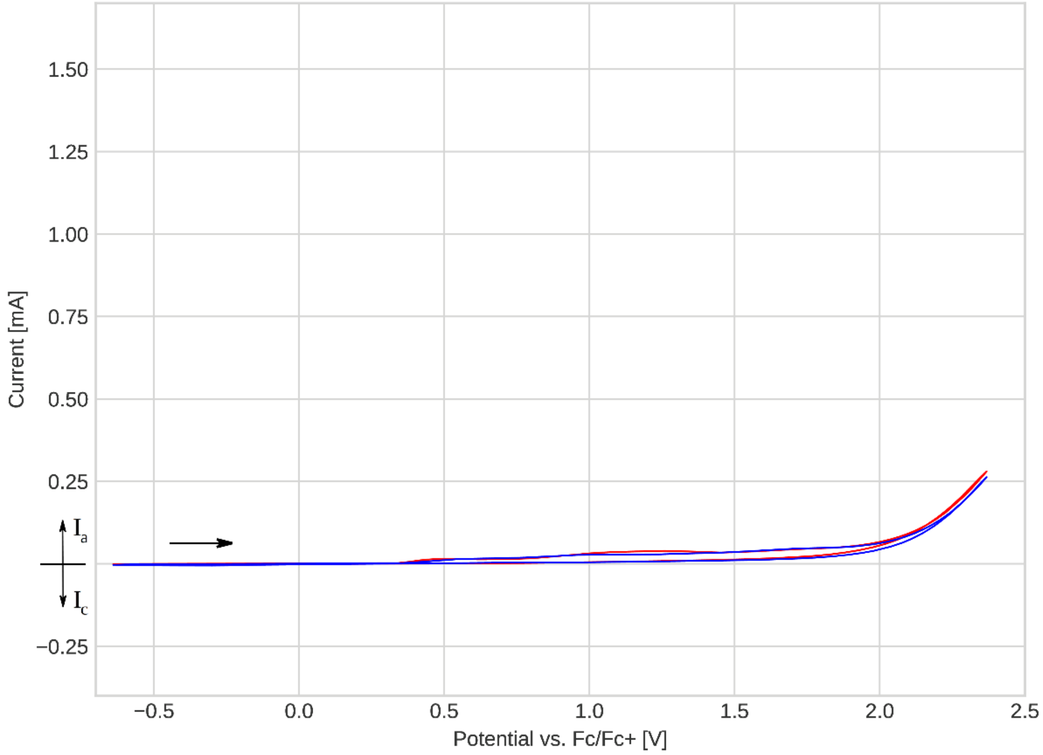

Supplementary Figure 29. Blank (Bu_4NBF_4, MeOH/Pyridine 4:1).

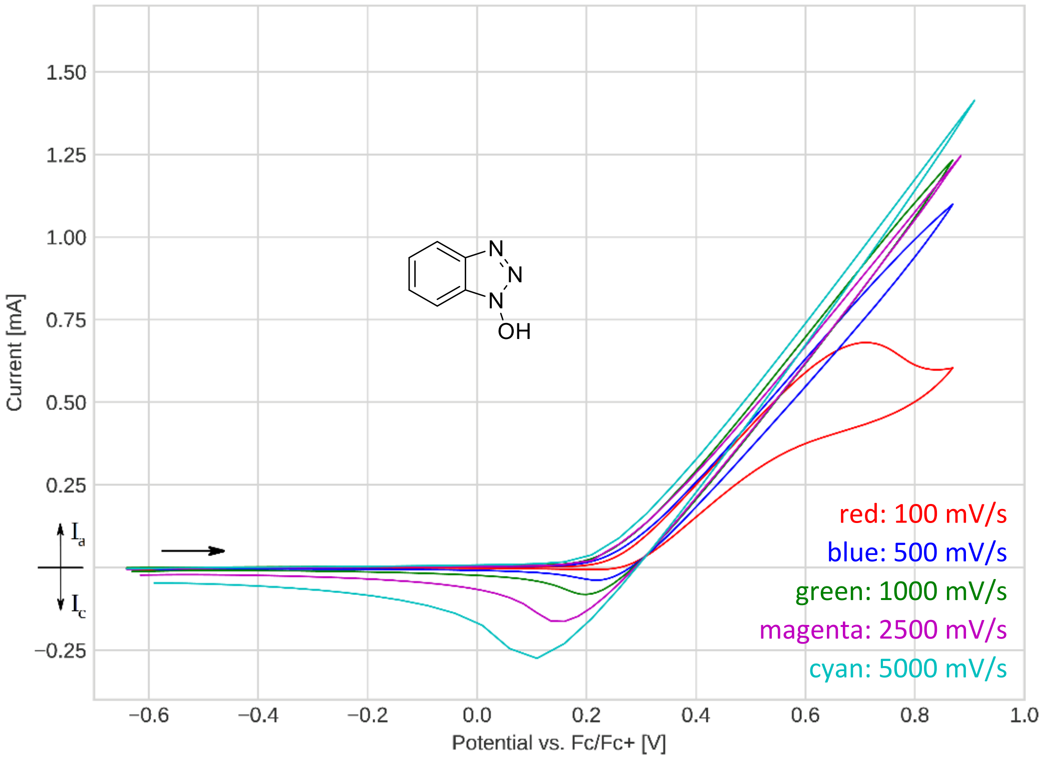

Supplementary Figure 30. Scans of varying scan rates for HOBt·H_2O **2a** (100 mM, MeOH/Pyridine 4:1).

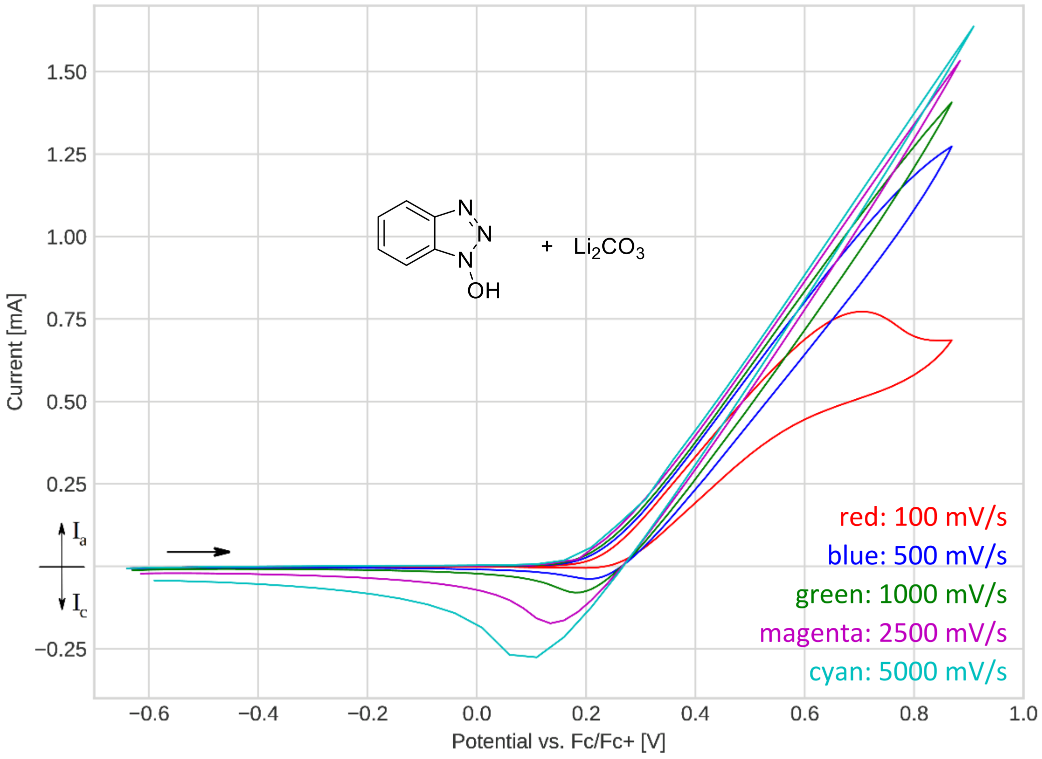

Supplementary Figure 31. Scans of varying scan rates for HOBt·H_2O **2a** (100 mM) with saturated Li_2CO_3 (MeOH/Pyridine 4:1).

NMR Spectra

The chemical shifts of the used solvent signals observed for ^{1}H NMR, ^{13}C NMR and ^{19}F NMR spectra are listed in the following chart. The multiplicity is shown as "*s*" for a singlet, "*d*" for a doublet, etc.

Solvent	^{1}H NMR (ppm)	^{13}C NMR (ppm)	^{19}F NMR (ppm)
**Chloroform-d**	7.26 (s)	77.2 (t)	–
**Dimethylsulfoxide-d_6**	2.5 (quint)	39.5 (sept)	–
**Methanol-d_4**	4.87 (s), 3.31(quint)	49.1 (sept)	–
**Hexafluorobenzene**	–	–	−163.0 (s)
**Trifluoromethoxybenzene**	–	–	−57.4 (s)

6-(Trifluoromethyl)-1*H*-benzo[*d*][1,2,3]triazol-1-ol (2b) [CAS: 26198-21-0]

^1H NMR (DMSO-d_6, 400 MHz)

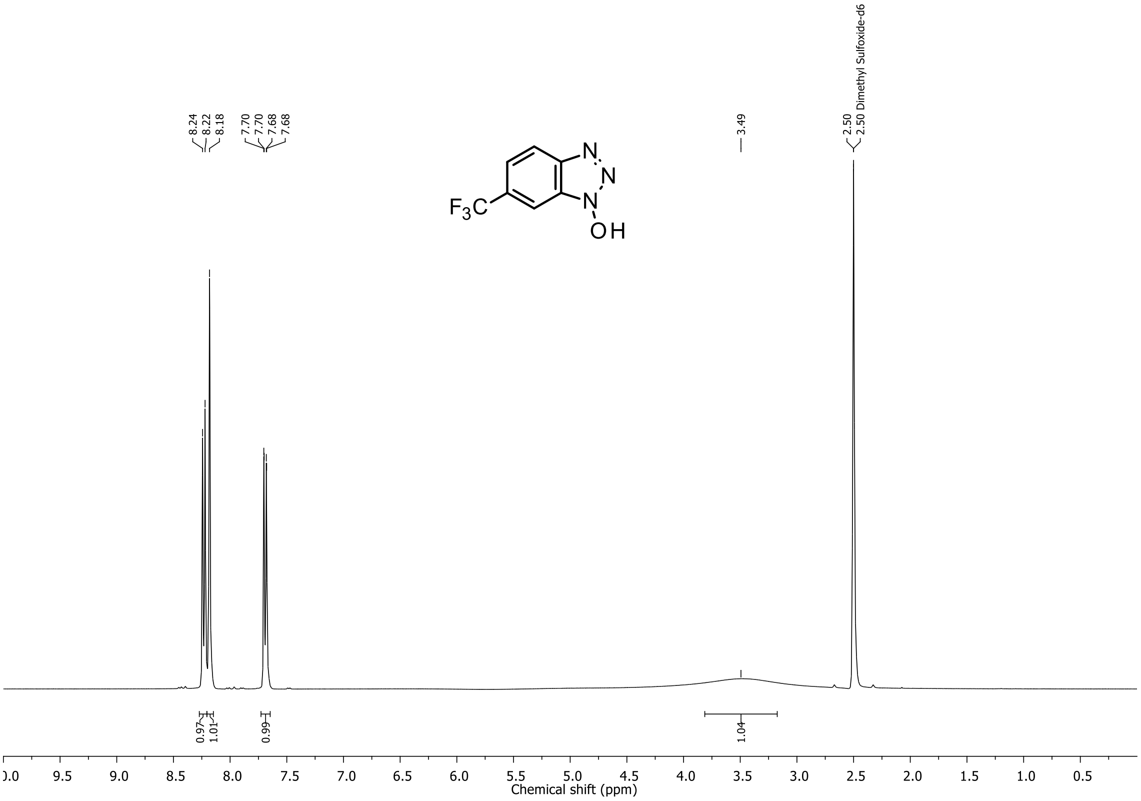

^{13}C NMR (DMSO-d_6, 101 MHz)

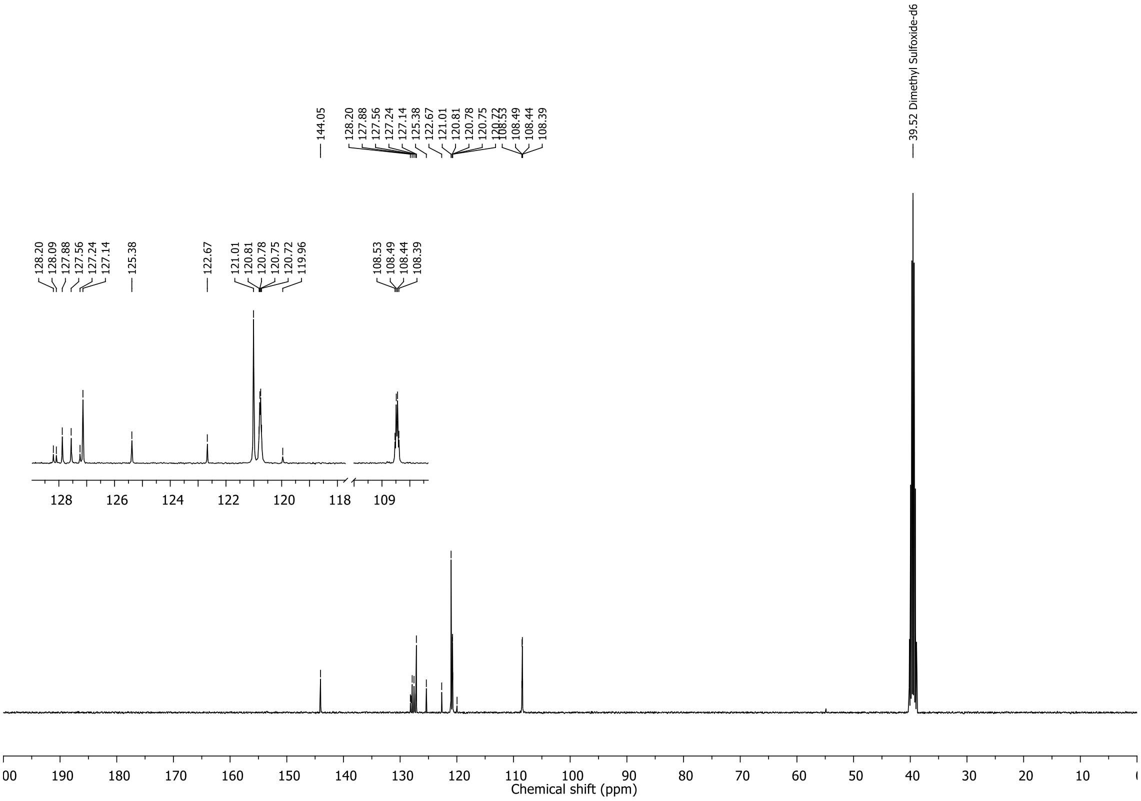

6-Nitro-1*H*-benzo[*d*][1,2,3]triazol-1-ol (2c) [CAS: 26185-63-7]

^{1}H NMR ($MeOD-d_4$, 300 MHz)

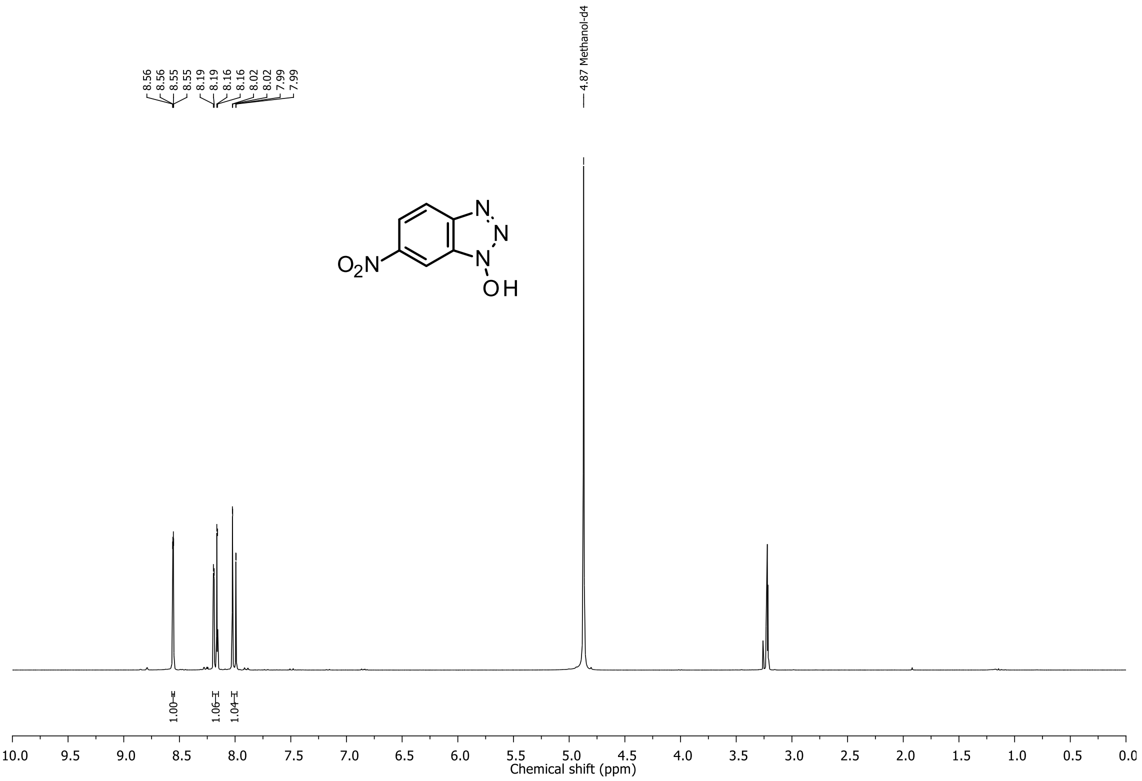

^{13}C NMR ($MeOD-d_4$, 75 MHz)

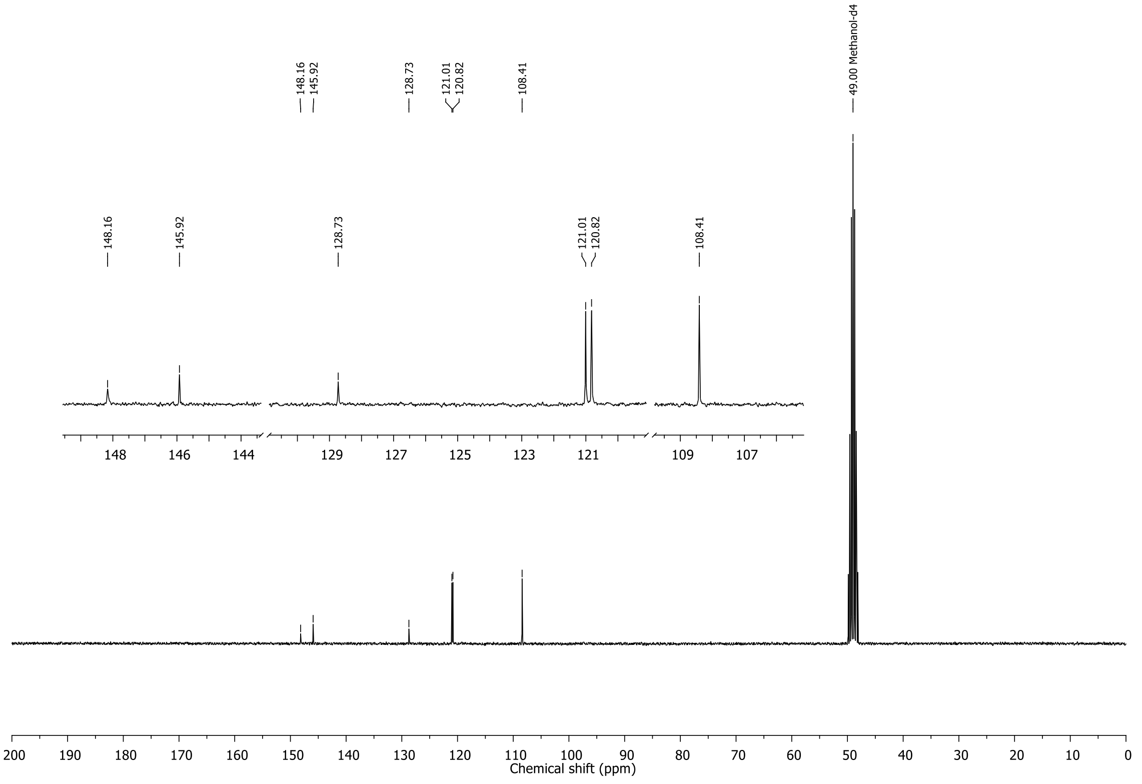

4-Chloro-6-(trifluoromethyl)-1*H*-benzo[*d*][1,2,3]triazol-1-ol (2d) [CAS: 2287307-42-8]

^{1}H NMR (MeOD-d$_4$, 300 MHz)

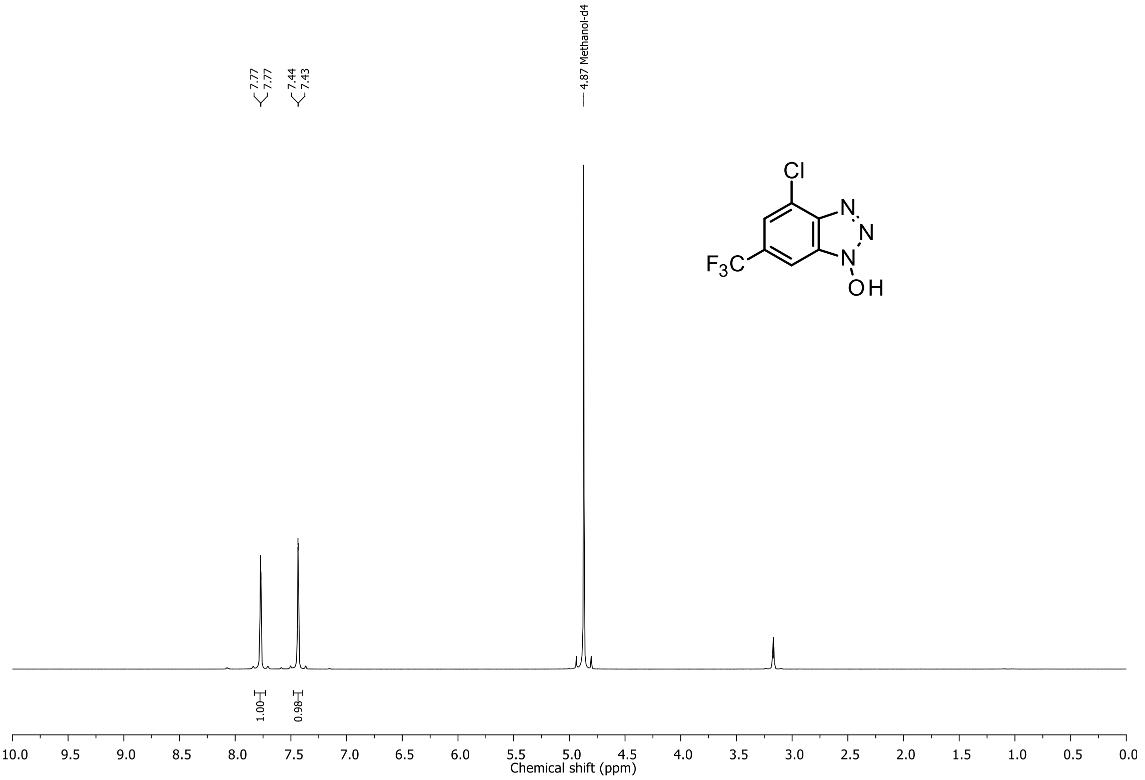

^{13}C NMR (MeOD-d$_4$, 75 MHz)

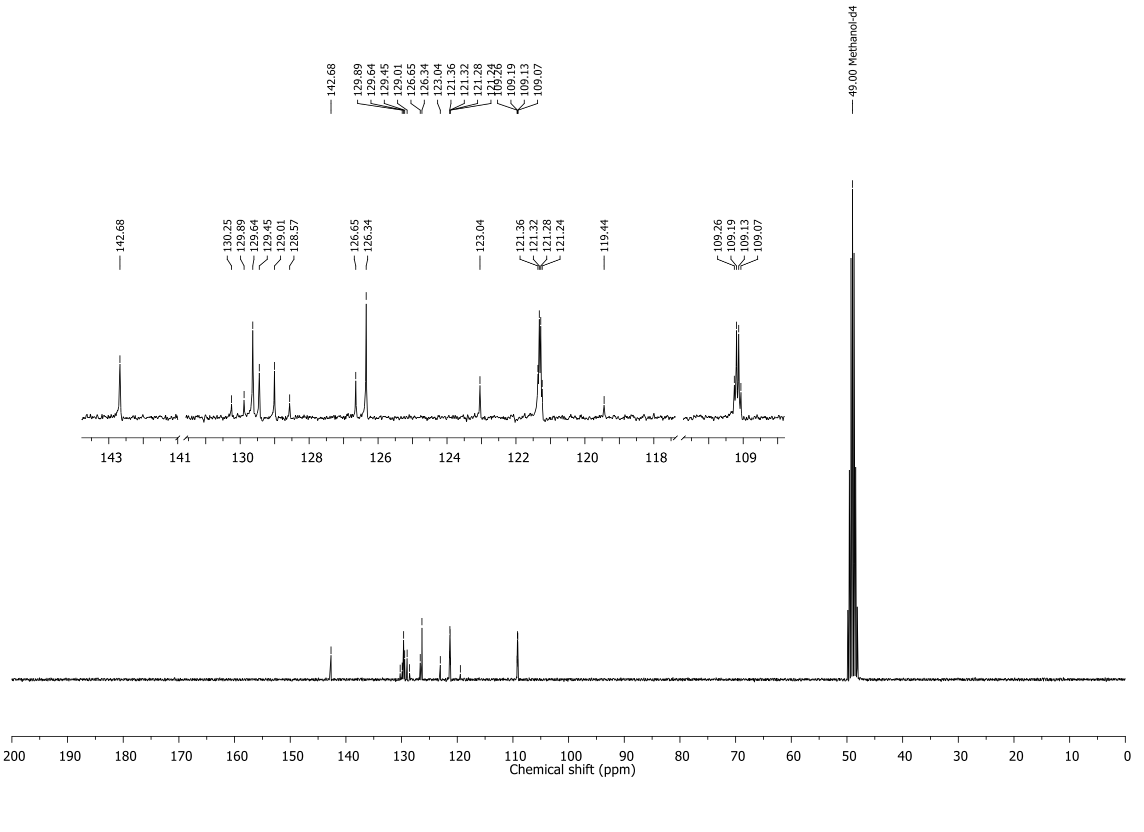

^{19}F NMR (MeOD-d_4, 235 MHz)

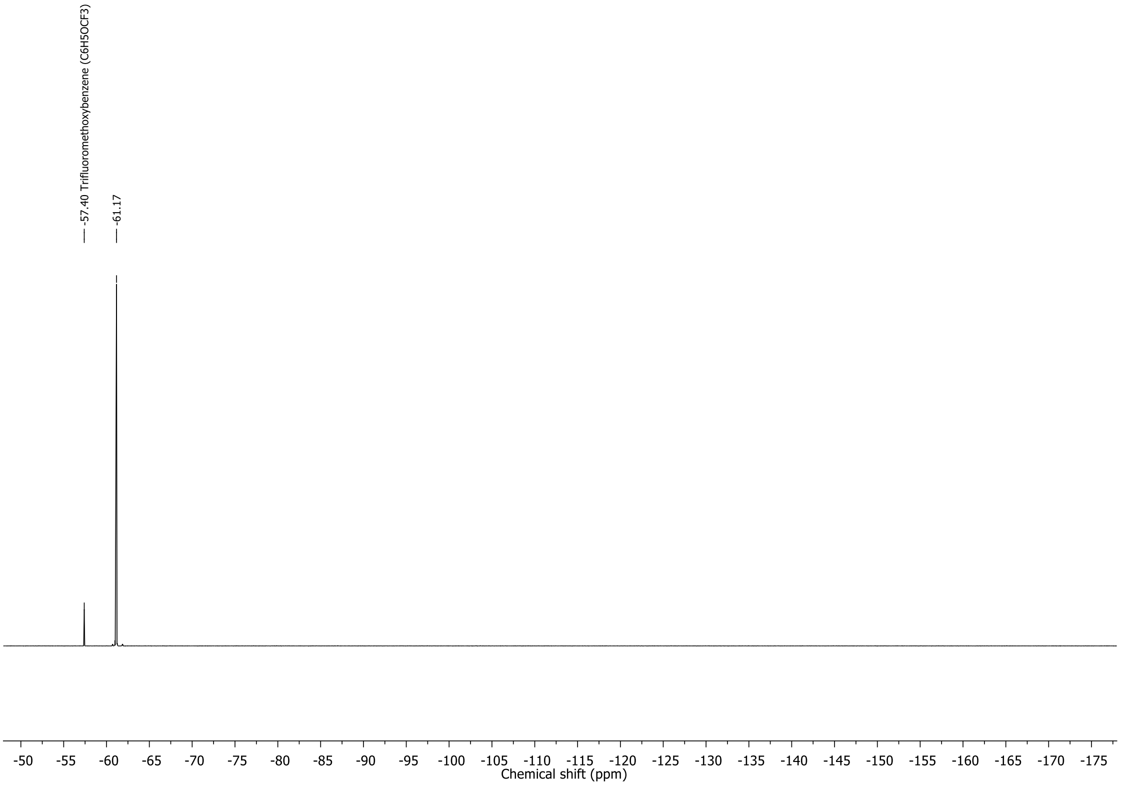

1-(2,2,2-Trifluoroethoxy)-1*H*-benzo[*d*][1,2,3]triazole (1ba) [CAS: 1855674-60-0]

^{1}H NMR ($CDCl_3$, 300 MHz)

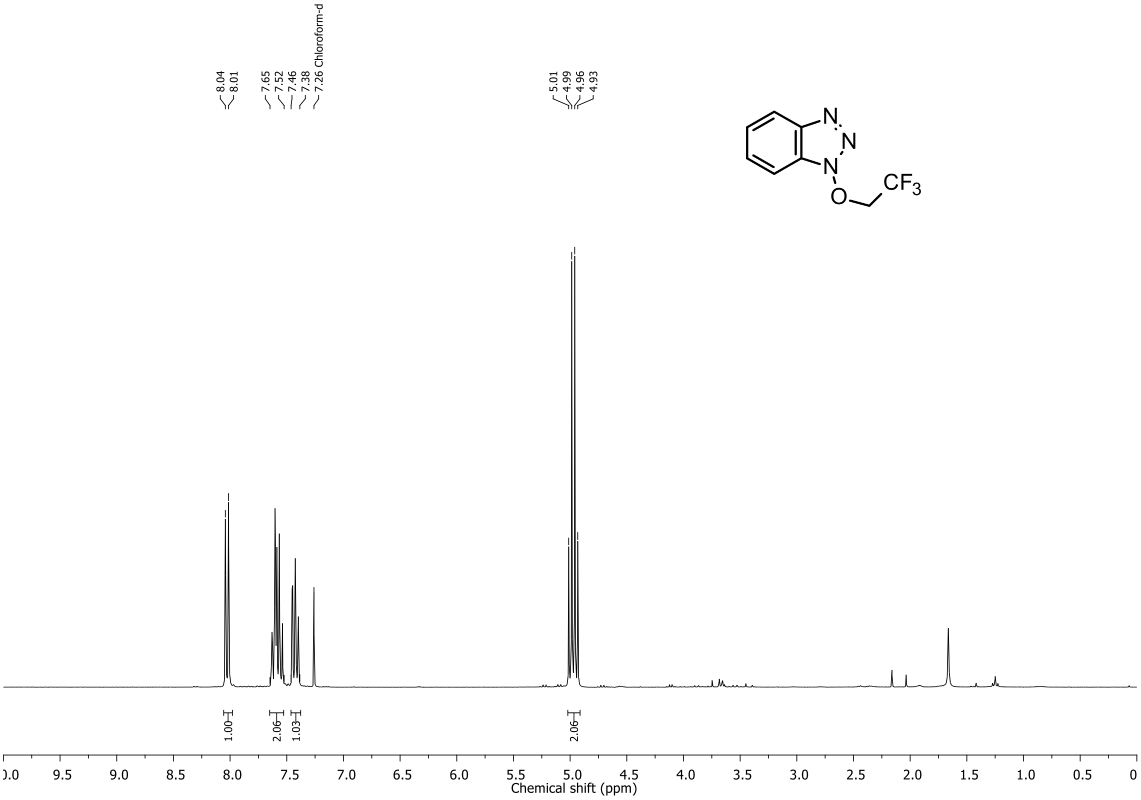

^{13}C NMR ($CDCl_3$, 101 MHz)

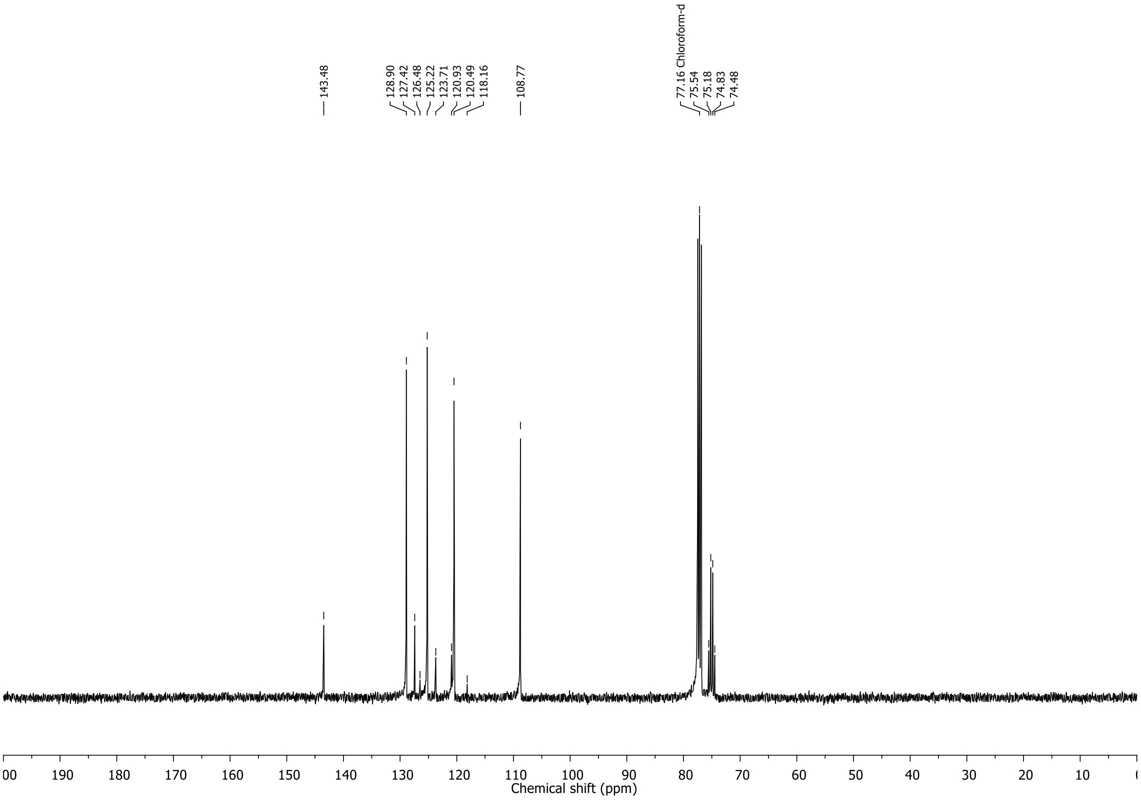

1-Phenethoxy-1*H*-benzo[*d*][1,2,3]triazole (1aa) [CAS: 1637647-71-2]

^{1}H NMR ($CDCl_3$, 300 MHz)

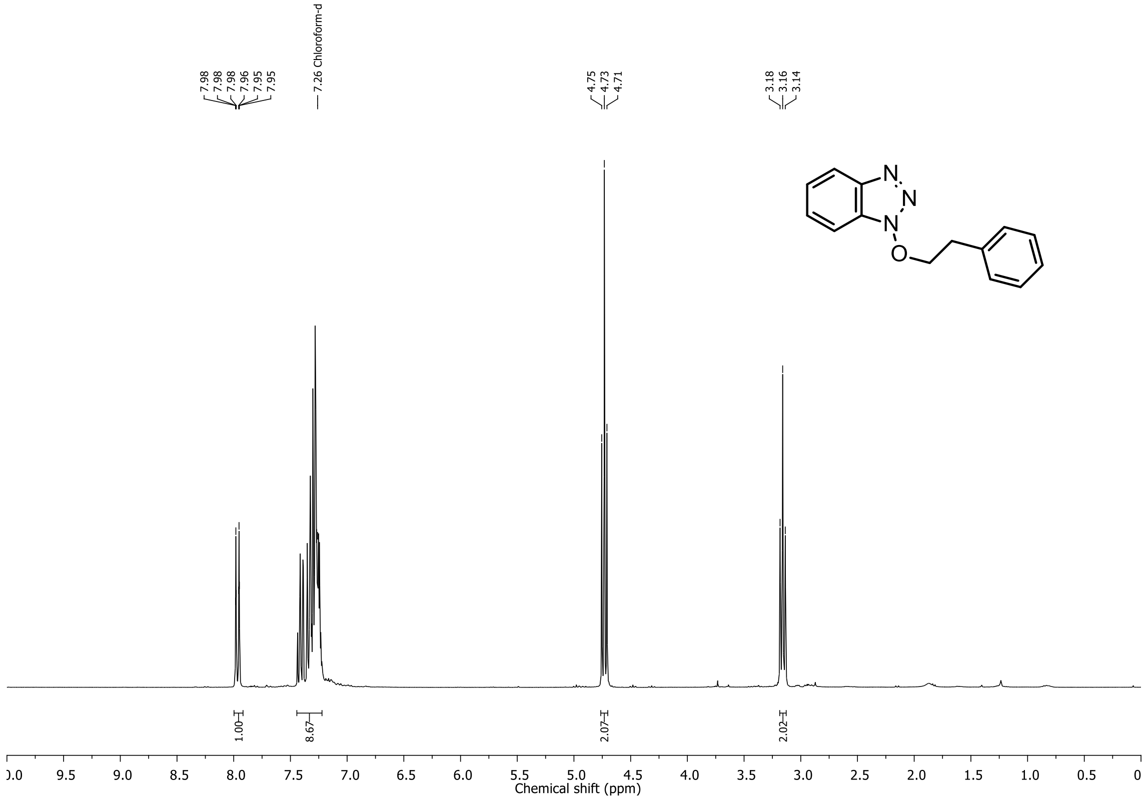

^{13}C NMR ($CDCl_3$, 63 MHz)

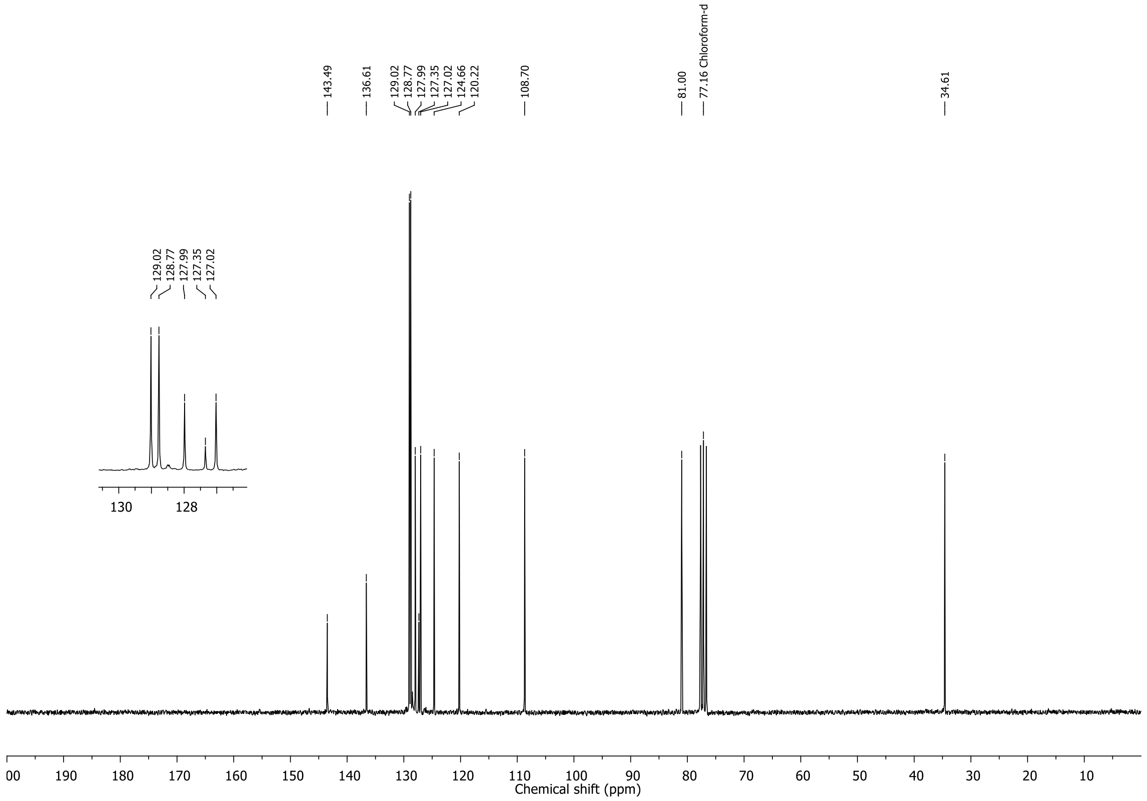

1-Methoxy-1*H*-benzo[*d*][1,2,3]triazole (1ca) [CAS: 22713-34-4]

^{1}H NMR ($CDCl_3$, 300 MHz)

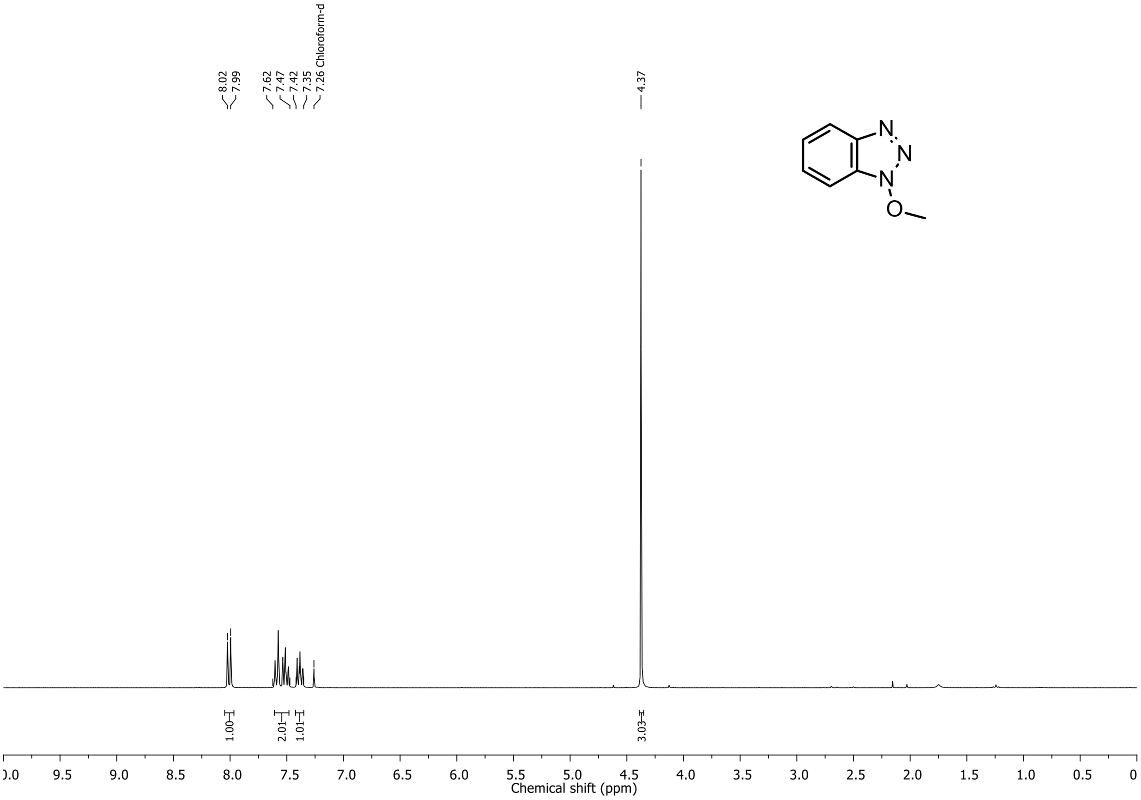

^{13}C NMR ($CDCl_3$, 75 MHz)

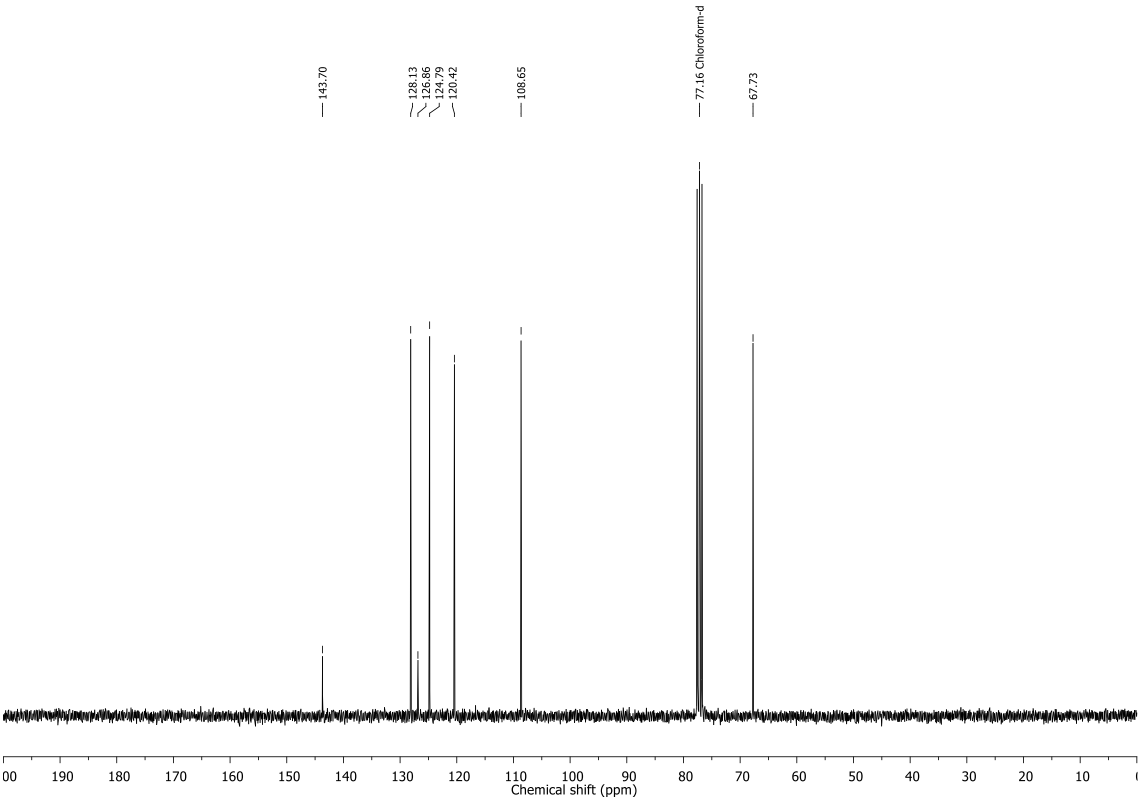

1-Ethoxy-1*H*-benzo[*d*][1,2,3]triazole (1da) [CAS: 57223-16-2]

^{1}H NMR ($CDCl_3$, 250 MHz)

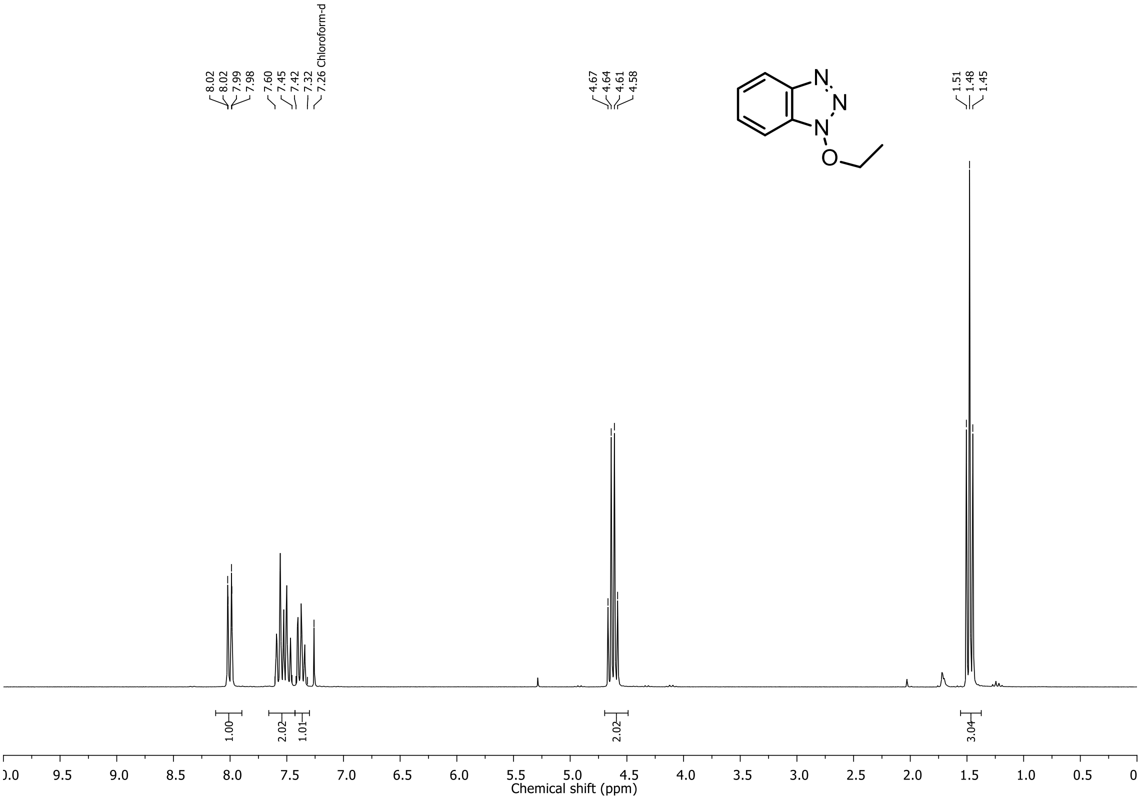

^{13}C NMR ($CDCl_3$, 63 MHz)

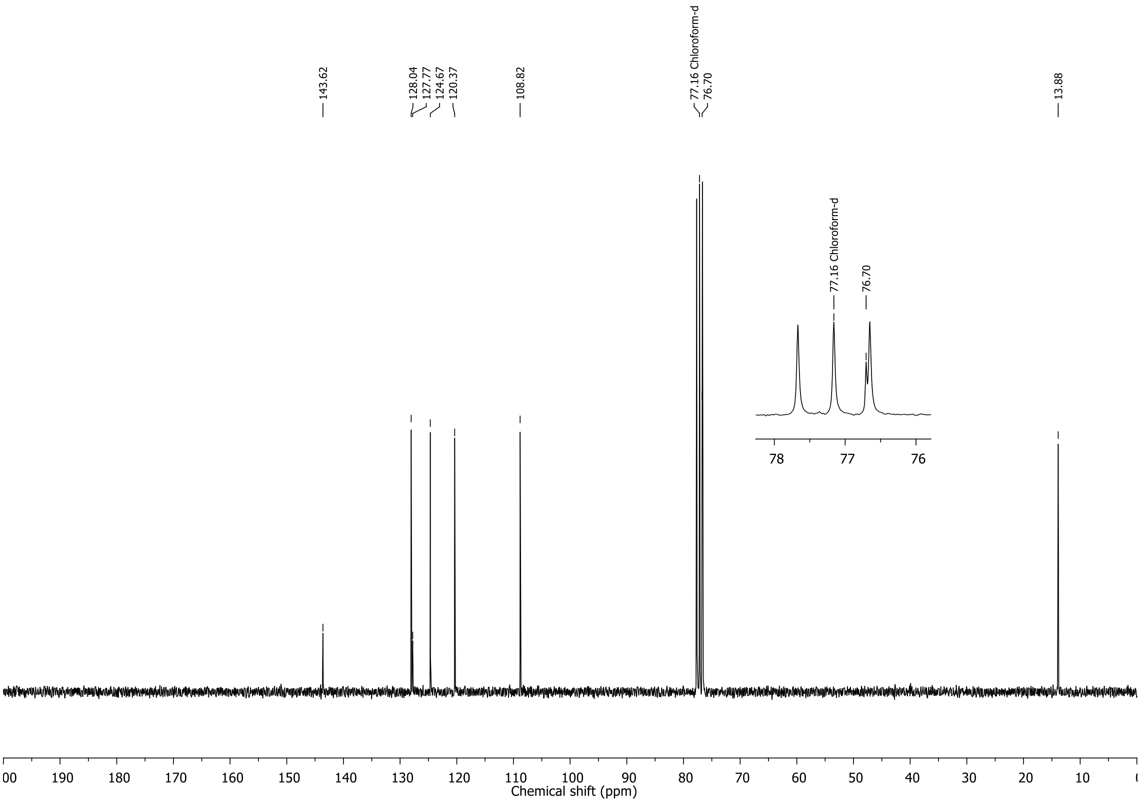

1-Propoxy-1*H*-benzo[*d*][1,2,3]triazole (1ea) [CAS: 60454-98-0]

^{1}H NMR (CDCl$_3$, 300 MHz)

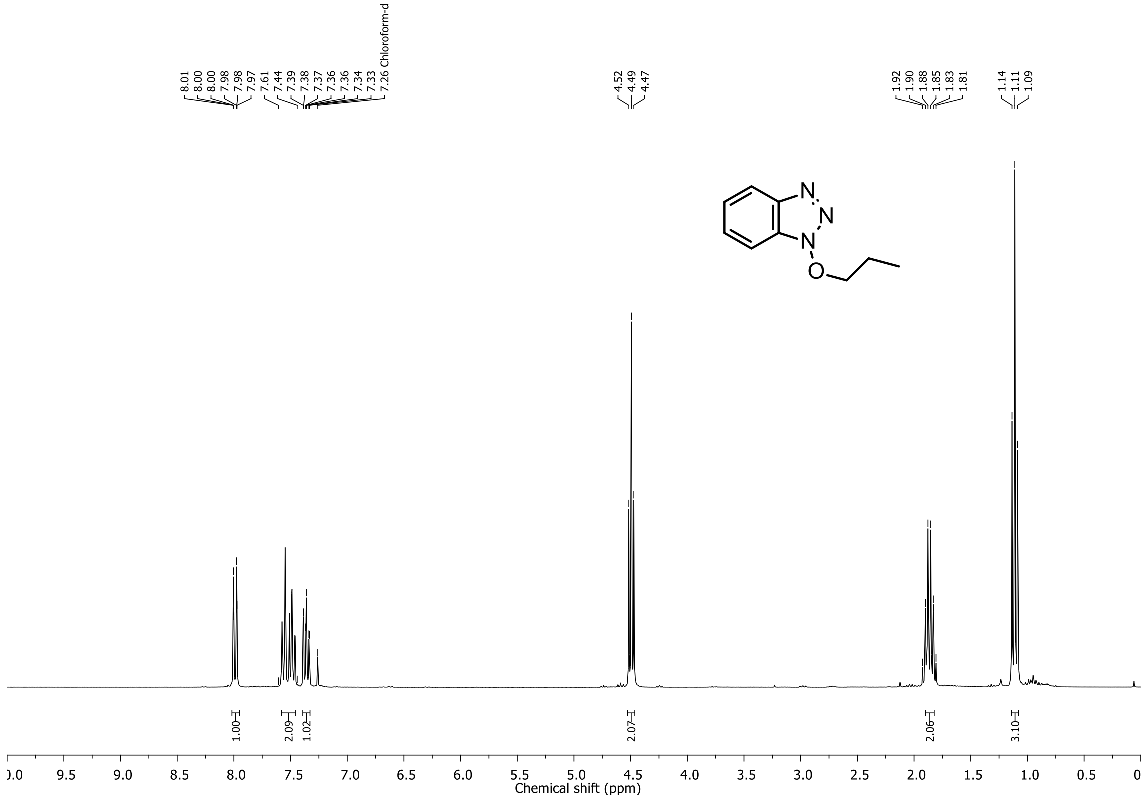

^{13}C NMR (CDCl$_3$, 75 MHz)

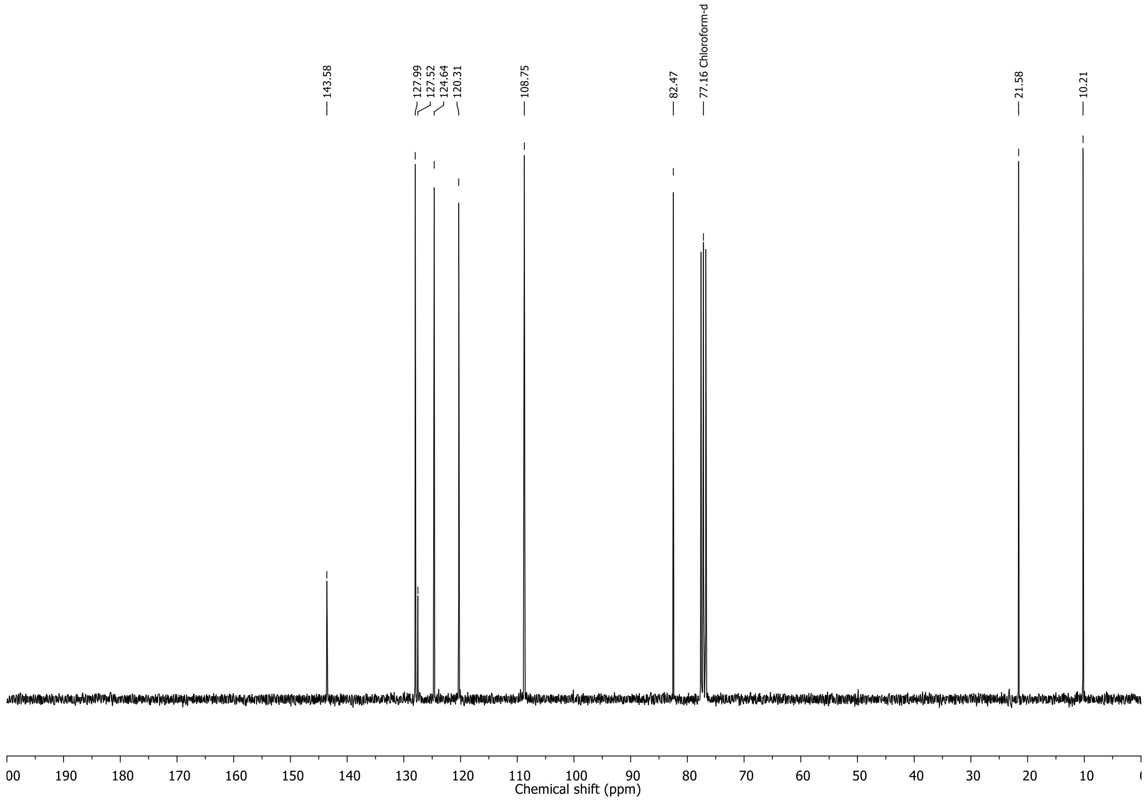

1-(Pentyloxy)-1*H*-benzo[*d*][1,2,3]triazole (1fa) [CAS: 60455-00-7]

^{1}H NMR ($CDCl_3$, 300 MHz)

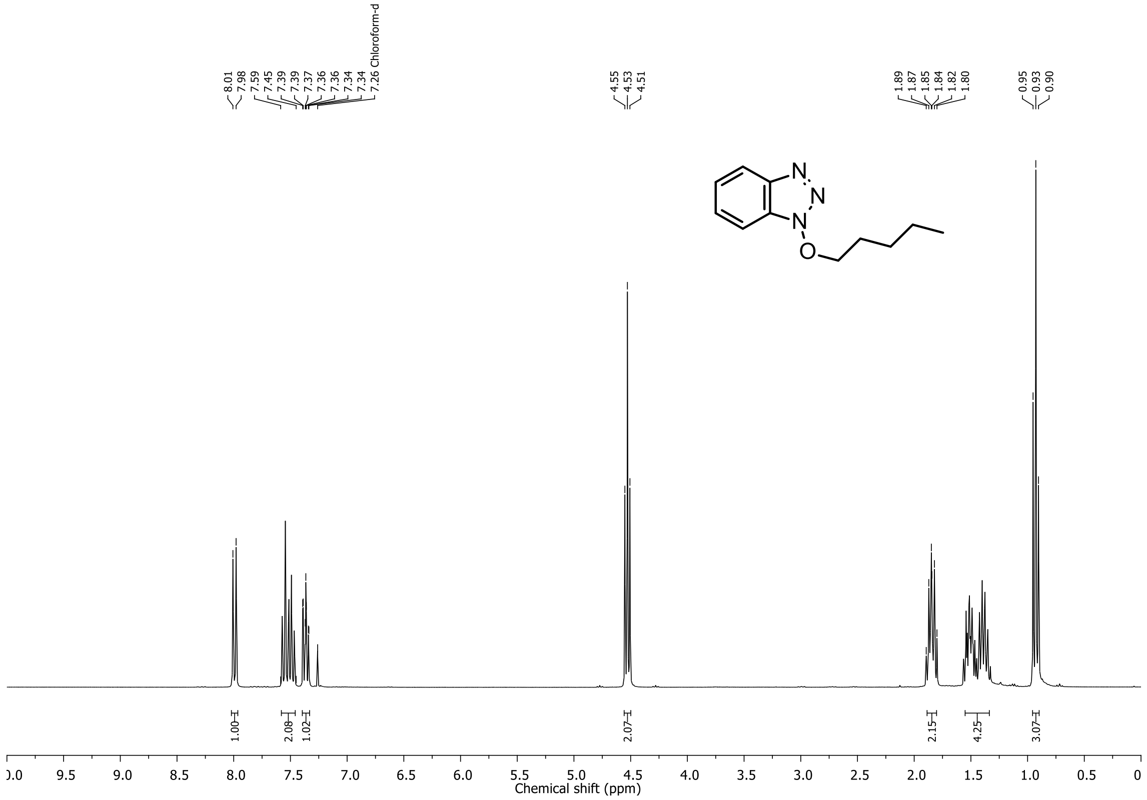

^{13}C NMR ($CDCl_3$, 75 MHz)

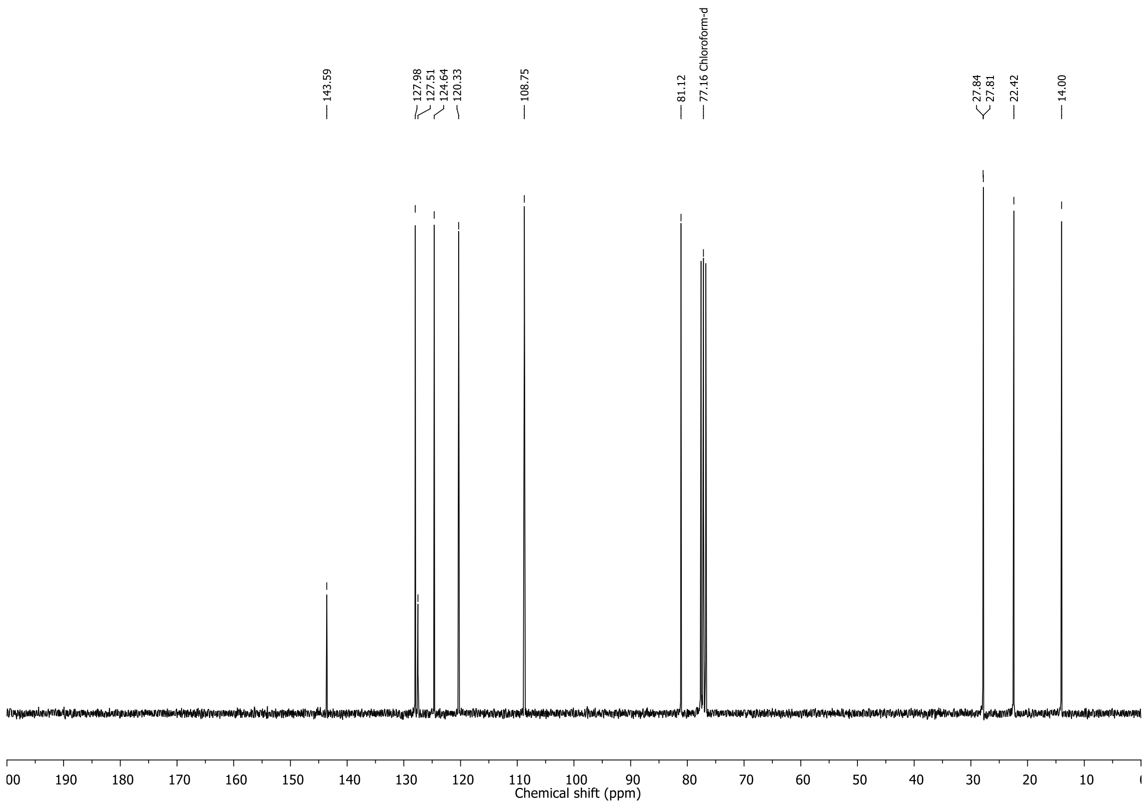

1-Isobutoxy-1*H*-benzo[*d*][1,2,3]triazole (1ga) [CAS: 1882571-07-4]

^{1}H NMR ($CDCl_3$, 300 MHz)

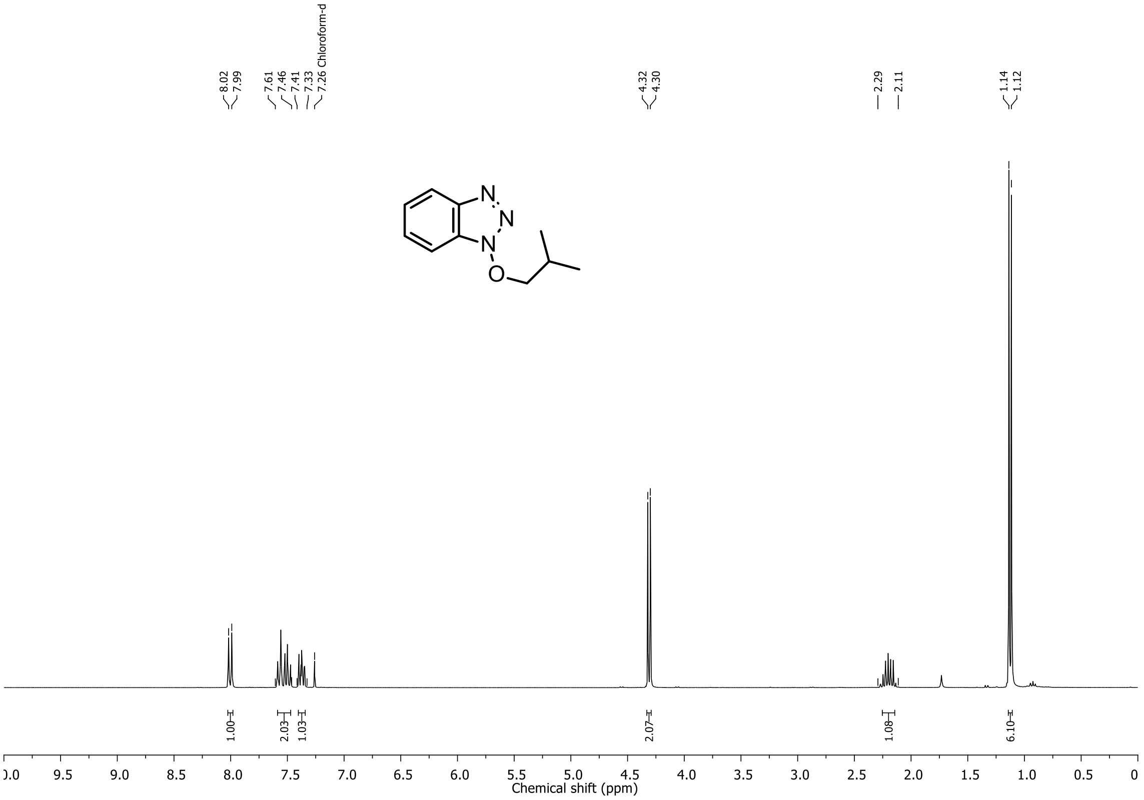

^{13}C NMR ($CDCl_3$, 75 MHz)

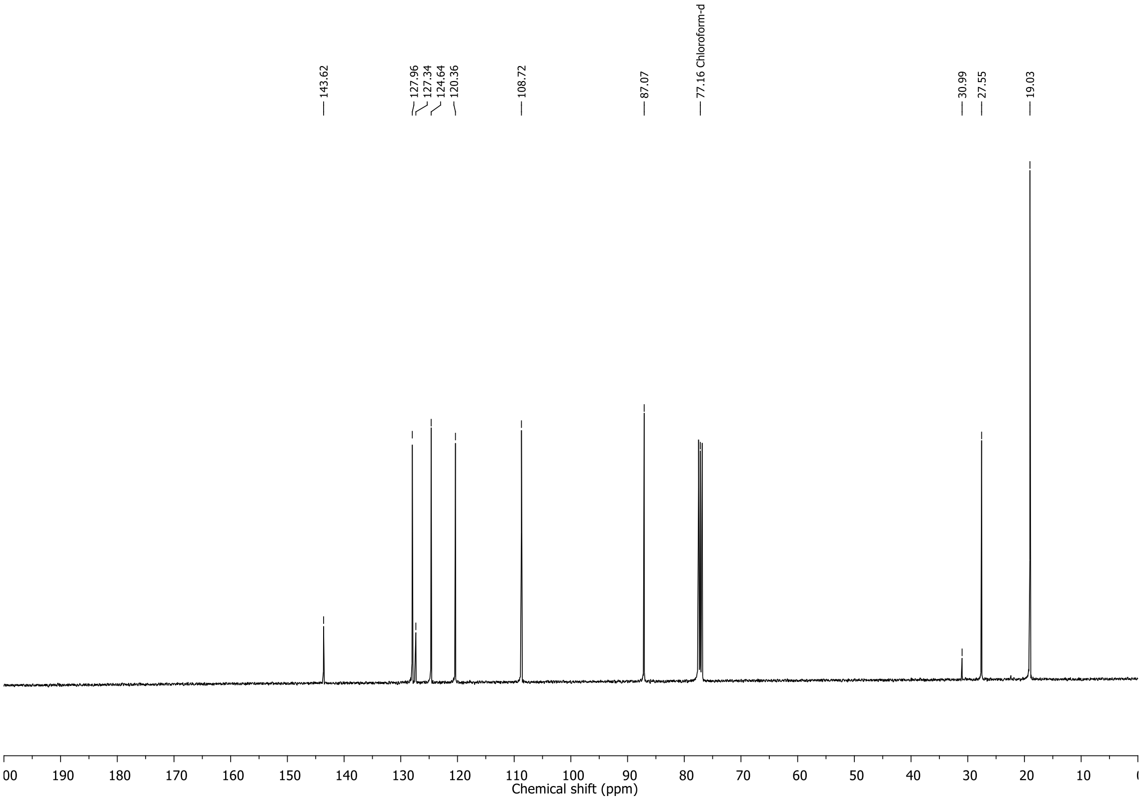

1-(Neopentyloxy)-1*H*-benzo[*d*][1,2,3]triazole (1ha) [CAS: 2160874-31-5]

^{1}H NMR ($CDCl_3$, 300 MHz)

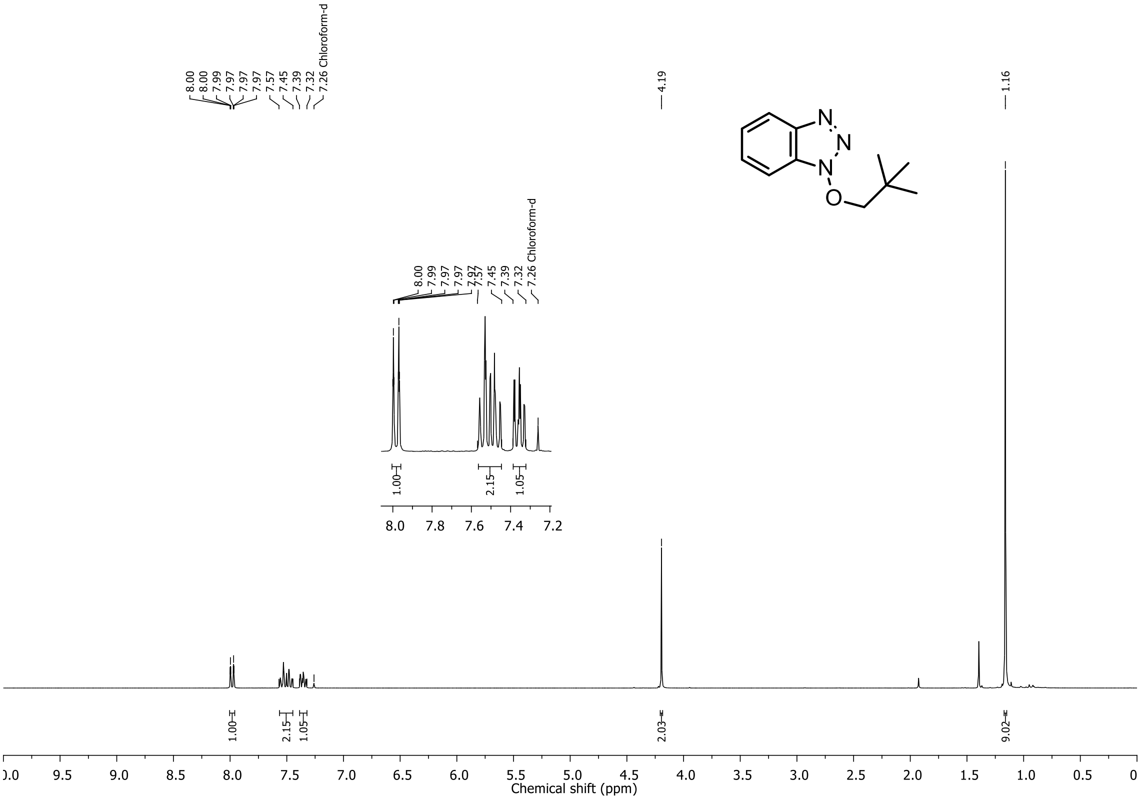

^{13}C NMR ($CDCl_3$, 75 MHz)

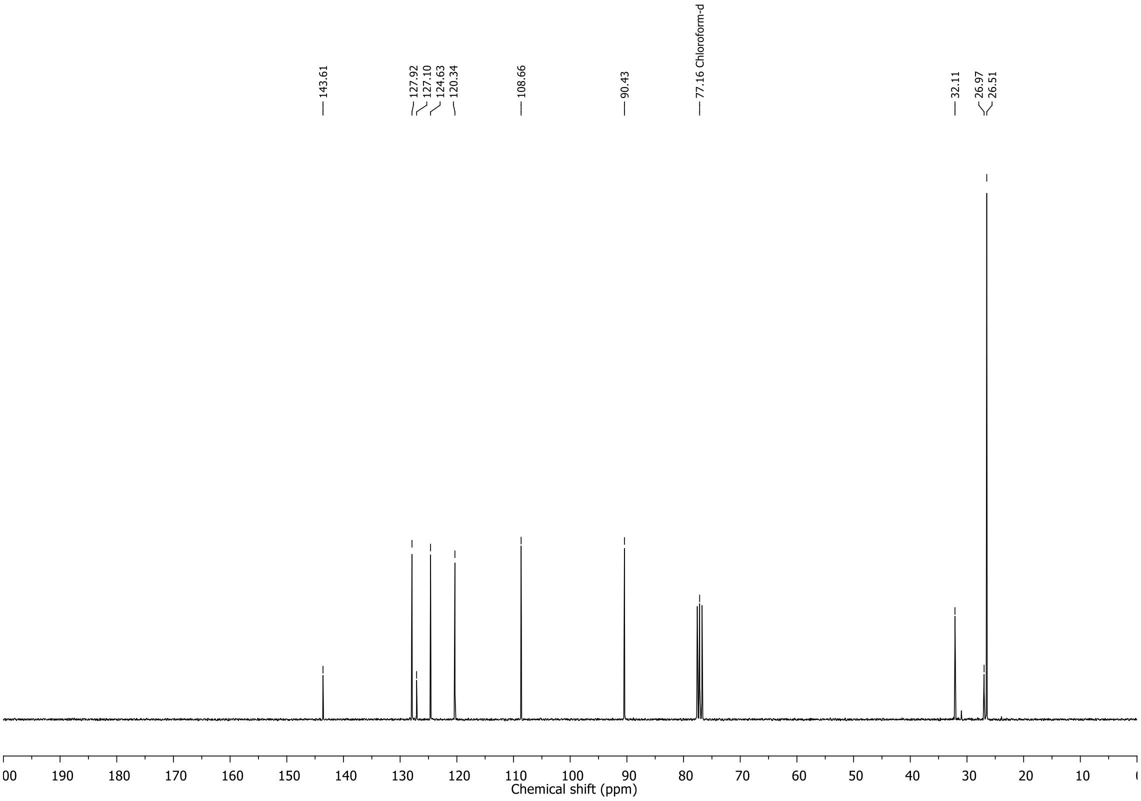

1-(Benzyloxy)-1*H*-benzo[*d*][1,2,3]triazole (1ia) [CAS: 68930-15-4]

^{1}H NMR ($CDCl_3$, 300 MHz)

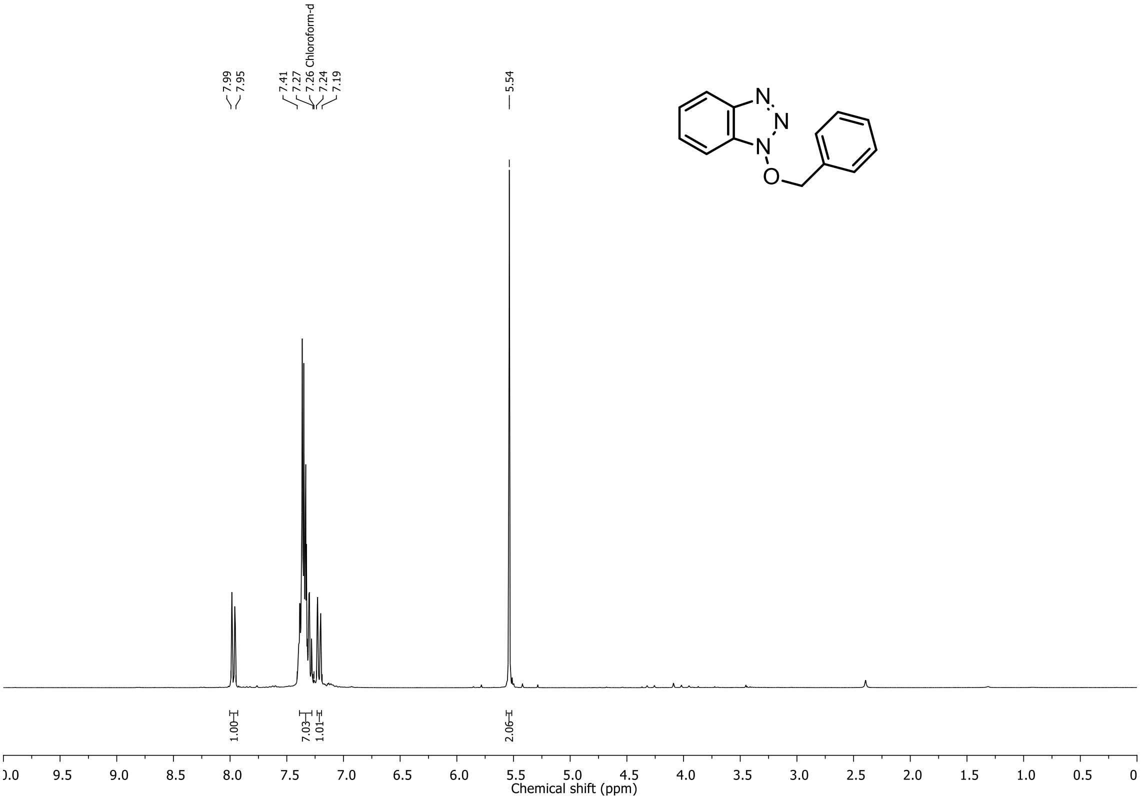

^{13}C NMR ($CDCl_3$, 75 MHz)

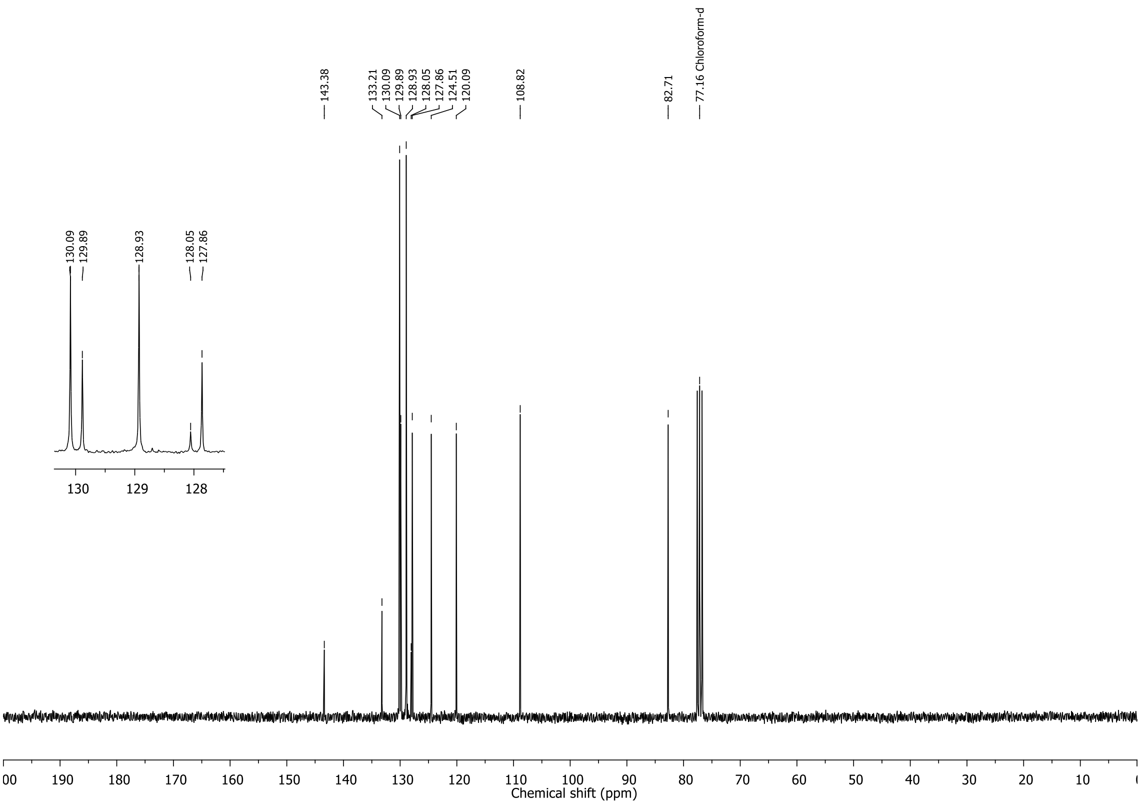

1-(2-Cyclohexylethoxy)-1*H*-benzo[*d*][1,2,3]triazole (1ja)

^{1}H NMR ($CDCl_3$, 300 MHz)

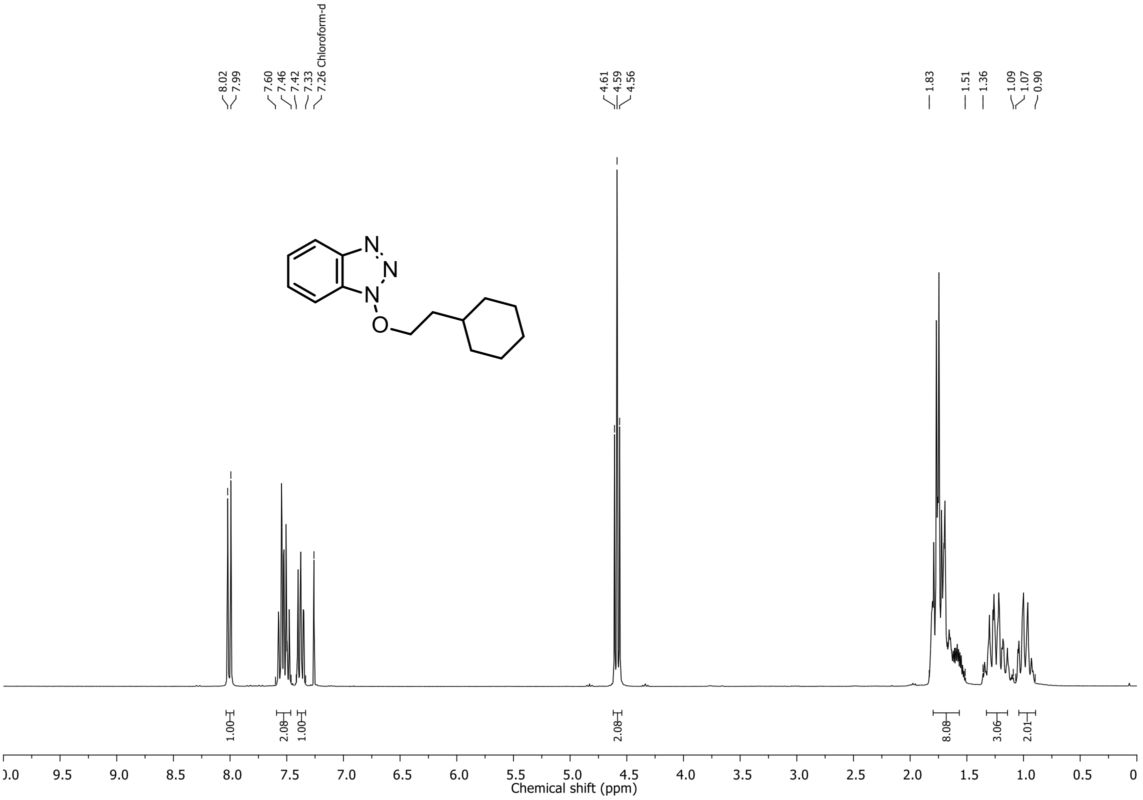

^{13}C NMR ($CDCl_3$, 75 MHz)

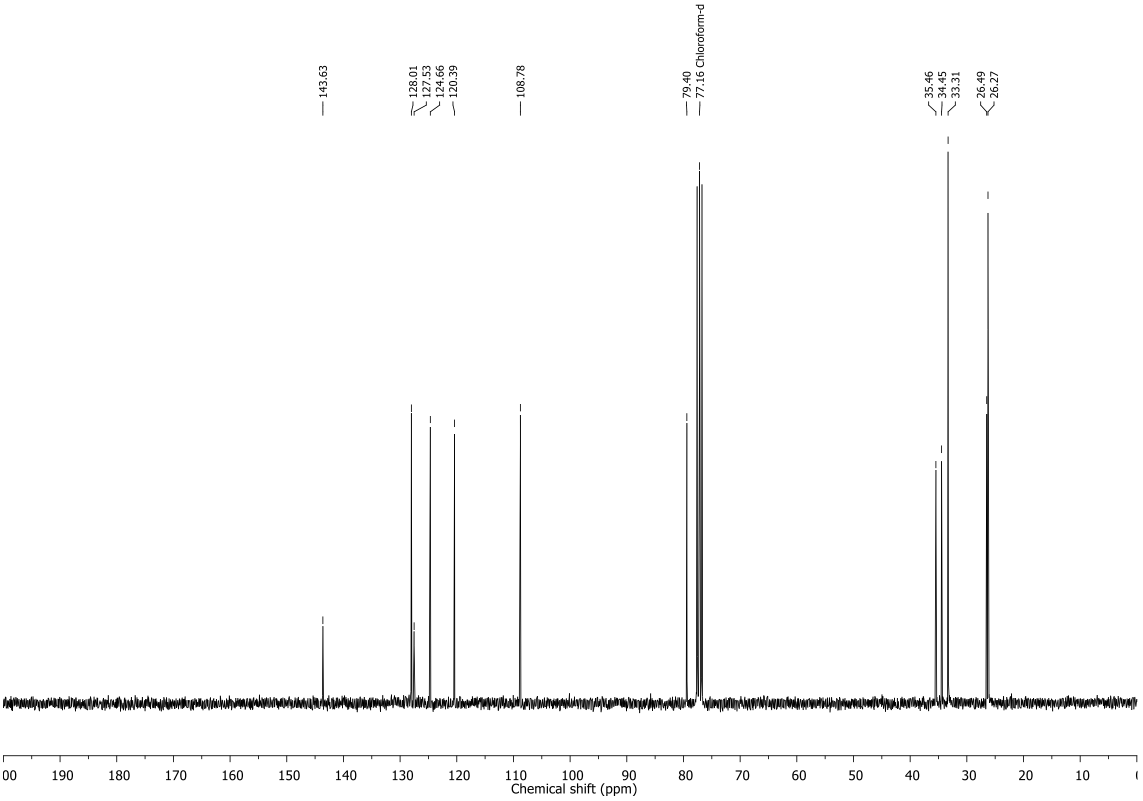

1-(2-Chloroethoxy)-1*H*-benzo[*d*][1,2,3]triazole (1ka) [CAS: 213249-23-1]

^{1}H NMR ($CDCl_3$, 300 MHz)

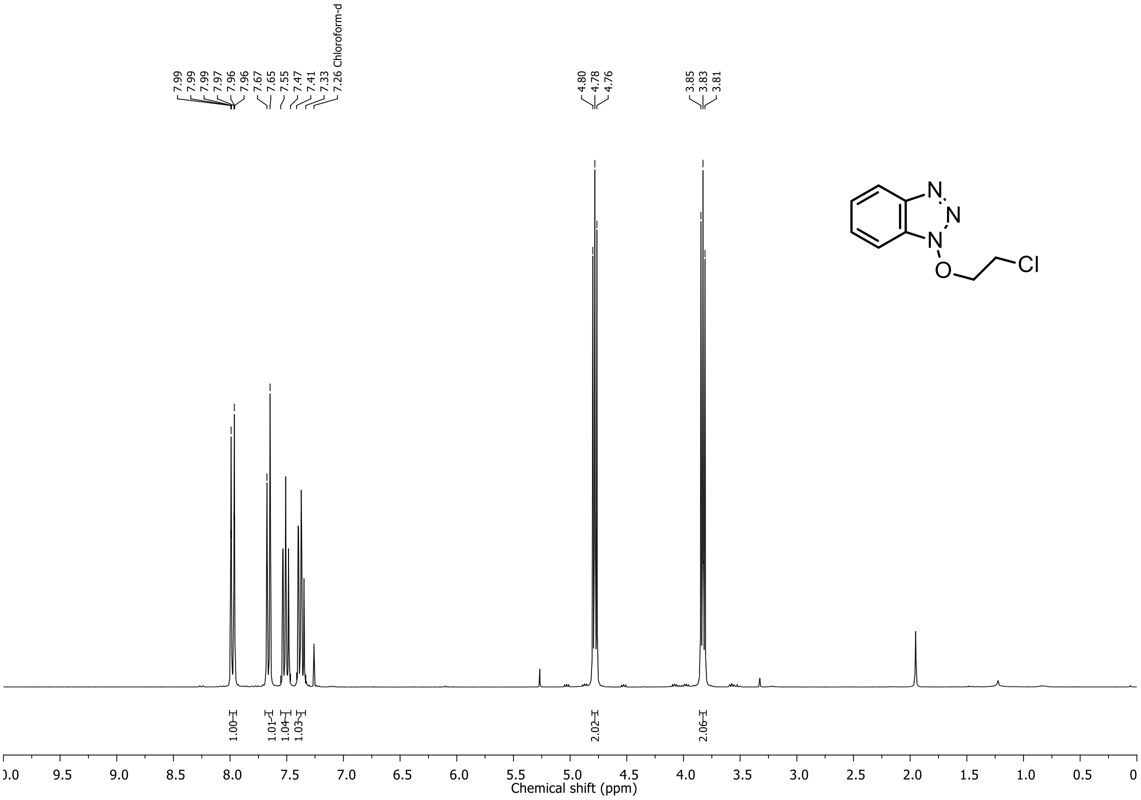

^{13}C NMR ($CDCl_3$, 75 MHz)

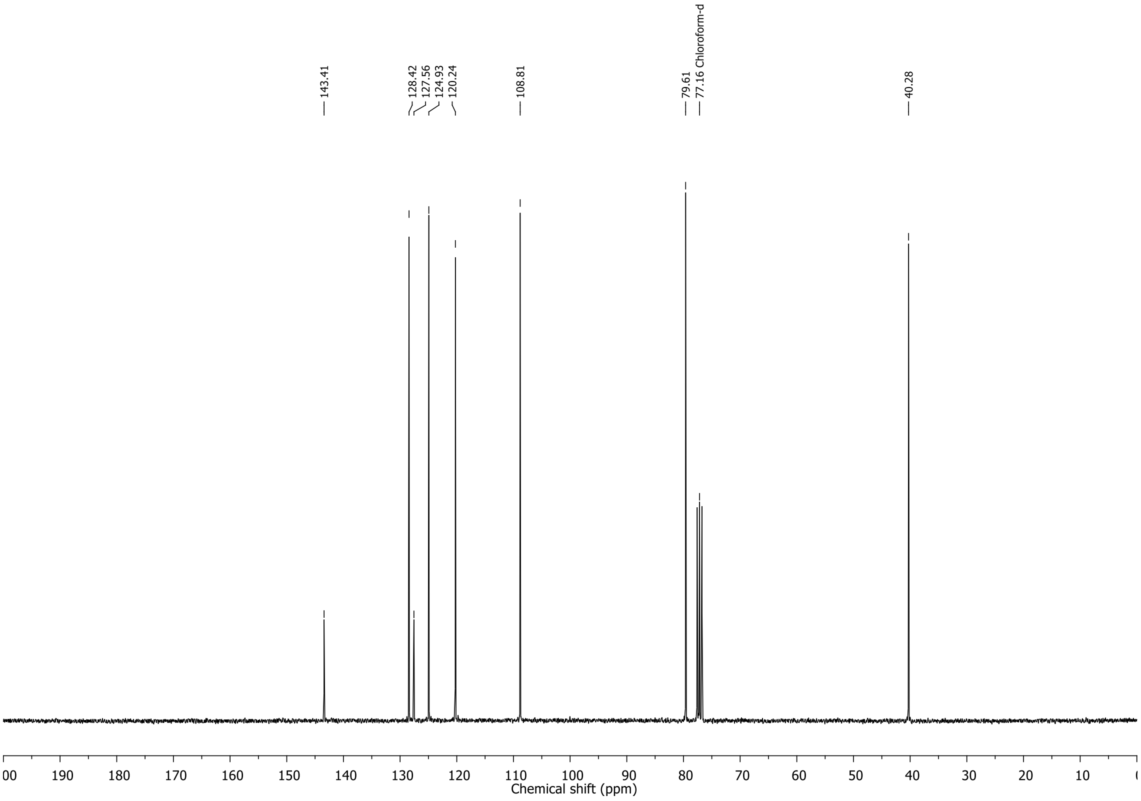

6-((1*H*-Benzo[*d*][1,2,3]triazol-1-yl)oxy)hexanenitrile (1la)

^{1}H NMR ($CDCl_3$, 300 MHz)

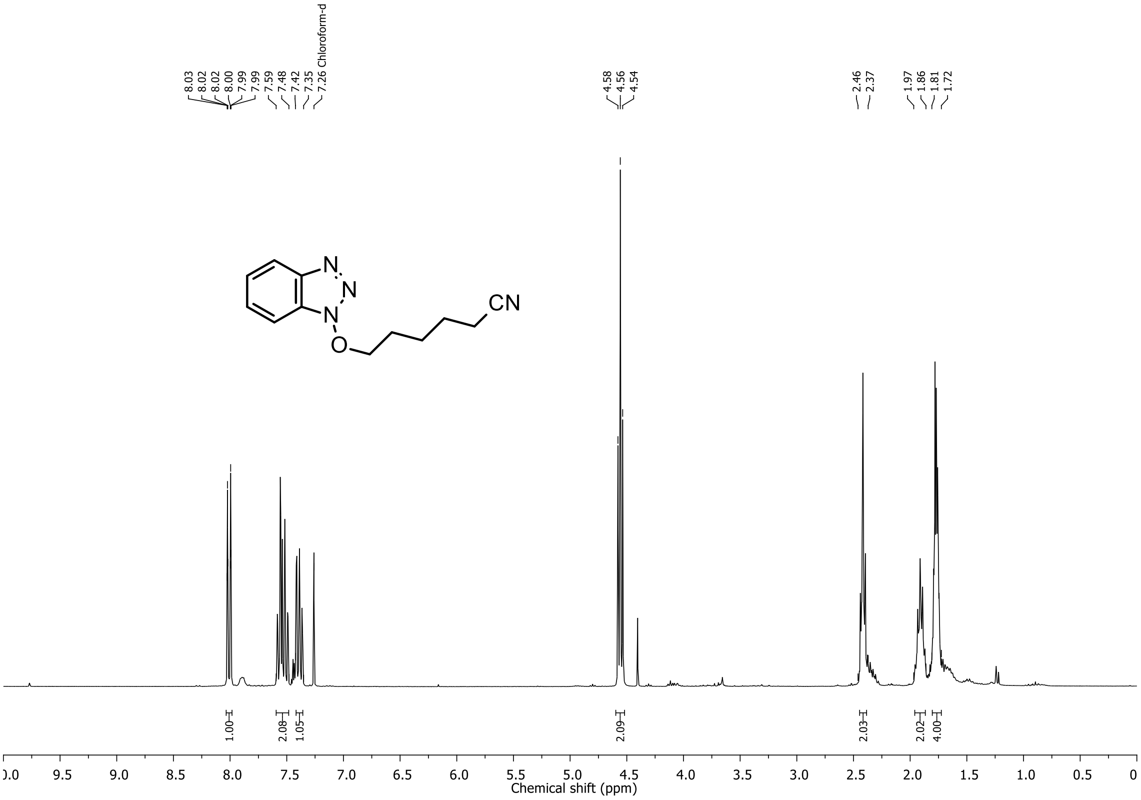

^{13}C NMR ($CDCl_3$, 75 MHz)

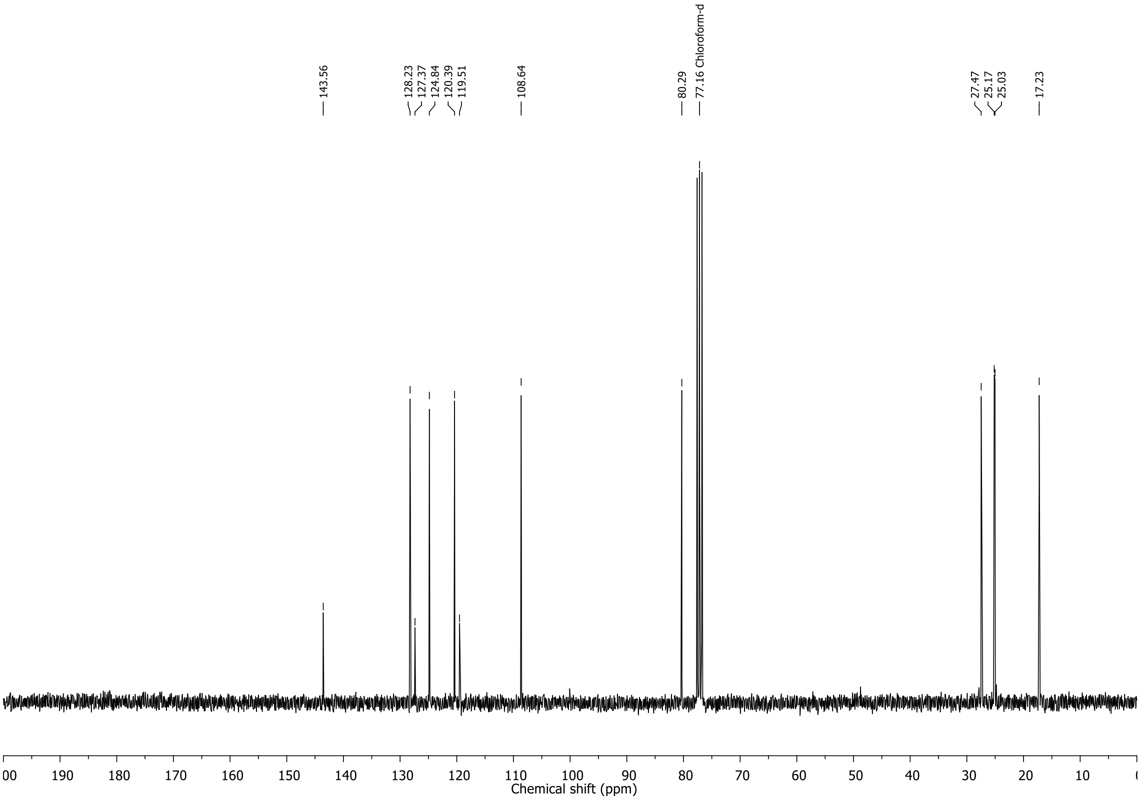

1-((5-Azidopentyl)oxy)-1*H*-benzo[*d*][1,2,3]triazole (1ma)

^{1}H NMR ($CDCl_3$, 300 MHz)

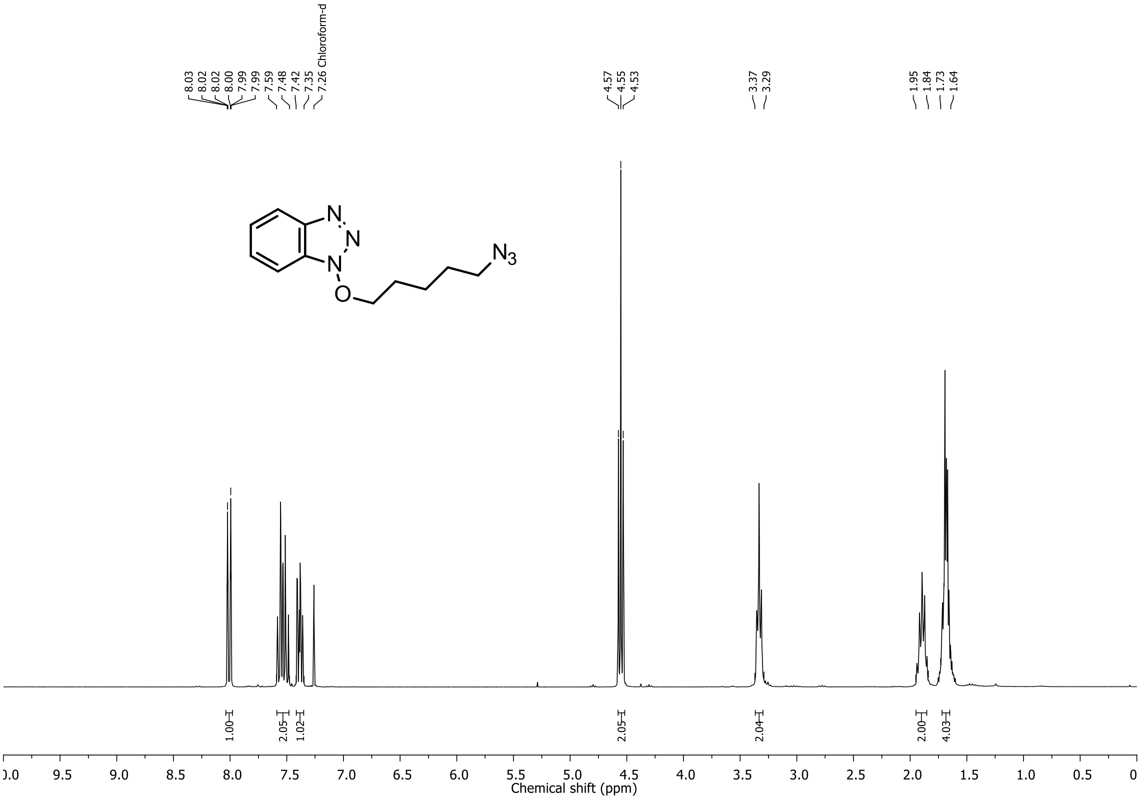

^{13}C NMR ($CDCl_3$, 75 MHz)

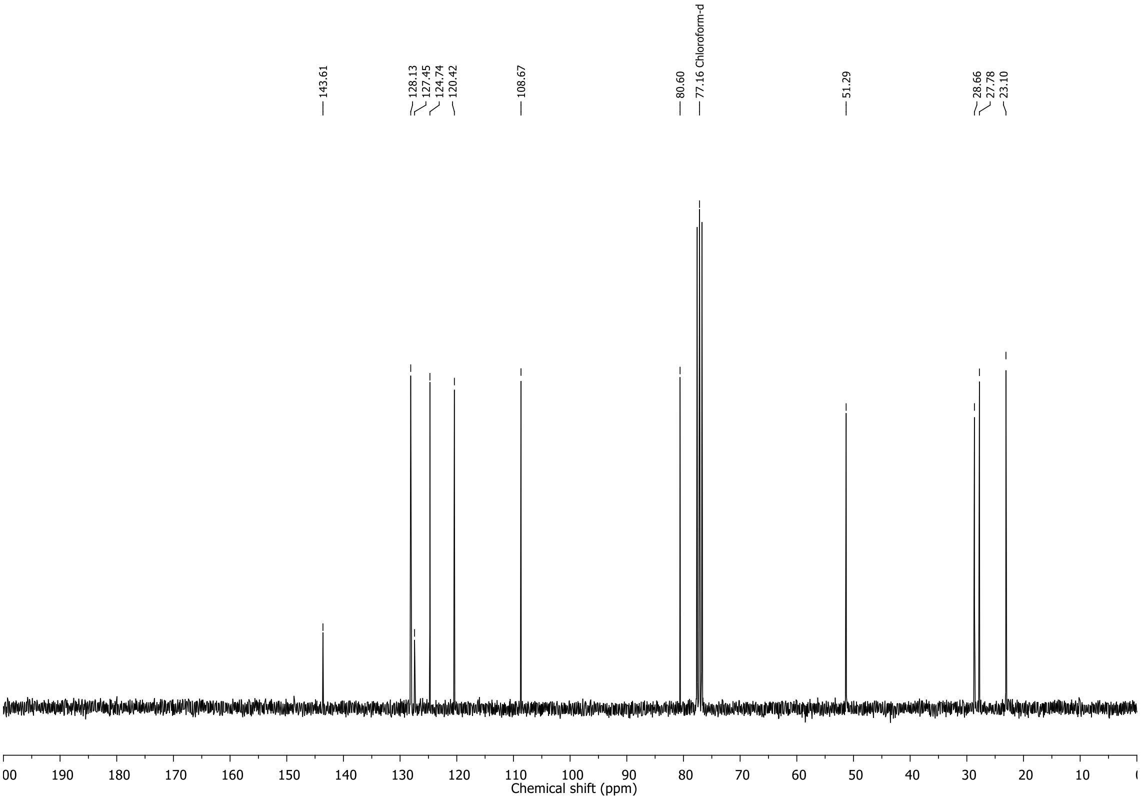

1-(But-3-en-1-yloxy)-1*H*-benzo[*d*][1,2,3]triazole (1na) [CAS: 77204-11-6]

^{1}H NMR ($CDCl_3$, 300 MHz)

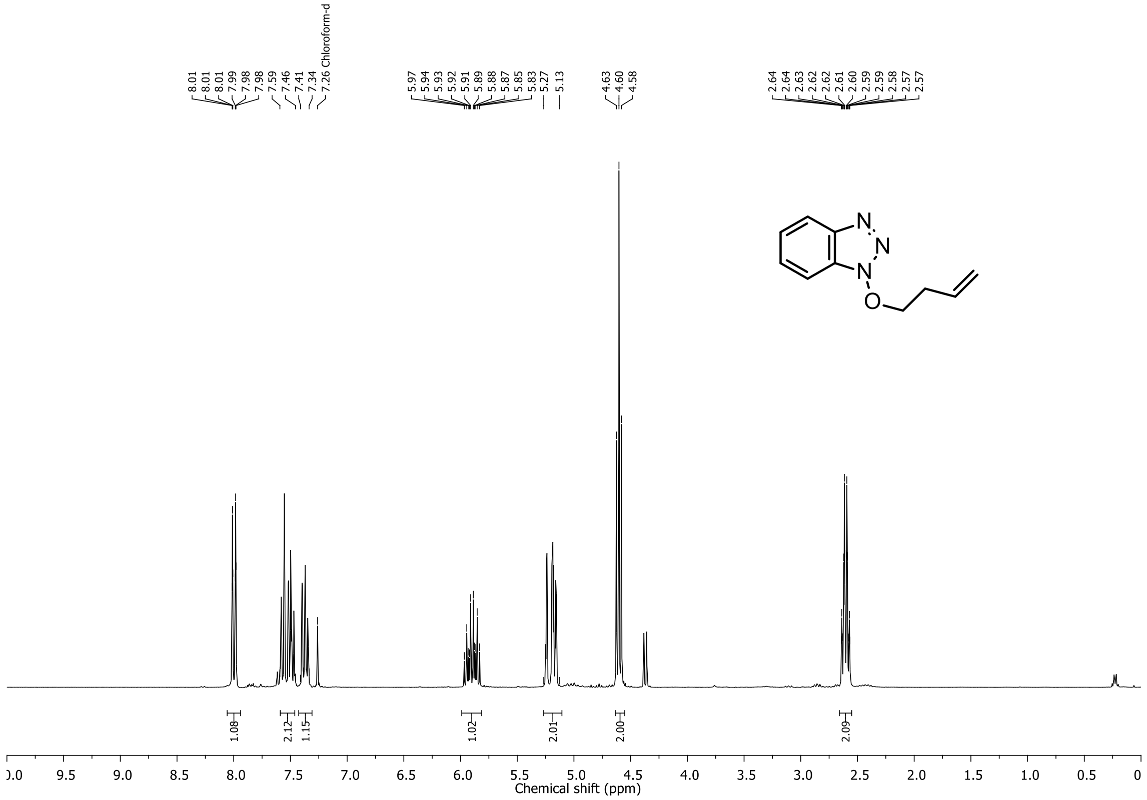

^{13}C NMR ($CDCl_3$, 101 MHz)

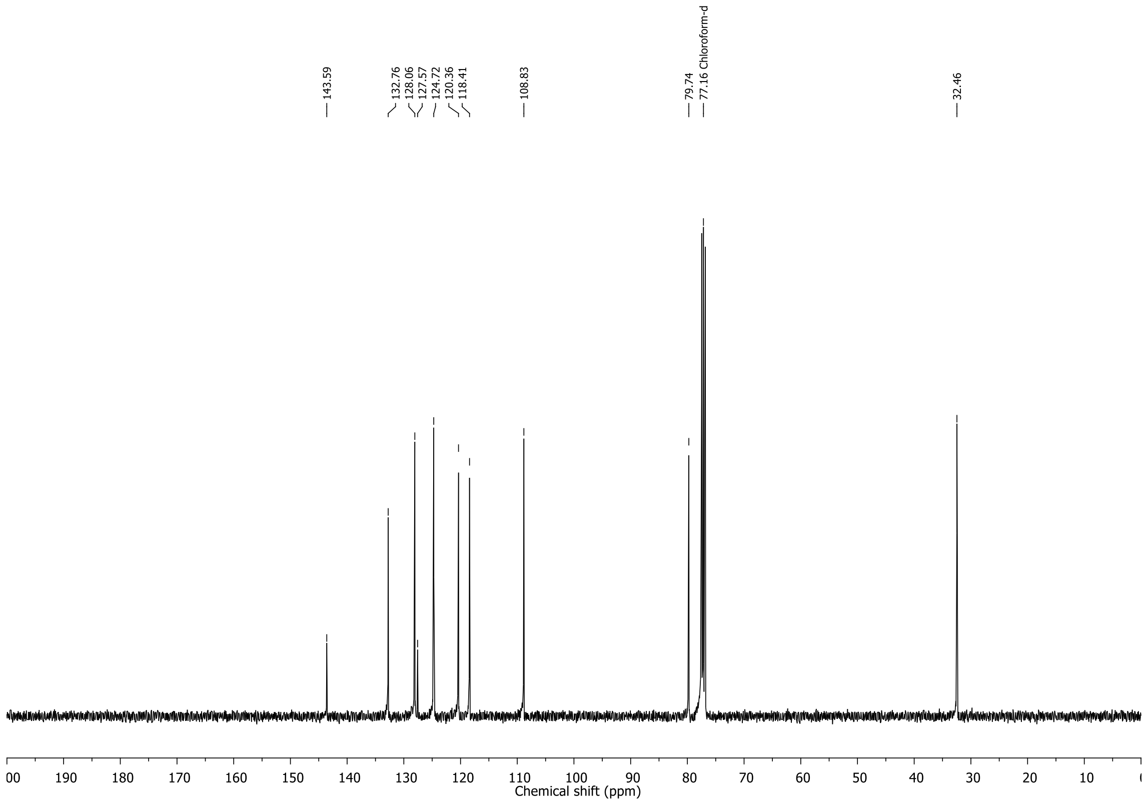

1-(Hex-5-en-1-yloxy)-1*H*-benzo[*d*][1,2,3]triazole (1oa)

^{1}H NMR ($CDCl_3$, 300 MHz)

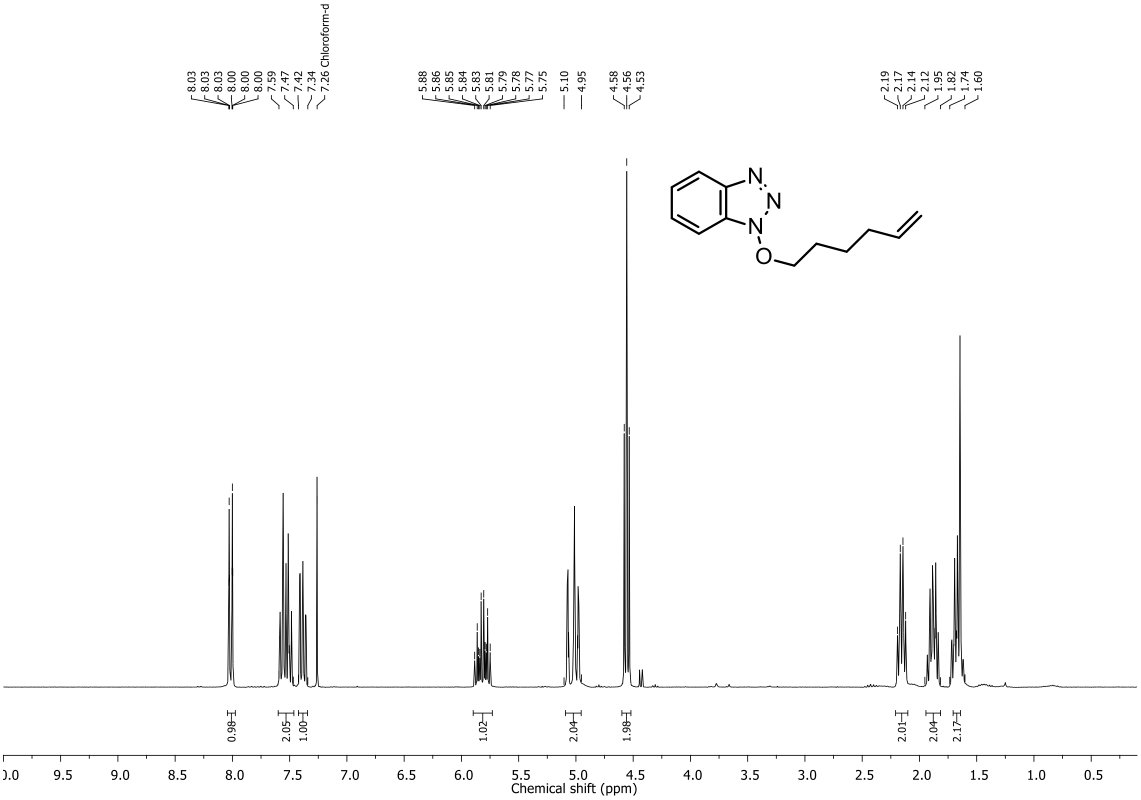

^{13}C NMR ($CDCl_3$, 75 MHz)

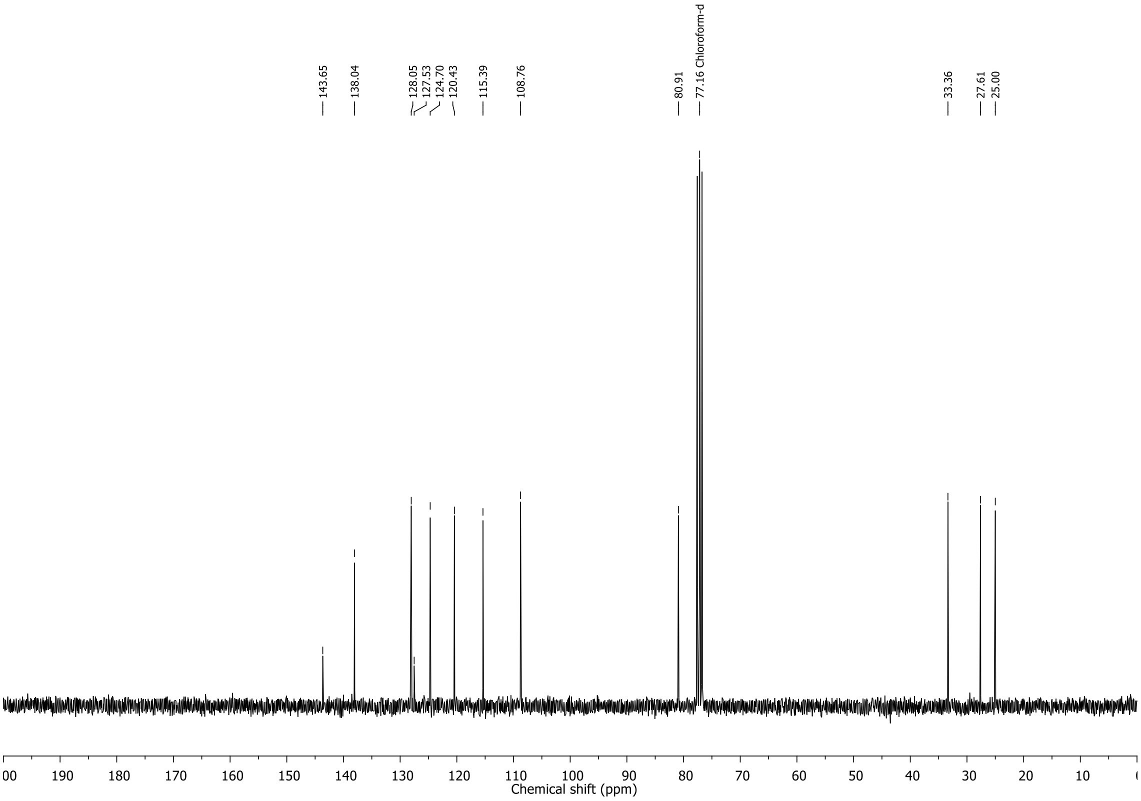

1-Propoxy-1*H*-benzo[*d*][1,2,3]triazole (3pa)

^{1}H NMR ($CDCl_3$, 300 MHz)

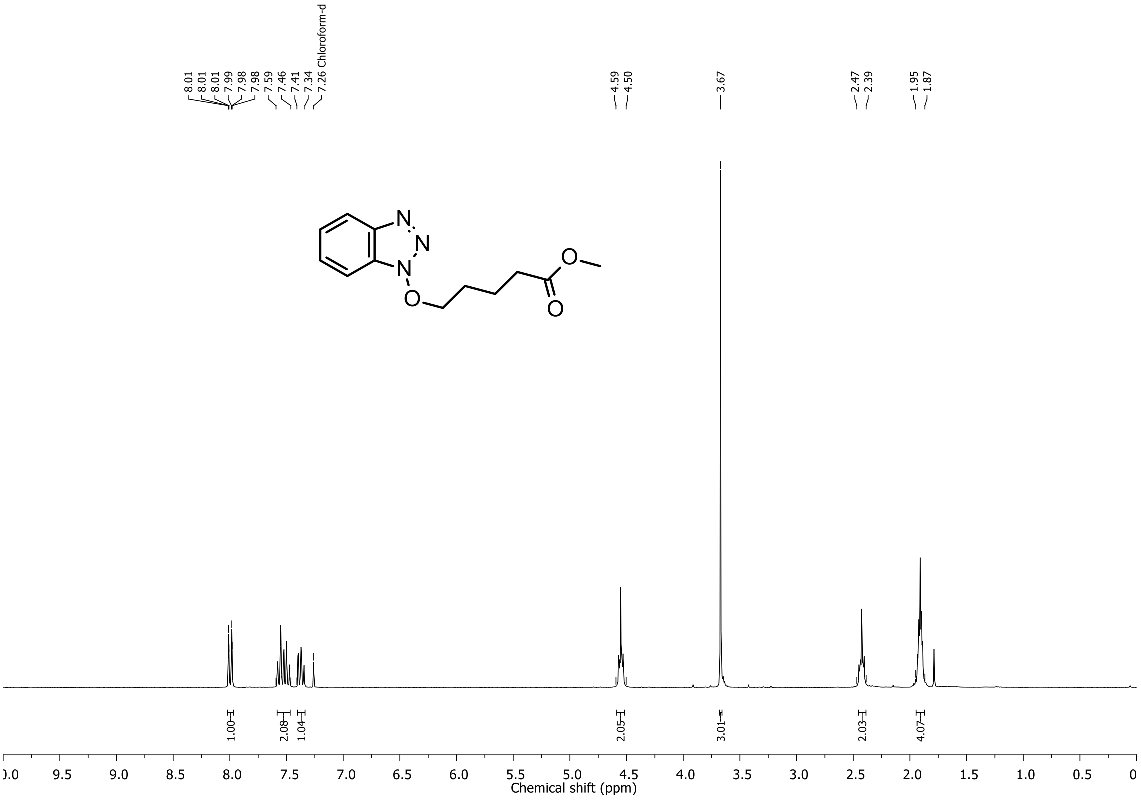

^{13}C NMR ($CDCl_3$, 101 MHz)

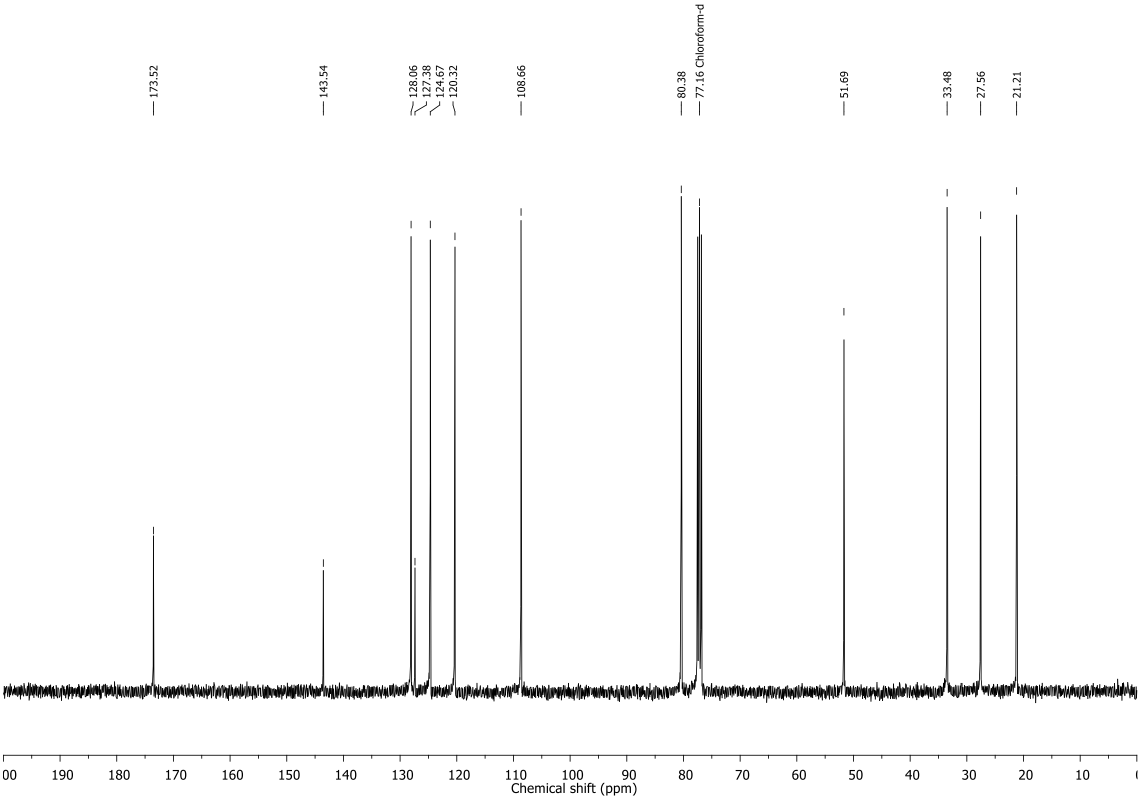

5-((1*H*-Benzo[*d*][1,2,3]triazol-1-yl)oxy)-1-phenylpentan-1-one (3qa)

^{1}H NMR ($CDCl_3$, 300 MHz)

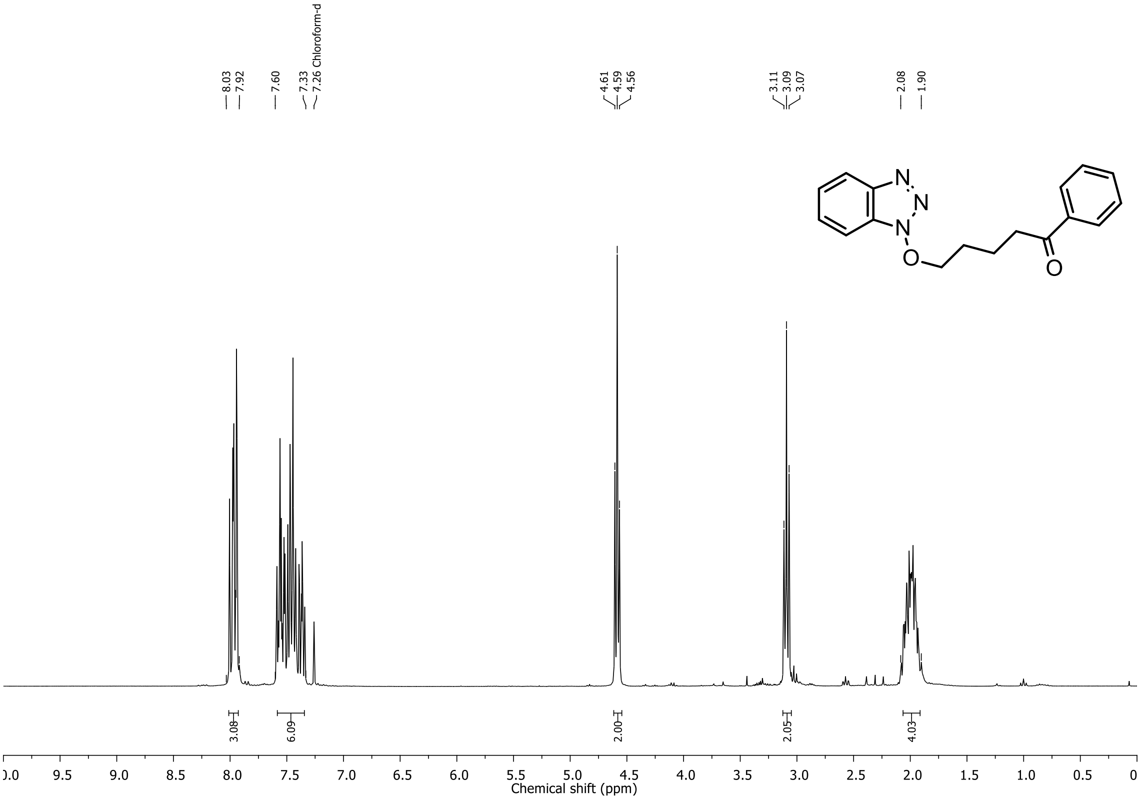

^{13}C NMR ($CDCl_3$, 75 MHz)

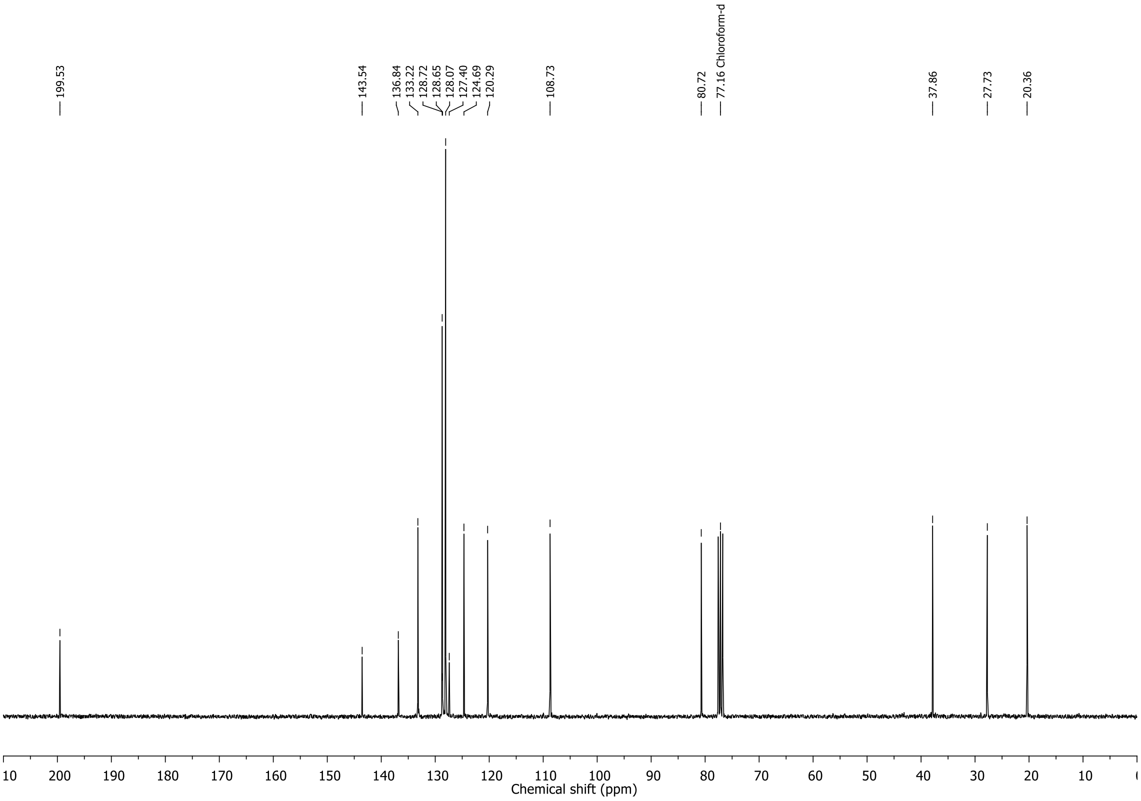

1-((1*H*-Benzo[*d*][1,2,3]triazol-1-yl)oxy)propan-2-ol (3ra) [CAS: 1866463-44-6]

^{1}H NMR ($CDCl_3$, 300 MHz)

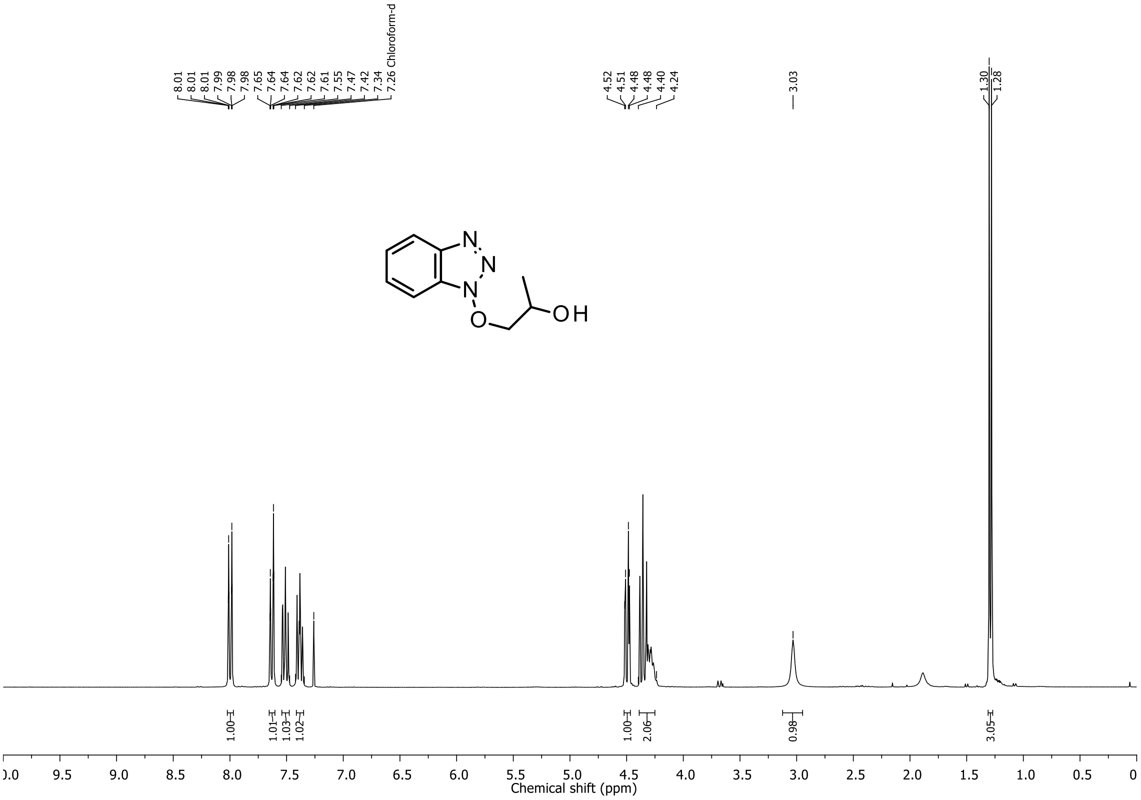

^{13}C NMR ($CDCl_3$, 75 MHz)

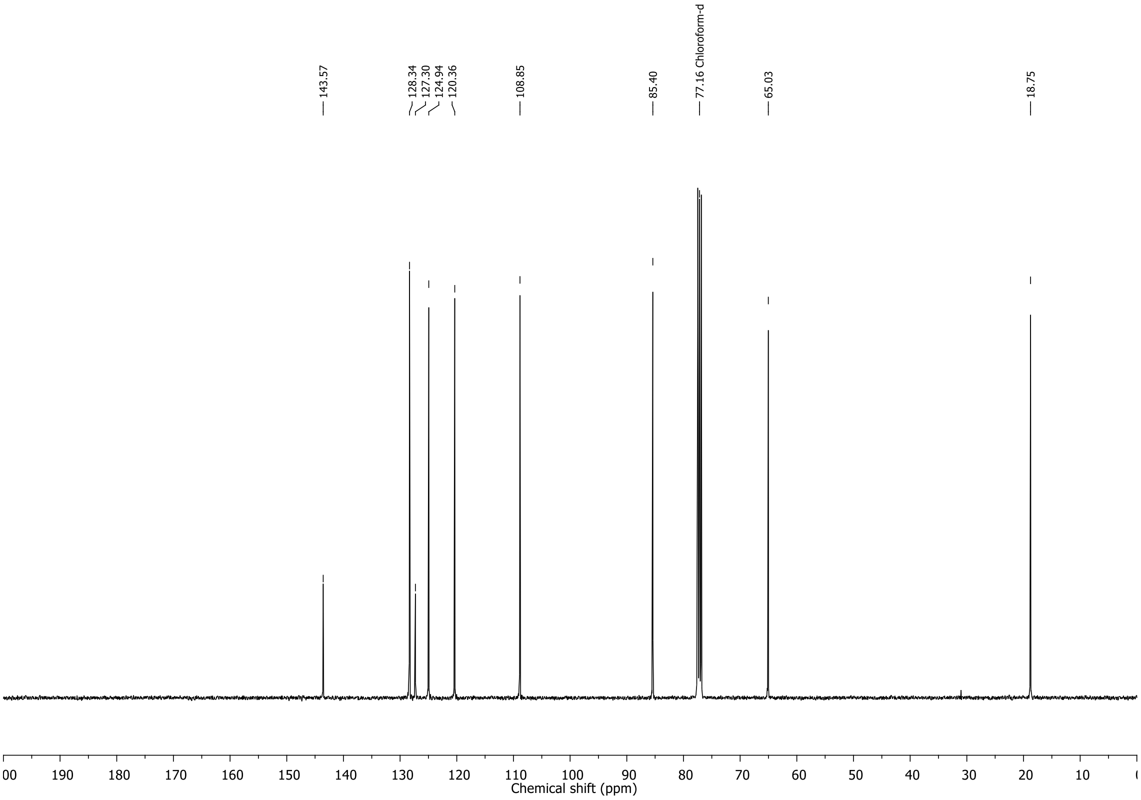

1-((2-Methyl-1,3-dioxolan-2-yl)methoxy)-1*H*-benzo[*d*][1,2,3]triazole (3sa)

^{1}H NMR ($CDCl_3$, 300 MHz)

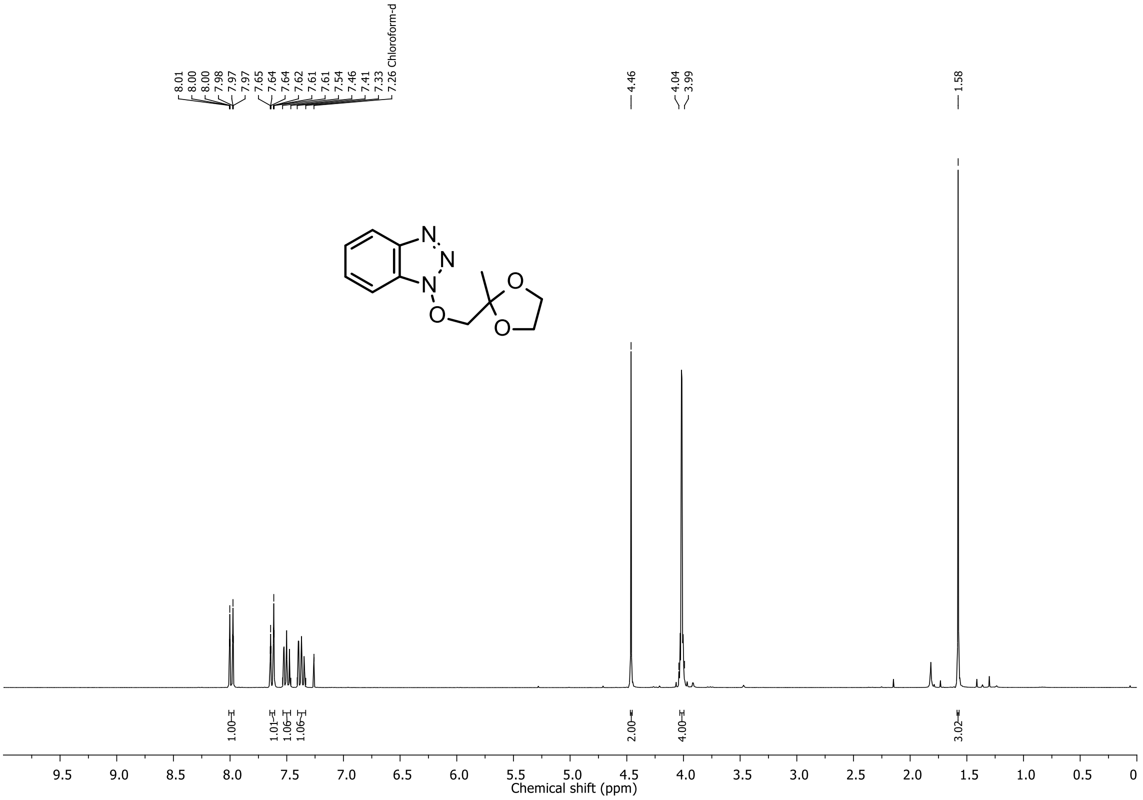

^{13}C NMR ($CDCl_3$, 75 MHz)

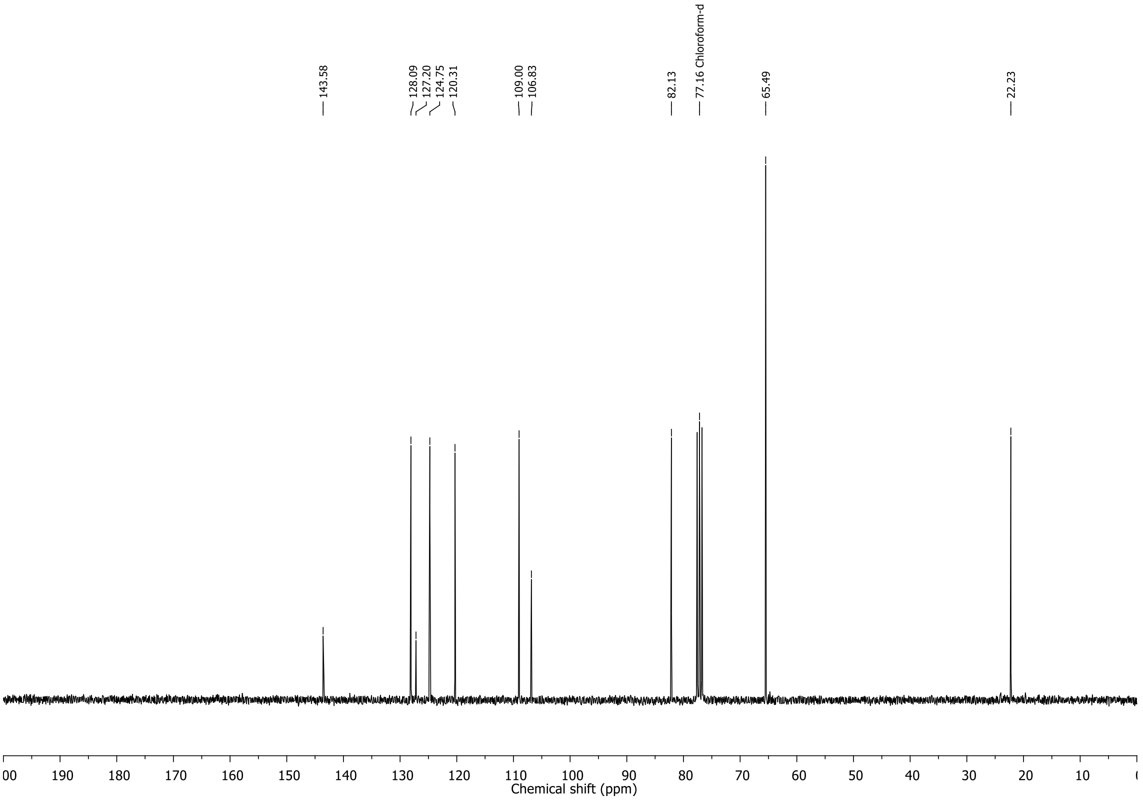

Methyl 4-((1*H*-benzo[*d*][1,2,3]triazol-1-yl)oxy)-2-((*tert*-butoxycarbonyl)amino)butanoate (3ta)

^{1}H NMR ($CDCl_3$, 300 MHz)

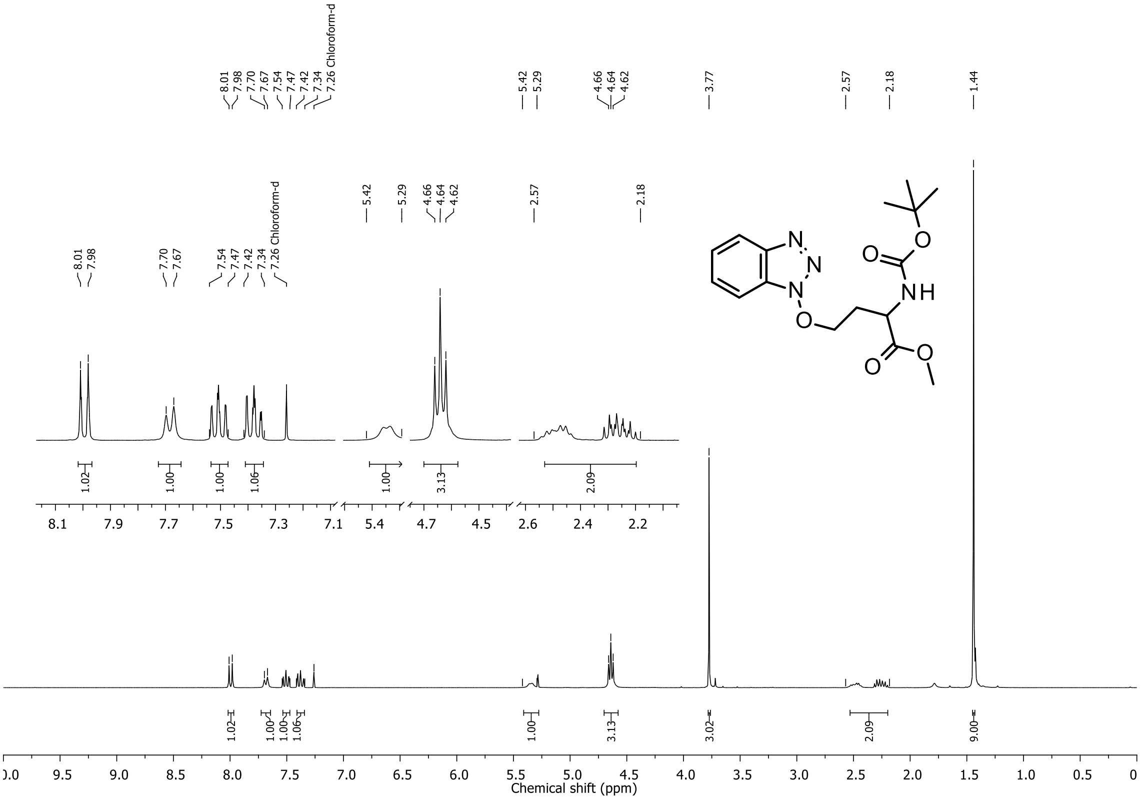

^{13}C NMR ($CDCl_3$, 75 MHz)

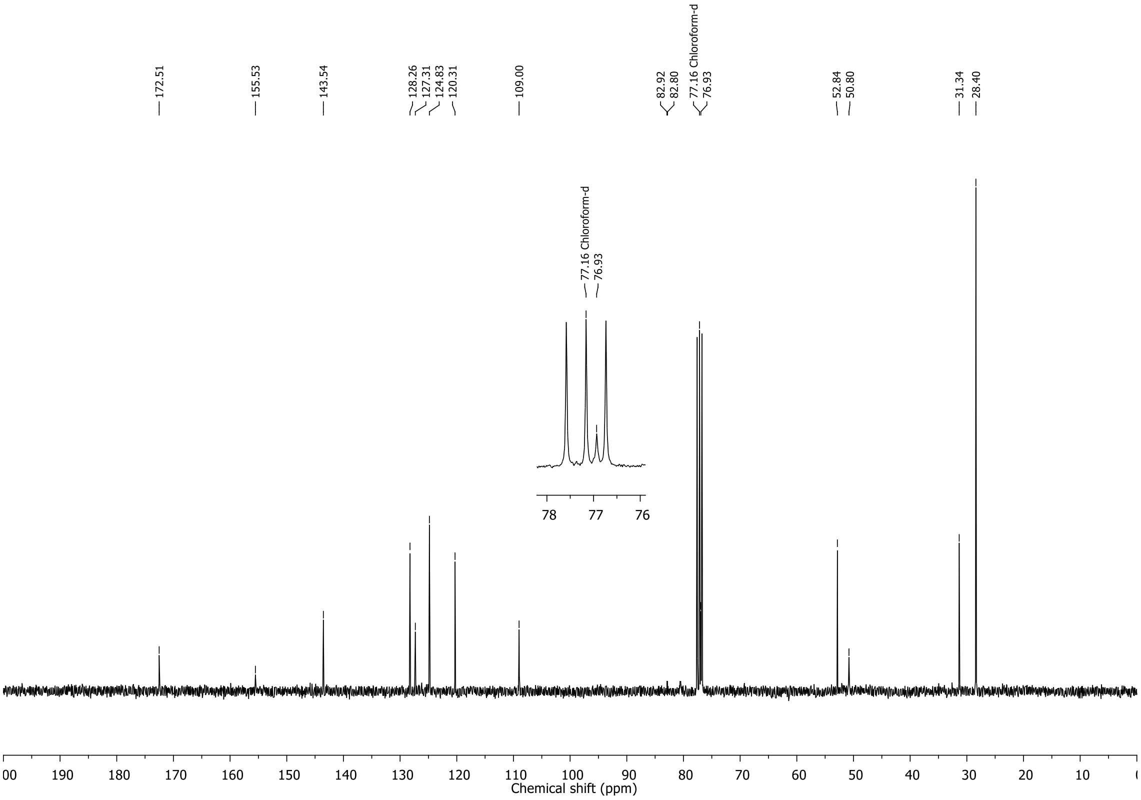

(3*R*,5*R*,8*R*,9*S*,10*S*,12*S*,13*R*,14*S*,17*R*)-17-((*R*)-4-((1*H*-Benzo[*d*][1,2,3]triazol-1-yl)oxy)butan-2-yl)-10,13-dimethylhexadecahydro-1*H*-cyclopenta[*a*]phenanthrene-3,12-diol (3ua)

^{1}H NMR ($CDCl_3$, 300 MHz)

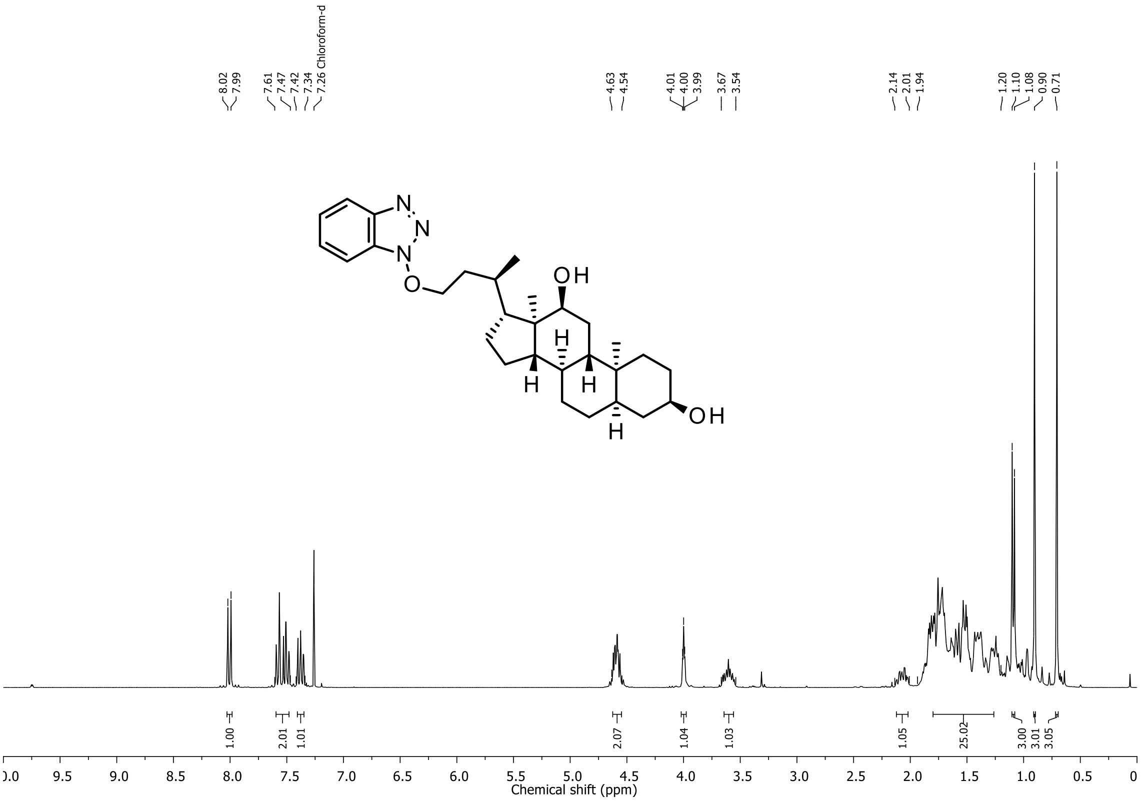

^{13}C NMR ($CDCl_3$, 75 MHz)

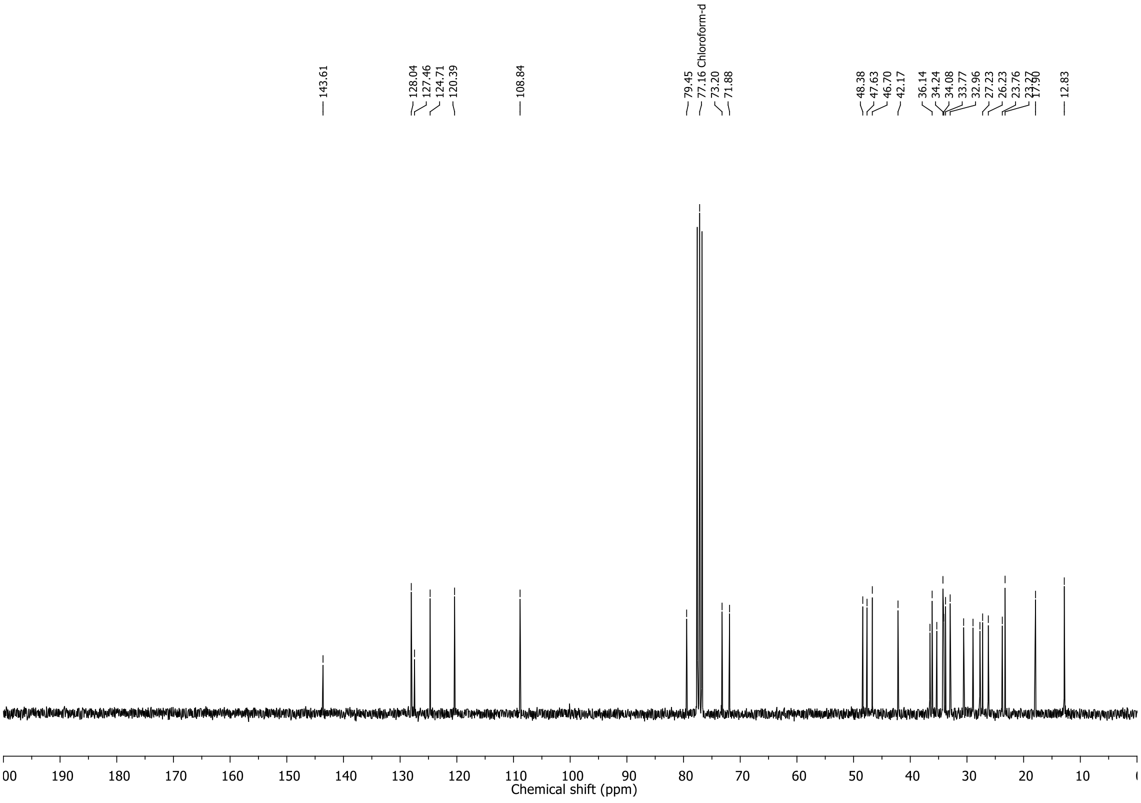

(8*R*,9*S*,10*S*,13*R*,14*S*,17*R*)-17-((*R*)-4-((1*H*-Benzo[*d*][1,2,3]triazol-1-yl)oxy)butan-2-yl)-10,13-dimethyldecahydro-1*H*-cyclopenta[*a*]phenanthrene-3,7,12(2*H*,4*H*,8*H*)-trione (3va)

^{1}H NMR (CDCl$_3$, 300 MHz)

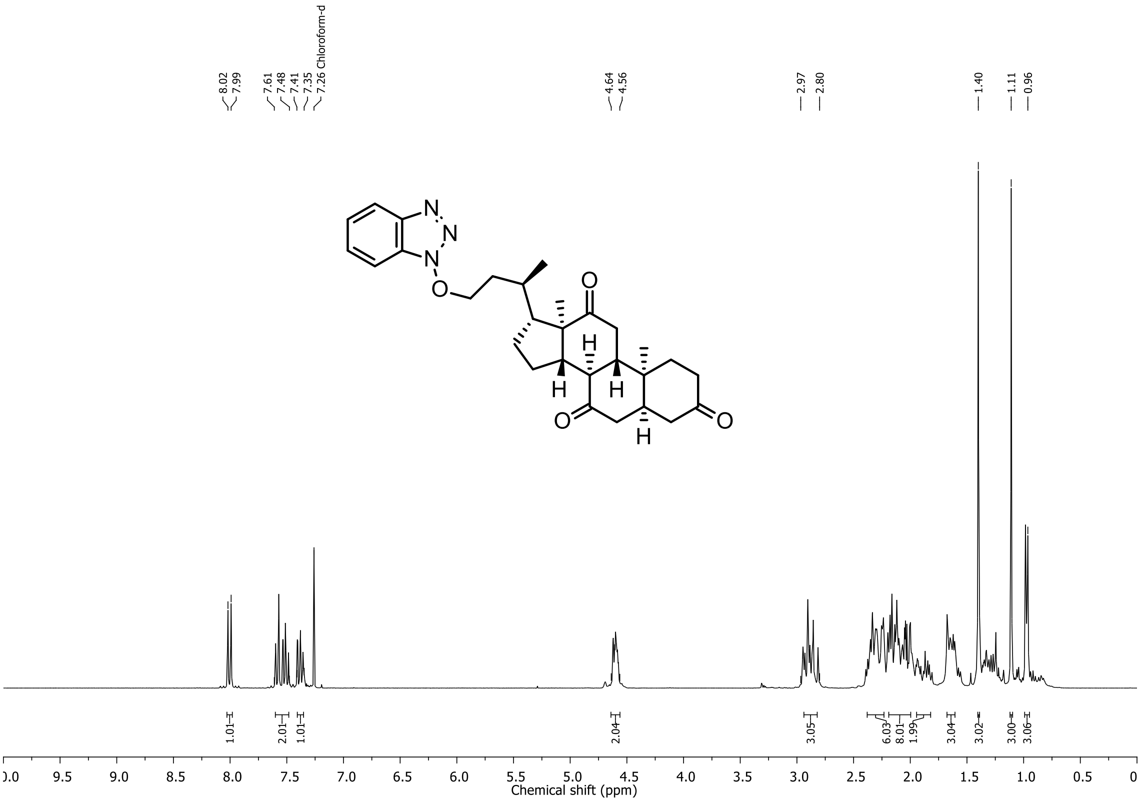

^{13}C NMR (CDCl$_3$, 75 MHz)

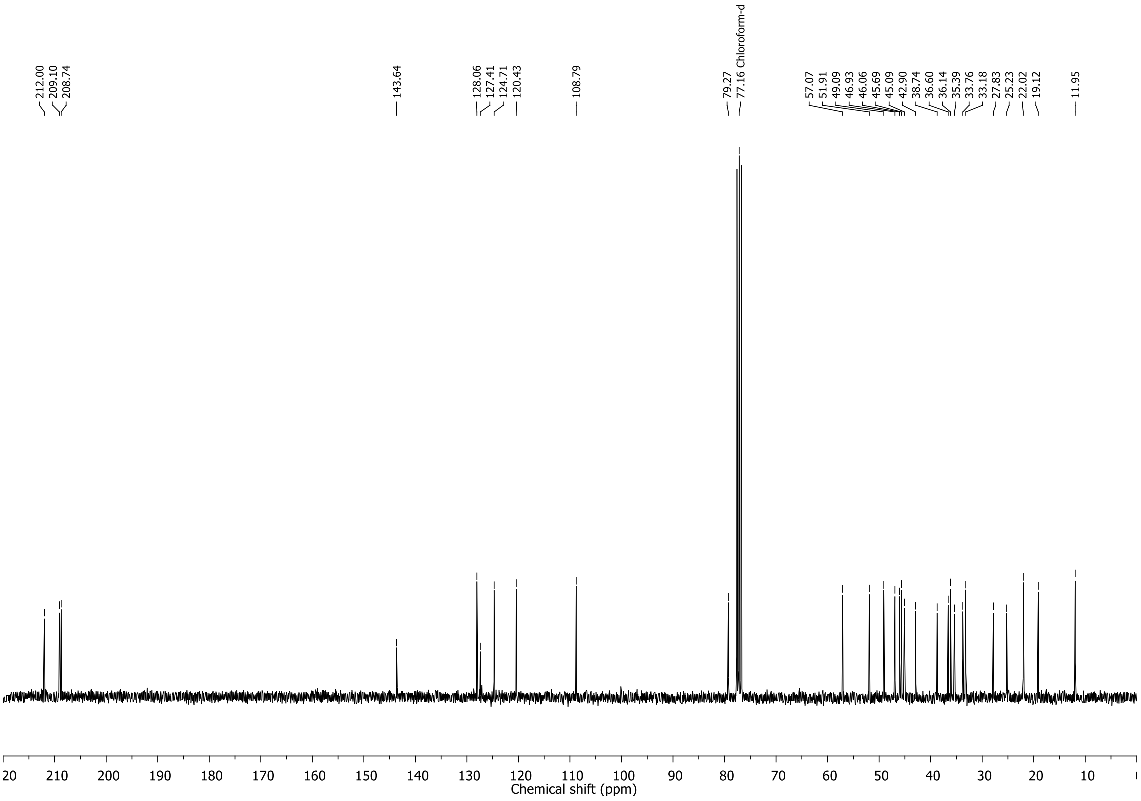

(*E*)-1-(Hexadec-7-en-1-yloxy)-1*H*-benzo[*d*][1,2,3]triazole (3wa)

^{1}H NMR ($CDCl_3$, 300 MHz)

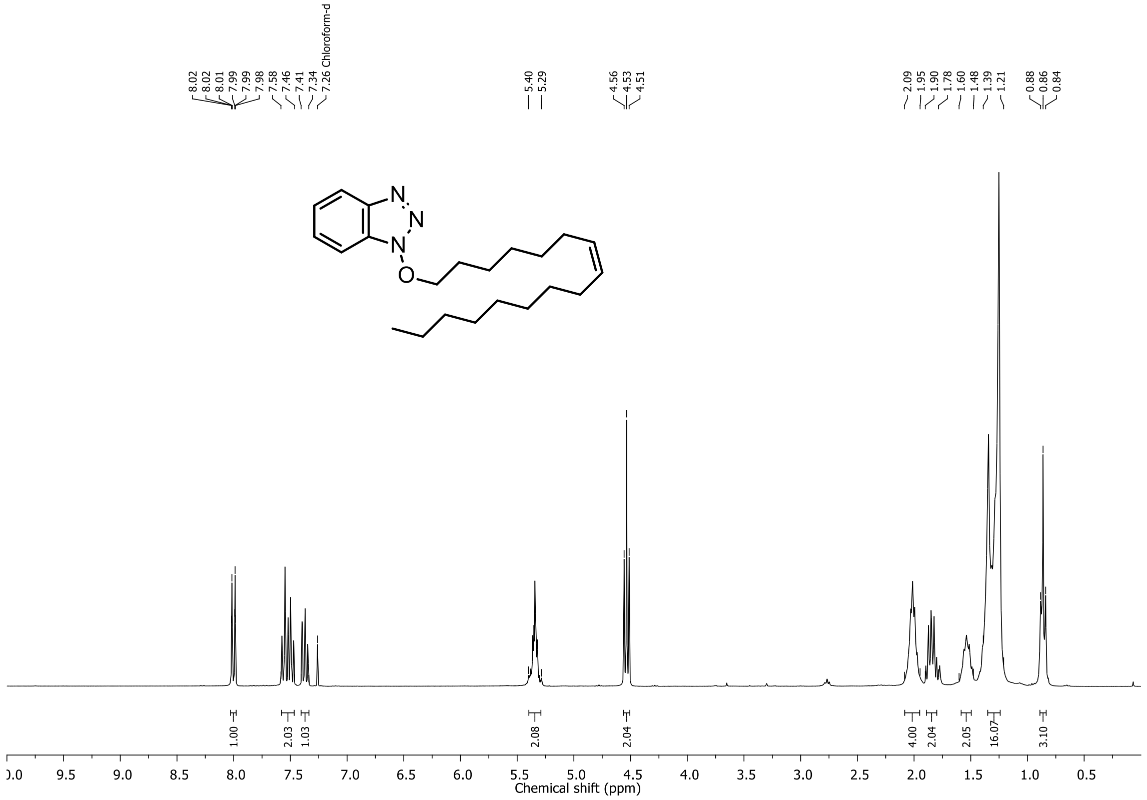

^{13}C NMR ($CDCl_3$, 101 MHz)

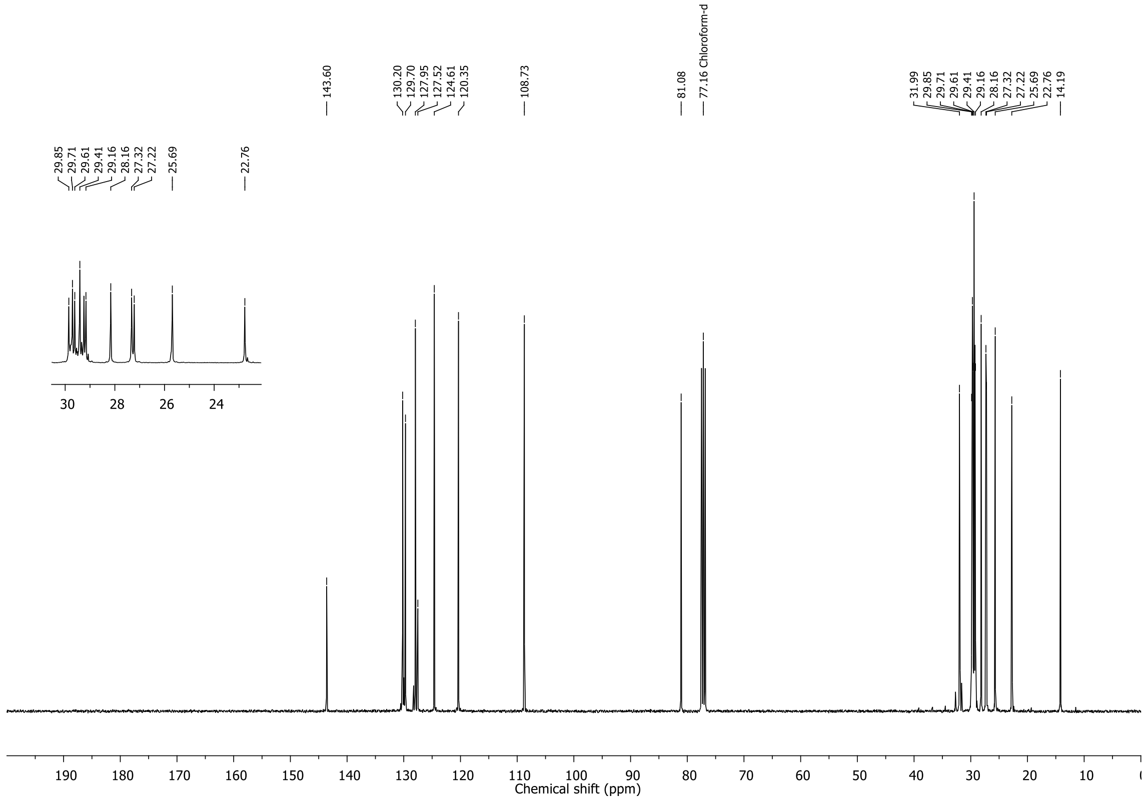

1-Isopropoxy-1*H*-benzo[*d*][1,2,3]triazole (3xa) [CAS: 57223-17-3]

^{1}H NMR ($CDCl_3$, 250 MHz)

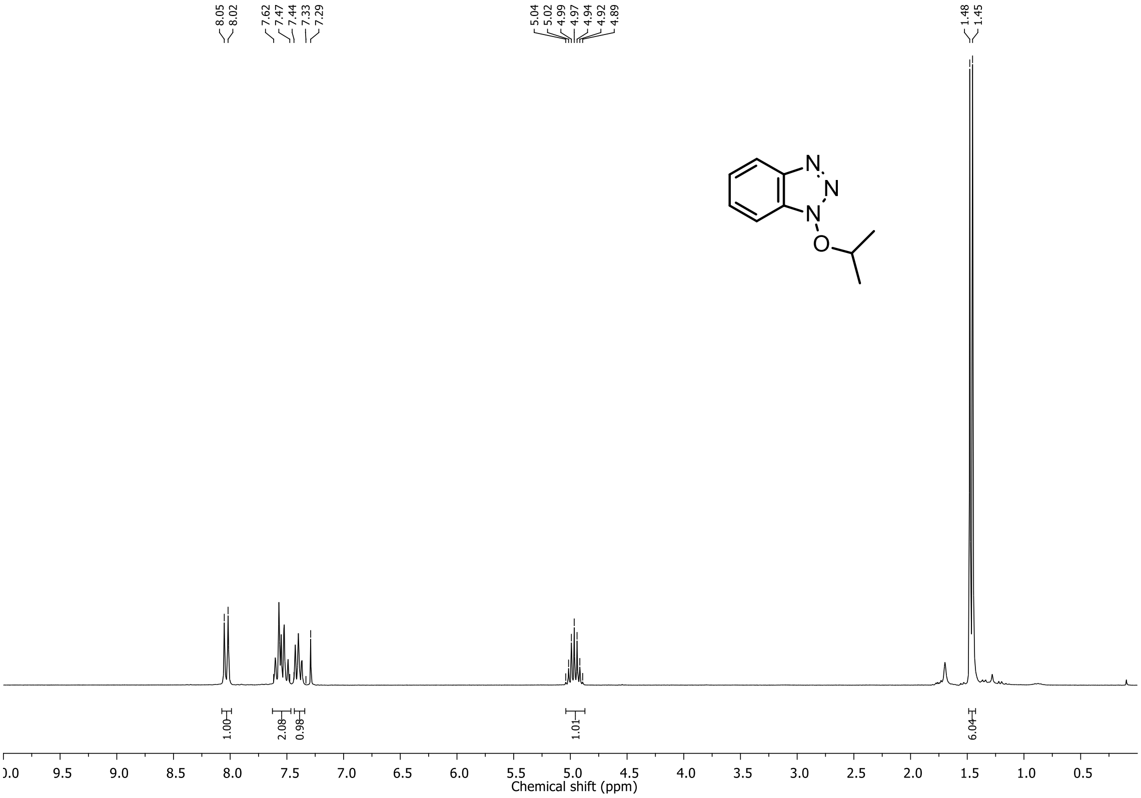

^{13}C NMR ($CDCl_3$, 63 MHz)

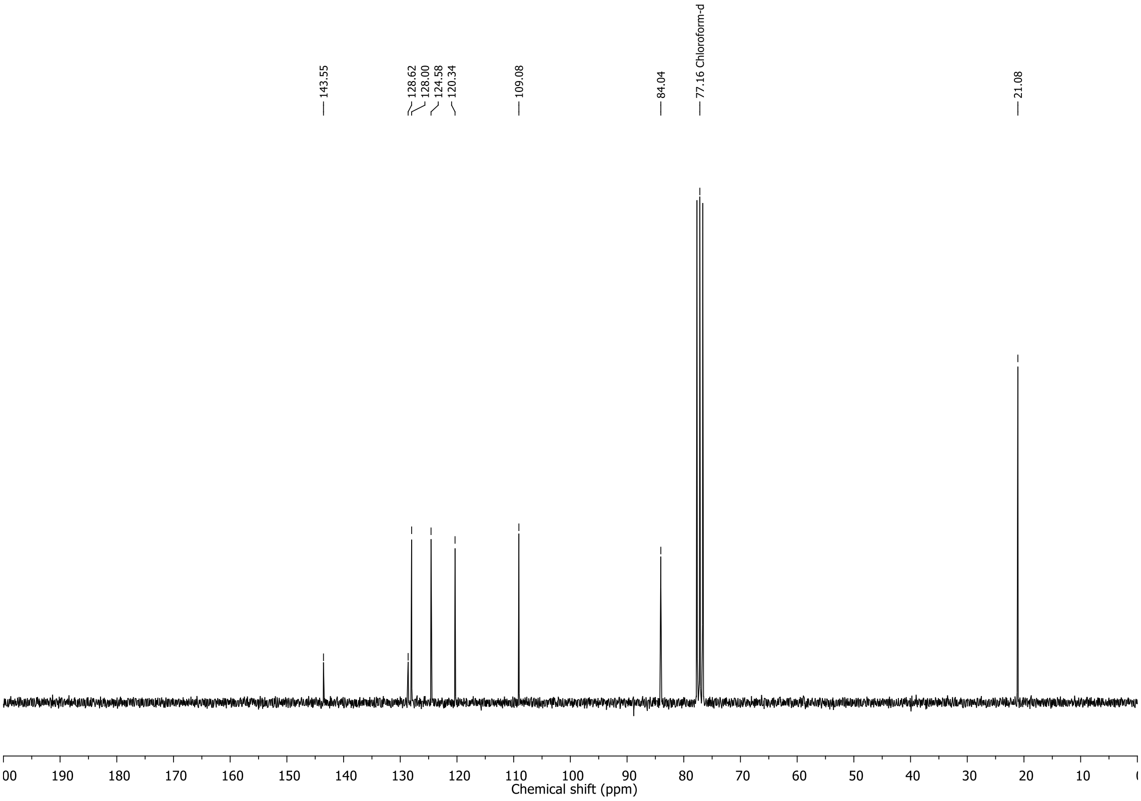

1-(Pentan-3-yloxy)-1*H*-benzo[*d*][1,2,3]triazole (3ya)

^{1}H NMR ($CDCl_3$, 300 MHz)

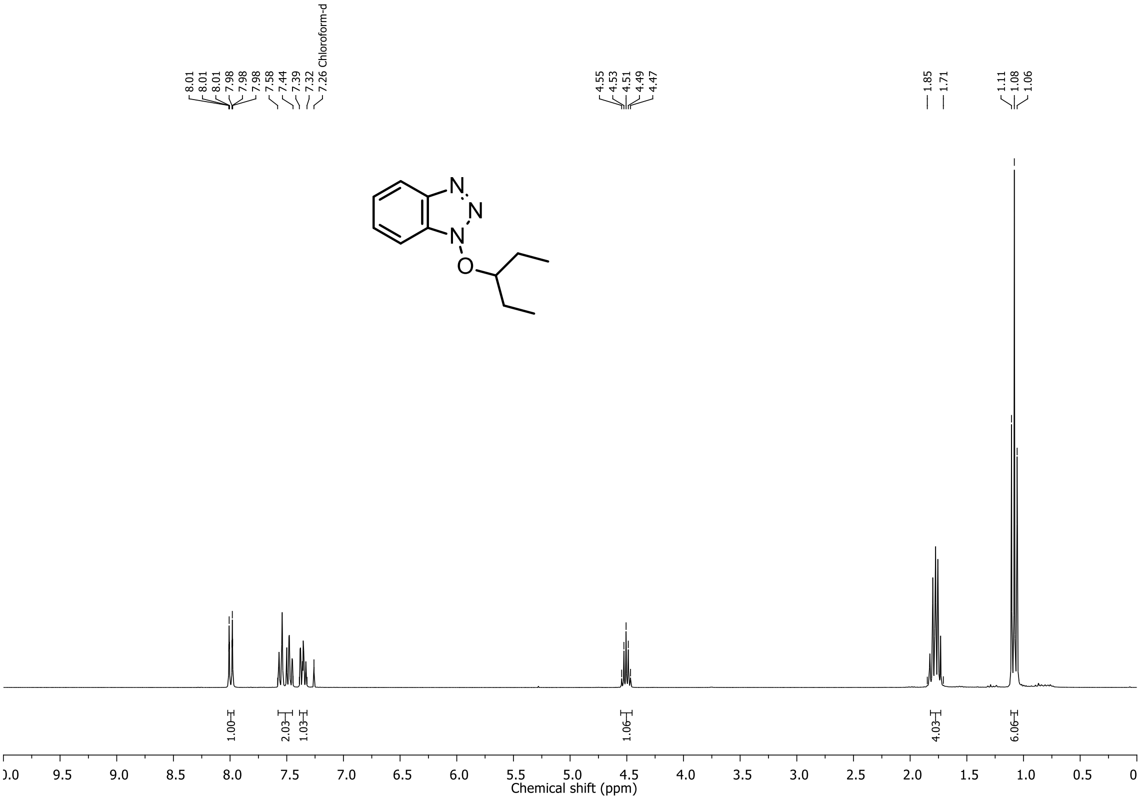

^{13}C NMR ($CDCl_3$, 75 MHz)

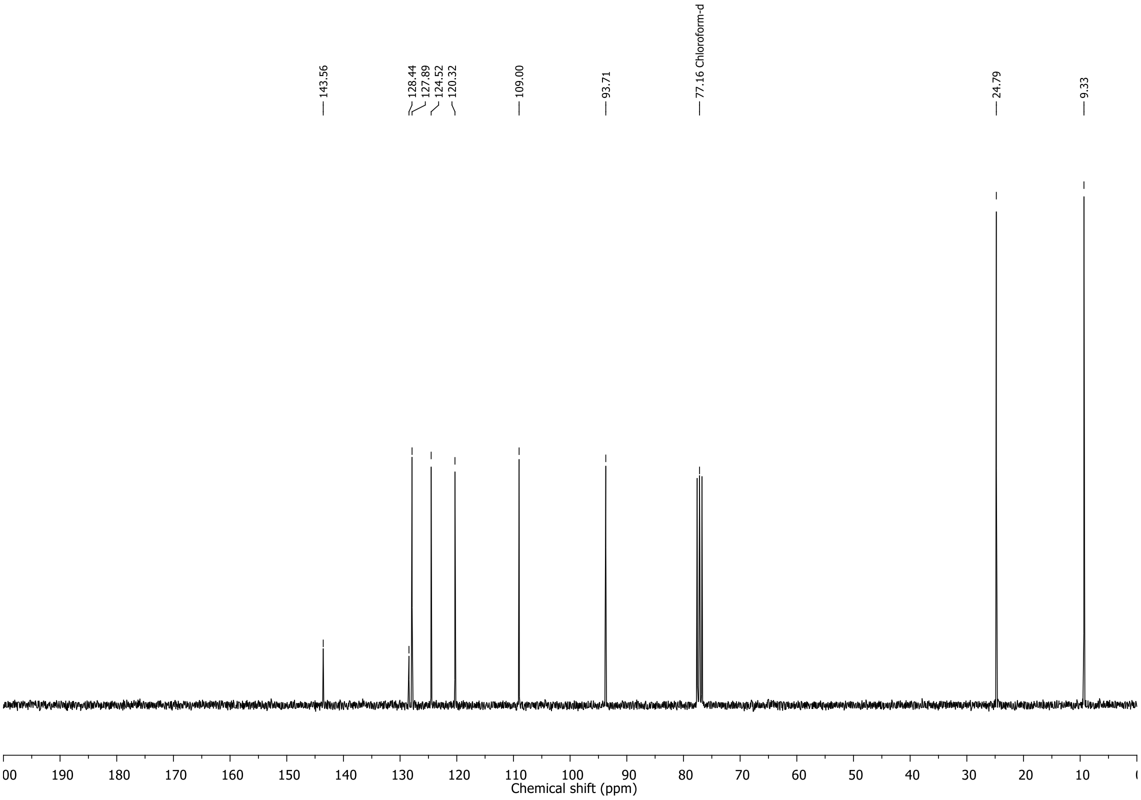

1-(Heptan-4-yloxy)-1*H*-benzo[*d*][1,2,3]triazole (3za)

^{1}H NMR ($CDCl_3$, 300 MHz)

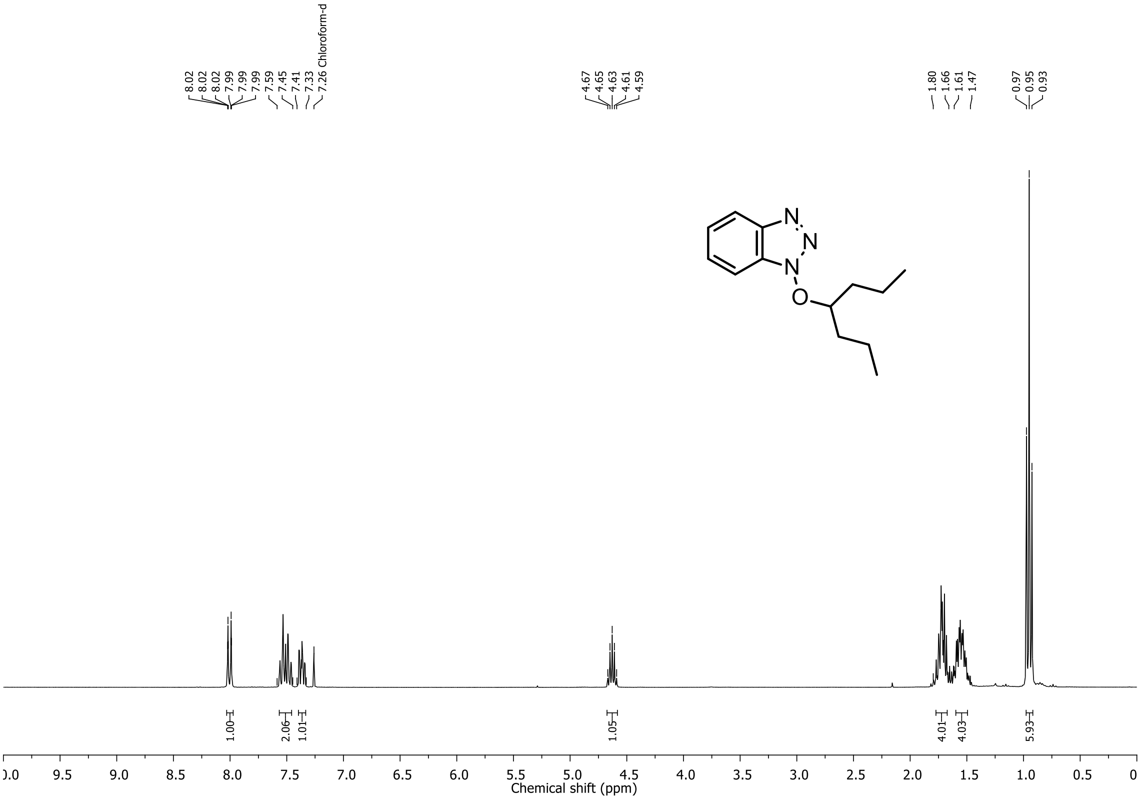

^{13}C NMR ($CDCl_3$, 75 MHz)

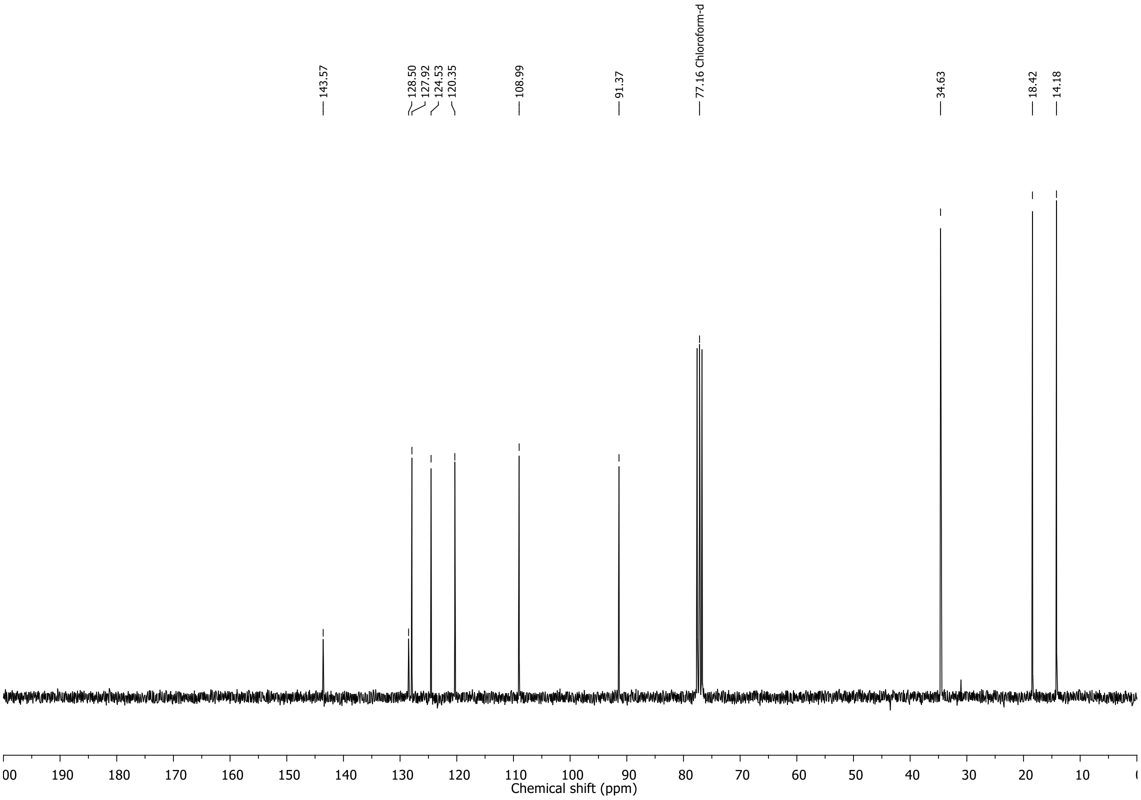

1-(Hexan-2-yloxy)-1*H*-benzo[*d*][1,2,3]triazole (3a′a)

^{1}H NMR ($CDCl_3$, 300 MHz)

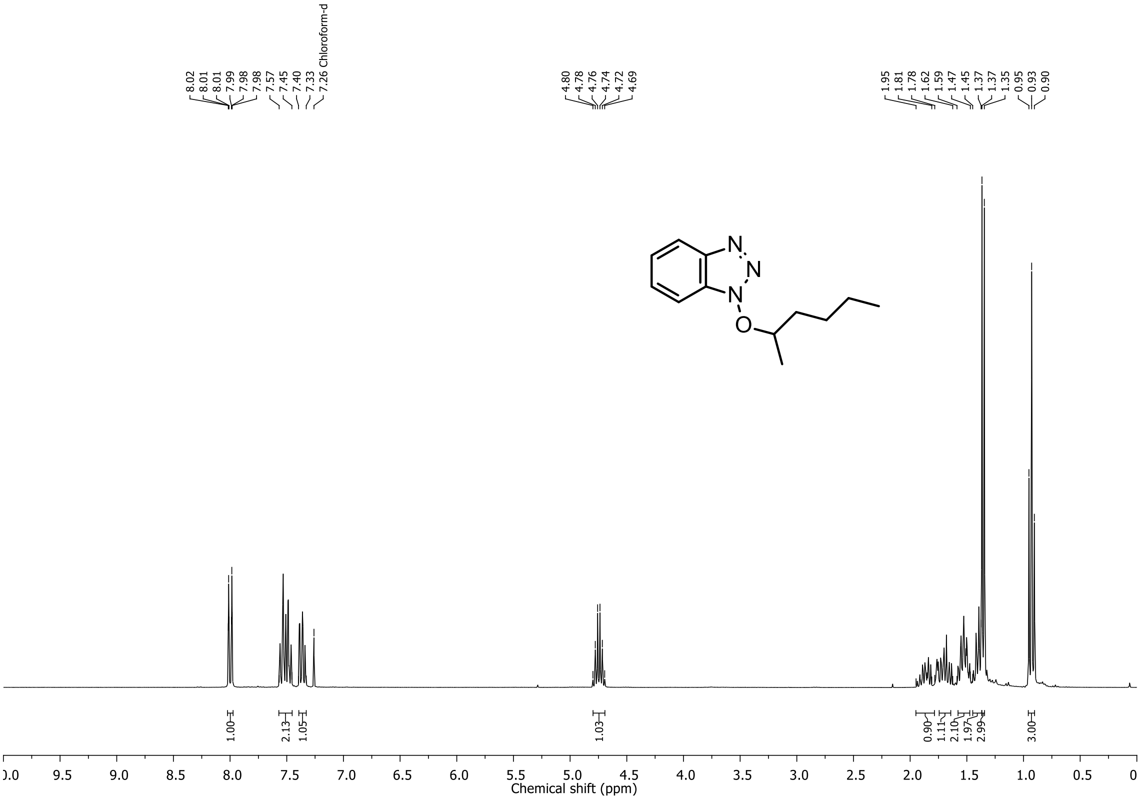

^{13}C NMR ($CDCl_3$, 75 MHz)

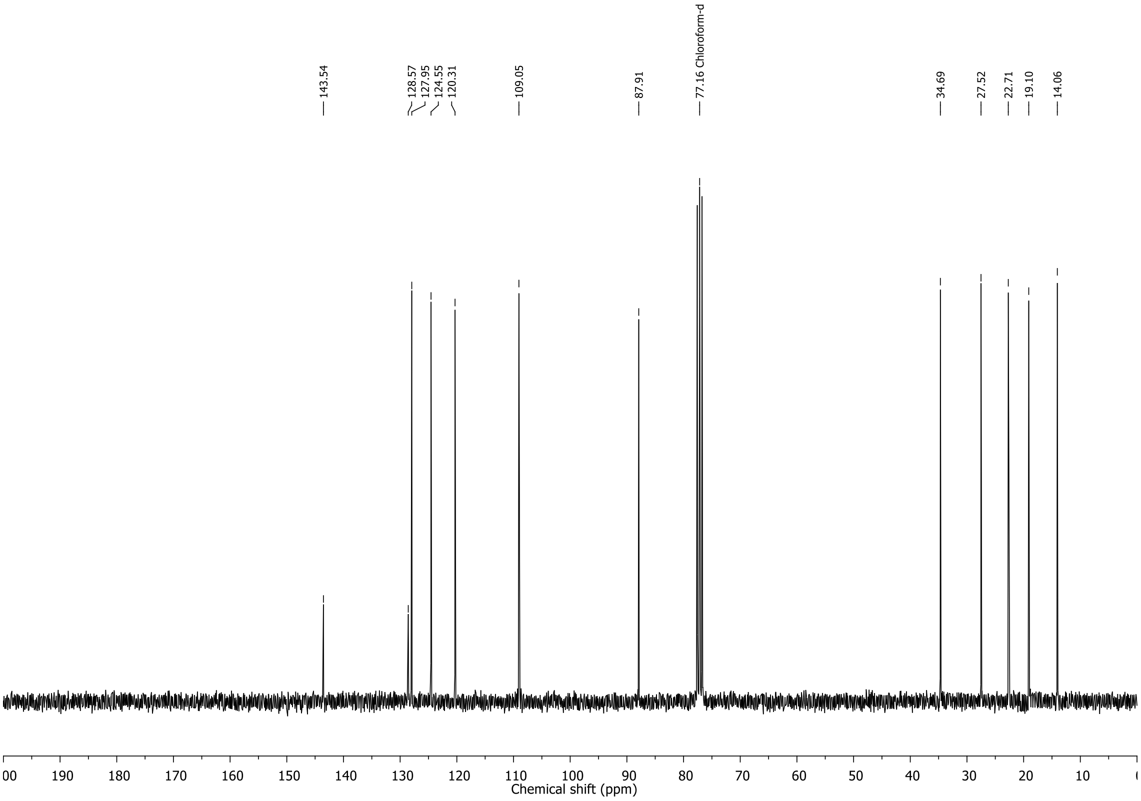

1-((1-Phenylpropan-2-yl)oxy)-1*H*-benzo[*d*][1,2,3]triazole (3b′a)

^{1}H NMR ($CDCl_3$, 300 MHz)

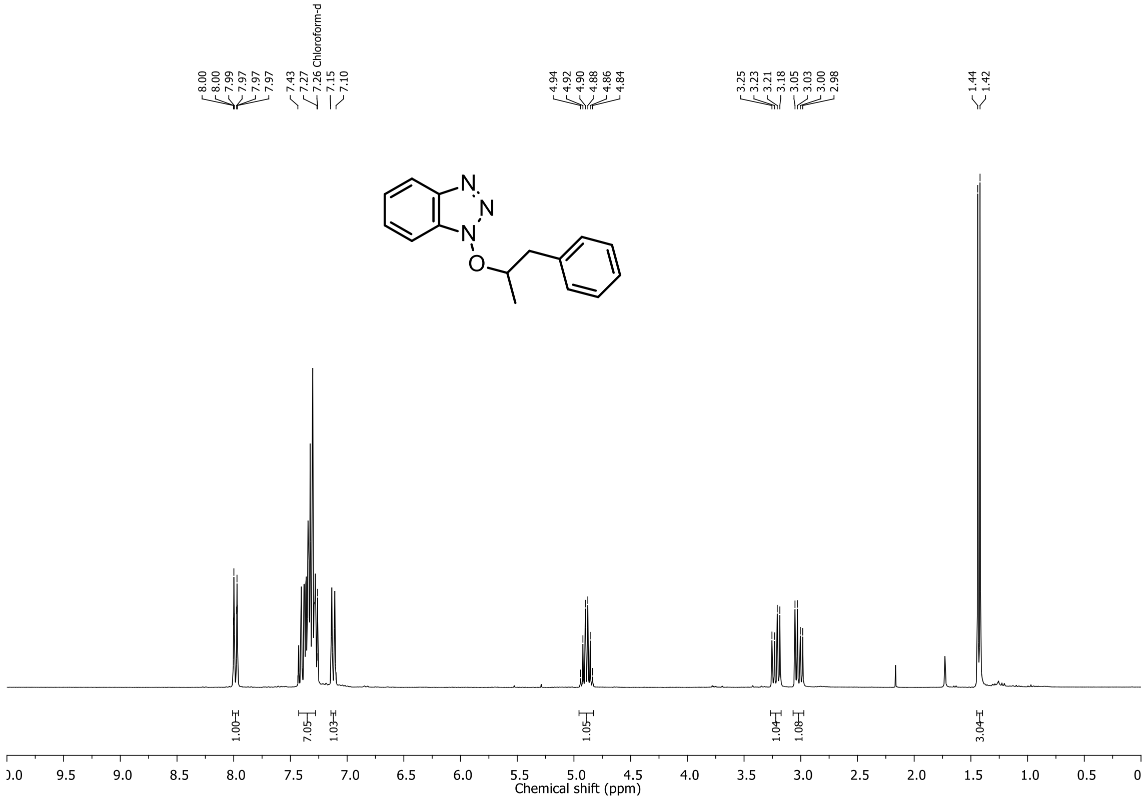

^{13}C NMR ($CDCl_3$, 75 MHz)

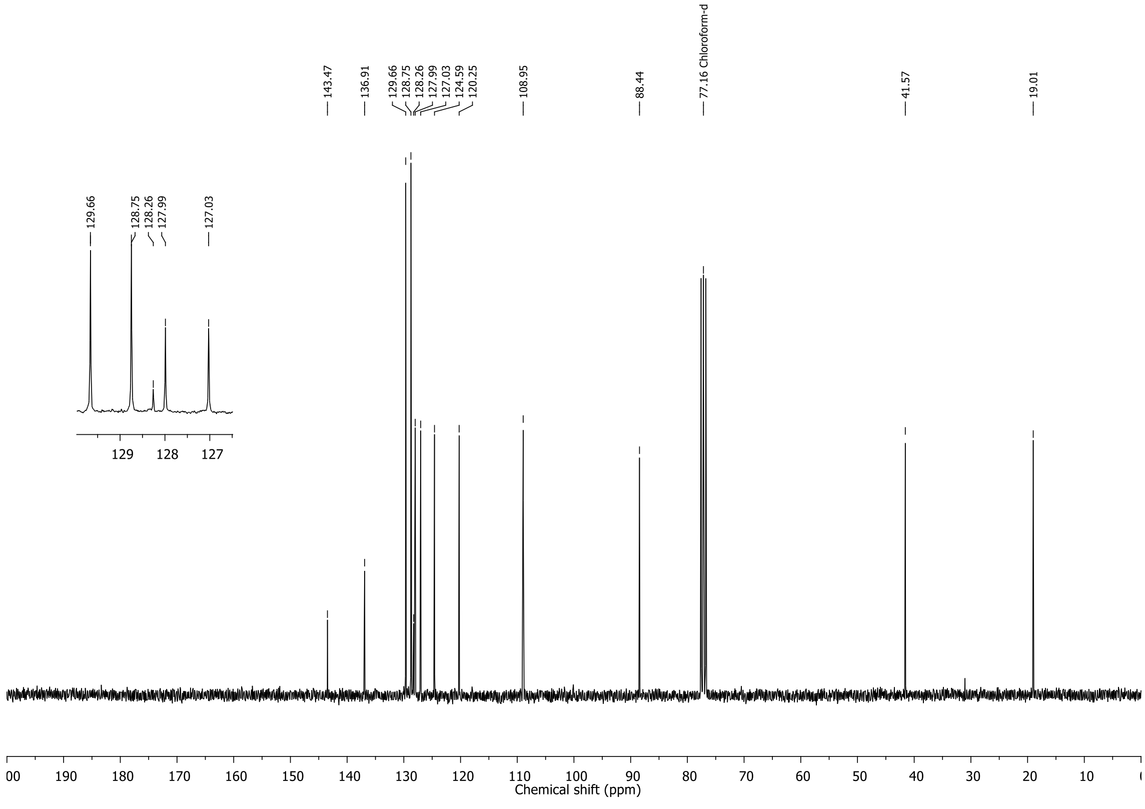

1-Cyclopropoxy-1*H*-benzo[*d*][1,2,3]triazole (3c′a) [CAS: 1864424-46-3]

^{1}H NMR ($CDCl_3$, 300 MHz)

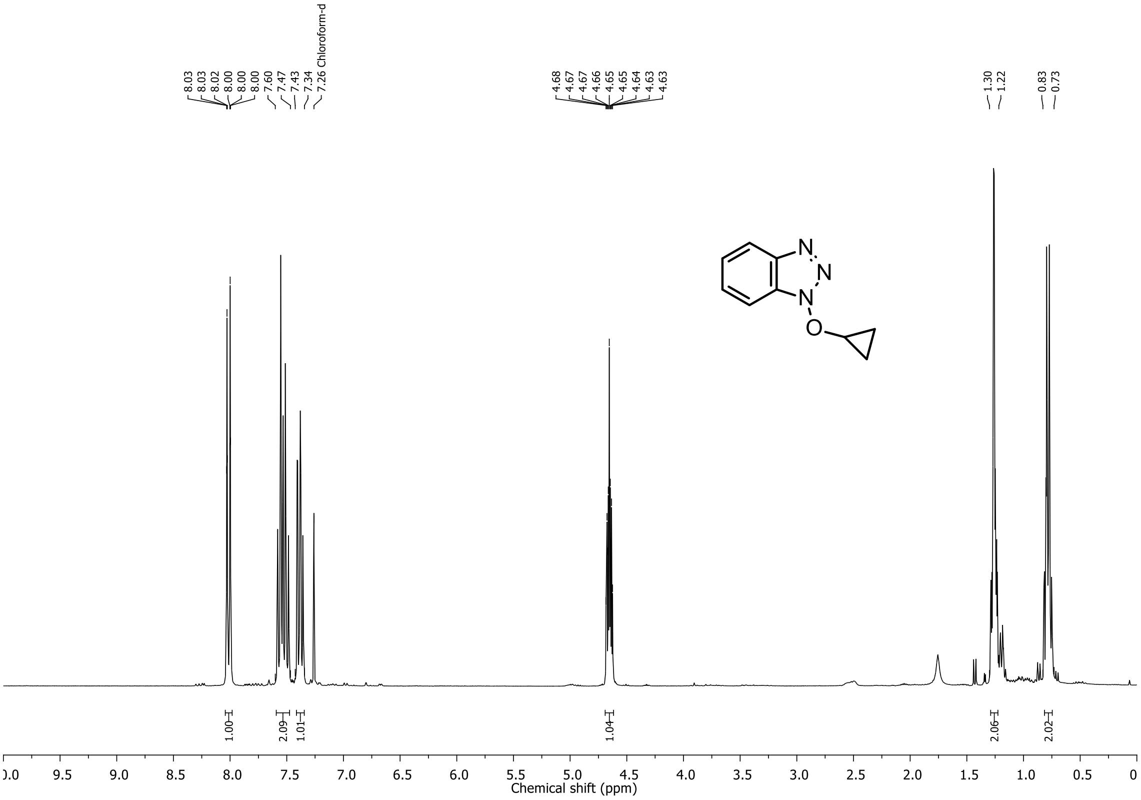

^{13}C NMR ($CDCl_3$, 75 MHz)

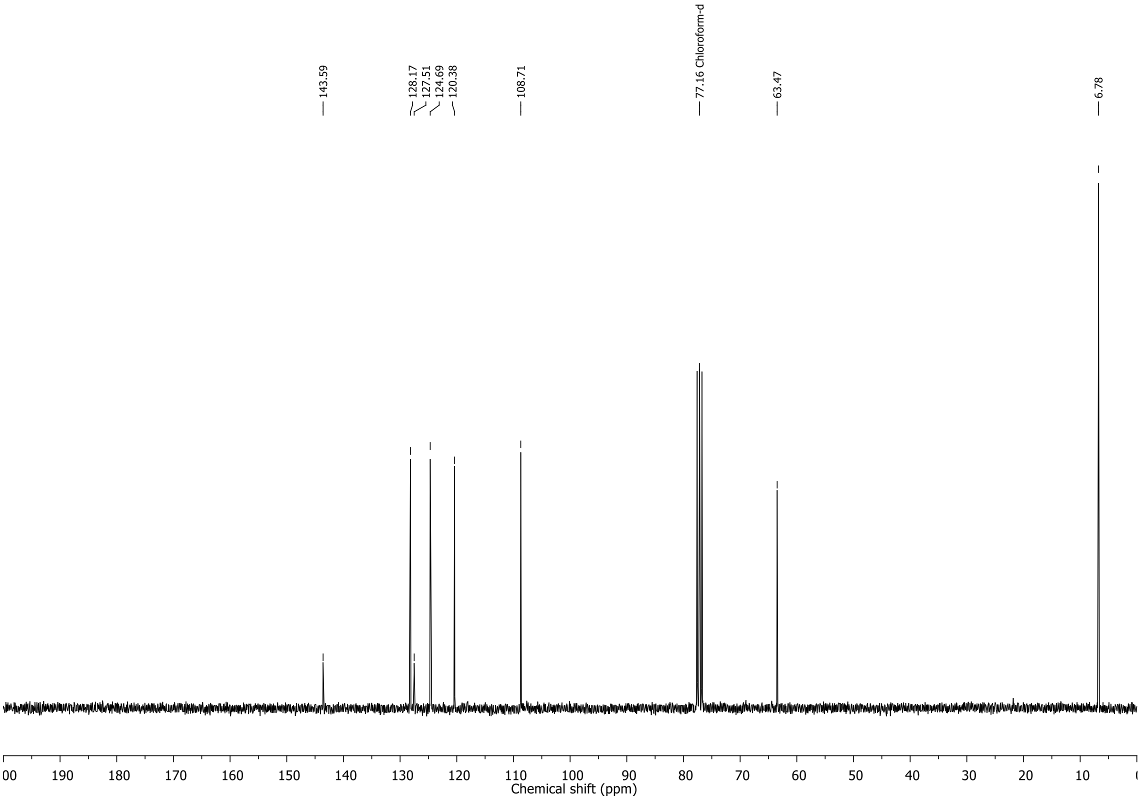

1-(Cyclopentyloxy)-1*H*-benzo[*d*][1,2,3]triazole (3d′a) [CAS: 60455-02-9]

^{1}H NMR ($CDCl_3$, 300 MHz)

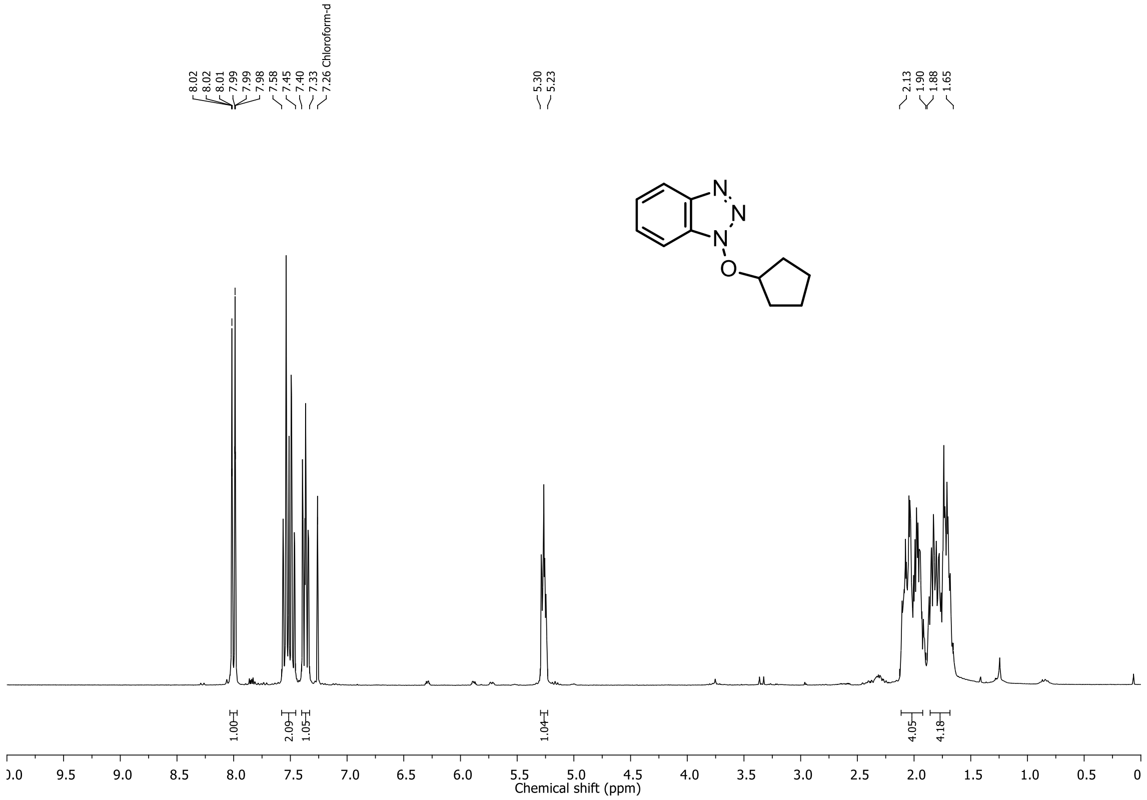

^{13}C NMR ($CDCl_3$, 75 MHz)

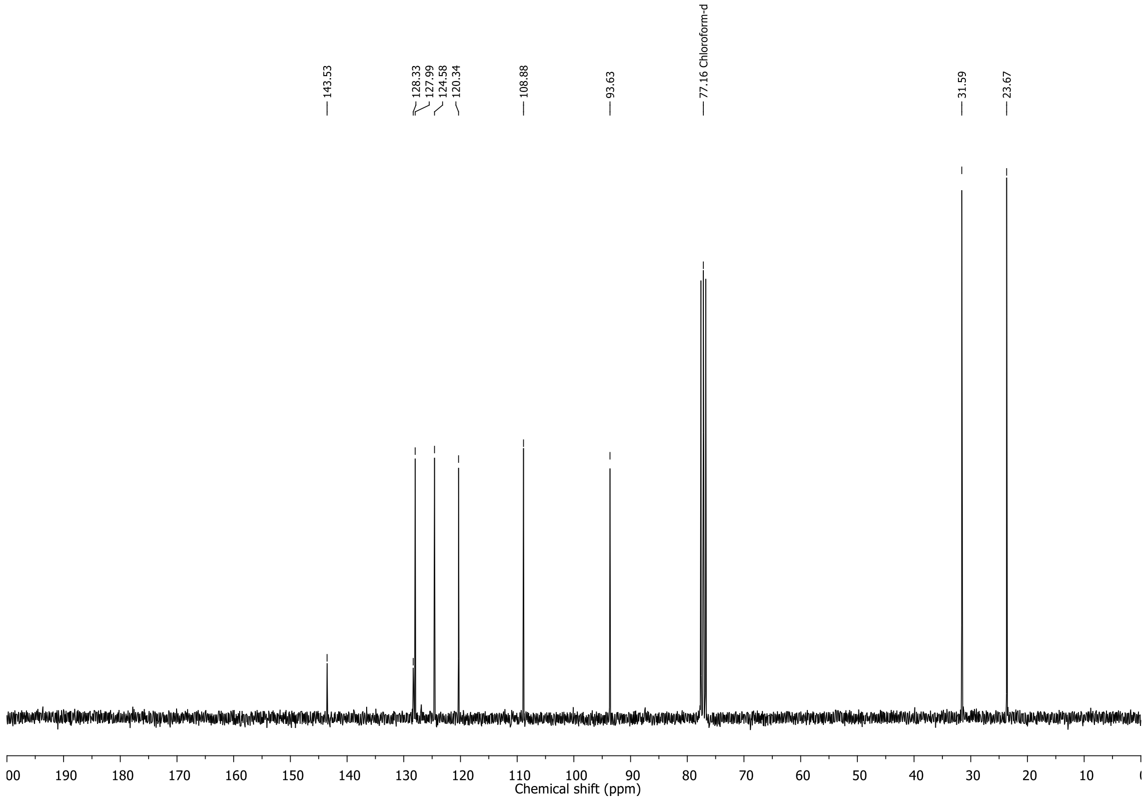

1-(Cyclohexyloxy)-1*H*-benzo[*d*][1,2,3]triazole (3e′a) [CAS: 1876594-76-1]

^{1}H NMR ($CDCl_3$, 300 MHz)

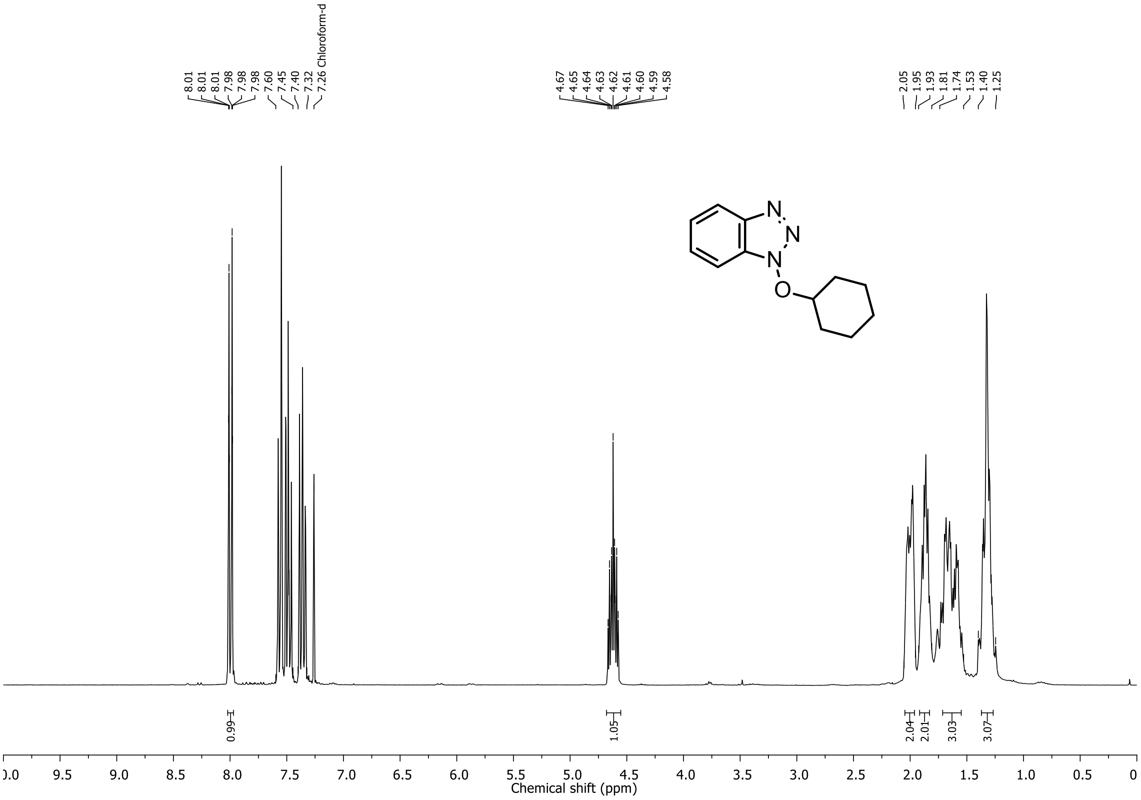

^{13}C NMR ($CDCl_3$, 75 MHz)

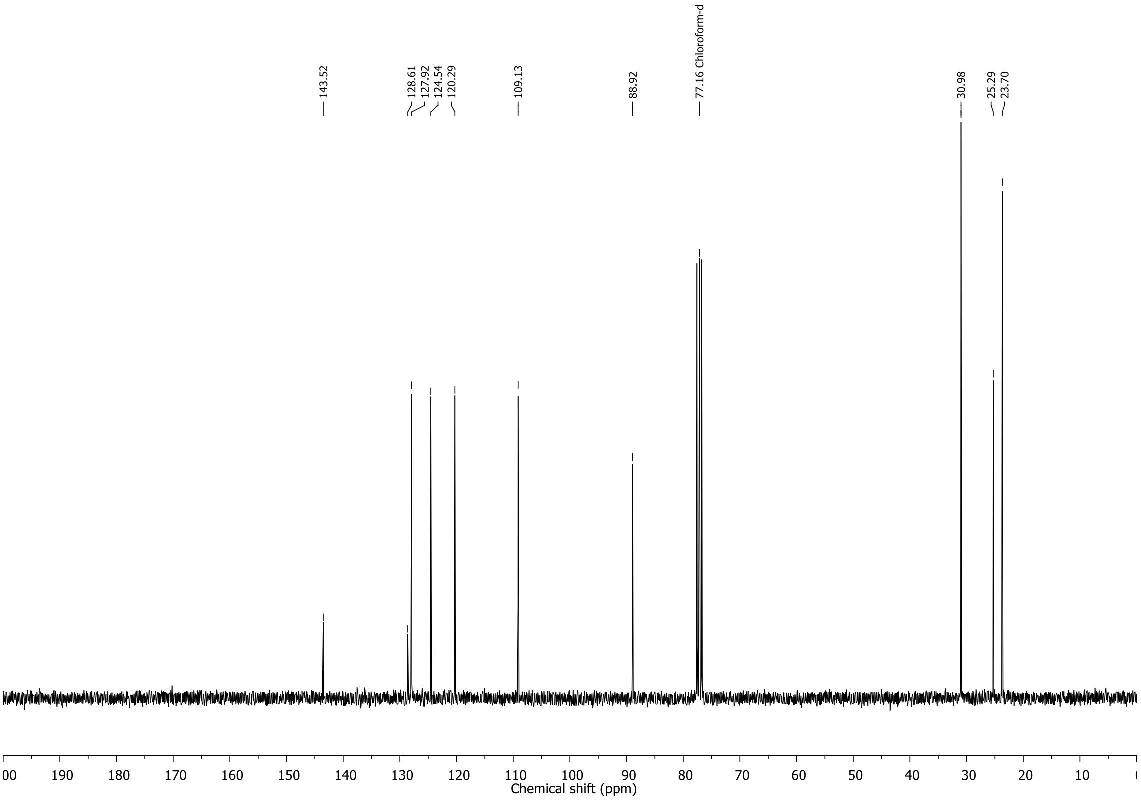

1-(Difluoromethoxy)-1*H*-benzo[*d*][1,2,3]triazole (3f′a) [CAS: 1861169-68-7]

^{1}H NMR ($CDCl_3$, 300 MHz)

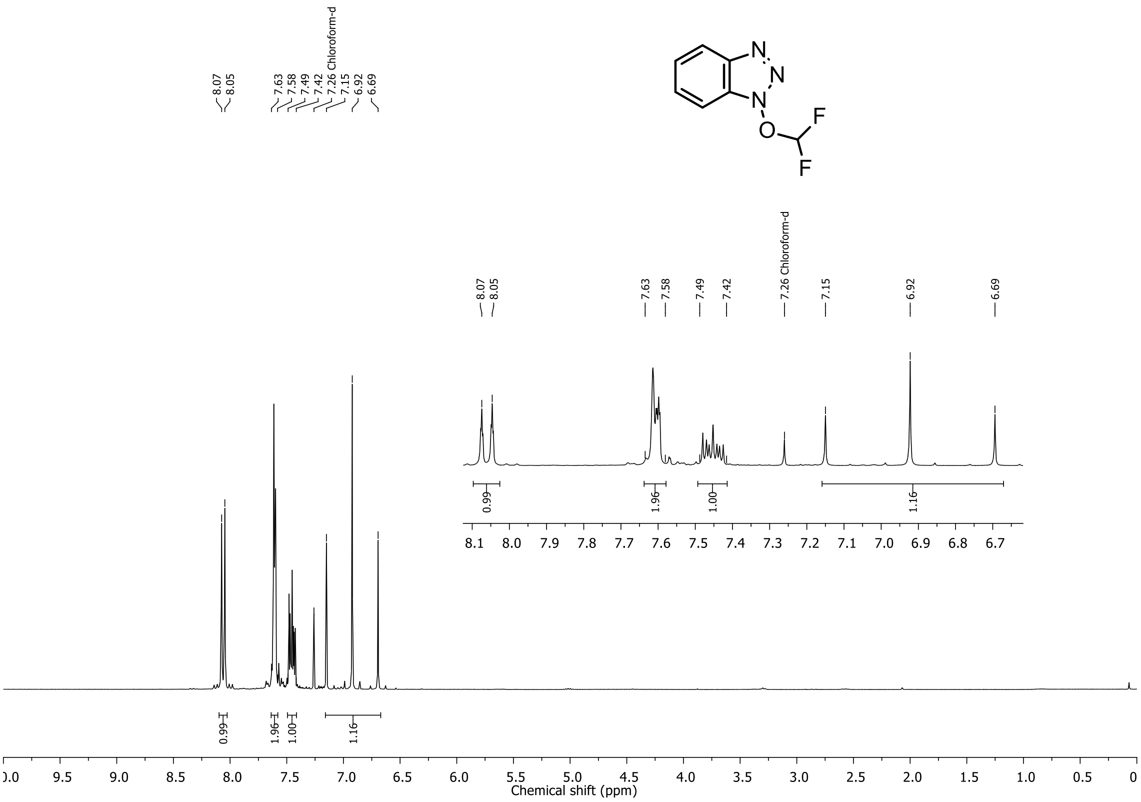

^{13}C NMR ($CDCl_3$, 75 MHz)

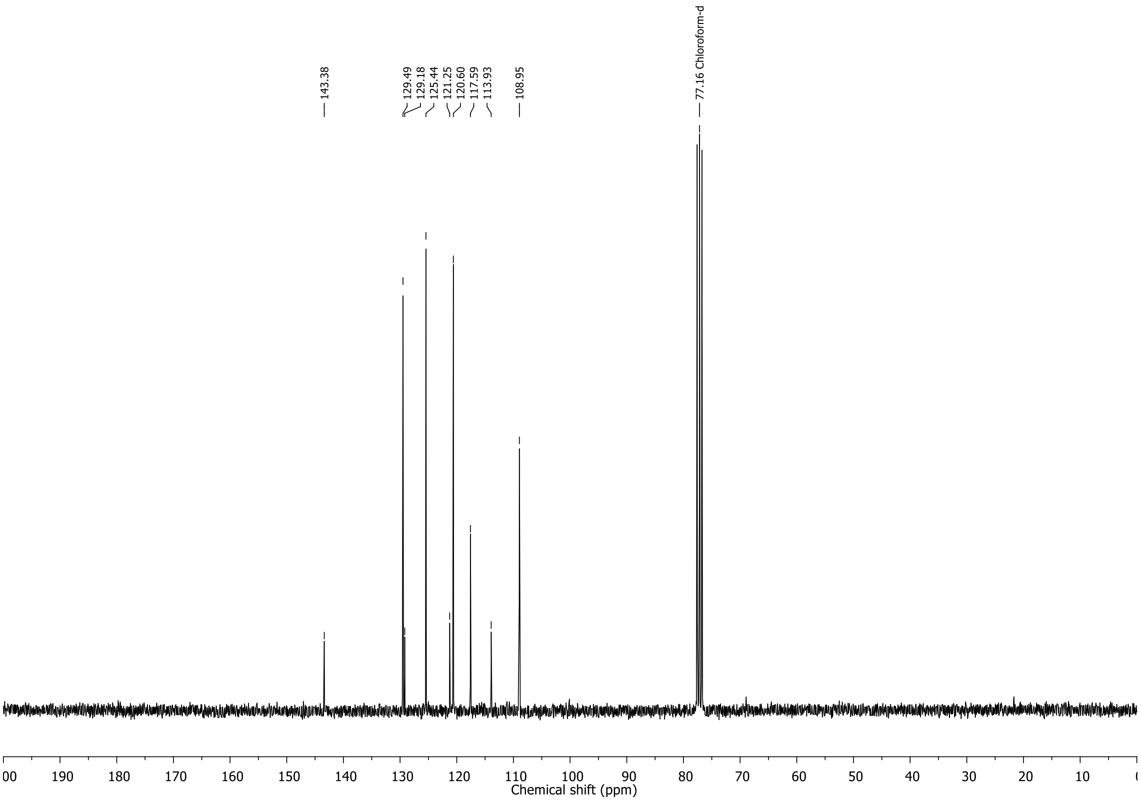

^{19}F NMR ($CDCl_3$, 235 MHz)

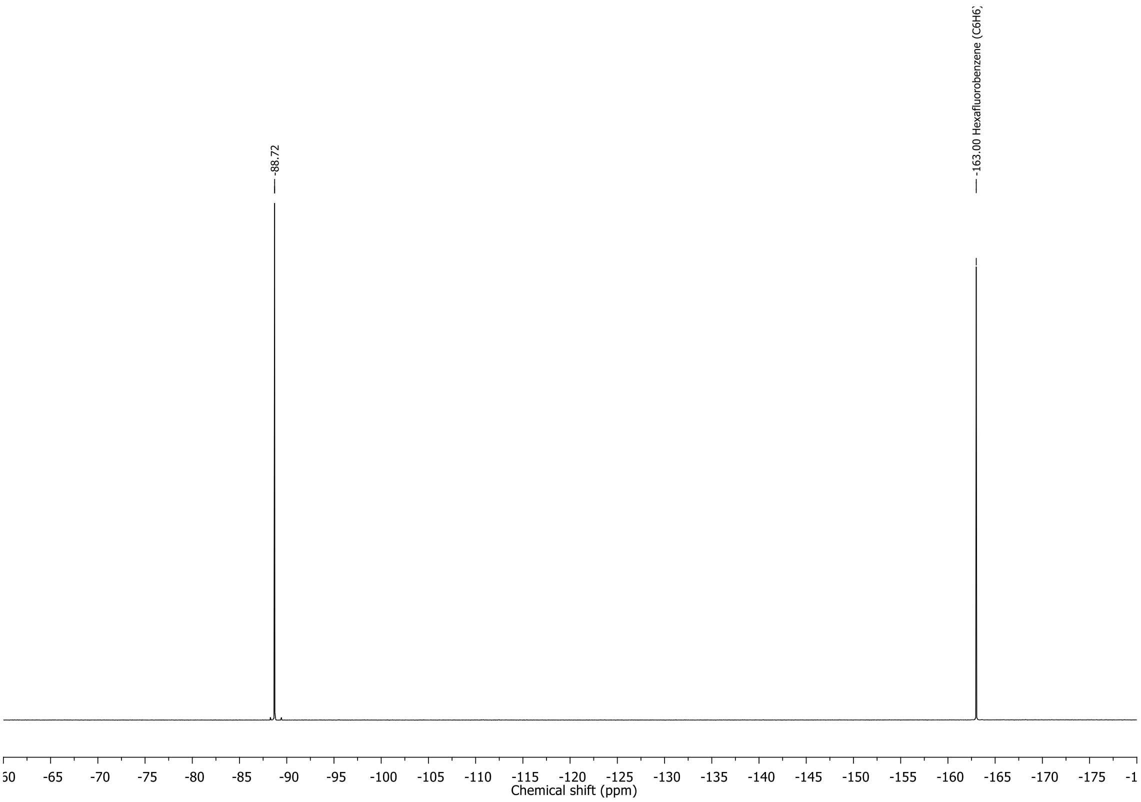

1-(Difluoromethoxy)-6-nitro-1*H*-benzo[*d*][1,2,3]triazole (3f′c)

^{1}H NMR ($CDCl_3$, 250 MHz)

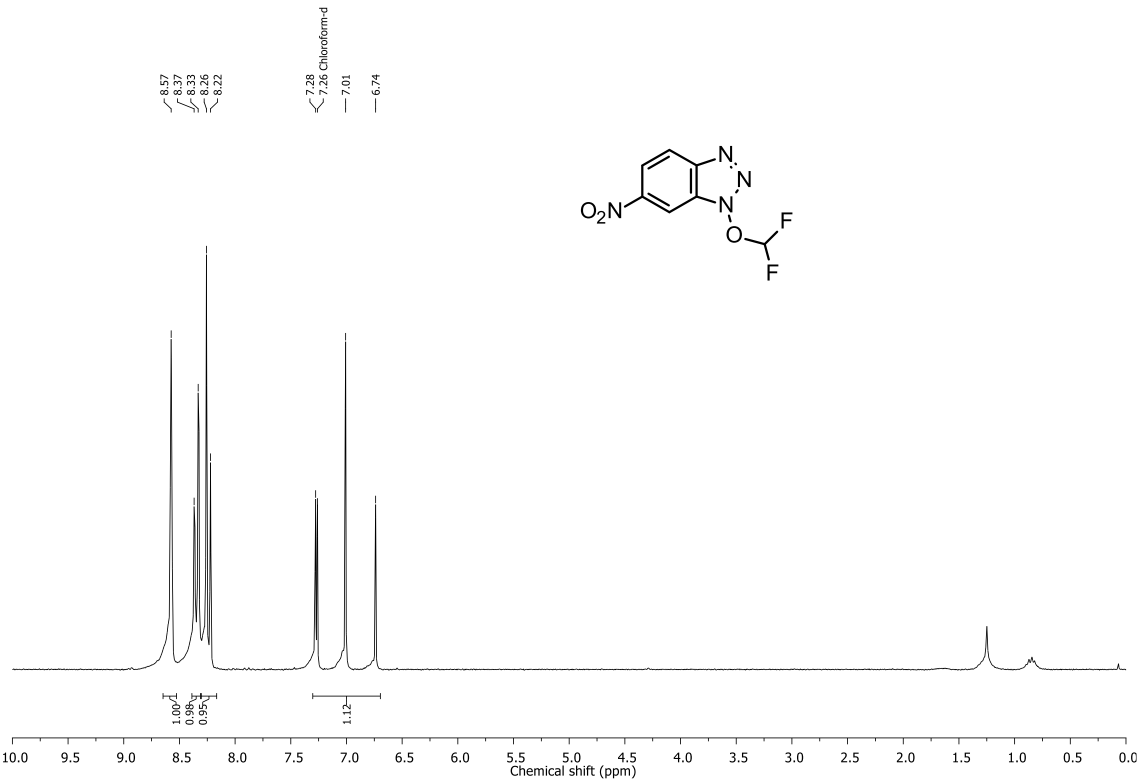

^{13}C NMR ($CDCl_3$, 63 MHz)

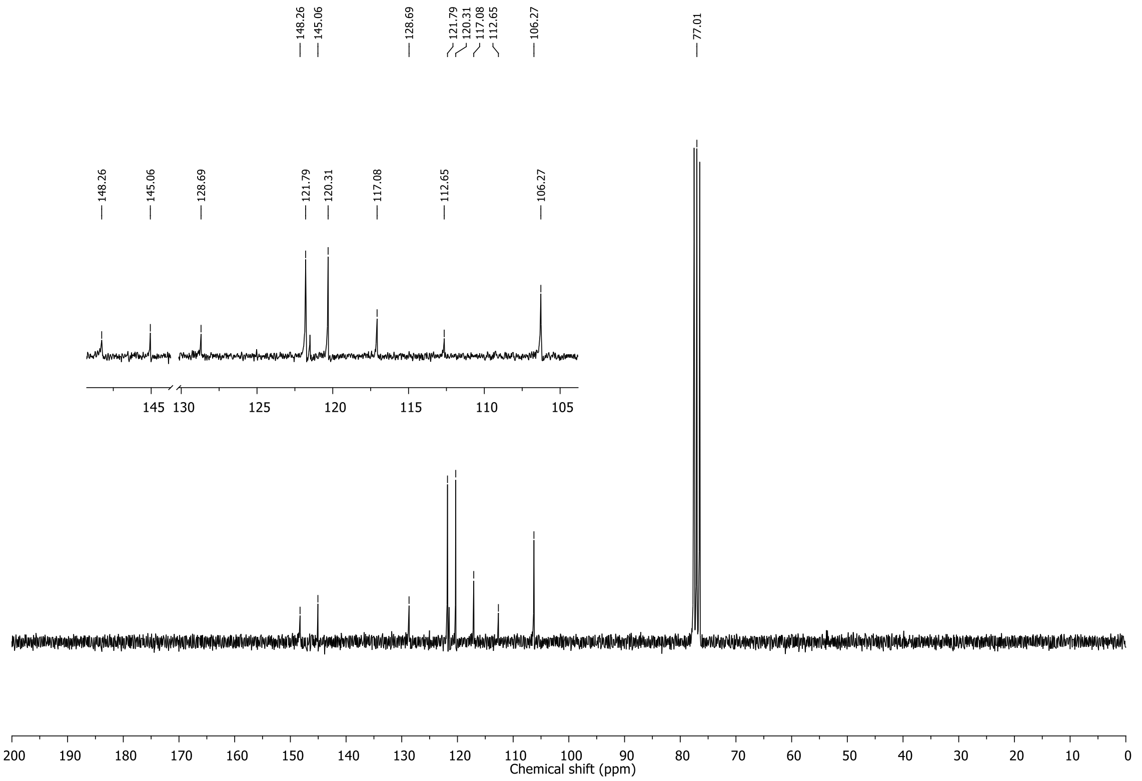

^{19}F NMR ($CDCl_3$, 235 MHz)

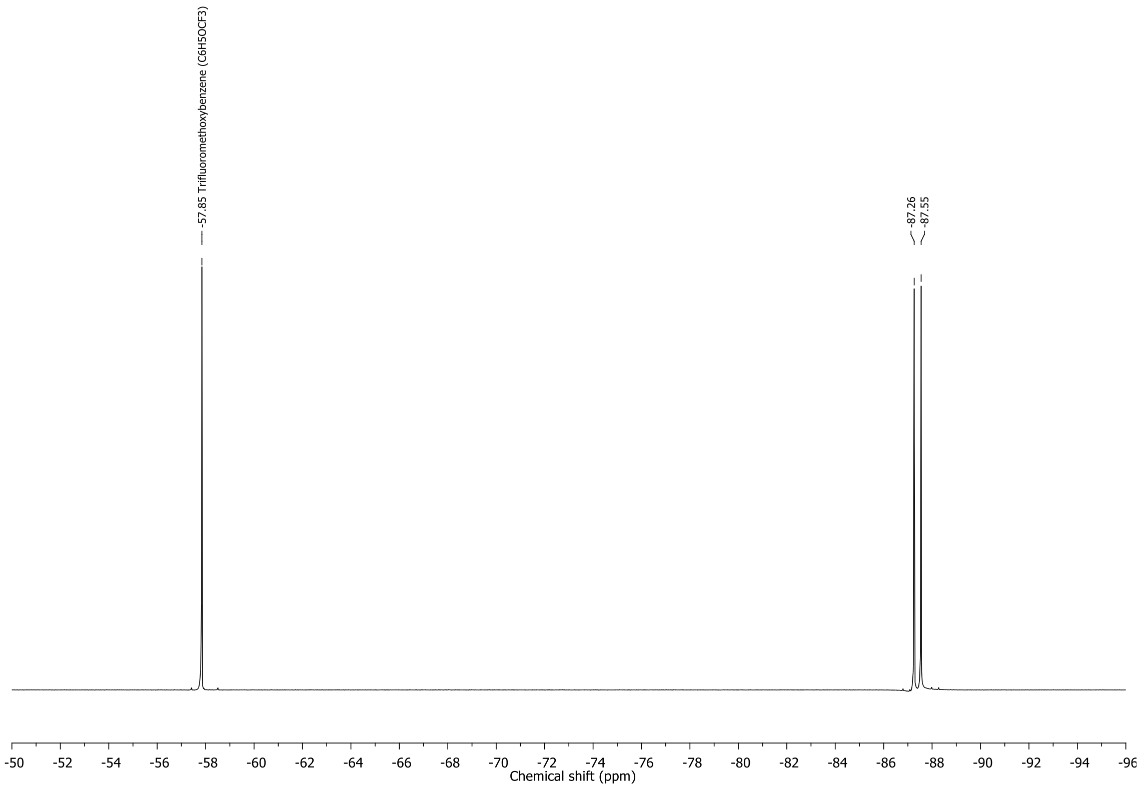

4-Chloro-1-(difluoromethoxy)-6-(trifluoromethyl)-1*H*-benzo[*d*][1,2,3]triazole (3f′d)

^{1}H NMR ($CDCl_3$, 250 MHz)

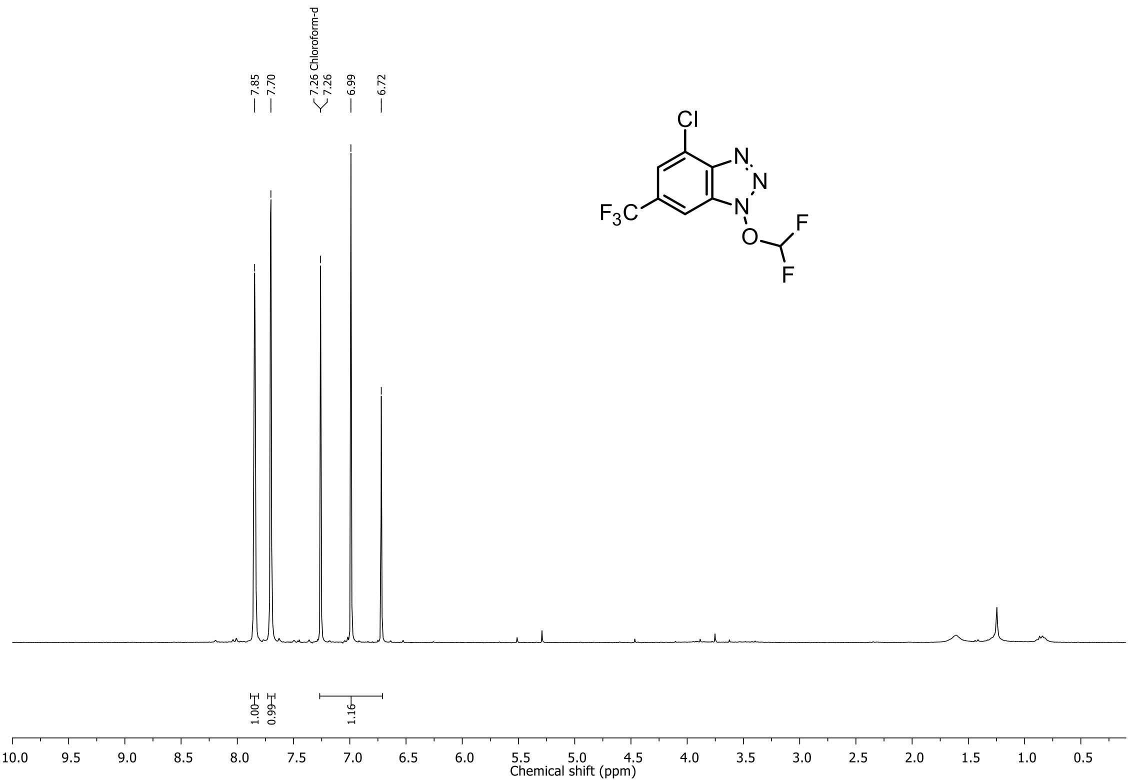

^{13}C NMR ($CDCl_3$, 63 MHz)

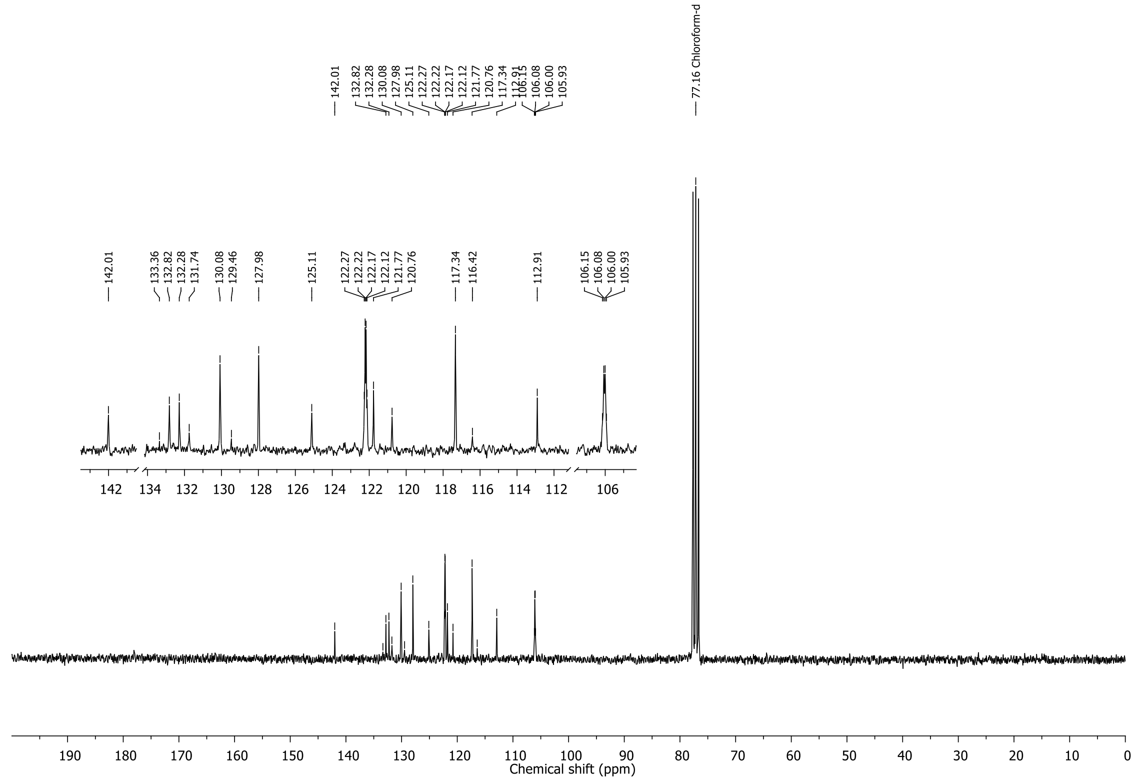

^{19}F NMR ($CDCl_3$, 235 MHz)

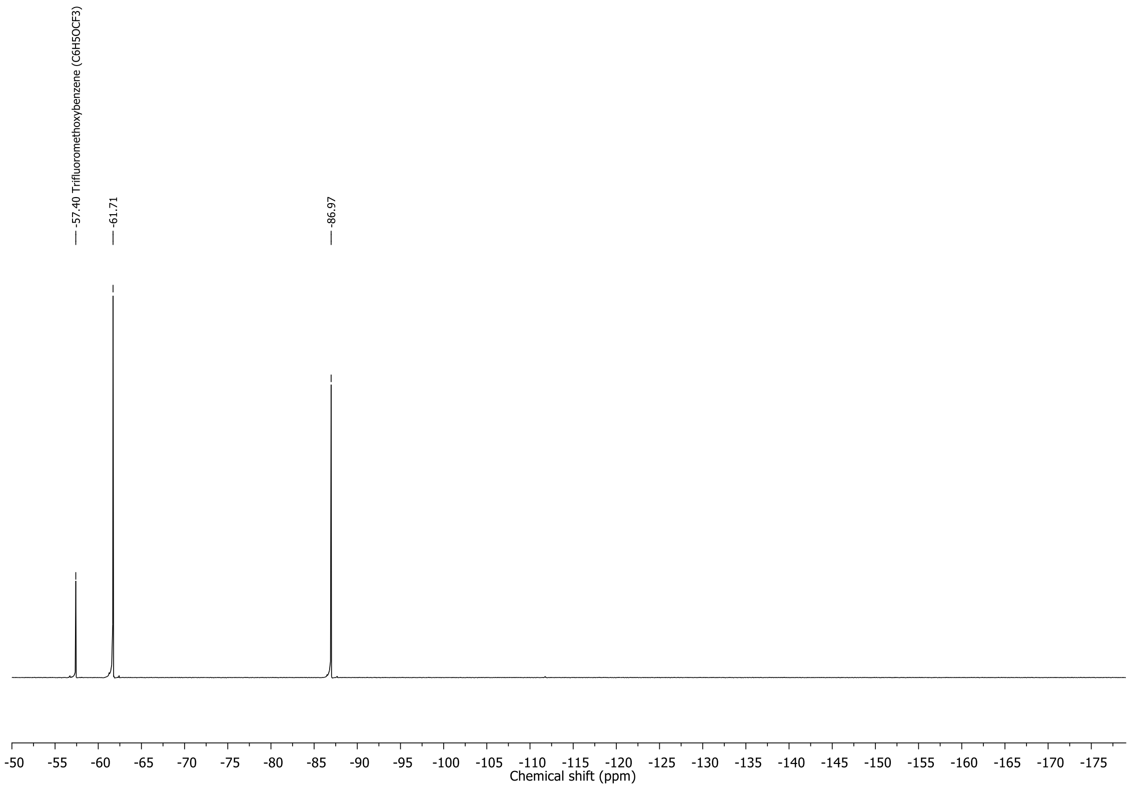

2-Phenylethanol (5a[4]) [CAS: 60-12-8]

^{1}H NMR ($CDCl_3$, 300 MHz)

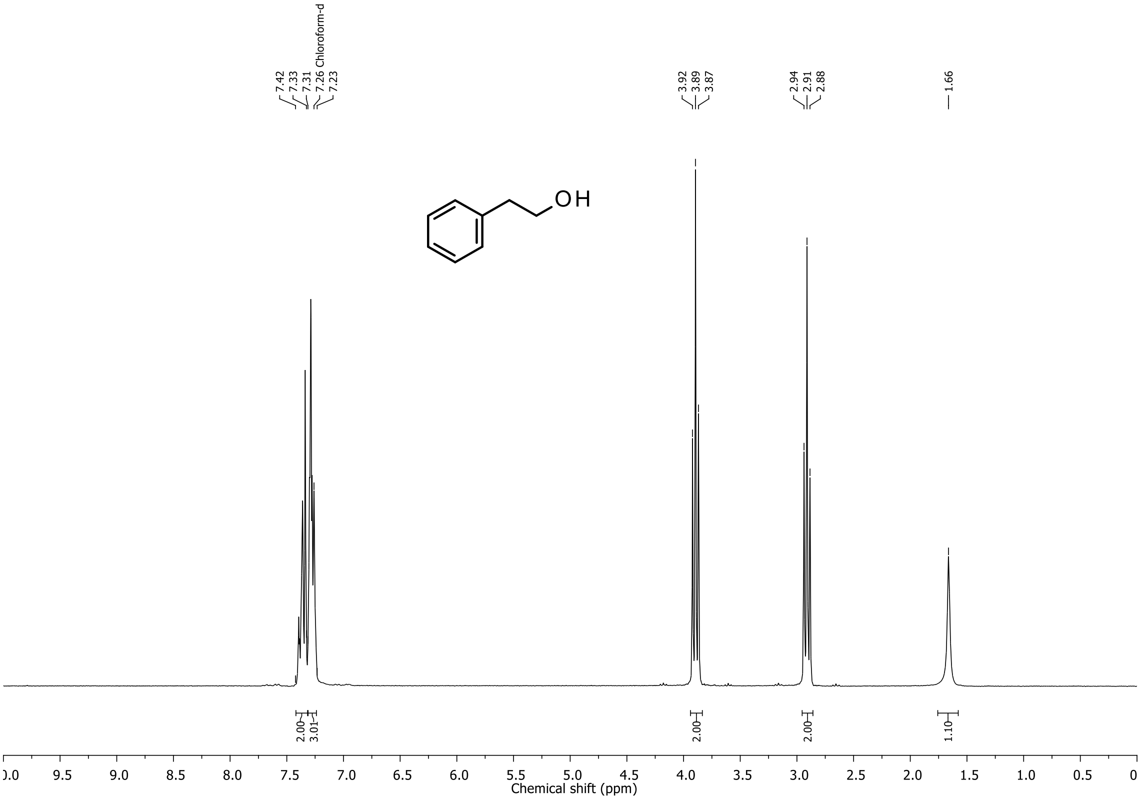

^{13}C NMR ($CDCl_3$, 63 MHz)

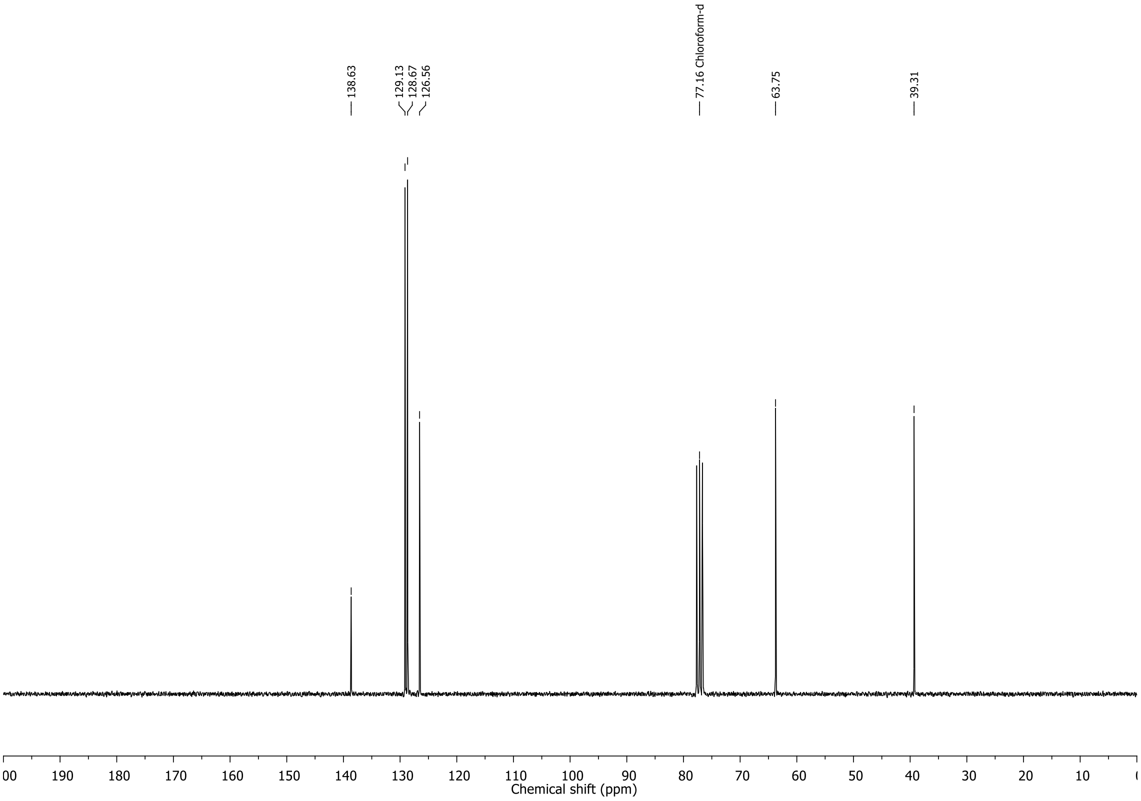

3-Methyl-1-phenethoxy-1*H*-benzo[*d*][1,2,3]triazol-3-ium trifluoromethanesulfonate (12aa)

^{1}H NMR (DMSO-d6, 300 MHz)

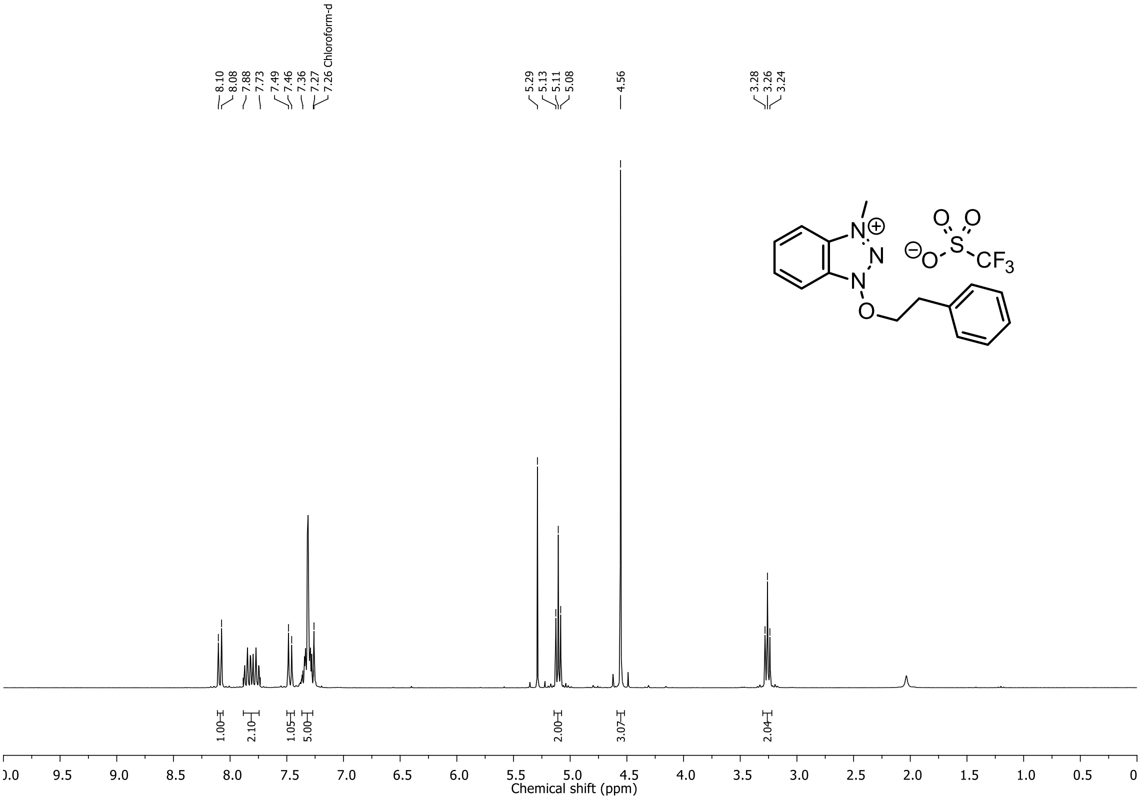

^{13}C NMR (DMSO-d6, 63 MHz)

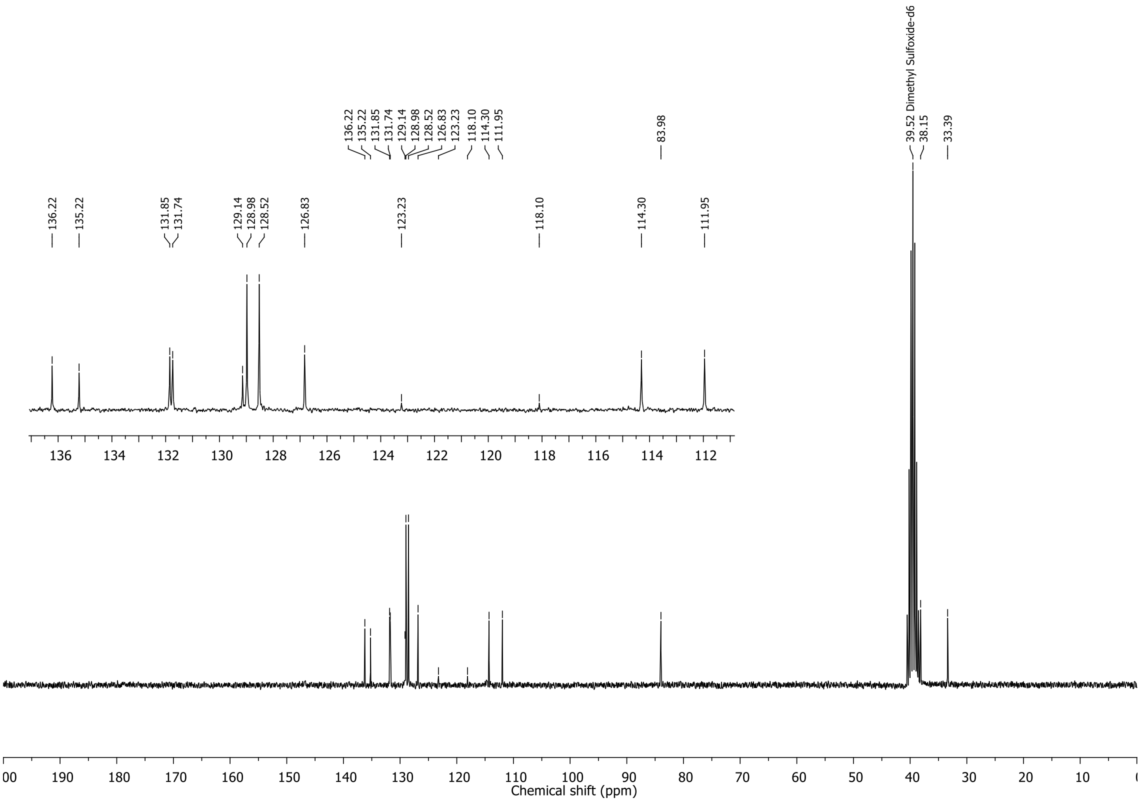

^{19}F NMR (DMSO-d_6, 235 MHz)

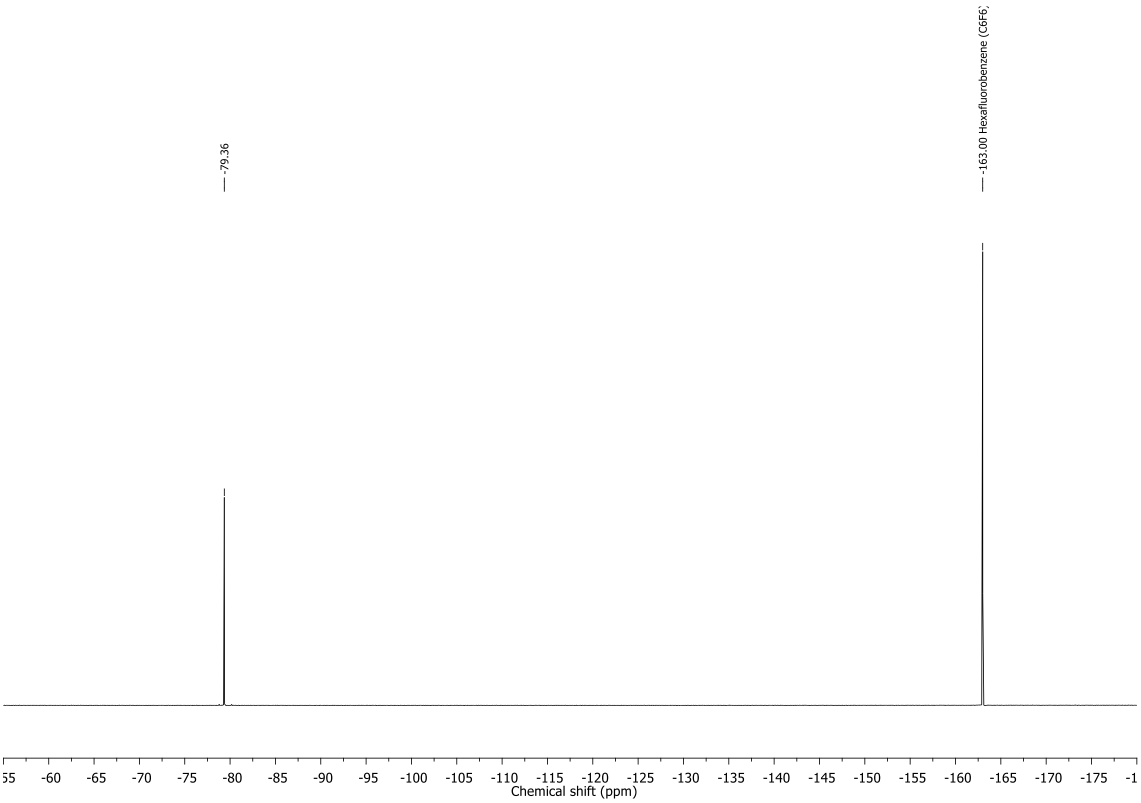

(2-Iodoethyl)benzene (13a) [CAS: 17376-04-4]

^{1}H NMR ($CDCl_3$, 250 MHz)

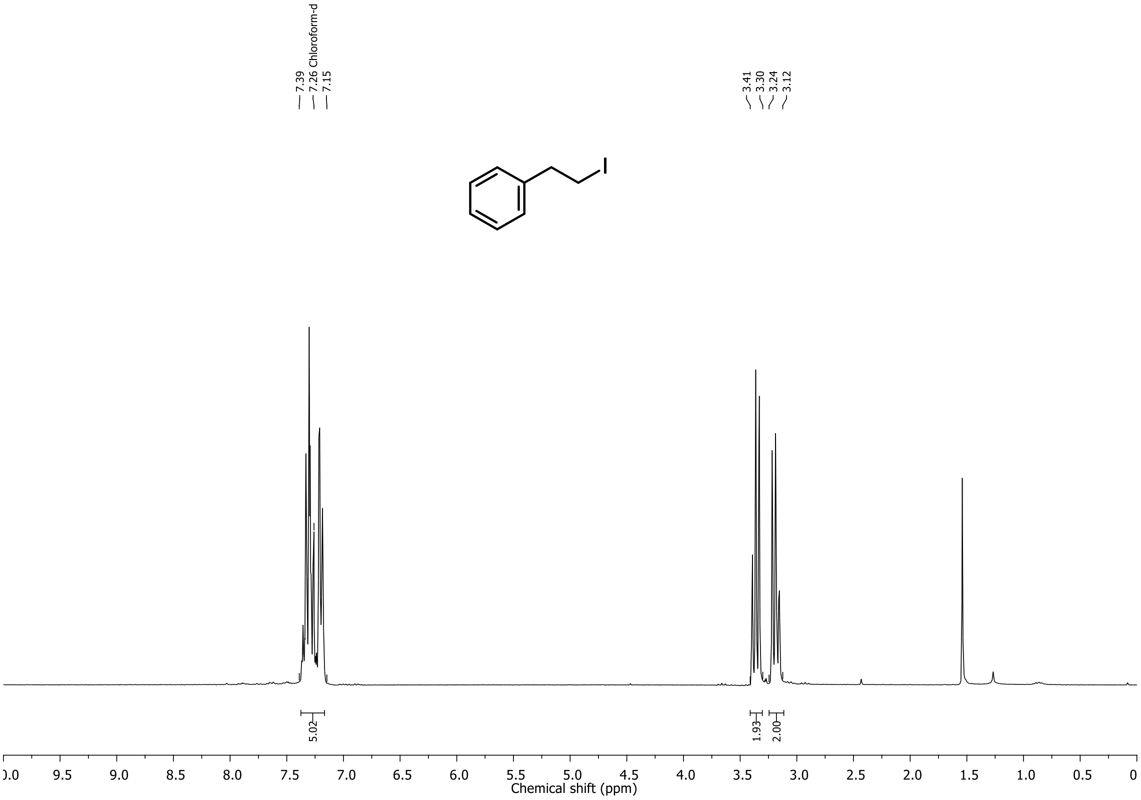

^{13}C NMR ($CDCl_3$, 63 MHz)

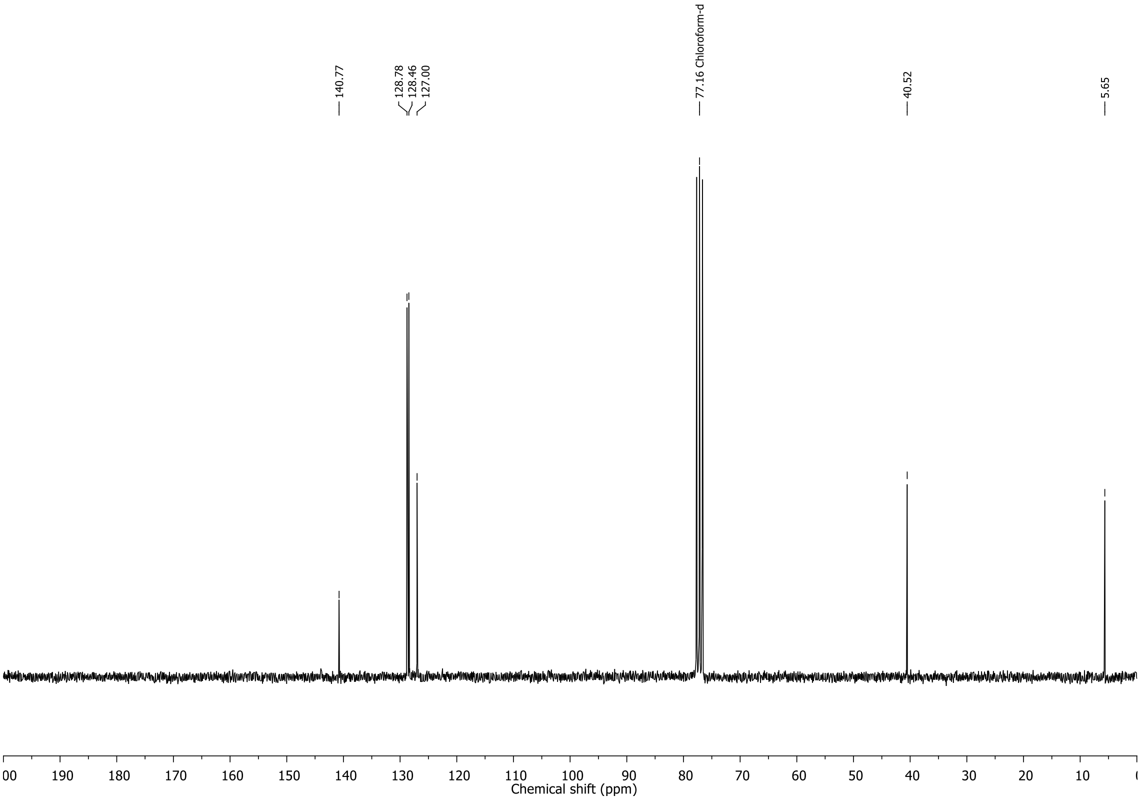

(2-Azidoethyl)benzene (14a) [CAS: 6926-44-9]

^{1}H NMR ($CDCl_3$, 250 MHz)

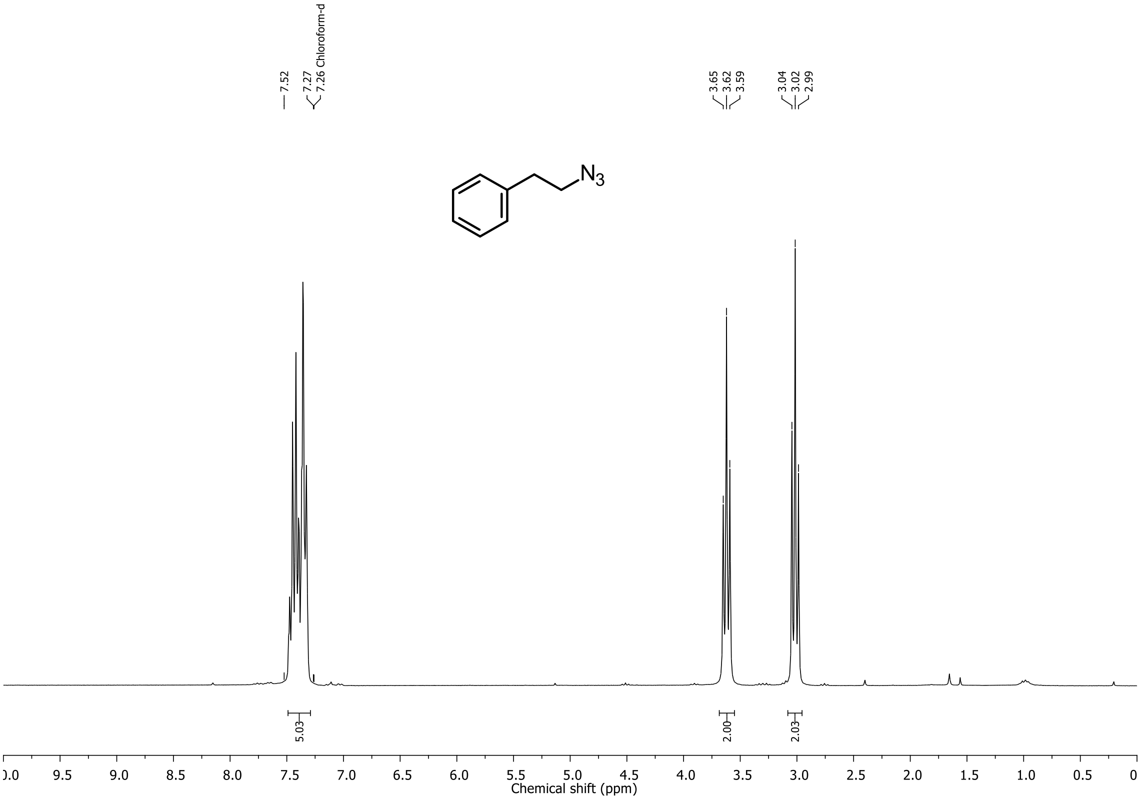

^{13}C NMR ($CDCl_3$, 63 MHz)

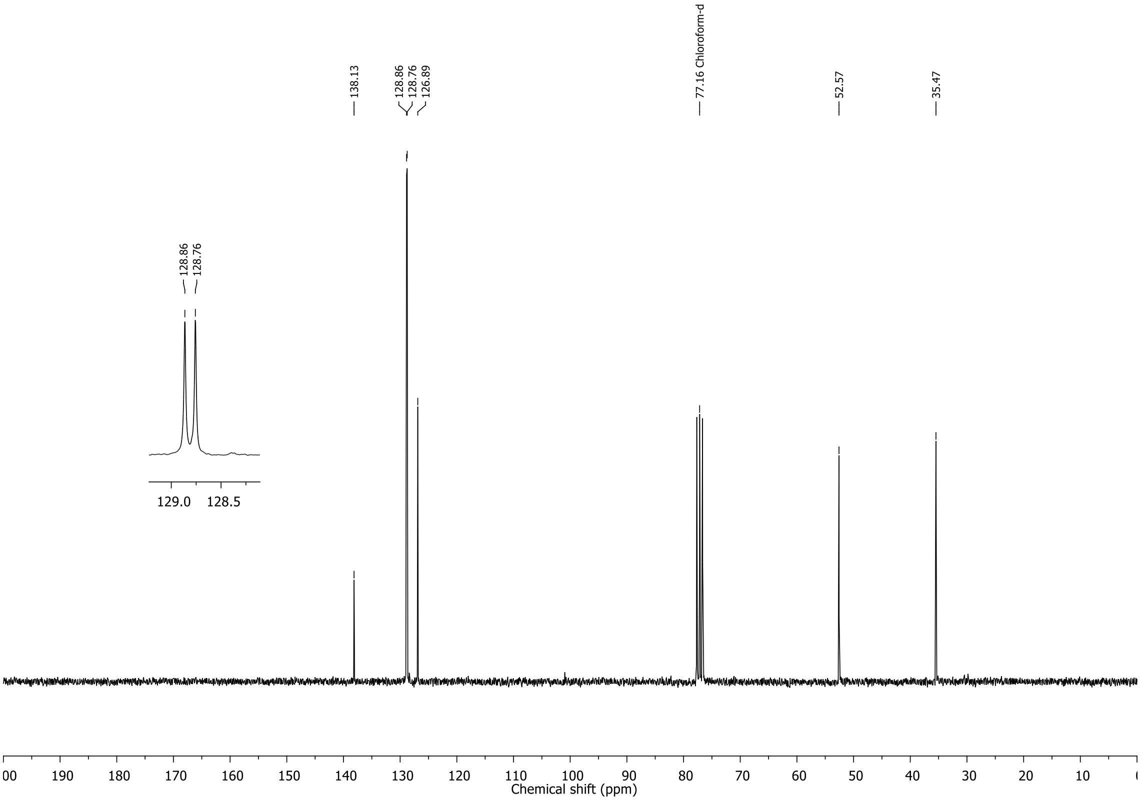

(2-Thiocyanatoethyl)benzene (15a) [CAS: 5654-72-8]

^{1}H NMR ($CDCl_3$, 250 MHz)

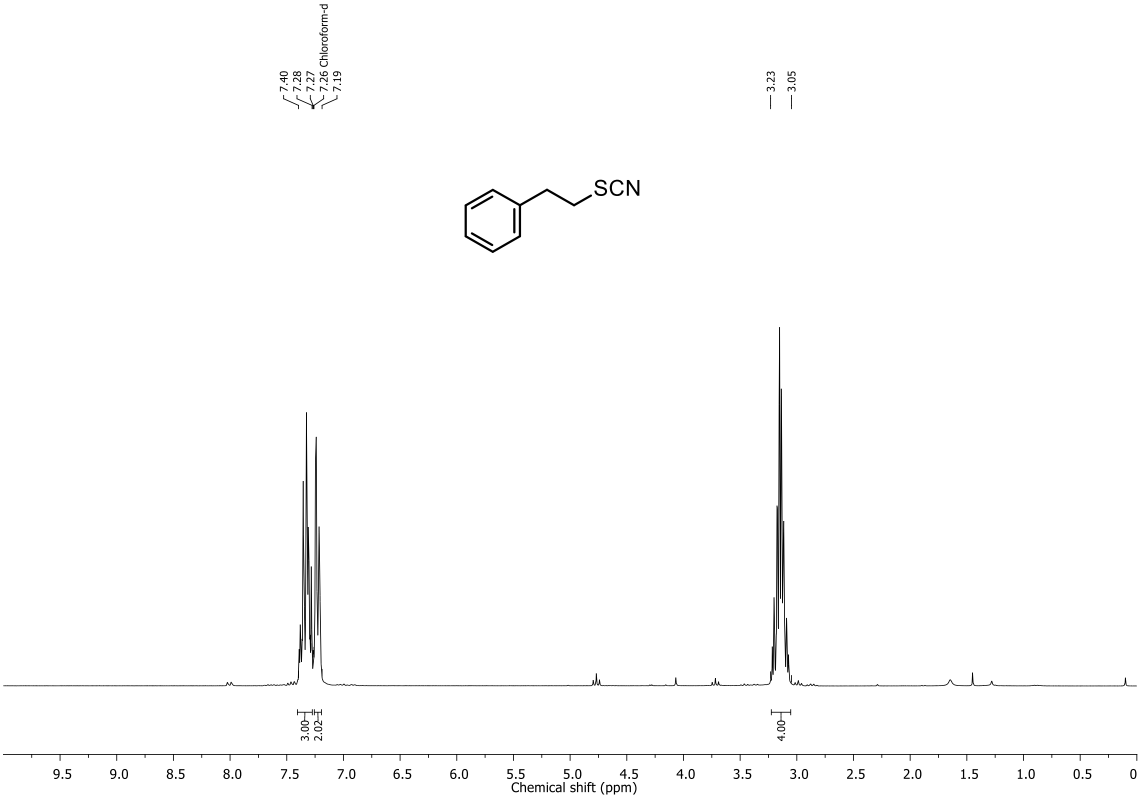

^{13}C NMR ($CDCl_3$, 63 MHz)

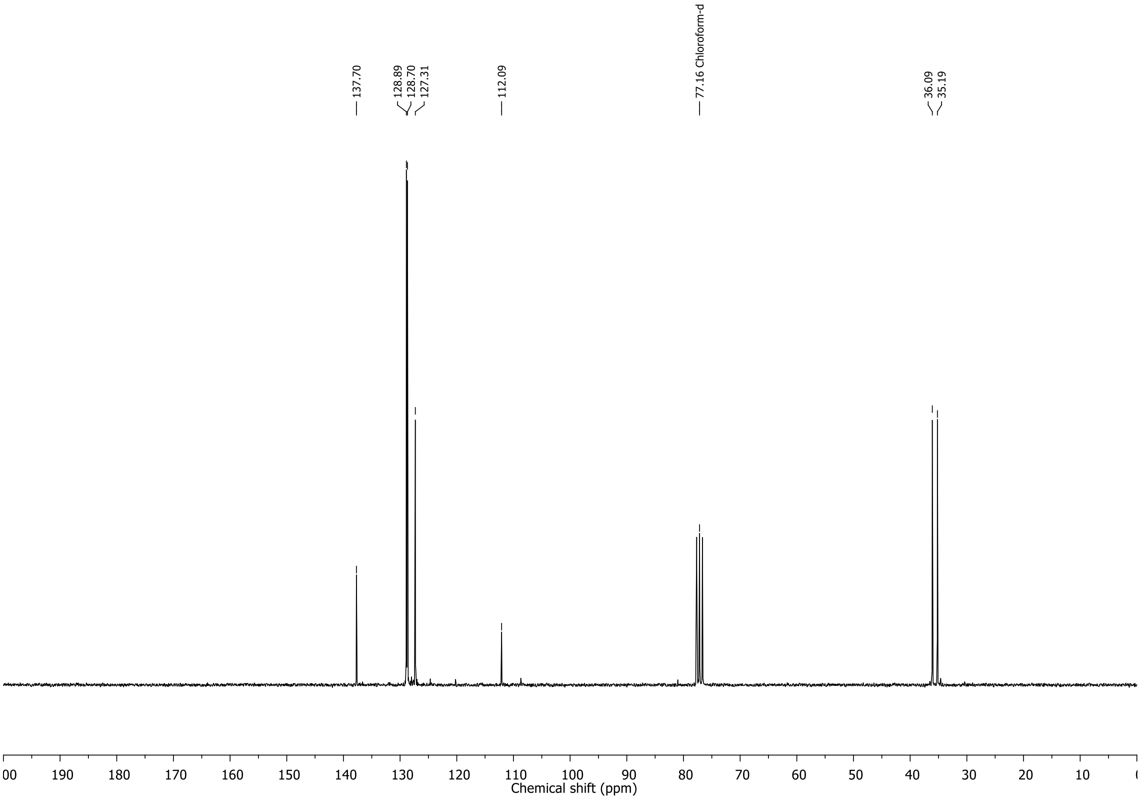

(2-Selenocyanatoethyl)benzene (16a) [CAS: 2218515-35-4]

^{1}H NMR ($CDCl_3$, 250 MHz)

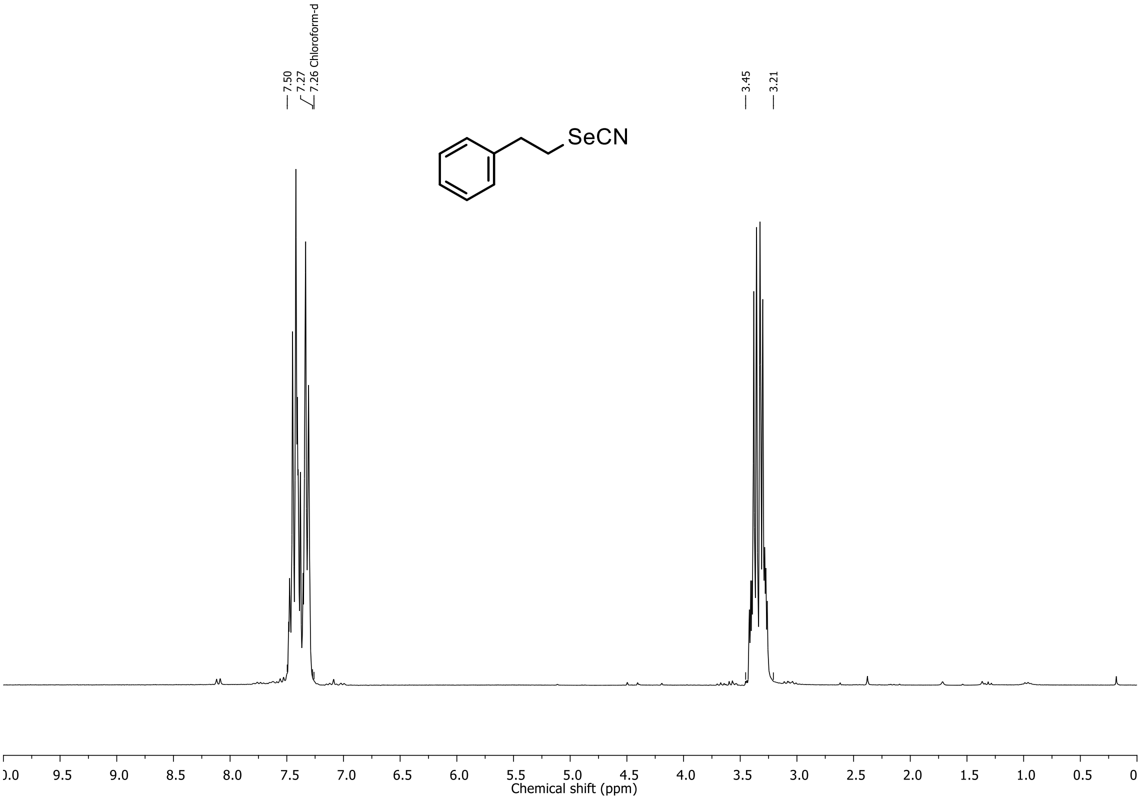

^{13}C NMR ($CDCl_3$, 63 MHz)

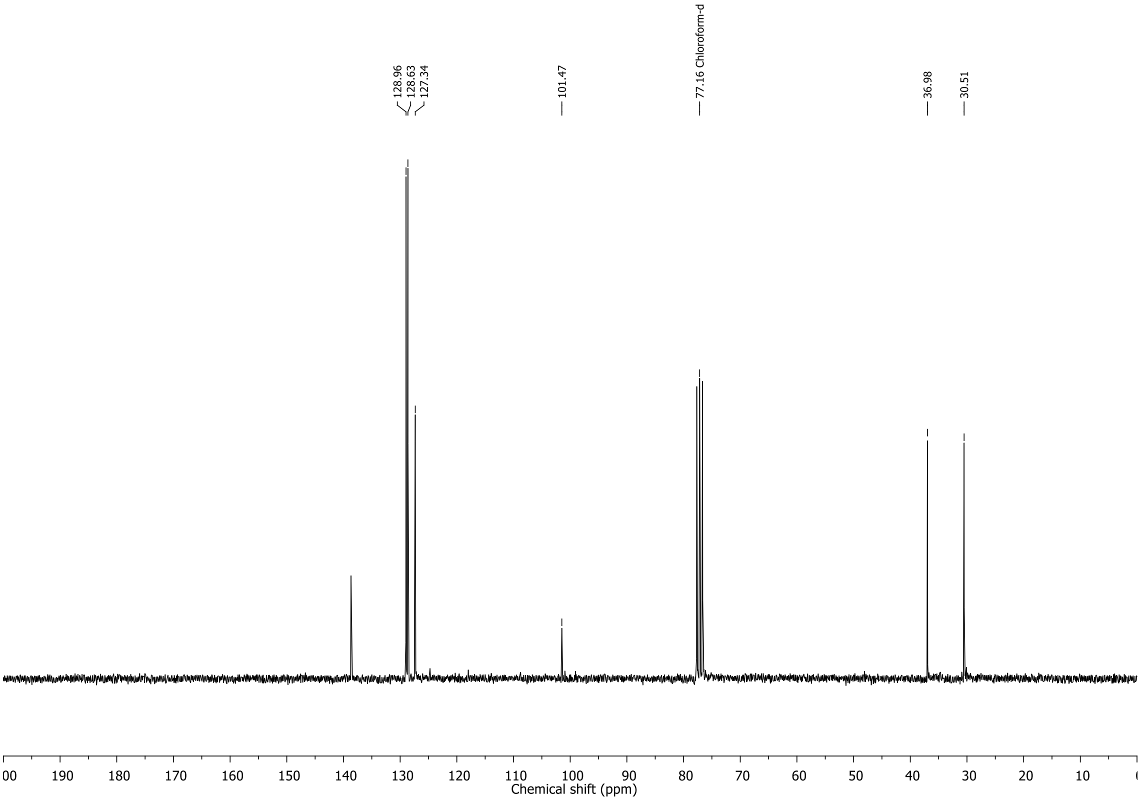

_O_-Ethyl _S_-phenethyl carbonodithioate (17a) [CAS: 3278-36-2]

^{1}H NMR ($CDCl_3$, 300 MHz)

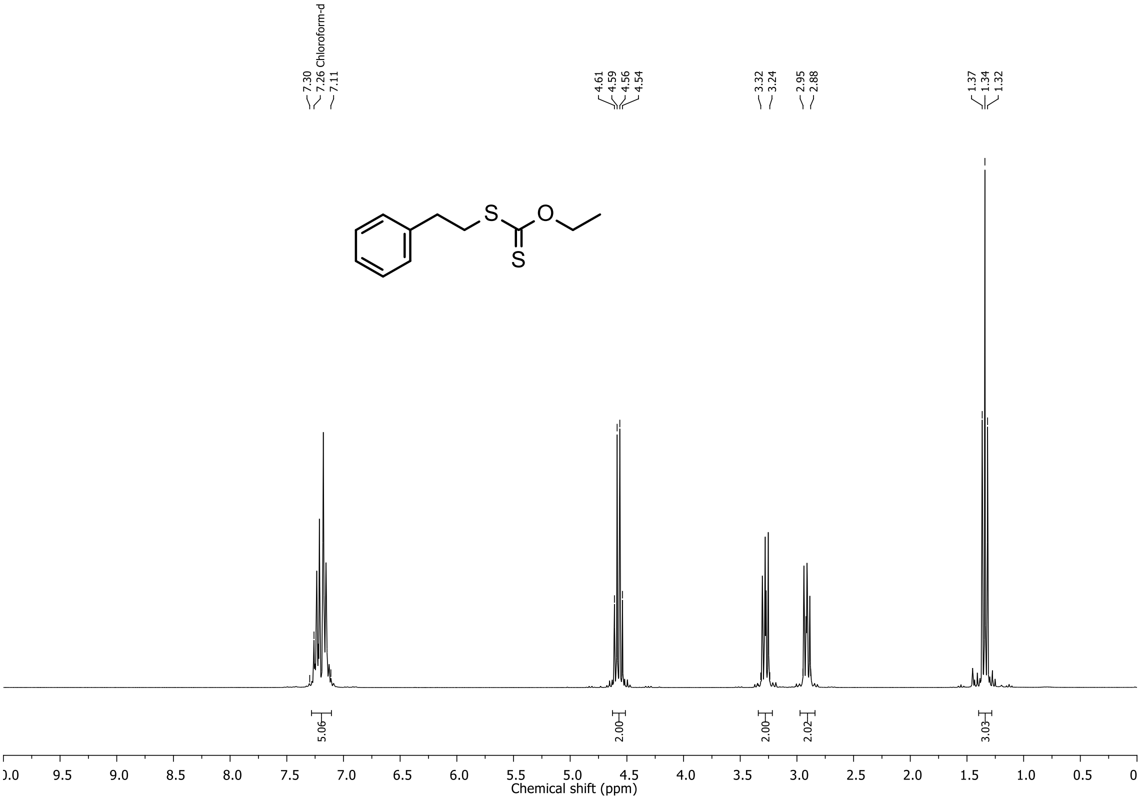

^{13}C NMR ($CDCl_3$, 63 MHz)

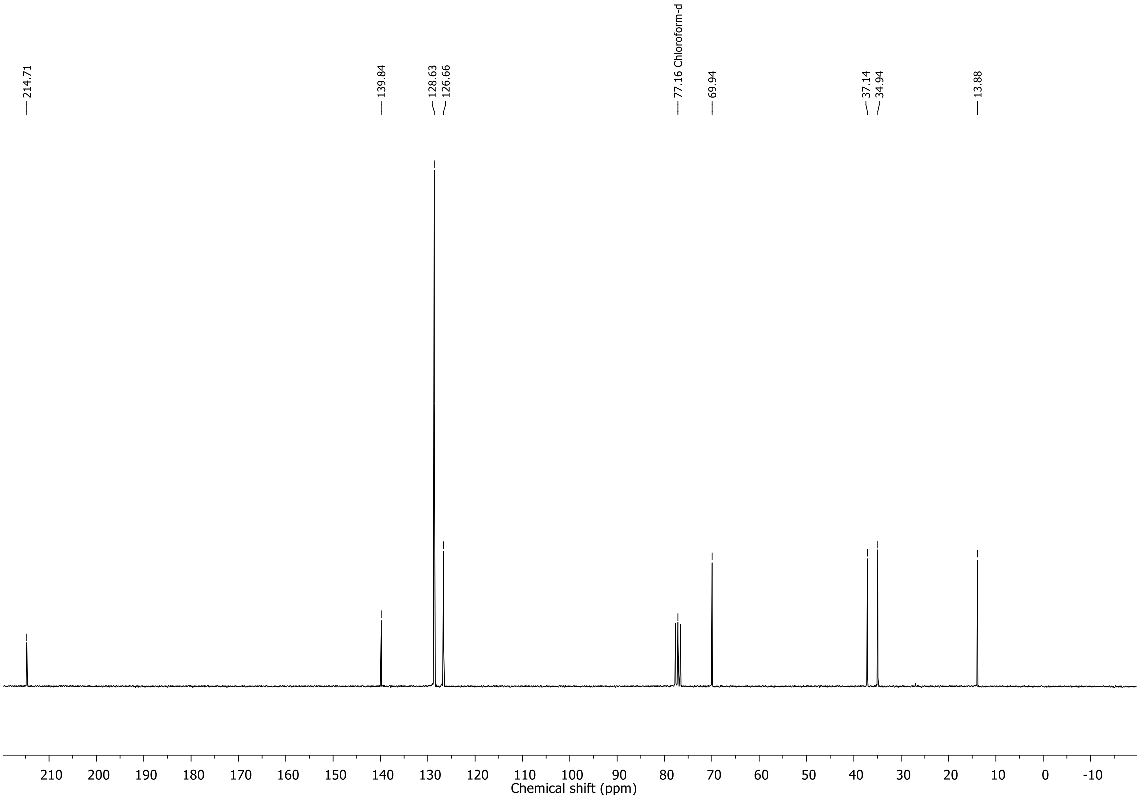

Phenethyl benzoate (18a) [CAS: 94-47-3]

^{1}H NMR ($CDCl_3$, 250 MHz)

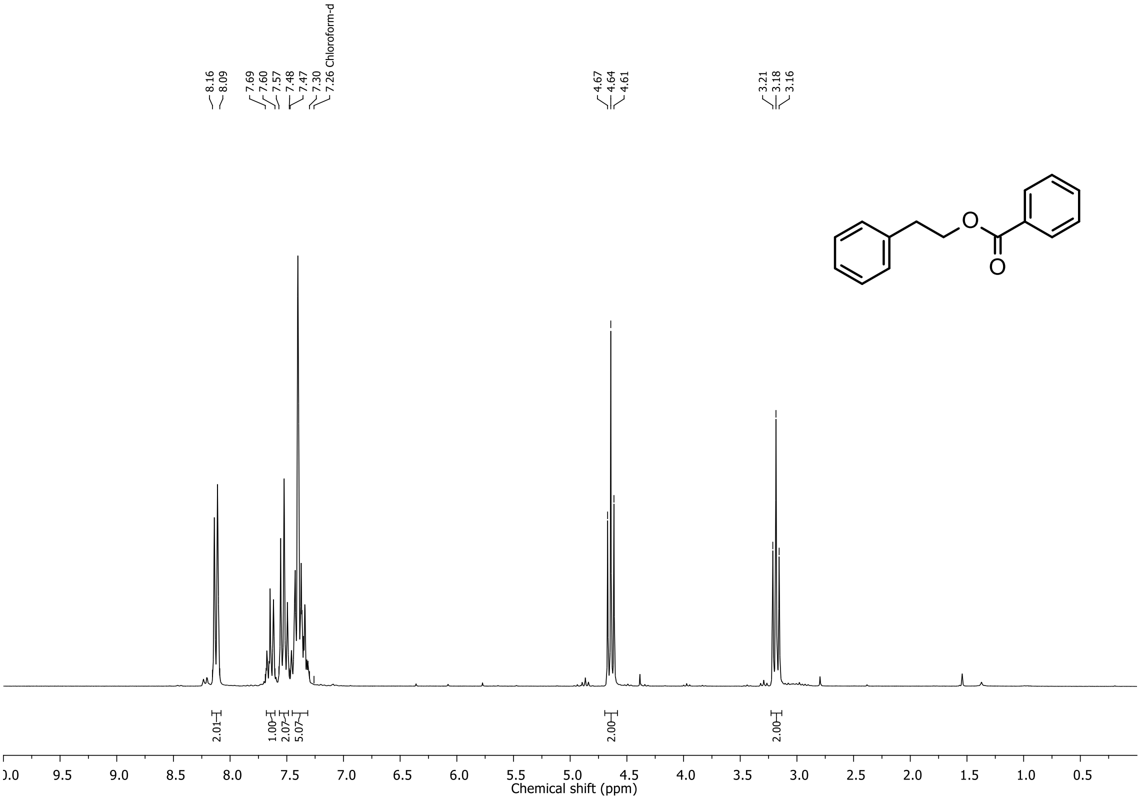

^{13}C NMR ($CDCl_3$, 63 MHz)

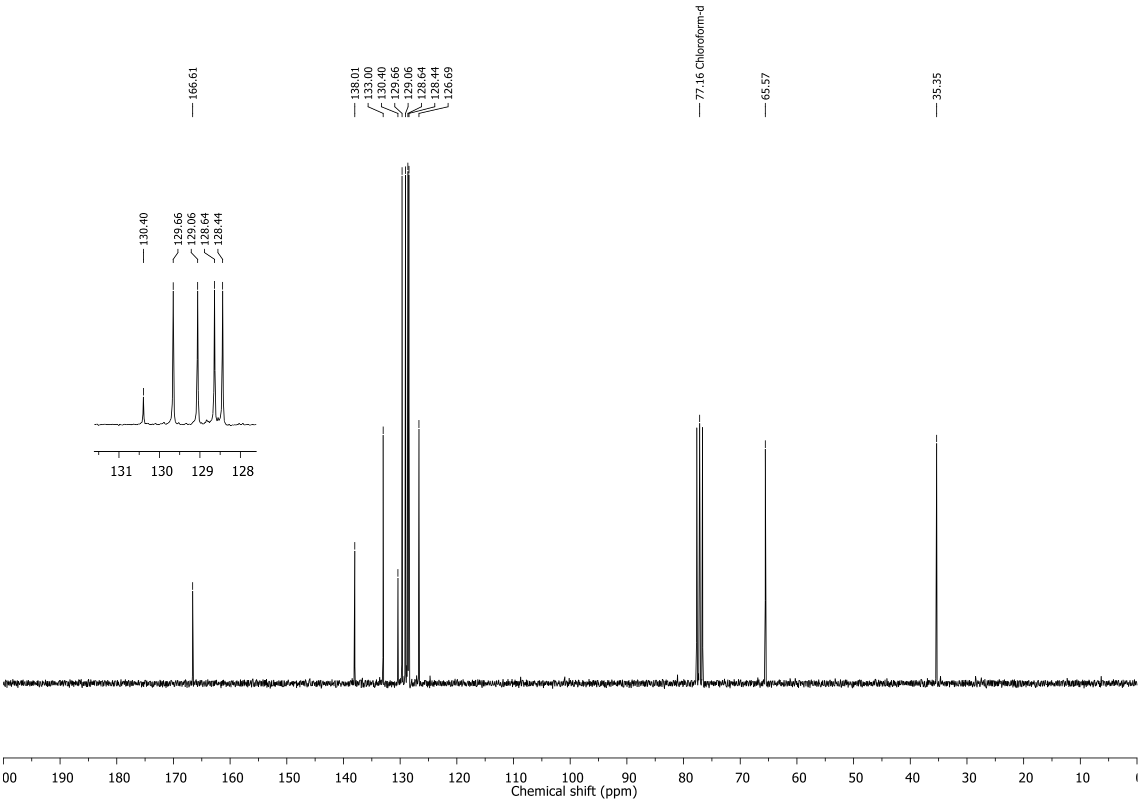

***N*-Phenethylaniline (19a)** [CAS: 1739-00-0]

^{1}H NMR ($CDCl_3$, 250 MHz)

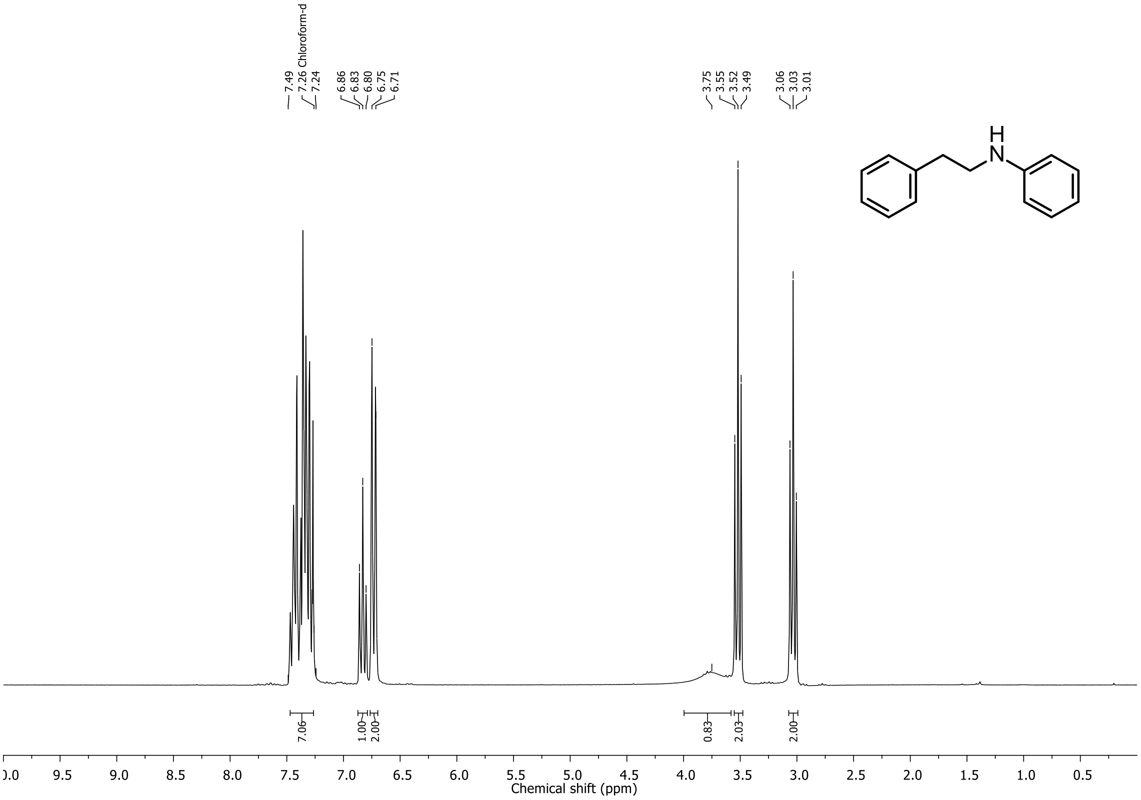

^{13}C NMR ($CDCl_3$, 63 MHz)

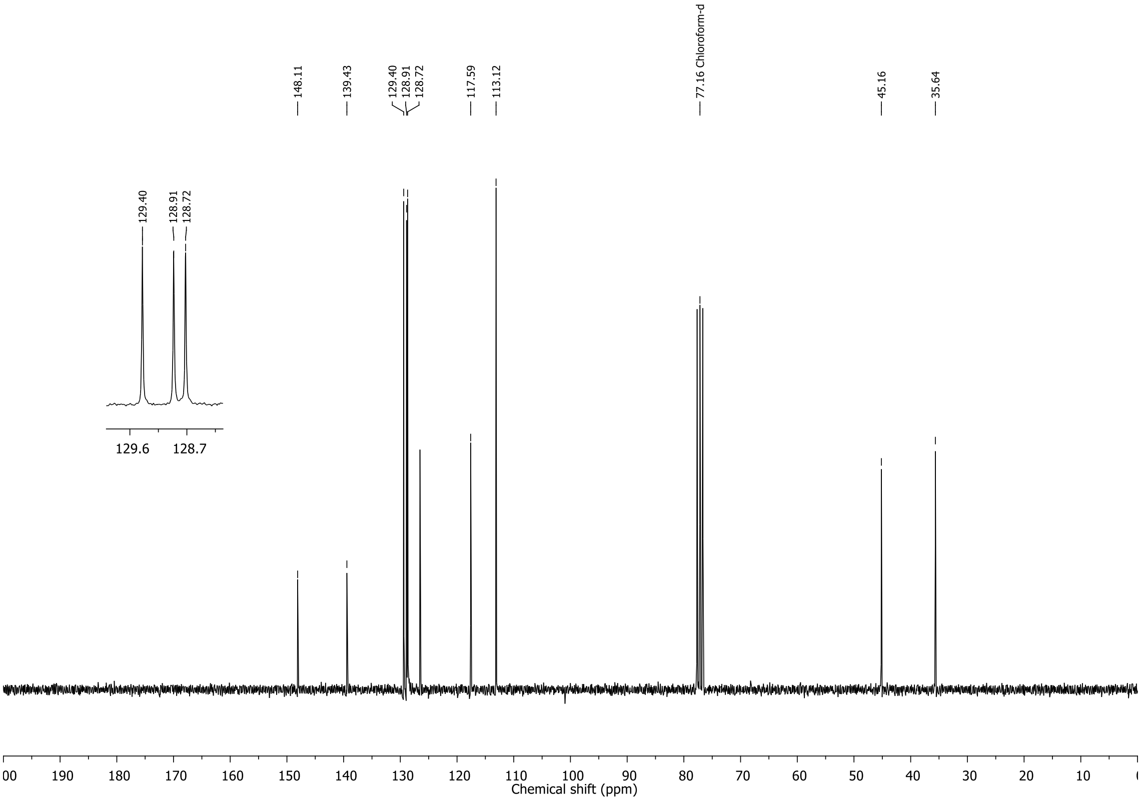

4-Phenethylmorpholine (20a) [CAS: 46346-12-7]

^{1}H NMR ($CDCl_3$, 250 MHz)

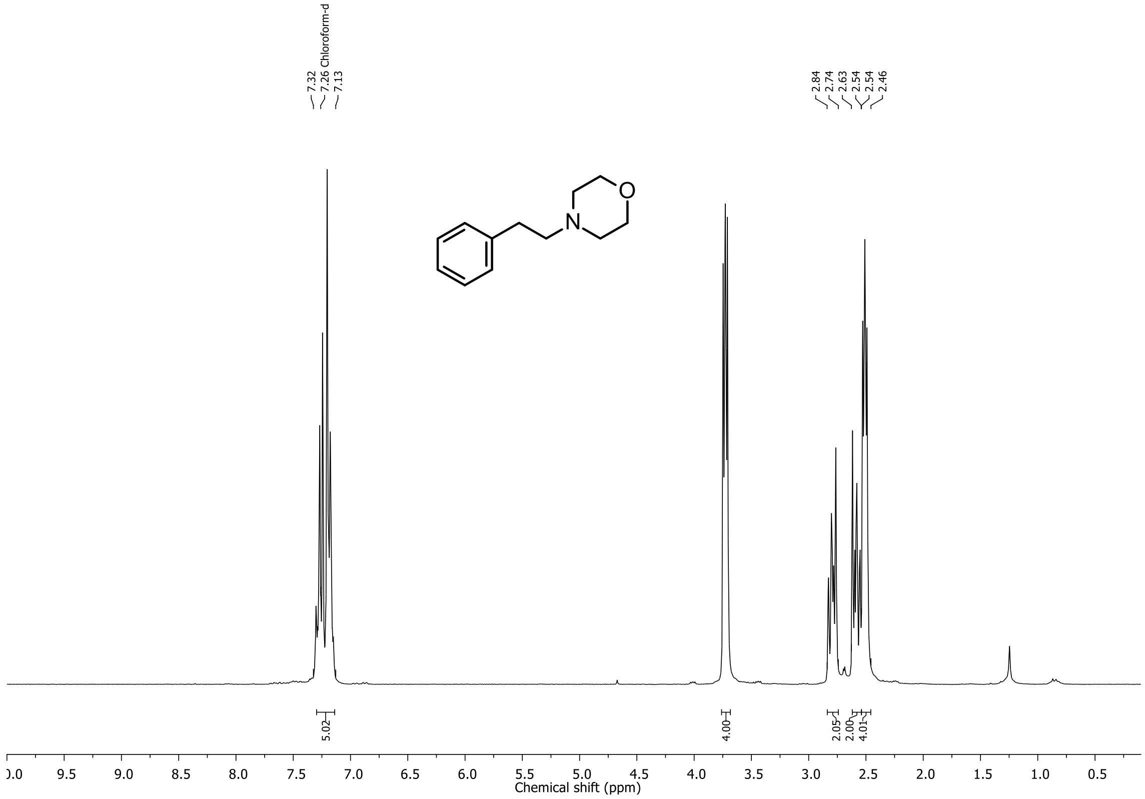

^{13}C NMR ($CDCl_3$, 63 MHz)

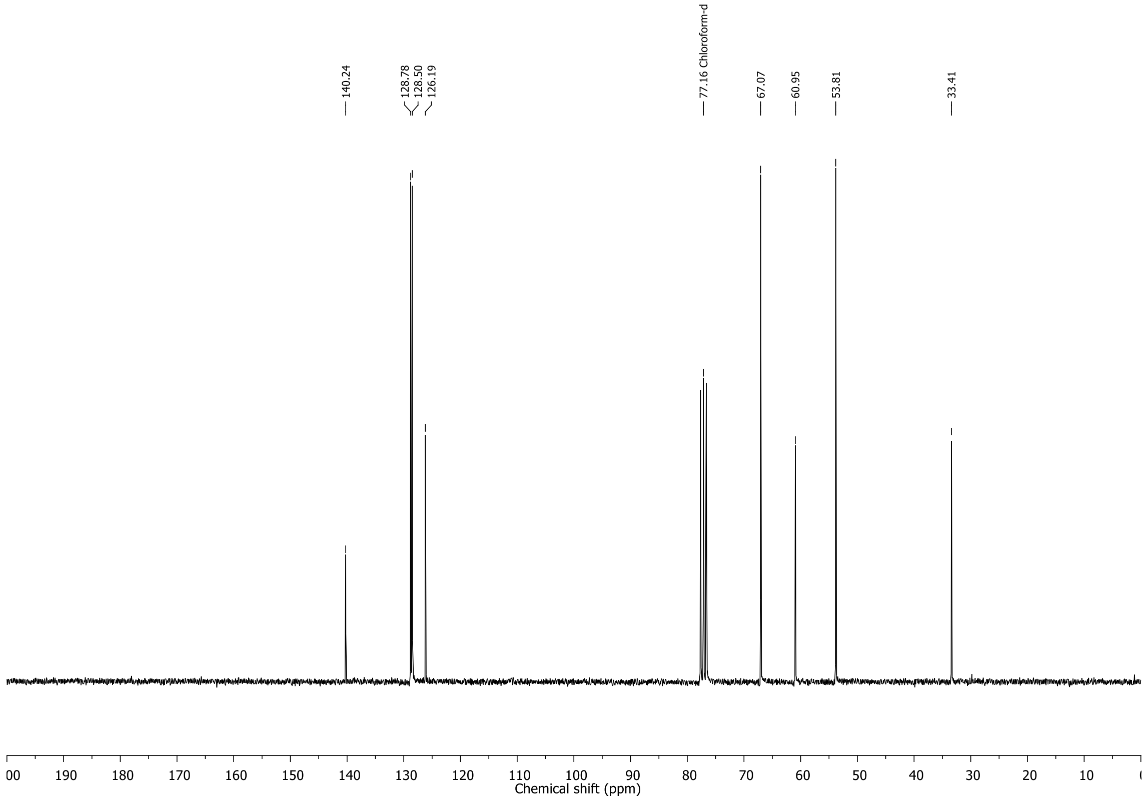

2,2,6,6-tetramethyl-1-phenethoxypiperidine (3a-OTEMP) [CAS: 131428-11-0]

^{1}H NMR ($CDCl_3$, 250 MHz)

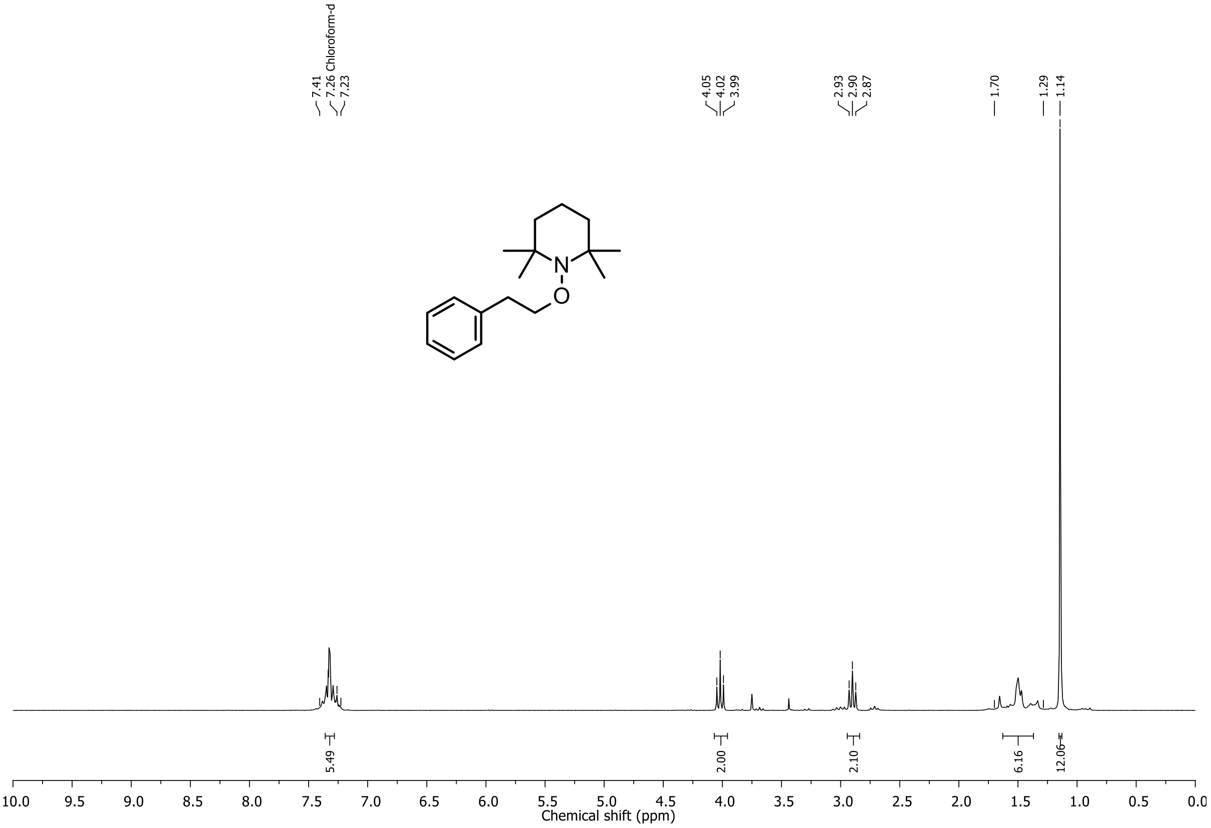

^{13}C NMR ($CDCl_3$, 63 MHz)

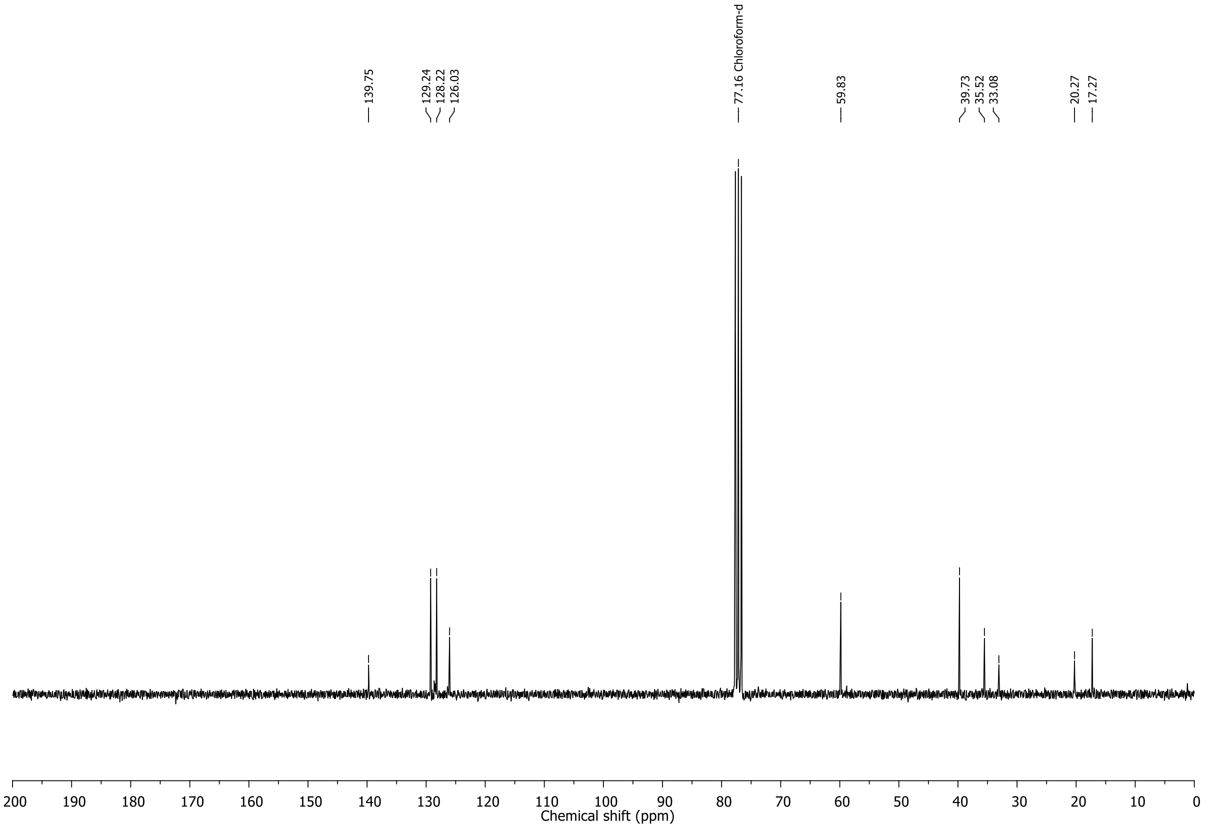

Supplementary References

1. Armarego, W. L. F. *Purification of laboratory chemicals*. (Elsevier, 2017).
2. Fichter, Fr. & Bloch, E. Elektrolysen von Salzen zweibasischer organischer Säuren (Bernsteinsäure, Glutarsäure, Brenzweinsäure, äthyl-malonsäure) mit Nitraten. *Helv. Chim. Acta* **22**, 1529–1540 (1939).
3. Fichter, F. R. & Gunst, R. Elektrolysen von Mischungen von Pivalinaten mit Nitraten. *Helv. Chim. Acta* **22**, 1300–1307 (1939).
4. Fichter, Fr. & Leupin, O. Elektrolyse von Salzen der Önanthsäure für sich oder im Gemisch mit Nitraten. *Helv. Chim. Acta* **21**, 616–625 (1938).
5. Fichter, Fr. & Metz, F. Elektrolyse von Nitrat-Acetat-Gemischen. *Helv. Chim. Acta* **18**, 1005–1007 (1935).
6. Fichter, Fr. & Metz, F. Elektrolyse von Mischungen von n-Butyraten mit Nitraten. *Helv. Chim. Acta* **19**, 597–606 (1936).
7. Fichter, Fr. & Metz, F. Elektrolysen von Mischungen von Isovalerianaten und Nitraten. *Helv. Chim. Acta* **19**, 880–890 (1936).
8. Fichter, Fr. & Ruegg, R. Elektrolysen von Gemischen fettsaurer Salze mit Halogeniden und Nitraten. *Helv. Chim. Acta* **20**, 1578–1590 (1937).
9. Fichter, F. & Stenzl, H. Die Kolbe'sche Elektrosynthese mit aromatischen Säuren: Benzoesäure, Phenyl-essigsäure, β-Phenyl-propionsäure, Phenoxy-essigsäure. *Helv. Chim. Acta* **22**, 970–978 (1939).
10. Fichter, Fr. & Sutter, P. Elektrolysen von Mischungen von Isobutyraten mit Nitraten. *Helv. Chim. Acta* **21**, 891–900 (1938).
11. Fichter, Fr. & Sutter, P. Elektrolysen von Mischungen von Nitraten mit den Salzen der Methyl-äthyl-essigsäure. *Helv. Chim. Acta* **21**, 1401–1407 (1938).
12. Fichter, Fr., Siegrist, W. & Buess, H. Elektrolysen von Mischungen von Propionaten und Nitraten. *Helv. Chim. Acta* **18**, 18–25 (1935).
13. Rubio-Presa, R., Fernández-Rodríguez, M. A., Pedrosa, M. R., Arnáiz, F. J. & Sanz, R. Molybdenum-Catalyzed Deoxygenation of Heteroaromatic *N*- Oxides and Hydroxides using Pinacol as Reducing Agent. *Adv. Synth. Catal.* **359**, 1752–1757 (2017).

14. Khattab, S. N., Hassan, S. Y., Hamed, E. A., Albericio, F. & El-Faham, A. Synthesis and Aminolysis of N,N-Diethyl Carbamic Ester of HOBt Derivatives. *Bull. Korean Chem. Soc.* **31**, 75–81 (2010).
15. Crépin, A. *et al.* Aminoacid-derived mercaptoimidazoles. *Org Biomol Chem* **7**, 128–134 (2009).
16. von Wantoch Rekowski, M. *et al.* Insights into Soluble Guanylyl Cyclase Activation Derived from Improved Heme-Mimetics. *J. Med. Chem.* **56**, 8948–8952 (2013).
17. Kaname, M., Yoshifuji, S. & Sashida, H. Ruthenium tetroxide oxidation of cyclic *N*-acylamines by a single layer method: formation of ω-amino acids. *Tetrahedron Lett.* **49**, 2786–2788 (2008).
18. Lakshman, M. K. *et al.* Facile synthesis of 1-alkoxy-1*H*-benzo- and 7-azabenzotriazoles from peptide coupling agents, mechanistic studies, and synthetic applications. *Beilstein J. Org. Chem.* **10**, 1919–1932 (2014).
19. Feld, W. A., Paessun, R. J. & Serve, M. P. The Phase Transfer Catalyzed Alkylation of 1-Hydroxybenzotriazole. I. Scope. *J. Macromol. Sci. Part - Chem.* **15**, 891–896 (1981).
20. Chu, Y., Shan, Z., Liu, D. & Sun, N. Asymmetric Reduction of Oxime Ethers Promoted by Chiral Spiroborate Esters with an O_3BN Framework. *J. Org. Chem.* **71**, 3998–4001 (2006).
21. Andia, A. A., Miner, M. R. & Woerpel, K. A. Copper(I)-Catalyzed Oxidation of Alkenes Using Molecular Oxygen and Hydroxylamines: Synthesis and Reactivity of α-Oxygenated Ketones. *Org. Lett.* **17**, 2704–2707 (2015).
22. Zhao, Y. & Antonietti, M. Visible-Light-Driven Conversion of Alcohols into Iodide Derivatives with Iodoform. *ChemPhotoChem* **2**, 720–724 (2018).
23. Sebest, F. *et al.* Ring-Expanded *N*-Heterocyclic Carbenes for Copper-Mediated Azide-Alkyne Click Cycloaddition Reactions. *ChemCatChem* **10**, 2041–2045 (2018).
24. Kiasat, A. R., Zayadi, M. & Mehrjardi, M. F. Regioselective ring opening of epoxides using NH4SCN/silica sulfuric acid: An efficient approach for the synthesis of β-hydroxy thiocyanate under solvent-free conditions. *Chin. Chem. Lett.* **19**, 665–668 (2008).
25. Glenadel, Q., Ayad, C., D'Elia, M.-A., Billard, T. & Toulgoat, F. Nucleophilic trifluoromethylthiolation of organoselenocyanates with trifluoromethanesulfenamide reagent: Access to CF3SSe-containing compounds. *J. Fluor. Chem.* **210**, 112–116 (2018).
26. Le Minor, A., Kanjo, I. E. & Villemin, D. Synthesis of S-dithiocarbonates with polymer-supported xanthates. *Polym. Bull.* **21**, 445–448 (1989).

27. Chen, X. *et al.* Fe-catalyzed esterification of amides *via* C–N bond activation. *RSC Adv.* **8**, 4571–4576 (2018).

28. Shao, Z., Fu, S., Wei, M., Zhou, S. & Liu, Q. Mild and Selective Cobalt-Catalyzed Chemodivergent Transfer Hydrogenation of Nitriles. *Angew. Chem. Int. Ed.* **55**, 14653–14657 (2016).

29. Mitsudome, T. *et al.* Mild Hydrogenation of Amides to Amines over a Platinum-Vanadium Bimetallic Catalyst. *Angew. Chem. Int. Ed.* **56**, 9381–9385 (2017).

30. Patel, V. F. & Pattenden, G. Free radical reactions in synthesis. Homolysis of alkylcobalt complexes in the presence of radical-trapping agents. *J. Chem. Soc. Perkin 1*, 2703–2708 (1990).

31. Gollas, B., Krauß, B., Speiser, B. & Stahl, H. Design of a Single-Unit Haber-Luggin Capillary/Dual Reference-Electrode System. *Curr Sep* **13**, 42–44 (1994).

32. Bourbonnais, R., Leech, D. & Paice, M. G. Electrochemical analysis of the interactions of laccase mediators with lignin model compounds. *Biochim. Biophys. Acta BBA - Gen. Subj.* **1379**, 381–390 (1998).

33. Galli, C., Gentili, P., Lanzalunga, O., Lucarini, M. & Pedulli, G. F. Spectrophotometric, EPR and kinetic characterisation of the >N–O• radical from 1-hydroxybenzotriazole, a key reactive species in mediated enzymatic oxidations. *Chem Commun* 2356–2357 (2004).

34. Brandi, P., Galli, C. & Gentili, P. Kinetic Study of the Hydrogen Abstraction Reaction of the Benzotriazole-*N*-oxyl Radical (BTNO) with H-Donor Substrates. *J. Org. Chem.* **70**, 9521–9528 (2005).
